# Multi‐Pathway Consequent Chemoselectivities of CpRuCl(PPh_3_)_2_/MeI‐Catalysed Norbornadiene Alkyne Cycloadditions

**DOI:** 10.1002/chem.201603173

**Published:** 2016-09-13

**Authors:** Wei‐Hua Mu, De‐Cai Fang, Shu‐Ya Xia, Rui‐Jiao Cheng, Gregory A. Chass

**Affiliations:** ^1^ Faculty of Chemistry and Chemical EngineeringYunnan Normal UniversityNO. 298, Yieryi StreetKunming650092P. R. China; ^2^ College of ChemistryBeijing Normal UniversityNO. 19, Xinjiekouwai StreetBeijing100875P. R. China; ^3^ School of Biological and Chemical SciencesQueen Mary University of LondonMile End RoadLondonE1 4NSUK

**Keywords:** chemoselectivity, cycloaddition, norbornadienes, reaction mechanisms, ruthenium

## Abstract

Chemoselectivities of five experimentally realised CpRuCl(PPh_3_)_2_/MeI‐catalysed couplings of 7‐azabenzo‐norbornadienes with selected alkynes were successfully resolved from multiple reaction pathway models. Density functional theory calculations showed the following mechanistic succession to be energetically plausible: (1) CpRuI catalyst activation; (2) formation of crucial metallacyclopentene intermediate; (3) cyclobutene product (**P2**) elimination (Δ*G*_Rel(RDS)_≈11.9–17.6 kcal mol^−1^). Alternative formation of dihydrobenzoindole products (**P1**) by isomerisation to azametalla‐cyclohexene followed by subsequent CpRuI release was much less favourable (Δ*G*_Rel(RDS)_≈26.5–29.8 kcal mol^−1^). Emergent stereoselectivities were in close agreement with experimental results for reactions **a**, **b**, **e**. Consequent investigations employing dispersion corrections similarly support the empirical findings of **P1** dominating in reactions **c** and **d** through **P2**→**P1** product transformations as being probable (Δ*G*≈25.3–30.1 kcal mol^−1^).

## Introduction

Norbornadienes (NBD) and oxa‐ or azanorbornadienes are excellent synthons for preparation of various annulations such as dihydrobenz[g]indoles,^1^ cyclobutenes,^2^ deltacyclenes,^3^ benzonorbornanes,^4^ polynorbornadienes,^5^ epoxynaphthalenes,^6^ dihydronaphthalenes,^7^ diamines^8^ etc., through ring‐openings and reactions with substituted alkynes,^1^, ^2^, ^3^ arenes,^4^ silylacetylenes^6^ and amines.^8^ Ru‐catalysed ring‐opening of such oxa‐ or aza‐norbornadienes show perfect substrate tolerance with moderate to high yields and good to excellent chemo‐, regio‐ and/or stereoselectivities.^9^

**Scheme 1:**
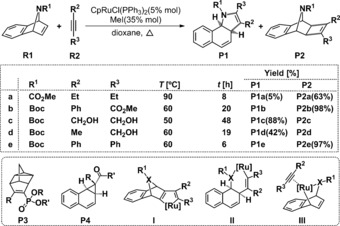
CpRuCl(PPh_3_)_2_/MeI‐catalysed reaction of 7‐azabenzonorbornadienes and selected alkynes characterized in this work (top). Substituent identities, reaction conditions and isolated yields (middle). Structures of other products (**P3**, **P4**) and proposed key intermediates (**I**, **II**, **III**) (bottom).^1^, ^3^, ^10^, ^11^, ^12^, ^13^

With aims of improving reaction efficiency, Tenaglia and Giordano established a novel reaction system in 2003 comprising of norbornadiene with alkynes mediated by a CpRuCl(PPh_3_)_2_/MeI catalyst system (Cp=cyclopentadienyl). The addition of MeI effectively precludes the [2+2+2] cycloaddition product (**P3**, Scheme 1) and significantly shortens reaction times (6 days→8 h), generating cyclobutene derivatives (**P2**) in moderate yields (∼51 %).^10^ This facilitated preparation of numerous benzonorcaradienes^11^ and benzoindoles^12^ from oxa‐ or azabenzonorbornadienes, using the CpRuCl(PPh_3_)_2_/MeI catalyst system. Reaction of oxabenzonorbornadiene with alkynes initiated by CpRuCl(PPh_3_)_2_/MeI in dioxane catalytically and selectively generates benzonorcaradienes (**P4**) as the major product,^11^ whereas reaction of 7‐azabenzonorbornadienes (**R1**) with alkynes (**R2**) offer chemoselective routes to 3a,9b‐dihydrobenzoindoles (**P1**, Scheme 1) or cyclobutene derivatives (**P2**).^12^, 1

To resolve the bases for the observed steroselectivities, we initiated a series of density functional theory (DFT) calculations. A putative mechanism involving the following general steps was formulated: (1) catalyst activation [CpRuCl(PPh_3_)_2_+MeI→CpRuI]; (2) complex formation of a distinct metallacyclopentene intermediate from 7‐azabenzonorbornadienes and alkynes with CpRuI; and (3) cyclobutene product formation (**P2**) by CpRuI elimination. Differing reactive orientations and conformations lead to the chemical transformations following multiple pathways, the details of which are provided in each section of the Results and Discussion and in the relevant figures. An additional pathway involving **P2**→**P1** product isomerisation was also addressed to help resolve experimental observations.

## Results and Discussion

Results emerging from all calculations on reactions **a**–**e** (Scheme 1), are presented in principal sections 3.1 Reaction Mechanisms and 3.2 Chemoselectivities. For clarity of dissemination, these are further partitioned into the following sub‐sections: 3.1.1 In situ catalyst (CpRuI) formation; 3.1.2 Mechanistic specificities: Symmetrical alkynes; 3.1.3 Mechanistic specificities: Unsymmetrical alkynes; 3.2.1 Chemoselectivities of reaction **a**; 3.2.2 Chemoselectivities of reaction **b**; 3.2.3 Chemoselectivities of reaction **c**; 3.2.4 Chemoselectivities of reactions **d** and **e**. The influence of theoretical methods and basis sets are reported in Section 3.3 (Influences of computational method).

### 1 Reaction mechanisms

#### 1.1 In situ catalyst (CpRuI) formation

**Figure 1:**
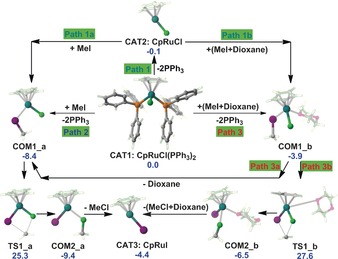
Energetically plausible mechanisms of CpRuI (**CAT3**) formation from CpRuCl(PPh_3_)_2_ and MeI. Relative free energies (kcal mol^−1^) are obtained at IDSCRF‐B3LYP/BS1 level in dioxane solution, at the lowest experimental temperature of 323 K.

Preliminary calculations using the B3LYP method were performed to explore the in situ (dioxane, 323 K) generation of CpRuI (**CAT3**) from pre‐catalysts CpRuCl(PPh_3_)_2_ and MeI. Corresponding mechanisms and free energy results are summarised in Figure 1. Three pathways (Path **1**–**3**) were resolved for the elimination of the two PPh_3_ ligands to form the CpRuCl_MeI complexes (**COM1_a**). Path **1** involves preliminary dissociation of the PPh_3_ groups to form CpRuCl (**CAT2**), releasing 0.1 kcal mol^−1^ free energy. This is followed by subsequent association of MeI (CpRuCl+MeI→CpRuCl_MeI), tested both in the absence (Path **1 a**) and presence (Path **1 b**) of an explicit dioxane solvent molecule, exothermically forming **COM1_a** (Δ*G*_rel_=−8.4 kcal mol^−1^) and **COM1_b** (Δ*G*_rel_=−3.9 kcal mol^−1^), respectively. Paths **2** and **3** comprise concerted and barrierless formations of **COM1_a** and **COM1_b** in the absence (CpRuCl(PPh_3_)_2_+MeI→CpRuCl**_**MeI+2PPh_3_) and presence (CpRuCl(PPh_3_)_2_+MeI+dioxane→CpRuCl**_**MeI[dioxane]+2PPh_3_), respectively, of an explicit dioxane solvent molecule. The latter, 4‐coordinated solvent‐associated Ru is 4.5 kcal mol^−1^ less favourable in free energy than the 3‐coordinated solvent‐free species. The destabilising effect of the explicit solvent molecule persists during the subsequent formation of CpRuI (**CAT3**, Δ*G*_rel_=−4.4 kcal mol^−1^), via **TS1_a** (Δ*G*_rel_= +25.3 kcal mol^−1^, TS=transition state) and **TS1_b** (Δ*G*_rel_= +27.6 kcal mol^−1^) and the post‐reaction complexes **COM2_a** (Δ*G*_rel_=−9.4 kcal mol^−1^) and **COM2_b** (Δ*G*_rel_=−6.5 kcal mol^−1^), followed by elimination of MeCl. The solvent‐associated path **3** lowers spontaneity by an average ∼3.2 kcal mol^−1^ (ΔΔ*G*_rel_=[4.5, 2.3, 2.9]/3). Thus, it is concluded that a dioxane molecule is never directly bound to Ru during the formation of CpRuI (**CAT3**).1

The proposed formation of CpRuI(PPh_3_)_2_ (**CAT4**) from CpRuCl(PPh_3_)_2_ and MeI^10^, ^11^, ^12^ was deemed unlikely due to the associated high free energy barriers, persisting even at 323 K (Δ*G*_rel_=+87.1 kcal mol^−1^, Figure S1(a) in the Supporting Information). Exhaustive attempts involving differing constitutional positioning and conformation torsioning of all molecular species failed to identify more energetically favourable direct routes to **CAT4**. We therefore speculate that the CpRuI(PPh_3_)_2_ species observed by X‐ray is formed along Path **2** by association of two PPh_3_ groups with CpRuI (**CAT3**). This is evidenced as being plausible due to the overall exothermicity of **CAT4** generation (Δ*G*_rel_=−1.5 kcal mol^−1^, Figure S1(b)).

#### 1.2 Mechanistic specificities: Symmetrical alkynes

**Scheme 2:**
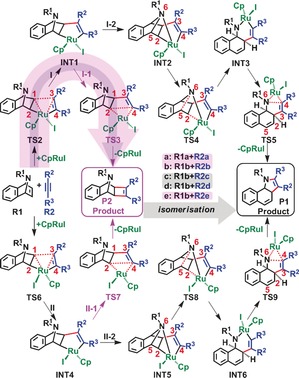
Putative formation mechanisms for dihydrobenzoindoles (**P1**) and cyclobutenes (**P2**) from 7‐azabenzonorbornadiene (**R1**) and symmetric alkynes (**R2 a**, **c**, **e**; Scheme 1), investigated at the IDSCRF‐B3LYP/BS1 level in dioxane solvent.

**Figure 2:**
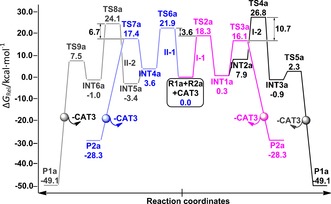
Four competing potential free energy surfaces (Δ*G*_rel_, kcal mol^−1^) for reaction **a**, obtained at the IDSCRF‐B3LYP/BS1 level in dioxane solvent at 363 K.

Mechanistic structural details and resultant spontaneities for reaction **a** are summarised in Scheme 2 and Figure 2, respectively. Therein, two differing pathways (Paths **I** and **II**) were explored in the competitive generation of dihydrobenzoindoles (**P1**) and cyclobutenes (**P2**) from 7‐azabenzonorbornadiene (**R1 a**) and 3‐hexyne (**R2 a**). Paths **I** and **II** differ by disparate orientations of Cp and I groups with respect to the Ru ring plane (Ru‐C1‐C2‐C3‐C4, Scheme 2), and competitive sub‐pathways to **P1** and **P2** are denoted by **I‐1**, **I‐2**, **II‐1**, **II‐2**, respectively. Overall, Path **I** is more spontaneous, with a tendency for the Cp ligand to remain above the Ru ring plane (**TS2 a**= 18.3 kcal mol^−1^), preferred by 3.6 kcal mol^−1^ over the sub‐plane orientation (**TS6 a**=21.9 kcal mol^−1^). This generates Ru‐cyclopentene intermediates (**INT1** and **INT4**) that subsequently isomerise to **INT2** and **INT5** by Ru−N complexation. This is followed by C5−N6 bond breaking via **TS4 a** and **TS8 a**, with barriers of 26.5 and 27.5 kcal mol^−1^, respectively to form ruthenium‐cyclohexene intermediates (**INT3** and **INT6**). This is the rate‐determining step (RDS) for pathways **I‐2** and **II‐2**, and overall, it serves to form the cyclohexene moiety in the benzoindole product (**P1**).2, 2

Alternatively, at **INT1** and **INT4**, the reaction may pursue cyclobutene generation through reductive elimination to produce dihydrobenzoindole, via **TS3 a** and **TS7 a** (affording **P2**) with barriers of 15.8 and 13.8 kcal mol^−1^ for paths **I‐1** and **II‐1**, respectively. Hence, **P2** formation is 10.7 and 13.7 kcal mol^−1^ more spontaneous than **P1** formation, on paths **I** and **II** respectively. This is a reasonable explanation for why 63% of **P2 a** has been isolated experimentally.

Supplementary calculations involving exhaustive attempts to identify possible transition structures and reaction paths to **P1 a** and **P2 a** formation via intermediate **III** (Scheme 1) were all unsuccessful. Searches did afford two structures arising from cleavage of a single C−N linkage in **R1 a** (**TS10 a** and **TS10 ax**), but were prohibitive at 27.2 and 33.4 kcal mol^−1^, respectively (Figure S2 in the Supporting Information). Similar product routes arose for reactions **b**–**e**, raising the applicability of potential routes for future experimental explorations, and are thus discussed below.

#### 1.3 Mechanistic specificities: Unsymmetrical alkynes

**Figure 3:**
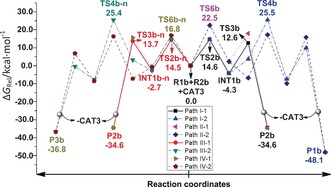
Eight competing potential free energy surfaces (Δ*G*_rel_, kcal mol^−1^) for reaction **b**, obtained at the IDSCRF‐B3LYP/BS1 level in dioxane solvent at 333 K.

Asymmetric substitution of the alkyne results in an additional splitting of the four paths described in reaction **a** for a symmetric alkyne. This forms an octet of paths to be investigated in the competitive generation of **P1 b**, **P2 b** and **P3 b** (**I‐1**, **I‐2**, **II‐1**, **II‐2**, **III‐1**, **III‐2**, **IV‐1**, **IV‐2**). From the outset, Path **I‐1** dominates firstly in the generation of ruthenium‐cyclopentene intermediate (**TS2 b**=14.6 kcal mol^−1^) and subsequently in reductive elimination (Figure 3). Mechanistic dimensionality is immediately reduced through preclusion of the latter two paths (**IV‐1** and **IV‐2**) involving C4−C2 binding (**TS6 b**–**n**, Figure 3 and Scheme S1 in the Supporting Information), due to their being 2.2 kcal mol^−1^ less spontaneous than C3−C1 binding (**TS2 b**, Figure 3 and Scheme 2).3

Although formation of **INT1 b–n** and **INT1 b** are competitive due to near‐identical barriers of **TS2 b–n** (14.5 kcal mol^−1^) and **TS2 b** (14.6 kcal mol^−1^), path **I‐1** is more spontaneous overall, with a maximal barrier of 16.9 kcal mol^−1^ at reductive elimination (**TS3 b**). This **TS3 b** is 1.1 kcal mol^−1^ thermodynamically more favourable than the corresponding **TS3 b–n** on path **III‐1**. Thus, path **I‐1** dominates in the generation of **P2 b** and is in good thermodynamic agreement with experimental yields of 98 %. This differs from Tam's conclusions for dominance of a pathway similar to path **IV‐1**, based on gas‐phase computations using the inferior LAN L2DZ basis set, involving a static general potential to describe all core electrons.^14^

### 2 Chemoselectivities

#### 2.1 Chemoselectivities of reaction **a**

**Figure 4:**
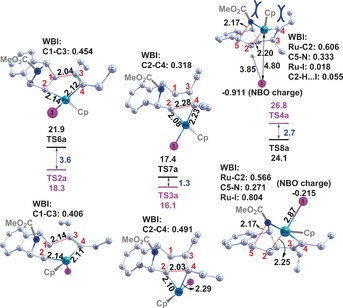
Optimised geometries and corresponding relative free energies for key transition structures in reaction **a**. Selected bond lengths (Å) and corresponding Wiberg bond indices (WBI) are listed. All hydrogens with the exception of those involved in intramolecular interactions are omitted for clarity.

**Figure 5:**
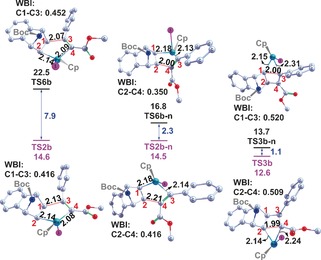
Optimised geometries and corresponding relative free energies for key transition structures in reaction **b**. Selected bond lengths (Å) and corresponding Wiberg bond indices (WBI) are listed. All hydrogens are omitted for clarity.

To resolve the structural bases for the observed chemoselectivities and corresponding energetics, key structures and their corresponding Wiberg bond indices (WBI) along **P2 a** and **P2 b** formation pathways for reactions **a** and **b** are presented in Figures 4 and 5, respectively. The reduced spontaneity of surmounting the **TS6 a** relative to **TS2 a** barriers, detailed in section 3.1.2, renders the contribution of path **II‐1** to **P2 a** production negligible and is attributed to a later transition state. This is evidenced by both the shorter _(cat‐complex)_C1−C3_(product)_ bond length (2.04 vs. 2.14 Å) and bigger Wiberg bond index (WBI) (i.e., stronger bonding, as per WBI≈0.454 vs. 0.406) of **TS6 a**, relative to those of **TS2 a**. The inverse is observed in the subsequent step, in which the earlier transition structure of **TS7 a** raises its free energy by 1.3 kcal mol^−1^ relative to that of **TS3 a**, further hampering **P2 a** formation along path **II‐1**. This is evidenced by the longer (2.28 vs.2.03 Å) and correspondingly weaker _(cat‐complex)_C2−C4_(product)_ bond (i.e., smaller WBI≈0.318 vs. 0.491), relative to that in **TS3 a**.4, 5

Crowding around Ru by the Cp, Et and CO_2_Me groups is alleviated by transfer of electronic density to the I atom, facilitating its departure. This is evidenced by a natural bond order (NBO) charge of −0.911 ē and the decreasing WBI of the Ru−I linkage at the **INT1 a**→**TS4 a** step (0.780→0.018) (see Figure S3 in the Supporting Information). This step is 10.7 kcal mol^−1^ less spontaneous than the path via **TS3 a**, effectively making path **I‐2** (thus **P1 a** production) improbable in this manner. Similarly, **P1 a** production by path **II‐2** is less probable than its corresponding path **II‐1**, with the **INT5 a**→**TS8 a** RDS step for the former being 13.7 kcal mol^−1^ less spontaneous than the RDS of the latter with **INT4 a**→**TS7 a**. These trends support our proposal for path **I‐1** dominance in the observed 63 % yield of **P2 a** at 363 K (Scheme 1).

#### 2.2 Chemoselectivities of reaction **b**

For reaction **b**, similar structure‐spontaneity trends to those in reaction **a** support the observed predominance for **P2 b** formation, with predominance of path **I‐1** and additional contributions from path **III‐1**, since the free energy barrier of **TS3 b–n** (16.4 kcal mol^−1^) is competitive with that of **TS3 b**. Paths **II‐1** and **IV‐1** are avoided due to their higher barriers at **TS6 b** and **TS6 b–n**, effectively slowing down reactions along these paths by 1.5×10^5^ and 28 times, respectively, with respect to path **I‐1** (kinetic calculations detailed in Figures 3 and S4 in the Supporting Information). The 7.9 kcal mol^−1^ reduction in spontaneity of **TS6 b**, relative to **TS2 b**, arises from its later transition structure; this is evidenced by its contracted _(cat‐complex)_C1−C3_(product)_ linkage (2.07 Å vs. 2.13 Å) and correspondingly larger WBI (0.452 vs. 0.416). The situation is similar for **TS6 b–n** and **TS2 b–n**, wherein the latter has an extended _(cat−complex)_C2−C4_(product)_ bond length (2.00 Å vs. 2.21 Å) and correspondingly larger WBI (0.350 vs. 0.416) (Figure 5).

Confidence in the spontaneity‐bonding‐Wiberg trends and the crucial product stoichiometry role of the atomic cohesion of C1−C3 and C2−C4 linkages is further procured by an identical WBI value (0.416) for the C1−C3 and C2−C4 linkages in **TS2 b** and **TS2 b–n**. This translates to a negligible free energy difference of 0.1 kcal mol^−1^ (Figures 3 and 5). Similarly for **TS3 b** and **TS3 b–n**, for which the C1−C3 and C2−C4 WBI values are 0.509 and 0.520, respectively, correspondingly bear near‐identical free energy barriers of 12.6 and 13.7 kcal mol^−1^, respectively (Figures 3 and 5).

#### 2.3 Chemoselectivities of reaction **c**

Results for reaction **c** are presented in Figure S5 (Supporting Information). Path **II** is avoided due the reduced spontaneity of **TS6 c** relative to that of **TS2 c** along path **I** (9.6 vs. 2.8 kcal mol^−1^). An 88.0 % yield of **P1 c** was observed experimentally (Scheme 1), without any **P2 c** formed. However, computations predict **P2 c** dominance if only paths of types **I** and **II** are considered, due to avoidance of **TS4 c** (path **I‐2**, 27.3 kcal mol^−1^) and a 9.7 kcal mol^−1^ free energy preference for **TS3 c** (path **I‐1**, 17.6 kcal mol^−1^). This highlights the importance of alternative path calculations.

**Scheme 3:**
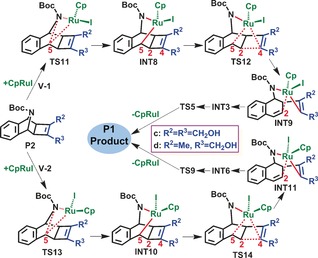
Putative transformation mechanisms of cyclobutenes (**P2**) into dihydrobenzoindoles (**P1**) in reactions **c** and **d**, under the help of CpRuI, as determined at the IDSCRF‐B3LYP/BS1 level in dioxane solvent.

The overwhelming thermodynamic stability of **P1 c** (−60.2 kcal mol^−1^), relative to that of **P2 c** (−35.1 kcal mol^−1^), hinted at the possibility of a kinetic→thermodynamic product transformation pathway. A novel type of path (**V**) involving the CpRuI catalyst oxidatively inserting into a C−N bond on the **P2 c** product, was thus tested to rectify this experimental–computational discord (Scheme 3 and Figure S6 in the Supporting Information). Differing constitutional orientations of Cp and I ligands divides the path into two differing channels (**V‐1** and **V‐2**). The initial oxidative insertion step is locally demanding at 41.0 and 40.8 kcal mol^−1^, for **TS11 c** and **TS13 c**, respectively. However, these barriers are globally surmountable due to sufficient energy remaining in the reaction ensemble relative to the original starting materials; the transformations are +5.9 and +5.7 kcal mol^−1^, relative to the starting materials, respectively. Subsequently, the _(cat‐complex)_C2−C4_(product)_ bond is cleaved at **TS12 c** and **TS14 c**, (31.5 and 10.9 kcal mol^−1^, respectively), the latter providing a possible route to **P1 c** dominance, although the oxidative insertion remains prohibitive.3

**Figure 6:**
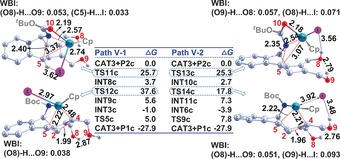
Optimised geometries and corresponding relative free energies for key transition structures along path **V** of reaction **a** (obtained at IDSCRF‐B3LYP+D3/BS1 level in dioxane solvent at 323 K). Selected bond lengths (Å) and corresponding Wiberg bond indices (WBI) are listed. All hydrogens with the exception of those involved in intramolecular interactions are omitted for clarity.

Further examination of the structures along these pathways revealed weak O−H⋅⋅⋅O and O−H⋅⋅⋅I interactions, which are specific to reaction **c** (Figure S7 in the Supporting Information) and are poorly described by the B3LYP method.^15^ Subsequent calculations employing the dispersion‐corrective B3LYP+D3 method rendered the oxidative insertion barriers to be manageable values of 25.7 (**TS11 c**) and 25.3 kcal mol^−1^ (**TS13 c**). More encouraging was the reduction of the **TS14 c** barrier to 15.1 kcal mol^−1^, allowing the C2−C4 linkage to be easily cleaved at 323 K and support **P1 c** dominance (Figure 6). Once again a spontaneity‐Wiberg correlation is apparent, with the O8−H⋅⋅⋅O9 and O9−H⋅⋅⋅O8 connections in **TS11 c** and **TS13 c** having near‐identical WBI values of 0.053 versus 0.057. This indicates that the barrier lowering in the latter TS arises from elsewhere in the transition structures. Indeed, the WBI of the C5−H⋅⋅⋅I interaction in **TS11 c** (0.033), is half that of the O8−H⋅⋅⋅I link in **TS13 c** (0.071); this relatively strong hydrogen‐halide interaction is responsible for the barrier elevation. Similarly, **TS14 c** has an advantage with the O8−H⋅⋅⋅O9 and O9−H⋅⋅⋅I intramolecular interactions, helping effect its observed spontaneity and manageable barrier at the B3LYP+D6 level.

Overall, the B3LYP+D3 results recover the experimental‐computational agreement, through its resolution of the product stereoselectivity (two bridged *cis* hydrogens) observed in most experiments.^1^, ^10^, ^12^, ^13^ Kinetic calculations from the RDS barriers in reaction **c**, further strengthens the agreement with the time for the entire transformation anticipated at ∼48 h (contrasted to the 8 and 20 h for reactions **a** and **b,** respectively), again in agreement with experimental trends.

#### 2.4 Chemoselectivities of reactions d and e

**Figure 7:**
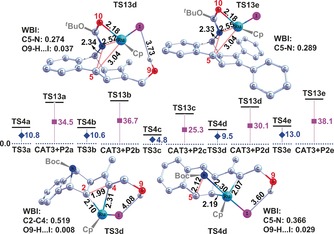
Relative free energies (kcal mol^−1^) of key transition structures in reactions **a**–**e**, with selected interatomic distances (in Å). All hydrogen atoms apart from those of OH groups are omitted for clarity.

Reaction mechanisms and profiles for reactions **d** and **e** are similar to those presented for reaction **c**, with **P1 d** and **P2 e** predicted as the corresponding major products in each case, in agreement with experimental observations of 42 % and 97 %, respectively (Scheme 1). **P1 d** emerges from path **I‐1** generation of **P2 d** and eventual **P2 d**→**P1 d** conversion along a type **V** pathway, whereas **P1 e** forms along a **I‐1** path only. Complete free energy profiles for reactions **d** and **e** are presented in Figures S8, S9, S10 and S11 (Supporting Information). Key steps of these transformations are compared in Figure 7, right side, against their matching transitions in reactions **a**–**c**. The RDSs on path type **V** for reactions **d** and **e** emerge as **TS13 d** and **TS13 e**, mediated by barriers of +30.1 and +38.1 kcal mol^−1^, respectively. The former supporting the 42 % **P1 d** product yield experimentally observed at 333 K over 19 h, the latter effectively blocking **P1 e** generation.7

### 3 Influence of computational methods

In determining the probability of **P2 a(b, e)**→**P1 a(b, e)** transformation, by paths **V‐1** or **V‐2**, and to more fully explore the influence of the method on relative free energies, calculations employing both B3LYP and B3LYP+D3 functionals were carried out for reactions **a** and **b**. The results in dioxane solvent are summarised in Tables 1, 2 and S1. Corresponding results for reactions **c**–**e** are also listed for sake of comparison. It is clear that path **V‐1** is more difficult to overcome than **V‐2** in all five reactions, featured by higher free energy barriers ranging from 37.6 to 43.0 kcal mol^−1^ at the IDSCRF‐B3LYP+D3/BS1 computational level and even higher free energy barriers at the IDSCRF‐B3LYP/BS1 computational level (ranging from 55.0 to 63.8 kcal mol^−1^). When the B3LYP method is employed, the activation free energy barriers corresponding to RDS in path **V‐2** (**TS13**) is predicted to be 48.7, 53.4, 40.8, 45.2, and 56.1 kcal mol^−1^ respectively for reactions a–e, suggesting no detectable transformation from **P2** to **P1** and this does not support the predominant formation of **P1 c** and **P1 d**.1, 2

**Table 1:** Relative free energies (kcal mol^−1^) of stationary points on path **V‐2** for reactions **a**–**e**, obtained at the IDSCRF‐B3LYP+D3/BS1 level in dioxane solvent, at experimental temperatures.^[a]^

	Reaction (temperature)				
Reaction step	**a** (363 K)	**b** (333 K)	**c** (323 K)	**d** (333 K)	**e** (333 K)
**P2+CAT3**	0.0	0.0	0.0	0.0	0.0
**TS13**	34.5	36.7	25.3	30.1	38.1
**INT10**	9.2	9.7	2.7	7.1	12.2
**TS14**	25.2	32.2	17.8	20.2	31.0
**INT11**	14.3	18.4	7.3	12.1	21.6
**INT6**	10.3	5.5	‐3.9	1.4	8.2
**TS9**	16.1	28.6	7.8	11.1	20.0
**P1+CAT3**	−23.6	−17.7	−27.9	−26.9	−19.6
***k***	1.282×10^−8^	5.692×10^−12^	5.125×10^−5^	1.221×10^−7^	6.862×10^−13^
***t***_**1/2**_	2.167×10^4^	4.880×10^7^	0.542×10^1^	2.275×10^3^	4.048×10^8^

[a] Rates (*k*, L mol^−1^ s^−1^) and half‐lives (*t*_1/2_, h) were determined from the free energy barriers of the RDS (**TS13**), at the IDSCRF‐B3LYP+D3/BS1 level.

**Table 2:** Relative free energies (kcal mol^−1^) of stationary points on path **V‐2** for reactions **a**–**e**, obtained at the IDSCRF‐B3LYP/BS1 level in dioxane solvent, at experimental temperatures.

	Reactio**n** (temperature)				
Reaction step	**a** (363 K)	**b** (333 K)	**c** (323 K)	**d** (333 K)	**e** (333 K)
**P2+CAT3**	0.0	0.0	0.0	0.0	0.0
**TS13**	48.7	53.4	40.8	45.2	56.1
**INT10**	25.9	29.4	20.9	23.9	31.8
**TS14**	41.6	49.9	31.8	34.7	50.8
**INT11**	32.9	37.0	26.4	30.0	42.6
**INT6**	27.3	24.7	15.5	19.2	29.5
**TS9**	35.8	50.4	24.5	28.9	42.4
**P1+CAT3**	−20. 7	−13.5	−25.1	−24.2	−14.5
***k***	3.619×10^−17^	6.239×10^−23^	1.668×10^−15^	1.502×10^−17^	1.055×10^−24^
***t***_**1/2**_	7.675×10^12^	4.452×10^18^	1.665×10^11^	1.849×10^13^	2.634×10^20^

The B3LYP+D3 method does generate reduced RDS (**TS13**) free energy barriers of 34.5, 36.7, 25.3, 30.1 and 38.1 kcal mol^−1^ for reactions **a**–**e**, respectively, aligning well with the experimentally observed yields of **P1**. For reactions **b** and **e**, their corresponding half‐lives of 4.880×10^7^ h (ca. 5571 years) and 4.048×10^8^ h (ca. 46210 years) nullify any corresponding **P2→P1** conversion via **TS13**; in agreement with experimental data. For reaction **a**, its lower **TS13** barrier is perhaps surmountable under the reaction conditions, providing a route to the ∼5 % isolated yield of **P1 a**. For H‐bound systems (**c** and **d**), these RDS free energy reductions correspond to representative half‐lives of 5.42 and 2.275×10^3^ h (ca. 3 months) and their experimentally observed chemoselectivities of 88 % and 42 %, respectively. The latter time of 3 months is within one order of magnitude (ca. 1.50 kcal mol^−1^) of the experimental reaction times of 6 days. Thus, **P2→P1** through path **V‐2** seems possible for reaction **a**, probable for reaction **c** and, at least to some extent, also so for reaction **d**. In summary, it is deemed necessary to include dispersion correction on the B3LYP method for systems demonstrating weak interactions.^15^, ^16^

## Conclusion

Density functional theory (DFT) calculations employing the IDSCRF‐B3LYP and IDSCRF‐B3LYP+D3 methods, with 2 differing basis sets, have been performed to probe the experimentally observed chemoselectivities of five CpRuCl(PPh_3_)_2_/MeI catalyzed cycloaddition reactions. The following conclusions can be drawn:

1. Multiple reaction pathways emerging from differing constitutional arrangements and conformations must be characterised in order to reproduce experimentally observed stoichiometries. Characterisation of the contributions of each pathway to (or justifying their exclusions from) the overall reaction path‐ensembles is crucial for raising confidence levels of the interpretation of the observed chemoselectivities.
2. The catalytic cycle begins with direct formation of CpRuI from CpRuCl(PPh_3_)_2_ and MeI precursors, then generation of a crucial five‐membered metallacyclopentene (**INT1**) with free energy barriers of 18.3 and 20.8 kcal mol^−1^ for reactions **a** and **e**, respectively. **INT1** generation serves as the RDS in the production of cyclobutene products in reactions **a** and **e**. Reductive elimination, with a barrier of 16.9 kcal mol^−1^, serves as the RDS for reaction **b**. In these three reactions, the competing pathways to dihydrobenzoindole production via **TS4** are effectively blocked by barriers of 26.5, 29.8 and 27.6 kcal mol^−1^ for reactions **a**, **b** and **e**, respectively.
3. Cyclobutene products in reactions **c** and **d** isomerise to the competing dihydrobenzoindole products through the cleavage of a C−N bond in 7‐azabenzonorbornadienes. This serves as the RDS in these processes, with barriers of 25.3 (**c**) and 30.1 kcal mol^−1^ (**d**).
4. Poorer agreement of chemoselectivities emerge from the B3LYP method for systems displaying contingent H‐bonding (i.e., reactions **c**, **d**), although the results are structurally informative and afford qualitative energetic ordering of reaction steps. To recover experimentally observed chemoselectivities, methods incorporating dispersion corrections, such as within B3LYP+D3, should be employed.

Our work has depicted the importance of manifold mechanisms to accurately and reproducibly resolve experimental chemoselectivities of azabenzonorbornadienes and alkynes. This work has led to complementary explorations of reactive regioselectivites of unsymmetrical alkynes, as well as the diastereoselective formation of dihydrobenzoindoles in related systems.

## Computational Methods

All models involved the full‐sized systems (i.e., no truncations) to accurately represent the real chemical transformations under investigation. Stable structures along the mechanistic profiles were initially optimised, their identities verified and relative free energies determined in solvent (see below) using the B3LYP method, as implemented in Gaussian 09 Program Package (G09),^17^ employing a basis set labelled ‘BS1’ for convenience. BS1 employs the 6‐31G(d,p) Pople basis set^18^ for C, H, O, N atoms and the standard double‐ζ valence polarized (DZVP) all‐electron basis set for the Ru atom.^19^ For the I atom, diffuse 1 s, 1 p and 1 d functions, taken from the aug‐cc‐pVTZ‐PP basis set,^20^ have been added to the standard 6‐311G(d) basis set.^21^ A second basis set combination, labelled ‘BS2’, differing from BS1 only in its use of the 6‐311++G(d,p) Pople basis set for C, H, O, N atoms, was also employed for selected computations. Experimental solvent effects (dioxane, *ϵ*=2.21), were addressed using the default self‐consistent reaction field (SCRF) polarisable continuum model (PCM),^22^ employing IDSCRF atomic radii^23^ to define the molecular cavity; denoted IDSCRF‐B3LYP.

All free energies reported throughout the work have been corrected to include translational entropy contributions in the condensed phase (*S*_trans(l)_) using the THERMO method^24^ towards avoiding the pitfalls associated with default gas‐phase calculations of *S*_trans_ originating from *S*_trans(g)_. Intrinsic reaction coordinate (IRC)^25^ calculations were carried out on selected reaction pathways to confirm key transition states (TSs) and connect two corresponding adjacent minima.

Furthermore, the dispersion‐corrected DFT‐D^26^ method (denoted IDSCRF‐B3LYP+D3) was chosen to characterise selected stationary points and reaction channels when necessary. NBO analyses,^27^ as implemented in G09, was also performed on selected stationary points at IDSCRF‐B3LYP/BS2//BS1 or IDSCRF‐B3LYP+D3/BS2//BS1 level, to investigate their electronic properties and bonding characteristics.

To dispel the spectres of methodological uncertainty and anomaly in the B3LYP results, single‐point energies using the more modern M062X, X3 LYP and CAM‐B3LYP functionals, as well as MP2 and B2PLYP methods were carried out on selected paths in reaction **a**; these are presented in Table S2 in the Supporting Information. For concision, only B3LYP or B3LYP+D3 results are discussed in the text.

## Supporting information

As a service to our authors and readers, this journal provides supporting information supplied by the authors. Such materials are peer reviewed and may be re‐organized for online delivery, but are not copy‐edited or typeset. Technical support issues arising from supporting information (other than missing files) should be addressed to the authors.

Supplementary
