# Supplementary material for: Multi‐Pathway Consequent Chemoselectivities of CpRuCl(PPh3)2/MeI‐Catalysed Norbornadiene Alkyne Cycloadditions
Source: Chemistry. 2016 Sep 13;22(43):15396–403. doi: 10.1002/chem.201603173 (PMC5082545; doi:10.1002/chem.201603173)
Supplement: Supplementary file 1 — Supplementary [file CHEM-22-15396-s001.pdf]

CHEMISTRY
A European Journal

Supporting Information

Multi-Pathway Consequent Chemoselectivities of CpRuCl(PPh_3)$_2$/MeI-Catalysed Norbornadiene Alkyne Cycloadditions

Wei-Hua Mu,*[a] De-Cai Fang,[b] Shu-Ya Xia,[a] Rui-Jiao Cheng,[a] and Gregory A. Chass*[c]

chem_201603173_sm_miscellaneous_information.pdf

Contents List	Page
**Figure S1**. Proposed formation mechanisms of CpRuI (**CAT3**) from $CpRuCl(PPh_3)_2$ and MeI, with corresponding relative free energies (kcal·mol^{-1}) obtained at IDSCRF-B3LYP/BS1 level in dioxane solution, 323K.	3
**Figure S2**. Free energy profiles and optimized structures (bond length in Å) corresponding to the C-N bond cleavage process through **TS10a** and **TS10ax**, obtained at IDSCRF-B3LYP/BS1 level in dioxane solution, 363K. All hydrogens are omitted for clarity.	3
**Scheme S1**. Alternative plausible formation routes of **P1b** and **P3b**, located at IDSCRF-B3LYP/BS1 level in dioxane solvent.	4
**Figure S3**. Optimised geometry for **INT1a**. Selected bond lengths (Å) and corresponding Wiberg bond indices (WBI) are listed. All hydrogens are omitted for clarity.	4
**Figure S4**. Potential free energy surfaces (PESs) corresponding to Path **I-IV** in reaction **b**, obtained at the IDSCRF-B3LYP/BS1 level in dioxane solvent at 333 K.	5
**Figure S5**. Potential free energy surfaces (PESs) corresponding to Path **I** and **II** in reaction **c**, obtained at the IDSCRF-B3LYP/BS1 level in dioxane solvent at 323 K.	5
**Figure S6**. Potential free energy surfaces (PESs) corresponding to Path **V** in reaction **c**, obtained at the IDSCRF-B3LYP/BS1 level in dioxane solvent at 323 K.	6
**Figure S7**. Optimized structures (bond length in Å) of selected stationary points in reaction **c**, obtained at the IDSCRF-B3LYP/BS1 level in dioxane solvent. All hydrogens with the exceptions of those involved in intra-molecular interactions are omitted for clarity.	6
**Figure S8**. Potential free energy surfaces (PESs) corresponding to Path **I** and **II** in reaction **d**, obtained at IDSCRF-B3LYP/BS1 levels in dioxane solvent at 333 K.	7
**Figure S9**. Potential free energy surfaces (PESs) corresponding to Path **V** in reaction **d**, obtained at IDSCRF-B3LYP+D3/BS1 level in dioxane solvent at 333 K.	7
**Figure S10**. Potential free energy surfaces (PESs) corresponding to Path **I** and **II** in reaction **e**, obtained at the IDSCRF-B3LYP/BS1 level in dioxane solvent at 333 K.	8
**Figure S11**. Potential free energy surfaces (PESs) corresponding to Path **V** in reaction **e**, obtained at IDSCRF-B3LYP+D3/BS1 level in dioxane solvent at 333 K.	8
**Table S1.** Relative free energies of stationary points on Path **V-I** of reaction **a**-**e**, obtained at both IDSCRF-B3LYP+D3/BS1 and IDSCRF-B3LYP/BS1 levels in dioxane solvent, at experimental temperatures.	9
**Table S2**. Single-point free energies obtained at the IDSCRF-M062X//B3LYP/BS1, IDSCRF-X3LYP//B3LYP/BS1, CAM-B3LYP//B3LYP/BS1, MP2(Full)//B3LYP/BS1 and B2PLYP(Full)//B3LYP/BS1 computational levels for selected paths in reaction **a** under 363 K, denoted as M062X, X3LYP, CAM-B3LYP, MP2 and B2PLYP respectively. Single-point free energies obtained at IDSCRF-B3LYP//BS3/BS1 level (363 K, denated as B3LYP//BS3/BS1[a]) are also listed for further comparison.	9
**Table S3**.The optimized cartesian coordinates (Å) for CpRuI (**CAT3**)'s formation process, located at IDSCRF-B3LYP/BS1 level in dioxane solvent.	10
**Table S4**.The optimized cartesian coordinates (Å) for reaction **a**, located at IDSCRF-B3LYP/BS1 level in dioxane solvent.	14
**Table S5**.The optimized cartesian coordinates (Å) for reaction **b**, located at IDSCRF-B3LYP/BS1 level in dioxane solvent.	25
**Table S6**.The optimized cartesian coordinates (Å) for reaction **c**, located at IDSCRF-B3LYP/BS1 level in dioxane solvent.	44
**Table S7**.The optimized cartesian coordinates (Å) for reaction **d**, located at IDSCRF-B3LYP/BS1 level in dioxane solvent.	55
**Table S8**.The optimized cartesian coordinates (Å) for reaction **e**, located at IDSCRF-B3LYP/BS1 level in dioxane solvent.	66
**Table S9**. The first three vibrational frequencies for stationary points corresponding to the formation process of **CAT3**, located at IDSCRF-B3LYP/BS1 level in dioxane solvent.	79
**Table S10.** The first three vibrational frequencies for stationary points of reaction **a**, located at IDSCRF-B3LYP/BS1 level in dioxane solvent.	79

**Table S11**. The first three vibrational frequencies for stationary points of reaction **a**, located at IDSCRF-B3LYP+D3/BS1 level in dioxane solvent.	79
**Table S12**. The first three vibrational frequencies for stationary points of reaction **b**, located at IDSCRF-B3LYP/BS1 level in dioxane solvent.	79
**Table S13**. The first three vibrational frequencies for stationary points of reaction **b**, located at IDSCRF-B3LYP+D3/BS1 level in dioxane solvent.	80
**Table S14**. The first three vibrational frequencies for stationary points of reaction **c**, located at IDSCRF-B3LYP/BS1 level in dioxane solvent.	80
**Table S15.** The first three vibrational frequencies for stationary points of reaction **c**, located at IDSCRF-B3LYP+D3/BS1 level in dioxane solvent.	80
**Table S16**. The first three vibrational frequencies for stationary points of reaction **d**, located at IDSCRF-B3LYP/BS1 level in dioxane solvent.	80
**Table S17**. The first three vibrational frequencies for stationary points of reaction **d**, located at IDSCRF-B3LYP+D3/BS1 level in dioxane solvent.	80
**Table S18.** The first three vibrational frequencies for stationary points of reaction **e**, located at IDSCRF-B3LYP/BS1 level in dioxane solvent.	81
**Table S19**. The first three vibrational frequencies for stationary points of reaction **e**, located at IDSCRF-B3LYP+D3/BS1 level in dioxane solvent.	81
**Table S20.** The total energies (*E*: a.u.), zero-point energies (*ZPE*: kcal·mol^{-1}) and Gibbs free energies [*G* and *G*(sol,323K): a.u.] for stationary points corresponding to the formation process of **CAT3**, located at IDSCRF-B3LYP/BS1 level in dioxane solvent.	81
**Table S21.** The total energies (*E*: a.u.), zero-point energies (*ZPE*: kcal·mol^{-1}) and Gibbs free energies [*G* and *G*(sol,363K): a.u.] for stationary points of reaction **a**, located at IDSCRF-B3LYP/BS1 level in dioxane solvent.	81
**Table S22.** The total energies (*E*: a.u.), zero-point energies (*ZPE*: kcal·mol^{-1}) and Gibbs free energies [*G* and *G*(sol,363K): a.u.] for stationary points of reaction **a**, located at IDSCRF-B3LYP+D3/BS1 level in dioxane solvent.	82
**Table S23**. The total energies (*E*: a.u.), zero-point energies (*ZPE*: kcal·mol^{-1}) and Gibbs free energies [*G* and *G*(sol,333K): a.u.] for stationary points of reaction **b**, located at IDSCRF-B3LYP/BS1 level in dioxane solvent.	83
**Table S24**. The total energies (*E*: a.u.), zero-point energies (*ZPE*: kcal·mol^{-1}) and Gibbs free energies [*G* and *G*(sol,333K): a.u.] for stationary points of reaction **b**, located at IDSCRF-B3LYP+D3/BS1 level in dioxane solvent.	84
**Table S25**. The total energies (*E*: a.u.), zero-point energies (*ZPE*: kcal·mol^{-1}) and Gibbs free energies [*G* and *G*(sol,323K): a.u.] for stationary points of reaction **c**, located at IDSCRF-B3LYP/BS1 level in dioxane solvent.	84
**Table S26**. The total energies (*E*: a.u.), zero-point energies (*ZPE*: kcal·mol^{-1}) and Gibbs free energies [*G* and *G*(sol,323K): a.u.] for stationary points of reaction **c**, located at IDSCRF-B3LYP+D3/BS1 level in dioxane solvent.	85
**Table S27.** The total energies (*E*: a.u.), zero-point energies (*ZPE*: kcal·mol^{-1}) and Gibbs free energies [*G* and *G*(sol,333K): a.u.] for stationary points of reaction **d**, located at IDSCRF-B3LYP/BS1 level in dioxane solvent.	86
**Table S28**. The total energies (*E*: a.u.), zero-point energies (*ZPE*: kcal·mol^{-1}) and Gibbs free energies [*G* and *G*(sol,333K): a.u.] for stationary points of reaction **d**, located at IDSCRF-B3LYP+D3/BS1 level in dioxane solvent.	86
**Table S29**. The total energies (*E*: a.u.), zero-point energies (*ZPE*: kcal·mol^{-1}) and Gibbs free energies [*G* and *G*(sol,333K): a.u.] for stationary points of reaction **e**, located at IDSCRF-B3LYP/BS1 level in dioxane solvent.	87
**Table S30**. The total energies (*E*: a.u.), zero-point energies (*ZPE*: kcal·mol^{-1}) and Gibbs free energies [*G* and *G*(sol,333K): a.u.] for stationary points of reaction **e**, located at IDSCRF-B3LYP+D3/BS1 level in dioxane solvent.	88

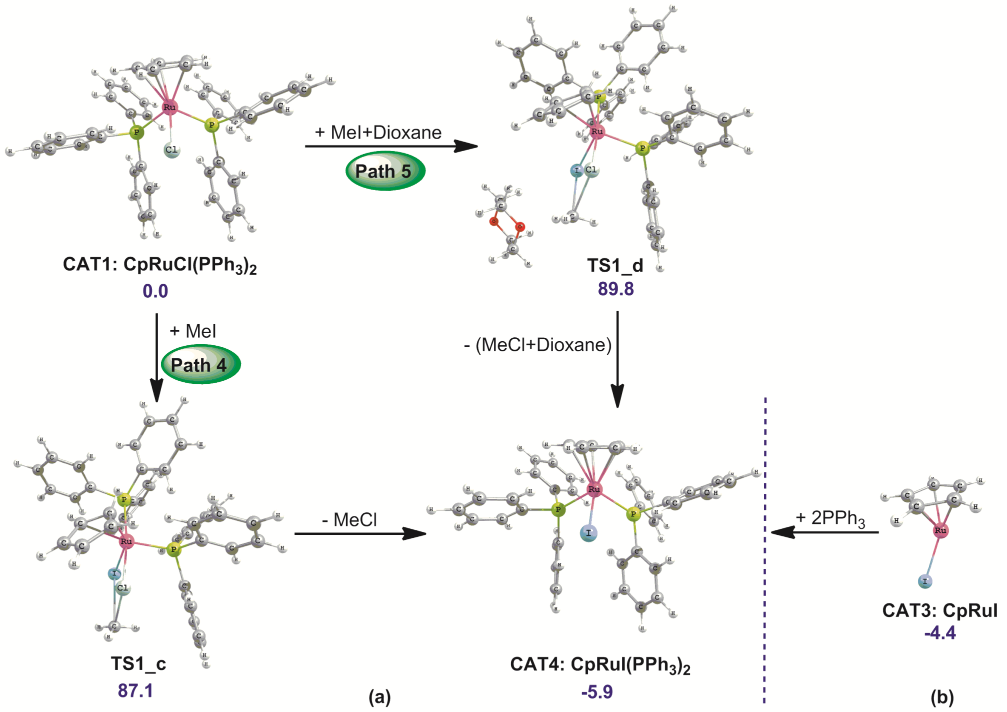

Figure S1. Proposed formation mechanisms of CpRuI (**CAT3**) from $CpRuCl(PPh_3)_2$ and MeI, with corresponding relative free energies ($kcal \cdot mol^{-1}$) obtained at IDSCRF-B3LYP/BS1 level in dioxane solution, 323K.

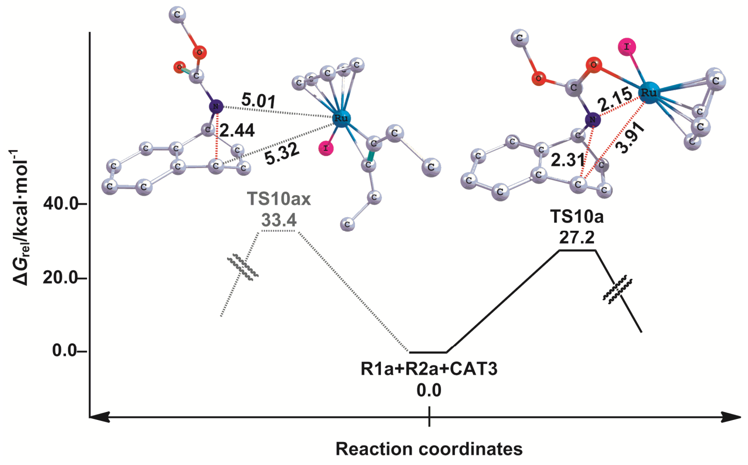

Figure S2. Free energy profiles and optimized structures (bond length in Å) corresponding to the C-N bond cleavage process through **TS10a** and **TS10ax**, obtained at IDSCRF-B3LYP/BS1 level in dioxane solution, 363K. All hydrogens are omitted for clarity.

Scheme S1. Alternative plausible formation routes of **P1b** and **P3b**, located at IDSCRF-B3LYP/BS1 level in dioxane solvent.

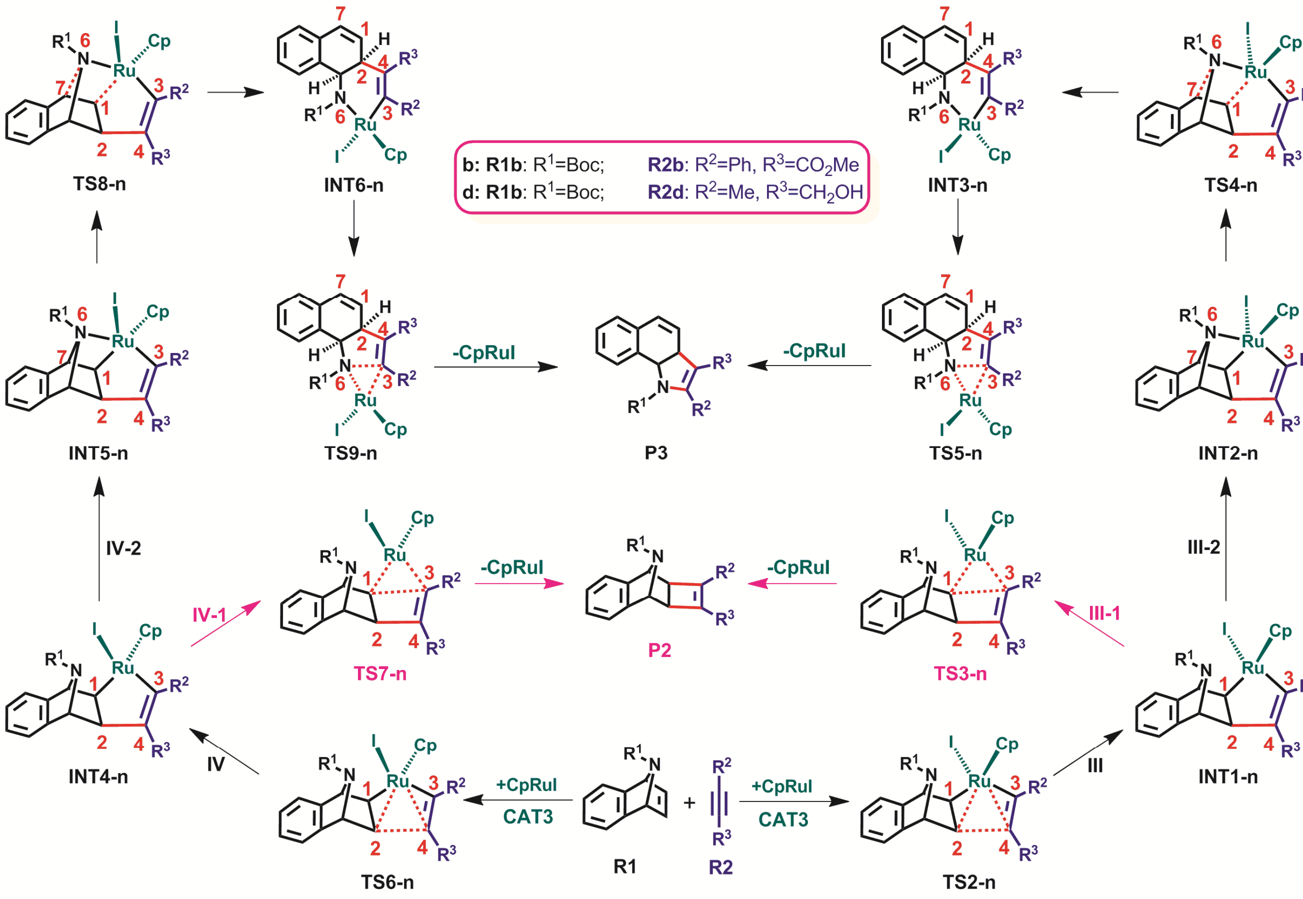

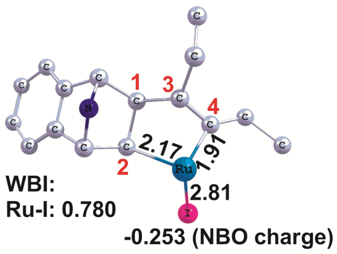

INT1a

Figure S3. Optimised geometry for **INT1a**. Selected bond lengths (Å) and corresponding Wiberg bond indices (WBI) are listed. All hydrogens are omitted for clarity.

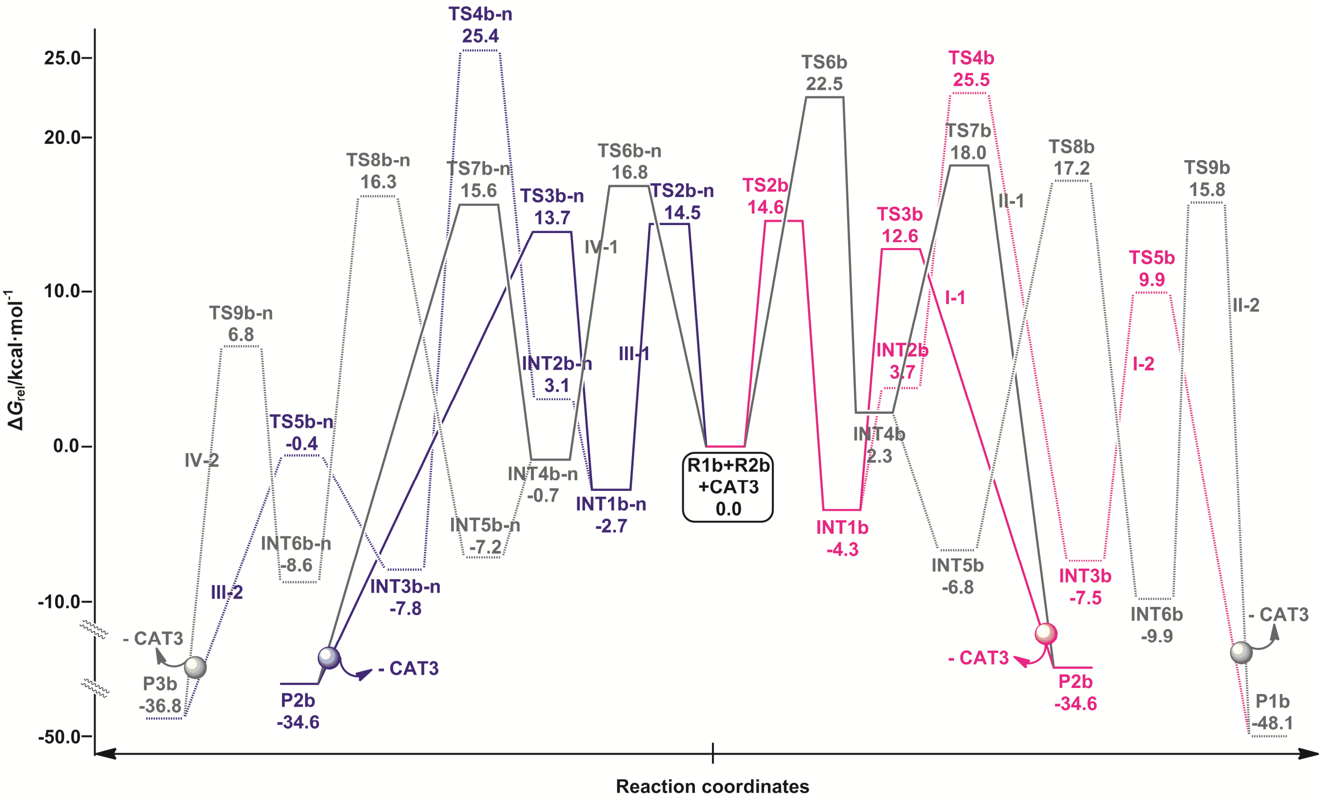

Figure S4. Potential free energy surfaces (PESs) corresponding to Path **I-IV** in reaction **b**, obtained at the IDSCRF-B3LYP/BS1 level in dioxane solvent at 333 K.

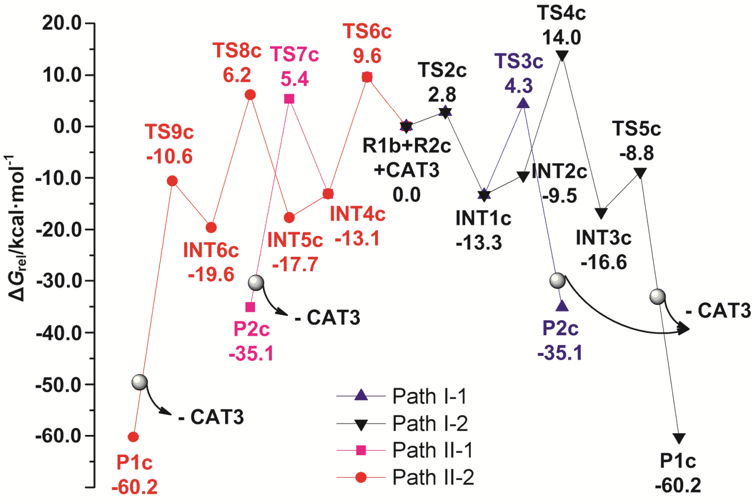

Figure S5. Potential free energy surfaces (PESs) corresponding to Path **I** and **II** in reaction **c**, obtained at the IDSCRF-B3LYP/BS1 level in dioxane solvent at 323 K.

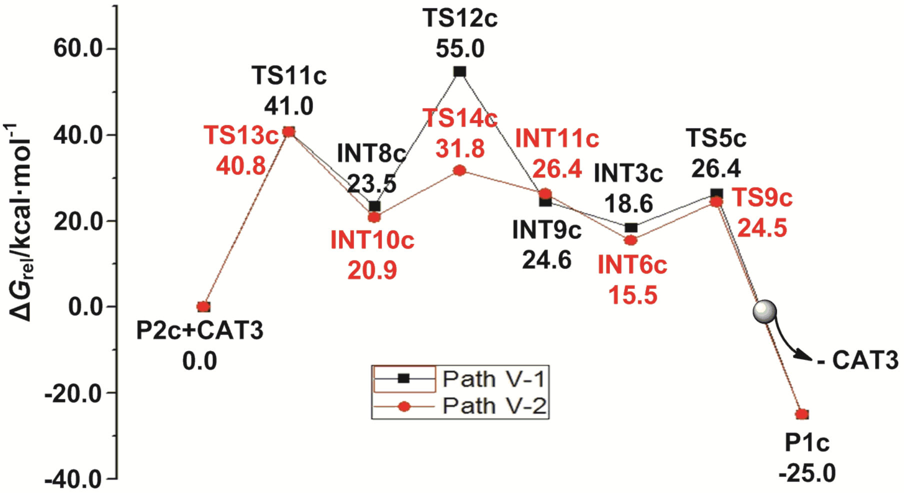

Figure S6. Potential free energy surfaces (PESs) corresponding to Path **V** in reaction **c**, obtained at the IDSCRF-B3LYP/BS1 level in dioxane solvent at 323 K.

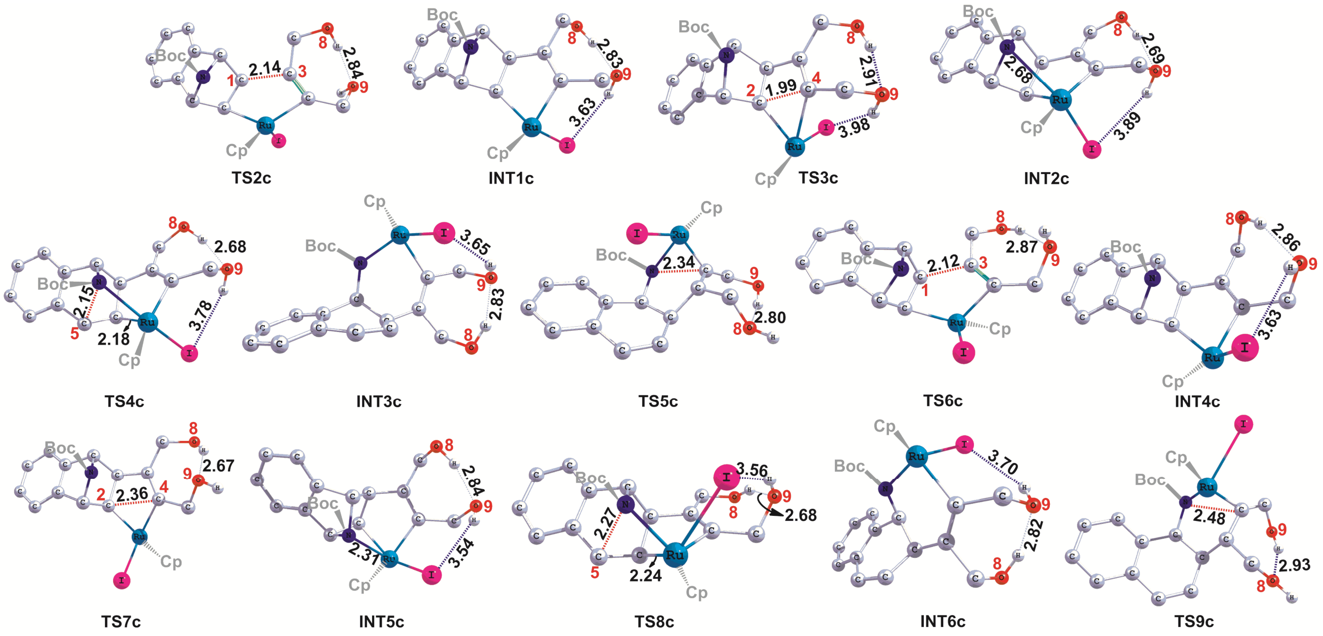

Figure S7. Optimized structures (bond length in Å) of selected stationary points in reaction **c**, obtained at the IDSCRF-B3LYP/BS1 level in dioxane solvent. All hydrogens with the exceptions of those involved in intra-molecular interactions are omitted for clarity.

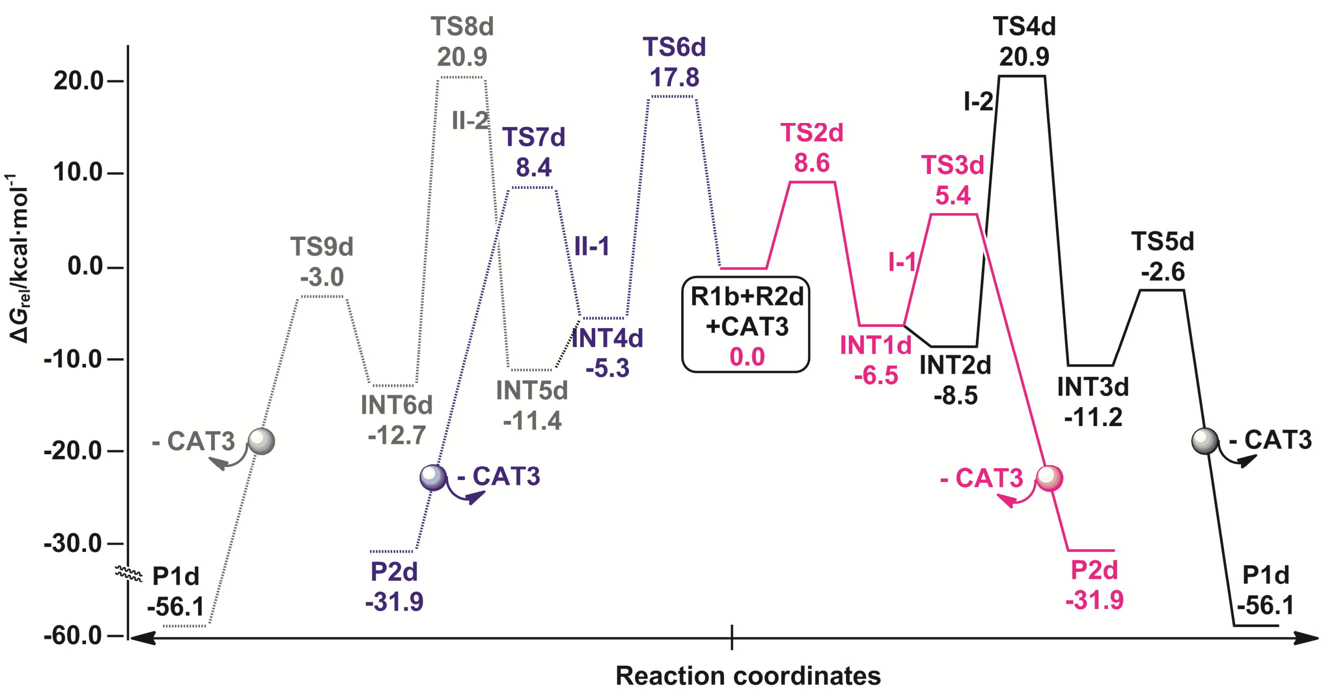

Figure S8. Potential free energy surfaces (PESs) corresponding to Path **I** and **II** in reaction **d**, obtained at IDSCRF-B3LYP/BS1 levels in dioxane solvent at 333 K.

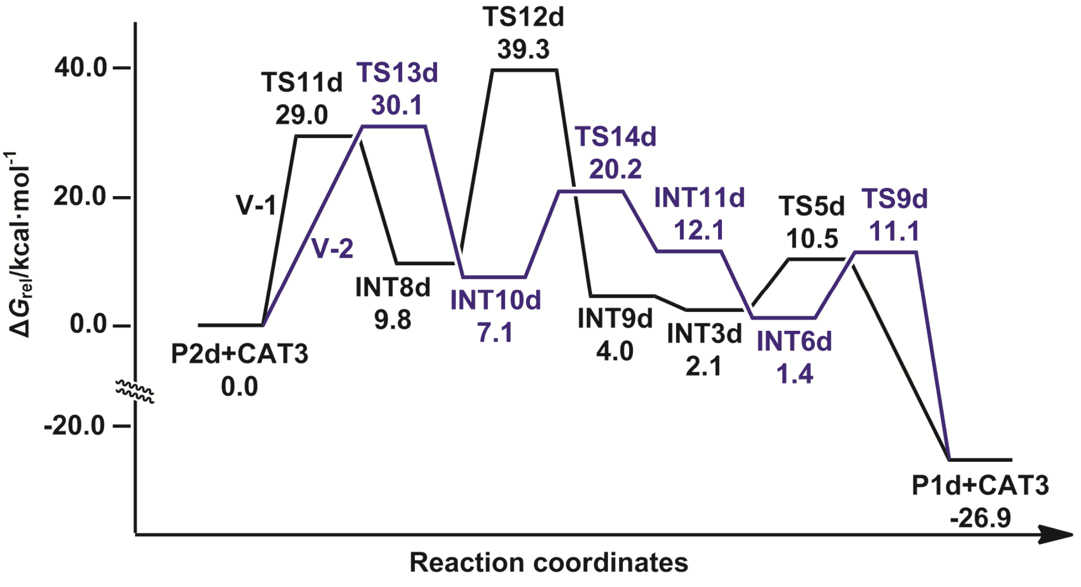

Figure S9. Potential free energy surfaces (PESs) corresponding to Path **V** in reaction **d**, obtained at IDSCRF-B3LYP+D3/BS1 level in dioxane solvent at 333 K.

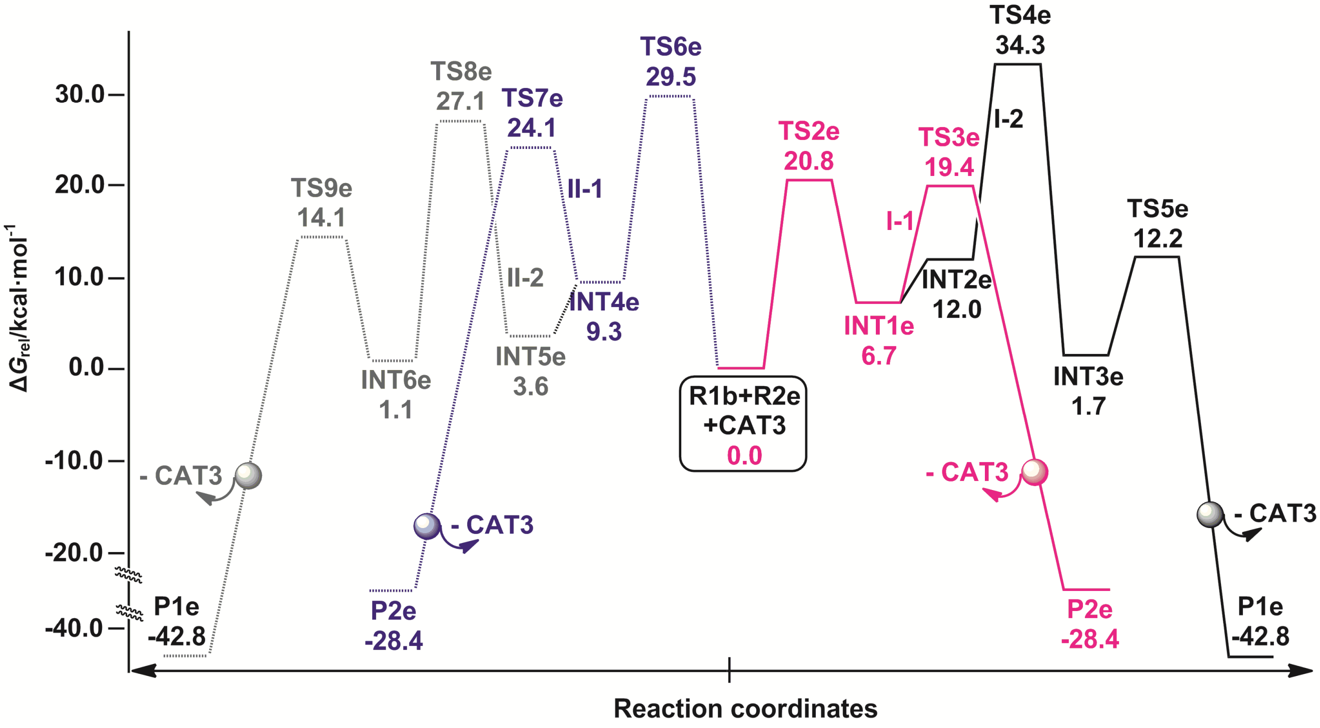

Figure S10. Potential free energy surfaces (PESs) corresponding to Path **I** and **II** in reaction **e**, obtained at the IDSCRF-B3LYP/BS1 level in dioxane solvent at 333 K.

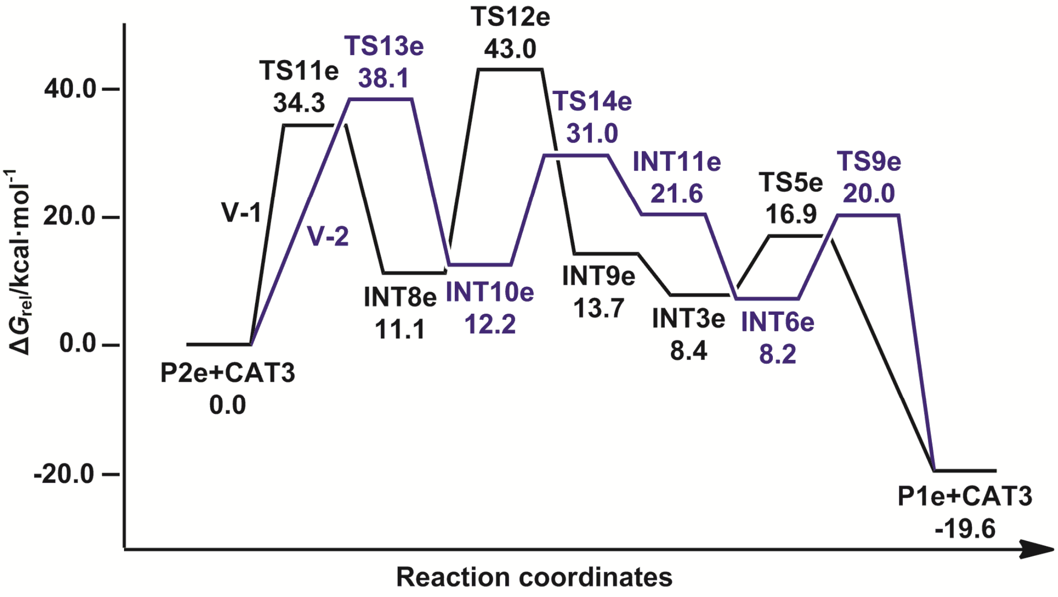

Figure S11. Potential free energy surfaces (PESs) corresponding to Path **V** in reaction **e**, obtained at IDSCRF-B3LYP+D3/BS1 level in dioxane solvent at 333 K.

Table S1. Relative free energies of stationary points on Path **V-I** of reaction **a-e**, obtained at both IDSCRF-B3LYP+D3/BS1 and IDSCRF-B3LYP/BS1 levels in dioxane solvent, at experimental temperatures.

	**a (363 K)**		**b (333 K)**		**c (323 K)**		**d (333 K)**		**e (333 K)**	
	B3LYP+D3	B3LYP	B3LYP+D3	B3LYP	B3LYP+D3	B3LYP	B3LYP+D3	B3LYP	B3LYP+D3	B3LYP
**P2+CAT3**	0.0	0.0	0.0	0.0	0.0	0.0	0.0	0.0	0.0	0.0
**TS11**	31.7	43.6	31.1	47.7	25.7	41.0	29.0	43.7	34.3	50.8
**INT8**	11.3	27.0	9.8	28.9	3.7	23.5	9.8	25.9	11.1	31.3
**TS12**	**40.3**	**58.1**	**41.2**	**60.1**	**37.6**	**55.0**	**39.3**	**56.4**	**43.0**	**63.8**
**INT9**	15.4	33.7	15.3	34.6	5.6	24.6	4.0	23.6	13.7	35.6
**INT3**	8.7	27.4	6.6	27.1	-1.0	18.6	2.1	20.7	8.4	30.1
**TS5**	11.3	30.6	23.9	44.5	5.0	26.4	10.5	29.3	16.9	40.6
**P1+CAT3**	-23.6	-20.7	-17.7	-13.5	-27.9	-25.0	-26.9	-24.2	-19.6	-14.5

Table S2. Single-point free energies obtained at the IDSCRF-M062X//B3LYP/BS1, IDSCRF-X3LYP//B3LYP/BS1, CAM-B3LYP//B3LYP/BS1, MP2(Full)//B3LYP/BS1 and B2PLYP(Full)//B3LYP/BS1 computational levels for selected paths in reaction **a** under 363 K, denoted as M062X, X3LYP, CAM-B3LYP, MP2 and B2PLYP respectively. Single-point free energies obtained at IDSCRF-B3LYP//BS3/BS1 level (363 K, denated as B3LYP//BS3/BS1[a]) are also listed for further comparison.

	**B3LYP/BS1**		**M062X**		**X3LYP**		**CAM-B3LYP**	
	**ΔG**	**Δ(ΔG)**	**ΔG**	**Δ(ΔG)**	**ΔG**	**Δ(ΔG)**	**ΔG**	**Δ(ΔG)**
**Path I-1**								
**R1a+R2a+CAT3**	0.0		0.0		0.0		0.0	
**TS2a**	18.4	**18.4**	17.3	**17.3**	18.4	18.4	19.7	**19.7**
**INT1a**	0.4		-0.4		-0.1		1.5	
**TS3a**	16.2	15.8	15.0	15.4	16.4	16.5	17.5	16.0
**CAT3+P2a**	-28.7		-29.5		-28.7		-28.3	
**Path V-2**								
**CAT3+P2a**	-28.7		-29.5		-28.7		-28.3	
**TS13a**	**20.4**	**49.2**	**19.0**	48.5	19.4	**48.2**	21.3	49.6
**INT10a**	-2.3		-3.7		**-3.5**		-1.6	
**TS14a**	13.4	15.7	12.2	15.9	13.2	16.5	14.0	15.6
**INT11a**	4.7		4.2		4.3		5.7	
**INT6a**	-0.9		-1.8		-0.9		0.2	
**TS9a**	**7.6**	8.5	7.7	9.5	8.2	9.1	**9.3**	9.1
**CAT3+P1a**	-49.5		-50.4		-49.8		-49.5	
**Differences of ΔG[b]**	**0.0**		**-1.4**		**-1.2**		**+1.7**	
**Differences of Δ(ΔG)[b]**		**0.0**		**-1.1**		**-1.0**		**+1.7**

[a]: When the basis set for Ru atom is changed to SDD and the other atoms keep the same as BS1, it's denoted as BS3.

[b]: Relative to the IDSCRF-B3LYP/BS1 results (First coulumn).

Table S2. (Continued)

	**B3LYP/BS1**	**MP2**	**B2PLYP**	**B3LYP/BS3[a]//BS1**

	ΔG	Δ(ΔG)	ΔG	Δ(ΔG)	ΔG	Δ(ΔG)	ΔG	Δ(ΔG)
**Path I-1**								
**R1a+R2a+CAT3**	0.0		0.0		0.0		0.0	
**TS2a**	18.4	**18.4**	18.8	18.8	16.3	**16.3**	19.5	**19.5**
**INT1a**	0.4		0.7		-1.3		1.0	
**TS3a**	16.2	15.8	16.7	16.0	14.1	15.4	16.7	15.7
**CAT3+P2a**	-28.7		-28.9		-29.0		-29.2	
**Path V-2**								
**CAT3+P2a**	-28.7		-28.9		-29.0		-29.2	
**TS13a**	20.4	49.2	20.8	49.7	18.5	47.5	20.8	50.0
**INT10a**	**-2.3**		-2.4		**-5.0**		-2.4	
**TS14a**	13.4	**15.7**	13.5	15.9	11.2	16.2	14.4	**16.8**
**INT11a**	**4.7**		5.0		2.9		**6.0**	
**INT6a**	**-0.9**		**0.3**		-2.7		-0.4	
**TS9a**	7.6	**8.5**	7.8	**7.5**	6.2	8.9	8.8	9.2
**CAT3+P1a**	-49.5		-50.2		-50.8		-49.7	
**Differences of ΔG[b]**	**0.0**		**+1.2**		**-2.7**		**+1.3**	
**Differences of Δ(ΔG)[b]**		**0.0**		**-1.0**		**-2.1**		**+1.1**

[a]: When the basis set for Ru atom is changed to SDD and the other atoms keep the same as BS1, it's denoted as BS3.

[b]: Relative to the IDSCRF-B3LYP/BS1 results (First coulumn).

Table S3.The optimized cartesian coordinates (Å) for CpRuI (**CAT3**)'s formation process, located at IDSCRF-B3LYP/BS1 level in dioxane solvent.

Species	Cartesian coordinates				Species	Cartesian coordinates			
**CAT1**	44	0.026852	-1.403034	-0.360273	**CAT4**	44	-0.009311	-0.520414	-1.207903
	15	1.860393	0.096248	-0.010530		15	-1.879069	0.291387	0.089645
	15	-1.861979	0.018663	0.026651		15	1.901934	0.303367	0.009108
	17	0.057383	-0.825119	-2.817286		6	0.268820	0.667067	-3.120893
	6	-0.286169	-2.879960	1.333414		1	0.539125	1.712602	-3.166448
	1	-0.599139	-2.618075	2.334419		6	1.187083	-0.439458	-3.159644
	6	-1.156570	-3.269982	0.254454		6	-1.052328	0.121864	-3.123290
	6	1.056330	-3.013610	0.864207		1	2.264879	-0.364659	-3.196504
	1	-2.236557	-3.302651	0.289325		6	0.434498	-1.639410	-3.155655
	6	-0.352196	-3.605398	-0.860785		1	-1.972844	0.690114	-3.125013
	1	1.950284	-2.822475	1.443064		6	-0.960588	-1.299376	-3.131195
	6	1.026134	-3.451216	-0.493040		1	0.834601	-2.643997	-3.159033
	1	-0.711520	-3.897033	-1.838396		1	-1.787249	-1.993861	-3.142418
	1	1.881877	-3.645158	-1.122553		6	-2.206303	2.127367	0.105608
	6	2.286181	0.564970	1.738605		6	-3.040894	2.729955	1.064388
	6	3.277951	1.521005	2.030191		6	-1.682511	2.932464	-0.915702
	6	1.637340	-0.066322	2.807256		6	-3.330507	4.093693	1.003796
	6	3.610779	1.824300	3.349849		1	-3.469561	2.133004	1.861642
	1	3.790737	2.033346	1.222559		6	-1.978030	4.296532	-0.982530
	6	1.970757	0.235728	4.131005		1	-1.026272	2.490889	-1.655095
	1	0.852614	-0.783099	2.598420		6	-2.801176	4.882095	-0.020605
	6	2.959069	1.179851	4.405519		1	-3.974524	4.538724	1.757361
	1	4.379204	2.564991	3.553758		1	-1.559330	4.898339	-1.784541
	1	1.449945	-0.263859	4.943127		1	-3.029023	5.943411	-0.067022
	1	3.218856	1.417906	5.433373		6	-3.460072	-0.335484	-0.687417
	6	3.420663	-0.761390	-0.578644		6	-3.589921	-1.711072	-0.953119
	6	3.434273	-1.340385	-1.862087		6	-4.520927	0.513639	-1.038922
	6	4.563175	-0.893796	0.225150		6	-4.752980	-2.219728	-1.532767
	6	4.570669	-1.999221	-2.333204		1	-2.775323	-2.385119	-0.705591
	1	2.544178	-1.286520	-2.482968		6	-5.678478	0.002814	-1.633464
	6	5.693787	-1.568273	-0.245349		1	-4.454460	1.579197	-0.854318

Species	Cartesian coordinates				Species	Cartesian coordinates			
	1	4.582007	-0.478684	1.225687		6	-5.801498	-1.364145	-1.878554
	6	5.705536	-2.116355	-1.527128		1	-4.834210	-3.287411	-1.718095
	1	4.562742	-2.429772	-3.331039		1	-6.484362	0.680719	-1.901793
	1	6.565191	-1.662362	0.397253		1	-6.703449	-1.759842	-2.337317
	1	6.586598	-2.636767	-1.892662		6	-2.123321	-0.122079	1.884549
	6	1.975213	1.751714	-0.849164		6	-1.422624	0.610637	2.855901
	6	1.467428	2.893382	-0.207481		6	-2.980863	-1.147663	2.308985
	6	2.528830	1.895987	-2.129797		6	-1.592688	0.340283	4.213667
	6	1.540803	4.146974	-0.813686		1	-0.746737	1.405577	2.559152
	1	1.021156	2.811716	0.778004		6	-3.141489	-1.424327	3.667915
	6	2.593556	3.150205	-2.739721		1	-3.536197	-1.731005	1.584986
	1	2.921732	1.034452	-2.655120		6	-2.452822	-0.679137	4.624931
	6	2.107330	4.280656	-2.082227		1	-1.042816	0.923405	4.946128
	1	1.146629	5.016386	-0.295452		1	-3.811393	-2.222898	3.974476
	1	3.032222	3.240178	-3.729751		1	-2.583762	-0.891405	5.682517
	1	2.166749	5.257125	-2.555207		6	1.981285	0.613045	1.842966
	6	-1.965878	1.819397	-0.420482		6	2.302110	1.848213	2.425852
	6	-2.578797	2.791007	0.386461		6	1.784339	-0.498155	2.681270
	6	-1.515121	2.184438	-1.700090		6	2.419045	1.969240	3.814128
	6	-2.732804	4.100401	-0.075114		1	2.480909	2.719417	1.806619
	1	-2.945819	2.534031	1.374060		6	1.916391	-0.376542	4.063298
	6	-1.685789	3.489642	-2.161297		1	1.525332	-1.460654	2.250557
	1	-1.031769	1.443428	-2.330205		6	2.232673	0.857815	4.636117
	6	-2.292012	4.452018	-1.351704		1	2.670694	2.933813	4.247082
	1	-3.207804	4.841169	0.562923		1	1.764235	-1.248762	4.692684
	1	-1.332882	3.754795	-3.153785		1	2.335965	0.951330	5.713837
	1	-2.420880	5.469036	-1.712526		6	2.456256	1.914430	-0.735508
	6	-2.346792	-0.034636	1.816580		6	3.573876	2.006226	-1.579401
	6	-3.299942	-0.946976	2.297746		6	1.678710	3.068415	-0.535128
	6	-1.658755	0.760124	2.749662		6	3.907001	3.215530	-2.196639
	6	-3.564437	-1.052230	3.665589		1	4.194828	1.135112	-1.755875
	1	-3.848849	-1.574592	1.603874		6	2.020081	4.278695	-1.138575
	6	-1.931676	0.663888	4.114500		1	0.794468	3.022599	0.090893
	1	-0.901617	1.459820	2.411129		6	3.136626	4.356636	-1.975070
	6	-2.885982	-0.244305	4.578698		1	4.775386	3.261423	-2.848258
	1	-4.307465	-1.764604	4.013842		1	1.407542	5.157915	-0.958320
	1	-1.392249	1.296395	4.814107		1	3.400592	5.297204	-2.450377
	1	-3.097199	-0.321649	5.641561		6	3.446279	-0.731925	-0.143874
	6	-3.414054	-0.552233	-0.836065		6	4.630600	-0.330105	0.500597
	6	-4.669801	-0.022464	-0.490388		6	3.458573	-1.916559	-0.889330
	6	-3.337598	-1.467592	-1.894154		6	5.796564	-1.084333	0.383784
	6	-5.821784	-0.423212	-1.165133		1	4.641882	0.575552	1.099381
	1	-4.751044	0.707735	0.309074		6	4.627135	-2.676201	-1.002810
	6	-4.493068	-1.861926	-2.575942		1	2.544433	-2.264934	-1.353274
	1	-2.368867	-1.841369	-2.206167		6	5.798085	-2.261223	-0.371053
	6	-5.736365	-1.348197	-2.209875		1	6.702138	-0.757045	0.887364
	1	-6.784948	-0.008553	-0.879953		1	4.612899	-3.598099	-1.577507
	1	-4.414737	-2.567283	-3.398805		1	6.705483	-2.852825	-0.456847
	1	-6.633860	-1.657236	-2.738892		53	-0.061382	-3.014139	0.182373
**CAT2**	44	-0.218756	-0.295967	0.038246	**CAT3**	44	0.623977	-0.365755	-0.000230
	17	-2.528162	0.094881	-0.080177		6	2.789629	-0.649250	0.388649
	6	1.911880	-0.773750	0.416229		1	3.234677	-1.572280	0.735679
	1	2.267932	-1.728286	0.779908		6	2.592105	-0.273277	-0.972938
	6	1.730173	-0.412457	-0.951804		6	2.251319	0.388294	1.218298
	6	1.488967	0.328615	1.225339		1	2.864537	-0.860243	-1.840238
	1	1.931859	-1.043412	-1.807373		6	1.942087	1.009526	-0.997066
	6	1.209744	0.927780	-0.999820		1	2.234117	0.392063	2.300194
	1	1.484829	0.357483	2.306987		6	1.738329	1.426108	0.364227
	6	1.064817	1.391554	0.353135		1	1.661415	1.564705	-1.882037
	1	0.972419	1.487179	-1.894830		1	1.278323	2.349978	0.687676
	1	0.700199	2.362478	0.660462		53	-2.011488	0.053030	0.000034
**MeI**	53	0.000000	0.000000	-0.045460	**MeCl**	6	0.008495	0.014715	0.006007
	6	0.000000	0.000000	2.135738		1	-0.002458	-0.004257	1.094709
	1	1.037476	0.000000	2.459909		1	1.031283	-0.004257	-0.367221
	1	-0.518738	0.898481	2.459909		1	-0.519329	0.890988	-0.367221
	1	-0.518738	-0.898481	2.459909		17	-0.843260	-1.460571	-0.596275
**COM1_a**	44	0.325271	-0.537617	-0.541885	**TS1_a**	44	0.765180	-0.103302	-0.111458
	17	0.471555	1.266605	-2.125574		17	0.109067	1.205917	1.758956
	6	0.370187	-2.450942	0.587909		6	1.344356	0.327420	-2.173236
	1	0.031101	-2.583599	1.606542		1	0.645489	0.678004	-2.920934

Species	Cartesian coordinates				Species	Cartesian coordinates			
	6	-0.429156	-2.581196	-0.590903		6	2.126036	1.160298	-1.294079
	6	1.688784	-2.076343	0.179873		6	1.672426	-1.038065	-1.905339
	1	-1.481047	-2.832807	-0.620792		1	2.103989	2.241041	-1.261084
	6	0.415269	-2.351681	-1.738017		6	2.897837	0.306402	-0.455877
	1	2.525027	-1.877879	0.837128		1	1.250420	-1.902661	-2.398327
	6	1.723180	-2.039915	-1.262032		6	2.598295	-1.057431	-0.817057
	1	0.102476	-2.385247	-2.772839		1	3.571793	0.619060	0.330071
	1	2.583205	-1.793916	-1.870034		1	3.018964	-1.940838	-0.355645
	53	-0.770272	1.074295	1.412644		53	-1.364220	-1.641059	0.392630
	6	-0.954352	3.098060	0.592918		6	-2.044238	0.088491	2.853280
	1	-0.566304	3.019097	-0.421631		1	-2.076467	1.156785	2.996880
	1	-2.010083	3.355774	0.620660		1	-2.924116	-0.381482	2.443495
	1	-0.354938	3.750310	1.223018		1	-1.403620	-0.498560	3.495880
**COM2_a**	44	-0.120004	-0.643097	-0.203961	**COM1_b**	44	-3.034498	1.984853	8.095301
	53	1.021824	1.604950	0.824670		17	-2.536659	3.197719	10.206290
	6	1.563977	-1.947208	-0.575037		6	-4.093855	1.776608	6.159888
	1	2.562284	-1.577580	-0.765331		1	-4.731472	0.949694	5.875993
	6	1.005405	-2.249390	0.705701		6	-4.516092	2.981291	6.838307
	6	0.551299	-2.203038	-1.568964		6	-2.696504	1.877034	5.913837
	1	1.504598	-2.149779	1.659817		1	-5.523189	3.218880	7.151946
	6	-0.351665	-2.691523	0.501420		6	-3.369495	3.821386	6.980015
	1	0.662574	-2.060234	-2.635806		1	-2.085864	1.131172	5.421784
	6	-0.616498	-2.692883	-0.904129		6	-2.234267	3.145308	6.424150
	1	-1.044878	-2.983931	1.279161		1	-3.349622	4.780478	7.479847
	1	-1.547066	-2.977431	-1.377116		1	-1.226837	3.533244	6.361336
	17	-2.328940	0.454430	-0.908624		53	-4.738089	0.178135	9.497618
	6	-2.559262	2.239521	-0.587908		6	-4.699906	0.772825	11.602553
	1	-3.427705	2.334102	0.060945		1	-4.041665	1.641420	11.624150
	1	-1.649593	2.595839	-0.106945		1	-5.722464	1.011453	11.884141
	1	-2.723600	2.714895	-1.552876		1	-4.306625	-0.069335	12.166246
**Dioxane**	8	-0.107824	-0.188248	0.027060		8	-1.279845	0.560678	8.768376
	6	0.073953	0.034930	1.423632		6	-1.073428	-0.663028	8.042200
	1	1.109569	0.361480	1.560368		1	-2.062653	-1.078104	7.839109
	1	-0.077354	-0.904156	1.980385		1	-0.568867	-0.441032	7.090882
	6	-1.451250	-0.578665	-0.248700		6	-0.233552	-1.623831	8.875024
	1	-1.529737	-0.700324	-1.333538		1	-0.793209	-1.907995	9.780708
	1	-1.671385	-1.545439	0.232589		1	-0.013419	-2.529485	8.301053
	6	-0.904149	1.089445	1.928768		8	1.012882	-1.037740	9.226191
	1	-0.825663	1.211104	3.013605		6	0.804341	0.176045	9.945030
	1	-0.684014	2.056221	1.447481		1	1.792888	0.592909	10.160243
	6	-2.429350	0.475851	0.256436		1	0.293213	-0.029364	10.898783
	1	-2.278041	1.414937	-0.300318		6	-0.016414	1.162517	9.129445
	1	-3.464967	0.149303	0.119698		1	-0.265621	2.057323	9.700947
	8	-2.247575	0.699029	1.653007		1	0.518388	1.439644	8.209707
**TS1_b**	44	0.106344	0.166376	0.043325	**COM2_b**	44	-1.055858	3.164685	5.878528
	17	-0.396883	-0.098812	2.354395		17	0.074339	5.214658	7.244031
	6	1.544240	-0.078222	-1.583740		6	-1.750936	2.081452	4.131679
	1	2.284821	0.669077	-1.834513		1	-1.943060	1.017962	4.095547
	6	1.716996	-1.154717	-0.636156		6	-2.704131	3.104842	4.466550
	6	0.230139	-0.180034	-2.129733		6	-0.518142	2.740468	3.780590
	1	2.607977	-1.343186	-0.052184		1	-3.722831	2.937954	4.788377
	6	0.512747	-1.916481	-0.607132		6	-2.059022	4.372805	4.362442
	1	-0.211713	0.485874	-2.857958		1	0.395384	2.249370	3.470847
	6	-0.421042	-1.293474	-1.498055		6	-0.701306	4.144250	3.923846
	1	0.296156	-2.777917	0.007902		1	-2.512222	5.334863	4.559090
	1	-1.435631	-1.630067	-1.657711		1	0.041457	4.907365	3.731321
	53	-0.682925	2.707358	0.305386		53	-2.713175	2.444660	8.028805
	6	-1.362005	2.209732	3.274335		6	-0.347478	5.386823	9.007154
	1	-0.835106	1.693851	4.061108		1	-0.651605	6.420137	9.163794
	1	-1.099255	3.240501	3.096960		1	-1.159654	4.688193	9.206185
	1	-2.364775	1.889488	3.029996		1	0.543721	5.142902	9.582322
	8	-2.209756	-3.588912	-0.022782		8	0.675221	1.958528	6.900552
	6	-2.887932	-4.713663	-0.580778		6	0.489354	0.542943	7.131452
	1	-2.364884	-4.974358	-1.506224		1	-0.526623	0.429156	7.511262
	1	-2.830838	-5.568348	0.112053		1	0.592488	0.012336	6.174517
	6	-4.349375	-4.372741	-0.852024		6	1.521143	0.046001	8.132180
	1	-4.406028	-3.585887	-1.622600		1	1.349699	0.526640	9.108012
	1	-4.896856	-5.251198	-1.208248		1	1.434955	-1.037718	8.255510
	8	-4.992894	-3.939055	0.341406		8	2.845915	0.309799	7.677247
	6	-4.314369	-2.816810	0.902061		6	3.023278	1.702537	7.457790

Species	Cartesian coordinates				Species	Cartesian coordinates			
	1	-4.838502	-2.559819	1.827786		1	4.042338	1.843337	7.084178
	1	-4.371438	-1.957009	0.214592		1	2.915156	2.257426	8.403636
	6	-2.854213	-3.152947	1.178445		6	2.015304	2.227600	6.443473
	1	-2.302800	-2.277785	1.532727		1	2.095969	3.310060	6.331303
	1	-2.795674	-3.947395	1.939303		1	2.168022	1.744868	5.467979
**TS1_c**	44	0.103016	-1.036839	0.537168	**TS1_d**	44	0.180710	0.023957	-1.045566
	15	1.669616	0.688676	0.079927		15	0.914188	1.863250	0.261404
	15	-1.982106	0.202617	0.008832		15	1.440135	-1.775627	0.099807
	6	-0.847652	-1.247163	2.645431		6	1.387212	-0.645769	-2.917343
	1	-1.477813	-0.373380	2.774835		1	2.325797	-1.036582	-2.538095
	6	-1.314529	-2.485606	2.044244		6	0.195755	-1.459488	-3.092108
	6	0.040305	-1.657406	3.723976		6	1.394218	0.268925	-4.049656
	1	-2.132532	-2.603332	1.350609		1	-0.050374	-2.371354	-2.570607
	6	-0.682063	-3.545762	2.696138		6	-0.479466	-0.996714	-4.223193
	1	0.560699	-0.982240	4.393790		1	2.154491	1.017514	-4.241823
	6	0.137997	-3.034717	3.744141		6	0.270536	0.055636	-4.823770
	1	-0.792708	-4.594558	2.440928		1	-1.430442	-1.374362	-4.584279
	1	0.774787	-3.623957	4.393344		1	-0.028363	0.628771	-5.693456
	17	2.008379	-2.404938	1.213652		17	-1.075388	1.646562	-2.378409
	6	2.928115	-3.739211	-0.848610		6	-3.501071	1.947740	-1.375886
	1	3.042512	-4.339357	0.040453		1	-3.497829	2.120477	-2.440804
	1	3.627646	-2.931698	-1.011380		1	-3.128637	2.740452	-0.742072
	53	0.542644	-2.265942	-1.896897		53	-2.222962	-0.167782	0.306442
	1	2.529329	-4.242384	-1.715271		6	2.241334	2.926738	-0.501733
	6	2.360464	1.572770	1.567274		6	2.651676	4.120073	0.122829
	6	3.392024	2.520187	1.422869		6	2.819239	2.581214	-1.726853
	6	1.901594	1.275311	2.853807		6	3.631276	4.924582	-0.455789
	6	3.924330	3.166207	2.536829		1	2.200272	4.424935	1.061748
	1	3.785267	2.749773	0.437500		6	3.799805	3.389970	-2.310945
	6	2.438174	1.919876	3.973313		1	2.479962	1.684185	-2.228582
	1	1.135416	0.520084	2.972764		6	4.210912	4.560023	-1.675340
	6	3.446436	2.869148	3.817303		1	3.937584	5.840710	0.041730
	1	4.718072	3.896682	2.406446		1	4.232503	3.105279	-3.266188
	1	2.070675	1.670235	4.964969		1	4.971009	5.191169	-2.127612
	1	3.866363	3.370008	4.685309		6	-0.334882	3.223818	0.593857
	6	3.303455	0.172810	-0.684705		6	-1.058001	3.338812	1.789485
	6	3.586638	0.275294	-2.053847		6	-0.565223	4.178968	-0.413247
	6	4.299252	-0.351535	0.159650		6	-1.981592	4.371442	1.974602
	6	4.821321	-0.133706	-2.565247		1	-0.901719	2.638159	2.598343
	1	2.858608	0.688747	-2.738371		6	-1.486578	5.209650	-0.228099
	6	5.531538	-0.759737	-0.350978		1	-0.024028	4.119342	-1.348927
	1	4.114229	-0.443051	1.222366		6	-2.201738	5.310163	0.967542
	6	5.798349	-0.654658	-1.718122		1	-2.523057	4.439071	2.914240
	1	5.014656	-0.037285	-3.630101		1	-1.641959	5.935485	-1.021685
	1	6.283850	-1.157600	0.324698		1	-2.917192	6.114929	1.113386
	1	6.759263	-0.969592	-2.115857		6	1.589399	1.522270	1.960316
	6	1.137764	2.087081	-1.023388		6	2.904864	1.839097	2.336486
	6	1.093164	3.423308	-0.593579		6	0.752034	0.921514	2.917289
	6	0.762980	1.803023	-2.348737		6	3.350167	1.610899	3.640436
	6	0.736493	4.445948	-1.475355		1	3.589753	2.273731	1.618797
	1	1.343542	3.678224	0.428838		6	1.189744	0.719354	4.225825
	6	0.433447	2.828049	-3.235003		1	-0.244506	0.597810	2.636747
	1	0.716228	0.774157	-2.688928		6	2.489307	1.069338	4.594522
	6	0.425121	4.155012	-2.803211		1	4.371969	1.866450	3.907747
	1	0.711228	5.472462	-1.119764		1	0.519466	0.263597	4.947818
	1	0.158566	2.582308	-4.255946		1	2.832579	0.906322	5.612389
	1	0.162569	4.953383	-3.491785		6	1.188249	-2.186401	1.909288
	6	-2.338243	0.883120	-1.697939		6	2.262178	-2.362116	2.796246
	6	-2.793005	2.195202	-1.903960		6	-0.111803	-2.375460	2.407795
	6	-2.194973	0.053978	-2.823282		6	2.044205	-2.748917	4.120169
	6	-3.127034	2.646849	-3.182324		1	3.278880	-2.190485	2.468365
	1	-2.882185	2.882424	-1.073067		6	-0.329881	-2.767774	3.728830
	6	-2.533138	0.503182	-4.100166		1	-0.969543	-2.195322	1.770833
	1	-1.796585	-0.947626	-2.714219		6	0.749681	-2.965015	4.590883
	6	-3.009723	1.801714	-4.285094		1	2.895453	-2.877787	4.783126
	1	-3.477128	3.667482	-3.310651		1	-1.347536	-2.913514	4.080737
	1	-2.417946	-0.165822	-4.948551		1	0.582747	-3.273477	5.619315
	1	-3.276869	2.152941	-5.278036		6	3.297363	-1.708967	-0.011360
	6	-2.389384	1.661037	1.090864		6	4.098486	-2.841969	0.230016
	6	-3.689860	2.198297	1.154221		6	3.935329	-0.502371	-0.319751

Species	Cartesian coordinates				Species	Cartesian coordinates			
	6	-1.386710	2.264932	1.857433		6	5.488744	-2.761178	0.162134
	6	-3.967590	3.302778	1.958466		1	3.635460	-3.793432	0.469693
	1	-4.491182	1.750617	0.575724		6	5.328520	-0.418560	-0.389423
	6	-1.662422	3.371210	2.665524		1	3.340973	0.377799	-0.521835
	1	-0.384264	1.860687	1.836582		6	6.109131	-1.547738	-0.148891
	6	-2.953453	3.893365	2.718044		1	6.087494	-3.648114	0.350192
	1	-4.978160	3.700271	1.993721		1	5.794995	0.530543	-0.638109
	1	-0.864742	3.814580	3.254889		1	7.192484	-1.487853	-0.205926
	1	-3.172898	4.751059	3.347884		6	1.111873	-3.449036	-0.677010
	6	-3.502431	-0.857927	0.279022		6	-0.005188	-4.213343	-0.303056
	6	-3.952278	-1.741886	-0.715130		6	1.917757	-3.928817	-1.724603
	6	-4.163067	-0.870345	1.520018		6	-0.294389	-5.422292	-0.937559
	6	-5.035178	-2.591614	-0.485211		1	-0.658623	-3.878027	0.492112
	1	-3.467342	-1.773880	-1.682348		6	1.629406	-5.139305	-2.357090
	6	-5.247739	-1.718793	1.748466		1	2.779172	-3.361421	-2.055881
	1	-3.837689	-0.216045	2.319787		6	0.523177	-5.893042	-1.965676
	6	-5.690382	-2.582255	0.746793		1	-1.161699	-5.995741	-0.622265
	1	-5.364942	-3.260529	-1.275271		1	2.271748	-5.486807	-3.161500
	1	-5.741091	-1.704534	2.716371		1	0.298557	-6.835013	-2.458041
	1	-6.534048	-3.243095	0.924783		1	-4.273920	1.295094	-0.993876
**PPh_3**	15	-3.825769	0.222096	-1.275286		8	-6.196968	0.193536	-0.584141
	6	-4.071228	1.649104	-0.117649		6	-7.268375	0.547051	0.288915
	6	-4.984632	1.661870	0.947532		1	-7.088497	1.577503	0.610387
	6	-3.303063	2.801226	-0.357022		1	-8.224685	0.506605	-0.256981
	6	-5.118231	2.792766	1.755967		6	-7.320834	-0.400961	1.481903
	1	-5.596473	0.787282	1.144626		1	-8.188120	-0.185421	2.114283
	6	-3.428120	3.926619	0.457209		1	-6.405241	-0.285161	2.084344
	1	-2.605051	2.816888	-1.190669		8	-7.453434	-1.749088	1.041941
	6	-4.338980	3.925542	1.515744		6	-6.374463	-2.103423	0.178591
	1	-5.832063	2.786882	2.575294		1	-6.552278	-3.134081	-0.144271
	1	-2.822860	4.806861	0.258947		1	-5.419822	-2.062362	0.726431
	1	-4.445127	4.804535	2.145301		6	-6.314439	-1.162466	-1.018905
	6	-5.323211	-0.822676	-0.954292		1	-5.437604	-1.370639	-1.638227
	6	-5.381917	-1.862140	-0.013469		1	-7.223862	-1.279027	-1.629852
	6	-6.459665	-0.562566	-1.738943					
	6	-6.548560	-2.613534	0.143905					
	1	-4.512821	-2.088534	0.596055					
	6	-7.628505	-1.305292	-1.573947					
	1	-6.425766	0.225348	-2.487598					
	6	-7.674790	-2.335442	-0.632207					
	1	-6.576394	-3.416660	0.875411					
	1	-8.498398	-1.087033	-2.187239					
	1	-8.580999	-2.921786	-0.508830					
	6	-2.473969	-0.741860	-0.450474					
	6	-2.063543	-0.559742	0.878994					
	6	-1.803304	-1.695252	-1.235394					
	6	-1.017694	-1.317588	1.410307					
	1	-2.559484	0.178725	1.500939					
	6	-0.766501	-2.460230	-0.701606					
	1	-2.093864	-1.835381	-2.273845					
	6	-0.369398	-2.271144	0.623832					
	1	-0.710715	-1.162319	2.441106					
	1	-0.262340	-3.195361	-1.322800					
	1	0.444513	-2.858979	1.038857					

Table S4. The optimized cartesian coordinates (Å) for reaction **a**, located at IDSCRF-B3LYP/BS1 level in dioxane solvent.

Species	Cartesian coordinates				Species	Cartesian coordinates			
**R1a**	6	3.079572	1.588476	-1.424321	**R2a**	6	-0.605482	0.001057	-0.000562
	6	3.059073	0.344454	-0.764484		6	0.605485	-0.001033	-0.000762
	6	2.434734	0.194655	0.458404		6	-2.070703	-0.028539	-0.001420
	6	1.812760	1.324475	1.025763		1	-2.445844	0.486676	0.892861
	6	1.833201	2.554333	0.373487		1	-2.446029	0.546587	-0.858318
	6	2.476371	2.701396	-0.871500		6	-2.649373	-1.454761	-0.050152
	1	2.410003	-0.763548	0.970180		1	-2.321151	-2.038045	0.814830
	1	1.305941	1.231457	1.982061		1	-3.743331	-1.423358	-0.048962
	1	1.342792	3.411409	0.826238		1	-2.321338	-1.977563	-0.953044
	1	2.485261	3.663329	-1.377077		6	2.070718	0.028581	0.000088

Species	Cartesian coordinates				Species	Cartesian coordinates			
	6	3.869120	1.343172	-2.727191		1	2.445861	-0.486780	-0.894108
	6	5.317287	1.044818	-2.298977		1	2.446050	-0.546397	0.857085
	6	5.297328	-0.134556	-1.672931		6	2.649365	1.454821	0.048581
	6	3.836612	-0.617488	-1.688021		1	2.321320	1.977767	0.951388
	1	3.713875	2.036727	-3.549873		1	3.743324	1.423434	0.047395
	1	6.146011	1.728290	-2.433422		1	2.321130	2.037953	-0.816499
	1	6.105953	-0.649919	-1.170685					
	1	3.642907	-1.682382	-1.583093					
	7	3.445205	-0.060793	-3.014187					
	6	2.235446	-0.403003	-3.571836					
	8	1.652746	-1.455412	-3.373606					
	8	1.813141	0.553731	-4.435761					
	6	0.609400	0.231352	-5.148380					
	1	-0.216521	0.056657	-4.454904					
	1	0.402019	1.097020	-5.777403					
	1	0.750663	-0.660752	-5.763580					
**P1a**	6	4.138979	-0.709634	-0.220626	**P2a**	6	1.593398	-0.512874	-0.694902
	6	3.217757	-1.483596	-0.922425		6	0.173748	-0.219404	-1.180412
	6	1.847441	-1.412896	-0.628358		6	-0.701063	-1.386576	-0.628958
	6	2.333939	0.234564	1.075942		6	0.165012	0.196646	1.022154
	6	3.698199	0.138768	0.796792		6	1.587896	-0.251079	0.688966
	1	5.197710	-0.781352	-0.453539		7	-0.216659	0.861355	-0.247768
	1	3.558019	-2.174887	-1.689460		1	0.044497	0.054940	-2.225649
	1	1.988009	0.909912	1.852206		1	0.025435	0.822777	1.900810
	1	4.410892	0.727652	1.366867		6	2.719510	-0.464149	1.460231
	6	1.396438	-0.507638	0.355443		1	2.720553	-0.268764	2.529383
	6	0.897896	-2.337476	-1.250140		6	3.881134	-0.926819	0.820084
	6	-0.858465	-1.732055	0.431056		1	4.787614	-1.079184	1.399126
	6	-0.322300	-2.535762	-0.735192		6	3.886758	-1.185966	-0.550952
	1	1.244096	-2.925987	-2.096837		1	4.797795	-1.537296	-1.027150
	1	-0.984399	-3.283965	-1.163892		6	2.731130	-0.991021	-1.325362
	6	-0.089202	-0.404427	0.655288		1	2.741468	-1.198105	-2.392137
	6	-2.266298	-1.254405	0.101821		6	-0.708070	-1.093167	0.924751
	6	-2.220729	0.022903	-0.320013		6	-2.215091	-0.929369	0.768405
	1	-0.831118	-2.343303	1.344377		6	-2.209220	-1.179679	-0.556214
	1	-0.218873	-0.089558	1.698619		1	-0.344068	-1.884281	1.587069
	7	-0.893301	0.545784	-0.194250		1	-0.332697	-2.364391	-0.952883
	6	-3.451041	-2.170408	0.204481		6	-3.266162	-0.572371	1.771110
	1	-4.294331	-1.769837	-0.367371		1	-3.045346	0.422714	2.184233
	1	-3.204171	-3.135383	-0.260978		1	-4.236195	-0.485715	1.266840
	6	-3.373919	0.872581	-0.779071		6	-3.250355	-1.213816	-1.629585
	1	-3.115093	1.364837	-1.718150		1	-3.025256	-0.434535	-2.372125
	1	-4.215422	0.202888	-0.978473		1	-4.225516	-0.954707	-1.199771
	1	0.410309	4.300328	-0.181001		1	-0.775932	4.716047	-0.253073
	6	1.110580	3.484949	-0.378315		6	0.081061	4.357956	0.323069
	8	0.575708	2.234454	0.076090		8	-0.018932	2.947783	0.564613
	1	2.031864	3.628062	0.186510		1	0.095137	4.822776	1.309154
	1	1.322236	3.451571	-1.449529		1	0.997296	4.595616	-0.222816
	6	-0.527907	1.808535	-0.585625		6	-0.034536	2.179098	-0.557717
	8	-1.106697	2.496018	-1.415034		8	0.046318	2.628152	-1.690449
	6	-3.903913	-2.429015	1.654459		6	-3.370895	-1.579606	2.929941
	1	-3.094678	-2.854322	2.257367		1	-2.421094	-1.665479	3.468095
	1	-4.222969	-1.498979	2.135139		1	-4.134972	-1.269343	3.649600
	1	-4.742667	-3.132430	1.681233		1	-3.635672	-2.575755	2.561402
	6	-3.811028	1.938105	0.242822		6	-3.342598	-2.572221	-2.346907
	1	-4.695574	2.469434	-0.122718		1	-4.099688	-2.545878	-3.136850
	1	-4.062120	1.482578	1.206264		1	-2.387522	-2.842159	-2.809484
	1	-3.024856	2.679493	0.403324		1	-3.610218	-3.369353	-1.645971
**TS2a**	6	2.956292	-1.245369	-0.827507	**INT1a**	6	-2.667672	1.723101	-0.444278
	6	3.029587	-1.092355	0.569805		6	-3.156388	1.218040	0.774333
	6	3.878322	-1.874432	1.333668		6	-4.093110	1.917931	1.518696
	6	4.678360	-2.821642	0.668610		6	-4.559995	3.140857	1.009371
	6	4.604131	-2.975107	-0.715467		6	-4.073229	3.644935	-0.198728
	6	3.728056	-2.187532	-1.484616		6	-3.106491	2.943353	-0.936952
	1	3.941617	-1.760322	2.412733		1	-4.469945	1.534022	2.463284
	1	5.365297	-3.438213	1.241316		1	-5.311620	3.699318	1.560277
	1	5.233999	-3.710372	-1.208196		1	-4.451418	4.591267	-0.575483
	1	3.673653	-2.314998	-2.562377		1	-2.726856	3.345299	-1.872463
	6	1.883588	-0.247267	-1.289231		6	-1.621102	0.715113	-0.922051
	6	0.560663	-0.842576	-0.751503		6	-0.413073	0.895153	0.028457

Species	Cartesian coordinates				Species	Cartesian coordinates			
	6	0.624536	-0.640994	0.690130		6	-0.943938	0.328488	1.370528
	6	2.016068	0.009149	0.917024		6	-2.404071	-0.088939	1.006815
	1	1.924559	0.088681	-2.322378		1	-1.418415	0.685356	-1.989063
	1	0.272232	-1.814900	-1.136411		1	-0.068830	1.926347	0.061178
	1	0.336397	-1.484464	1.306814		1	-0.956810	1.021491	2.221195
	1	2.191576	0.563521	1.831539		1	-2.884632	-0.829610	1.641302
	7	2.090210	0.834501	-0.302195		7	-2.193468	-0.542578	-0.387452
	6	2.915049	1.898810	-0.523760		6	-3.001253	-1.361035	-1.117895
	8	3.167534	2.357458	-1.629226		8	-3.003968	-1.431755	-2.339662
	8	3.354273	2.435766	0.641654		8	-3.752154	-2.159845	-0.312925
	6	4.138316	3.630461	0.497821		6	-4.564203	-3.119769	-1.005162
	1	3.556276	4.420736	0.016951		1	-5.095418	-3.667916	-0.226696
	1	4.411472	3.923111	1.511710		1	-5.273005	-2.620725	-1.670281
	1	5.033493	3.436613	-0.097613		1	-3.944954	-3.800261	-1.595257
	44	-1.154303	0.405645	-0.492936		44	1.244347	-0.469970	-0.276913
	6	-1.513074	2.586583	-1.080595		6	2.301946	-2.482439	-1.045592
	6	-0.404958	2.123079	-1.844971		6	0.871076	-2.516459	-1.219672
	6	-0.867843	1.057407	-2.682650		6	0.510609	-1.567451	-2.230899
	6	-2.251689	0.860116	-2.429839		6	1.677417	-0.858225	-2.570897
	6	-2.658644	1.800497	-1.433498		6	2.790831	-1.437946	-1.839343
	1	-1.489397	3.395376	-0.363177		1	2.879020	-3.114498	-0.384934
	1	0.600512	2.519041	-1.827689		1	0.194669	-3.221915	-0.755578
	1	-0.274050	0.498146	-3.392188		1	-0.490114	-1.409718	-2.610139
	1	-2.882971	0.113397	-2.890798		1	1.752966	-0.043158	-3.277286
	1	-3.661940	1.907041	-1.046126		1	3.817448	-1.104338	-1.899614
	53	-2.606747	-2.025586	-0.271469		53	2.843565	1.835325	-0.354671
	6	-0.738661	0.479328	1.906240		6	0.039146	-0.832680	1.571225
	6	-1.906091	0.733311	1.450186		6	1.413212	-0.549392	1.623335
	6	-0.018506	0.569884	3.217070		6	-0.456926	-2.186159	2.072621
	1	0.838168	1.250826	3.150053		1	-1.270441	-2.534414	1.426112
	1	-0.735517	1.075772	3.877835		1	0.344279	-2.928684	1.988107
	6	-3.244164	1.146169	1.979513		6	-0.945354	-2.127031	3.532292
	1	-3.353367	0.713357	2.983221		1	-0.141146	-1.832469	4.214098
	1	-4.031147	0.697221	1.365541		1	-1.763005	-1.410940	3.659176
	6	0.402136	-0.749772	3.884824		1	-1.309901	-3.109033	3.850633
	1	0.743231	-0.550741	4.905780		6	2.441846	-0.588317	2.689251
	1	-0.440174	-1.445705	3.936286		1	2.461387	0.443831	3.080668
	1	1.220502	-1.249456	3.360172		1	2.078989	-1.206596	3.522731
	6	-3.447599	2.668528	2.072038		6	3.854796	-1.002762	2.265939
	1	-2.675887	3.134552	2.692515		1	3.865903	-2.037623	1.910761
	1	-3.419113	3.144434	1.088179		1	4.223220	-0.358180	1.465589
	1	-4.421716	2.890068	2.519571		1	4.537219	-0.929751	3.117909
**TS3a**	6	-2.370399	-1.727796	0.048808	**INT2a**	6	-1.730037	2.443136	-0.608182
	6	-2.828750	-1.115955	-1.133418		6	-2.283923	2.143926	0.645966
	6	-3.414711	-1.864754	-2.141686		6	-3.013241	3.089645	1.353975
	6	-3.557574	-3.247515	-1.939842		6	-3.183876	4.354072	0.772929
	6	-3.101427	-3.853306	-0.767296		6	-2.623902	4.655234	-0.472749
	6	-2.485006	-3.096339	0.241918		6	-1.881414	3.699672	-1.179683
	1	-3.765419	-1.402248	-3.060276		1	-3.442802	2.864097	2.326088
	1	-4.032935	-3.853501	-2.705878		1	-3.759329	5.110813	1.298070
	1	-3.228432	-4.923551	-0.631876		1	-2.771197	5.642264	-0.901129
	1	-2.127977	-3.573757	1.150407		1	-1.446719	3.938122	-2.146069
	6	-1.741878	-0.599757	0.877350		6	-1.001659	1.193604	-1.055140
	6	-0.439222	-0.291462	0.106814		6	-0.309728	0.760339	1.238967
	6	-0.925075	0.471023	-1.150826		6	-1.845404	0.736268	0.985411
	6	-2.478322	0.364473	-0.998082		1	-0.890541	1.034506	-2.124938
	1	-1.672070	-0.734046	1.953387		1	-0.020735	1.567060	1.919139
	1	0.126114	-1.271174	-0.164825		1	-2.433719	0.173423	1.706084
	1	-0.571006	0.084050	-2.110246		7	-1.805412	0.081453	-0.383065
	1	-3.068520	1.076996	-1.569753		6	-3.009245	-0.354037	-1.013939
	7	-2.581454	0.544738	0.472058		8	-3.173206	-0.341625	-2.212431
	6	-3.725641	0.851990	1.164297		8	-3.878408	-0.806800	-0.106328
	8	-3.934647	0.554395	2.328700		6	-5.112995	-1.330815	-0.644051
	8	-4.566842	1.592732	0.401216		1	-5.637701	-0.556374	-1.206674
	6	-5.756149	2.036217	1.072414		1	-4.908114	-2.180956	-1.297912
	1	-6.319646	2.597061	0.326643		1	-5.694987	-1.640874	0.222322
	1	-5.505446	2.677050	1.921471		44	0.002953	-1.267928	-0.436731
	1	-6.339486	1.184564	1.430558		6	1.187184	-3.061983	-1.122321
	44	1.554704	-0.454096	0.747457		6	-0.035056	-3.094214	-1.851767
	6	2.869551	0.128774	2.440480		6	-0.070808	-1.924552	-2.687439

Species	Cartesian coordinates				Species	Cartesian coordinates			
	6	1.622262	-0.352994	2.978990		6	1.128683	-1.198424	-2.497758
	6	1.485993	-1.712641	2.588936		6	1.897411	-1.866102	-1.495138
	6	2.670872	-2.092016	1.855138		1	1.539918	-3.811706	-0.426666
	6	3.522669	-0.965792	1.790348		1	-0.789282	-3.868807	-1.814930
	1	3.283712	1.117737	2.572825		1	-0.886646	-1.649396	-3.344013
	1	0.919610	0.211212	3.577047		1	1.406510	-0.268388	-2.975513
	1	0.660186	-2.367457	2.835228		1	2.854487	-1.524352	-1.117559
	1	2.864673	-3.061598	1.416408		53	4.189227	1.213278	-0.232598
	1	4.468580	-0.916247	1.268779		6	0.221182	0.878897	-0.191618
	53	2.821215	-0.222757	-1.724611		1	1.207060	1.301265	-0.376339
	6	-0.356485	1.777759	-0.689822		6	0.228138	-0.592588	1.770443
	6	0.446950	1.515836	0.369769		6	-0.294093	-1.816196	1.419575
	6	-0.806125	3.111270	-1.218986		6	1.429871	-0.472370	2.700923
	1	-0.319596	3.922483	-0.669804		1	2.156970	0.201612	2.228931
	1	-1.885665	3.220328	-1.036035		1	1.928520	-1.441953	2.792407
	6	0.908549	2.590681	1.341431		6	-0.375127	-3.103164	2.166012
	1	1.036526	2.176399	2.343123		1	0.412865	-3.136854	2.931675
	1	0.113707	3.342803	1.436340		1	-0.219935	-3.949668	1.491020
	6	-0.531376	3.272254	-2.725821		6	1.043610	0.047331	4.096160
	1	-0.879502	4.248463	-3.077629		1	1.941959	0.156885	4.710909
	1	0.538223	3.190382	-2.940031		1	0.558147	1.027555	4.051382
	1	-1.046065	2.503701	-3.311867		1	0.364624	-0.642260	4.608869
	6	2.207458	3.289434	0.900205		6	-1.749480	-3.249525	2.856987
	1	2.065502	3.818325	-0.046846		1	-1.914415	-2.446123	3.581072
	1	2.522878	4.021405	1.652042		1	-2.562100	-3.222508	2.125217
	1	3.015812	2.570490	0.746628		1	-1.797535	-4.203993	3.388553
**TS4a**	6	-1.710623	2.380308	-0.507592	**INT3a**	6	3.229182	1.286912	0.883457
	6	-2.341709	1.934509	0.670836		6	3.083550	0.667600	-0.379738
	6	-3.312300	2.711099	1.281758		6	4.196257	0.098512	-1.003251
	6	-3.650628	3.944859	0.704996		6	5.448256	0.111203	-0.380355
	6	-3.038921	4.381035	-0.474239		6	5.588699	0.690939	0.881435
	6	-2.070136	3.593891	-1.102209		6	4.486888	1.276085	1.504749
	1	-3.819555	2.363748	2.177210		1	4.080401	-0.366544	-1.977271
	1	-4.414296	4.560706	1.171234		1	6.305654	-0.330293	-0.880312
	1	-3.324554	5.333180	-0.910638		1	6.556665	0.697126	1.375188
	1	-1.584648	3.930804	-2.013630		1	4.598372	1.747542	2.478189
	6	-0.673120	1.468525	-0.979550		6	2.083294	1.973500	1.480984
	6	-0.315230	0.689050	1.328054		6	1.000364	2.272005	0.748172
	6	-1.829982	0.554906	1.019512		6	0.937338	1.920648	-0.727805
	1	-0.427965	1.460932	-2.040877		6	1.692487	0.623350	-1.020259
	1	-0.120466	1.469925	2.072109		1	2.153466	2.276202	2.522959
	1	-2.388780	0.053862	1.814173		1	0.168103	2.815760	1.179118
	7	-1.799832	-0.236746	-0.242567		1	1.545242	2.689371	-1.234360
	6	-2.965501	-0.669173	-0.840416		1	1.805874	0.510386	-2.106656
	8	-3.029989	-1.283151	-1.894309		7	0.936372	-0.563253	-0.544431
	8	-4.063068	-0.319635	-0.118708		6	1.691135	-1.756498	-0.732991
	6	-5.320345	-0.702557	-0.698903		8	2.181070	-2.106620	-1.789891
	1	-5.452187	-0.233751	-1.677008		8	1.796056	-2.450270	0.411253
	1	-5.376811	-1.787523	-0.815457		6	2.586962	-3.651815	0.329546
	1	-6.081857	-0.352560	-0.001896		1	2.594241	-4.062275	1.338532
	44	0.112808	-1.229690	-0.471452		1	2.143068	-4.362043	-0.372747
	6	1.281191	-2.989338	-1.280470		1	3.601414	-3.413158	0.003254
	6	0.014572	-3.000685	-1.931377		44	-0.974546	-0.847661	-0.248244
	6	-0.067808	-1.814000	-2.738948		6	-2.997278	-1.824369	-0.692991
	6	1.141258	-1.095298	-2.603145		6	-2.057545	-2.086354	-1.736565
	6	1.967054	-1.783142	-1.660147		6	-1.054610	-2.970866	-1.203866
	1	1.674213	-3.756468	-0.626544		6	-1.386066	-3.268045	0.140382
	1	-0.742896	-3.769517	-1.865469		6	-2.554064	-2.531623	0.464412
	1	-0.931443	-1.525268	-3.320577		1	-3.895101	-1.229173	-0.760850
	1	1.391534	-0.153990	-3.075021		1	-2.116815	-1.750521	-2.762317
	1	2.946452	-1.456700	-1.332282		1	-0.210334	-3.355568	-1.760980
	53	4.099464	1.427815	-0.216729		1	-0.809807	-3.875603	0.824250
	6	0.286132	0.923203	-0.047282		1	-3.031170	-2.503197	1.434046
	1	1.346651	1.195866	-0.158483		53	-1.836616	0.448356	2.007502
	6	0.323643	-0.646173	1.783106		6	-0.443994	1.976646	-1.369067
	6	-0.108631	-1.881269	1.354234		6	-1.350022	0.982083	-1.295879
	6	1.520920	-0.495899	2.712712		6	-0.669410	3.284367	-2.129193
	1	2.198879	0.251641	2.280800		1	0.149119	3.408412	-2.854333
	1	2.084431	-1.433034	2.748103		1	-1.590040	3.254874	-2.713992
	6	-0.123487	-3.209081	2.029611		6	-0.710252	4.533515	-1.227995

Species	Cartesian coordinates				Species	Cartesian coordinates			
	1	0.674510	-3.243228	2.785186		1	-0.861524	5.435529	-1.831003
	1	0.065228	-4.011112	1.310987		1	-1.528647	4.468937	-0.503623
	6	1.108586	-0.083484	4.136991		1	0.219658	4.666392	-0.666005
	1	2.001500	0.048179	4.755253		6	-2.712000	1.118394	-1.972869
	1	0.560137	0.863767	4.149787		1	-2.587319	1.668116	-2.913749
	1	0.478767	-0.844382	4.609642		1	-3.104271	0.148643	-2.280201
	6	-1.480749	-3.457510	2.724997		6	-3.783960	1.831241	-1.130784
	1	-1.683359	-2.692978	3.480823		1	-3.965109	1.308390	-0.187798
	1	-2.300449	-3.450037	2.000954		1	-3.473826	2.849712	-0.878955
	1	-1.469985	-4.433078	3.219054		1	-4.729503	1.890536	-1.682303
**TS5a**	6	3.303730	0.478546	0.848440	**INT4a**	6	2.524666	0.789292	-1.444259
	6	2.312683	1.147415	0.102472		6	3.196892	-0.386796	-1.064634
	6	2.635039	1.639675	-1.162525		6	4.303795	-0.840345	-1.766399
	6	3.919213	1.481079	-1.690087		6	4.754897	-0.076686	-2.855419
	6	4.904856	0.830197	-0.946884		6	4.088936	1.091845	-3.233325
	6	4.593221	0.332527	0.316618		6	2.952219	1.531888	-2.536297
	1	1.877898	2.155641	-1.742564		1	4.823342	-1.751467	-1.479843
	1	4.148083	1.871658	-2.677932		1	5.636455	-0.394315	-3.405560
	1	5.906358	0.707621	-1.349791		1	4.460575	1.672283	-4.073516
	1	5.346627	-0.194816	0.897201		1	2.437106	2.440141	-2.838272
	6	2.956015	-0.116304	2.134280		6	1.335061	0.914129	-0.493229
	6	1.704775	-0.139551	2.613062		6	0.334723	-0.198752	-0.953546
	6	0.554862	0.580640	1.963417		6	1.056333	-1.502882	-0.452567
	6	0.945761	1.450239	0.721213		6	2.410525	-0.941140	0.115067
	1	3.753266	-0.605366	2.690161		1	0.936000	1.908826	-0.329694
	1	1.509232	-0.635593	3.559160		1	0.295247	-0.175093	-2.046082
	1	0.202302	1.295050	2.726000		1	1.274064	-2.198480	-1.273858
	1	1.023441	2.480359	1.085762		1	2.948420	-1.584666	0.806599
	7	-0.148336	1.396506	-0.242749		7	1.896931	0.304095	0.733356
	6	-0.665258	2.522112	-0.790807		6	2.592703	1.058484	1.643916
	8	-1.651424	2.585098	-1.526737		8	2.477799	2.262119	1.799171
	8	0.029956	3.663954	-0.451741		8	3.394498	0.269975	2.412637
	6	-0.471469	4.870904	-1.032453		6	4.094536	0.965213	3.452400
	1	-1.498186	5.068448	-0.711594		1	4.693405	0.207996	3.959754
	1	0.192563	5.662618	-0.681783		1	4.739062	1.743588	3.035979
	1	-0.453797	4.818638	-2.124333		1	3.393180	1.426586	4.152610
	44	-1.188570	-0.530801	-0.498365		44	-1.759404	-0.195514	-0.425366
	6	-3.278583	-1.324907	-0.991022		6	-4.087306	-0.886522	-1.103411
	6	-3.082491	-0.070730	-1.665199		6	-3.642474	0.231912	-1.831369
	6	-2.065344	-0.256536	-2.652282		6	-2.424527	-0.127043	-2.518850
	6	-1.594065	-1.577944	-2.549387		6	-2.135636	-1.496134	-2.203893
	6	-2.329576	-2.247088	-1.495314		6	-3.136202	-1.934757	-1.292329
	1	-4.007803	-1.525986	-0.217176		1	-4.956126	-0.924859	-0.459518
	1	-3.633877	0.845037	-1.517807		1	-4.115851	1.203930	-1.862884
	1	-1.681256	0.519835	-3.297546		1	-1.880610	0.495597	-3.216838
	1	-0.792669	-2.017504	-3.125297		1	-1.326715	-2.090165	-2.604925
	1	-2.204046	-3.277067	-1.194357		1	-3.184123	-2.917709	-0.843846
	53	1.037333	-2.287862	-0.488915		53	-1.786698	2.266244	0.629394
	6	-0.727784	-0.274011	1.701508		6	0.219551	-2.171900	0.602909
	6	-1.811595	0.352003	1.098208		6	-1.048263	-1.725671	0.750345
	6	-1.018100	-1.408275	2.690488		6	0.837523	-3.261989	1.456302
	1	-0.154631	-2.081570	2.666632		1	1.607850	-2.819529	2.103340
	1	-1.865565	-1.991444	2.318059		1	0.086977	-3.679765	2.132545
	6	-3.002405	1.121914	1.526646		6	1.467879	-4.405863	0.639285
	1	-3.592075	1.442904	0.665985		1	0.726330	-4.880163	-0.012190
	1	-3.621978	0.406259	2.093113		1	2.289794	-4.054642	0.008033
	6	-1.309219	-0.959137	4.134065		1	1.868810	-5.174440	1.307550
	1	-1.465467	-1.836155	4.770465		6	-2.035864	-2.195228	1.806301
	1	-0.487699	-0.380958	4.566790		1	-3.058257	-1.938912	1.506433
	1	-2.214784	-0.345970	4.193191		1	-2.013936	-3.292438	1.881330
	6	-2.669467	2.327547	2.428657		6	-1.775759	-1.575317	3.192024
	1	-2.039747	3.047568	1.899928		1	-0.785555	-1.844901	3.570703
	1	-3.595014	2.833980	2.716360		1	-1.826382	-0.483545	3.143698
	1	-2.151446	2.018871	3.340323		1	-2.522438	-1.922012	3.914625
**TS6a**	6	3.021582	-0.618705	-1.135105	**INT5a**	6	3.136110	0.106562	1.066227
	6	3.126740	-1.051874	0.201301		6	3.175402	0.469489	-0.290686
	6	4.184573	-1.839271	0.621131		6	4.379023	0.758161	-0.918791
	6	5.167934	-2.182601	-0.325230		6	5.556698	0.668268	-0.161281
	6	5.064127	-1.756400	-1.649021		6	5.517189	0.313092	1.189966
	6	3.975638	-0.972191	-2.073937		6	4.297566	0.030714	1.822833

Species	Cartesian coordinates				Species	Cartesian coordinates			
	1	4.270216	-2.175798	1.651303		1	4.416134	1.040982	-1.967272
	1	6.020507	-2.782196	-0.018713		1	6.513383	0.874538	-0.632948
	1	5.837955	-2.027821	-2.361828		1	6.443051	0.244402	1.753964
	1	3.902691	-0.643602	-3.107216		1	4.272411	-0.253011	2.871697
	6	1.705690	0.176166	-1.201668		6	1.676336	-0.140903	1.398619
	6	0.618229	-0.911404	-1.044168		6	1.113959	1.790432	0.032696
	6	0.704366	-1.336529	0.354530		6	1.731198	0.555093	-0.739363
	6	1.883327	-0.487425	0.911589		1	1.481568	-0.865147	2.185093
	1	1.584531	0.907540	-1.995393		1	1.903356	2.546995	0.144201
	1	0.637531	-1.677787	-1.813927		1	1.506316	0.490118	-1.799710
	1	0.745221	-2.401311	0.562437		7	1.108564	-0.582041	0.059818
	1	1.947019	-0.340561	1.984924		6	1.367553	-1.936079	-0.320490
	7	1.679487	0.758393	0.153358		8	1.329871	-2.857666	0.465054
	6	2.339902	1.932360	0.424866		8	1.625709	-2.029198	-1.624053
	8	2.652075	2.768959	-0.402539		6	1.755733	-3.373024	-2.131472
	8	2.557069	2.063188	1.760425		1	2.556955	-3.899548	-1.609054
	6	3.162190	3.304684	2.146448		1	0.813890	-3.909743	-2.002542
	1	2.520536	4.147499	1.877517		1	1.990670	-3.259706	-3.188729
	1	3.280960	3.248423	3.228985		44	-0.995170	-0.055080	0.842695
	1	4.134005	3.431600	1.662544		6	-2.982476	-0.186561	1.839583
	44	-1.421657	-0.654120	-0.446002		6	-2.532372	-1.562341	1.871702
	6	-0.652722	-0.893470	1.813031		6	-1.389874	-1.645375	2.684705
	6	-1.617161	-0.076100	1.586790		6	-1.080087	-0.323947	3.125502
	6	-3.502019	-0.607205	-1.335184		6	-2.099494	0.574228	2.655217
	6	-2.555891	-0.995361	-2.356428		1	-3.875185	0.172457	1.347908
	6	-2.030437	-2.264801	-1.988442		1	-2.992383	-2.380139	1.334918
	6	-2.611200	-2.647463	-0.742246		1	-0.799542	-2.533735	2.864939
	6	-3.542687	-1.636413	-0.361088		1	-0.261284	-0.052326	3.778701
	1	-4.086141	0.301820	-1.334254		1	-2.185427	1.622964	2.900087
	1	-2.333845	-0.439845	-3.256434		53	-1.965248	-1.199898	-1.564292
	1	-1.305326	-2.839791	-2.549117		6	0.776619	1.106184	1.371829
	1	-2.410028	-3.563012	-0.201409		1	0.720901	1.745951	2.250580
	1	-4.146725	-1.640417	0.536381		6	-0.134947	2.395992	-0.545548
	53	-1.302989	2.104875	-1.009122		6	-1.271207	1.753113	-0.222371
	6	-0.191582	-1.802740	2.915111		6	0.001063	3.648124	-1.388820
	1	0.893541	-1.947065	2.899895		1	0.504553	4.428555	-0.797305
	1	-0.422368	-1.292931	3.857819		1	-0.986891	4.047606	-1.635460
	6	-2.623577	0.777051	2.276375		6	-2.654227	2.249824	-0.598937
	1	-3.627765	0.410044	2.024297		1	-3.356240	1.414678	-0.652920
	1	-2.565266	1.779503	1.837155		1	-2.625409	2.667249	-1.614863
	6	-0.884251	-3.176049	2.898338		6	0.794629	3.438631	-2.691829
	1	-0.527972	-3.793116	3.729656		1	0.865488	4.371360	-3.262080
	1	-1.968803	-3.067367	2.990422		1	1.817663	3.100515	-2.491301
	1	-0.677750	-3.716936	1.968760		1	0.312696	2.688157	-3.327052
	6	-2.454929	0.855087	3.801038		6	-3.207643	3.323961	0.355450
	1	-2.577942	-0.124874	4.273668		1	-3.308076	2.943583	1.377192
	1	-1.465684	1.240731	4.065939		1	-2.549544	4.197858	0.395351
	1	-3.204394	1.528174	4.229368		1	-4.198534	3.660887	0.030336
**TS7a**	6	-2.094535	-1.664512	-0.714103	**INT6a**	6	-3.870758	0.707505	0.430610
	6	-2.874961	-0.723756	-1.413698		6	-2.824960	-0.106199	0.919351
	6	-3.685823	-1.114605	-2.468367		6	-3.127469	-1.296705	1.585011
	6	-3.722784	-2.478312	-2.802884		6	-4.454454	-1.690419	1.777135
	6	-2.945592	-3.409751	-2.109422		6	-5.491111	-0.888552	1.295192
	6	-2.104198	-3.008567	-1.059867		6	-5.199046	0.299291	0.625499
	1	-4.289282	-0.395830	-3.016569		1	-2.316883	-1.918110	1.957934
	1	-4.369129	-2.814306	-3.608955		1	-4.674895	-2.615901	2.301012
	1	-2.997375	-4.460063	-2.382127		1	-6.525442	-1.187492	1.441511
	1	-1.495635	-3.733154	-0.526214		1	-6.005316	0.925264	0.250309
	6	-1.302637	-0.855408	0.322969		6	-3.541958	1.945466	-0.275714
	6	-0.275533	-0.133959	-0.577660		6	-2.302679	2.456216	-0.259378
	6	-1.096697	1.003636	-1.229183		6	-1.197188	1.825026	0.569618
	6	-2.552087	0.629787	-0.787219		6	-1.372664	0.290976	0.699486
	1	-0.926829	-1.369586	1.201339		1	-4.340738	2.446990	-0.817923
	1	0.241980	-0.905160	-1.261792		1	-2.092410	3.375605	-0.793110
	1	-1.009813	1.076932	-2.318527		1	-1.385429	2.172571	1.602459
	1	-3.299745	1.414395	-0.874103		1	-0.805156	-0.000379	1.588858
	7	-2.257639	0.232864	0.610113		7	-0.705546	-0.456981	-0.388319
	6	0.598361	1.748995	0.231179		6	-1.490574	-1.178852	-1.331218
	6	-0.514099	2.143041	-0.425540		8	-1.400315	-2.375553	-1.515707
	6	-3.189902	0.107124	1.609510		8	-2.301552	-0.367459	-2.023993

Species	Cartesian coordinates				Species	Cartesian coordinates			
	8	-3.089258	-0.629857	2.574283		6	-3.200942	-1.022783	-2.937875
	8	-4.221200	0.971077	1.412437		44	1.208173	-0.471271	-0.603714
	6	-5.213823	0.966030	2.448795		6	2.396417	-0.107459	-2.483441
	1	-5.963498	1.694652	2.139135		6	3.327851	-0.595951	-1.520628
	1	-4.775326	1.254869	3.407341		6	2.953337	-1.935789	-1.211010
	1	-5.664070	-0.024517	2.550085		6	1.843205	-2.304888	-2.025238
	44	1.792550	0.057163	-0.601081		6	1.477172	-1.172455	-2.791564
	6	2.942537	1.856978	-1.367202		1	2.403172	0.868519	-2.948880
	6	3.775516	1.063521	-0.504081		1	4.166647	-0.062616	-1.099771
	6	3.987154	-0.202299	-1.149402		1	3.437254	-2.572081	-0.482694
	6	3.224985	-0.201946	-2.341232		1	1.314271	-3.247684	-1.993515
	6	2.579945	1.079197	-2.494221		1	0.664150	-1.119815	-3.503745
	1	2.619608	2.869297	-1.171419		53	1.792343	-1.034131	2.026005
	1	4.232166	1.386350	0.420803		6	0.206230	2.331686	0.242922
	1	4.570463	-1.024531	-0.760597		6	1.302248	1.613481	-0.083993
	1	3.122463	-1.038699	-3.021608		6	0.287179	3.865708	0.421186
	1	1.971621	1.394601	-3.330306		1	-0.581024	4.195610	1.003028
	53	2.060161	-1.652444	1.570531		1	1.156360	4.120066	1.036965
	6	-1.252590	3.451928	-0.271238		6	2.664911	2.286329	-0.204498
	1	-0.704666	4.118353	0.401561		1	3.180942	1.962895	-1.112045
	1	-2.220469	3.265364	0.215918		1	2.526645	3.362374	-0.336454
	6	1.213362	2.440189	1.426673		1	-3.771050	-0.221675	-3.406838
	1	1.100098	3.526331	1.291669		1	-2.642416	-1.589040	-3.686655
	1	2.286444	2.248603	1.493082		1	-3.864566	-1.699636	-2.395040
	6	-1.495165	4.170155	-1.610873		6	0.335622	4.691758	-0.878245
	1	-2.080374	3.552608	-2.300875		1	1.213420	4.449999	-1.484019
	1	-2.044792	5.104397	-1.457436		1	-0.542151	4.517699	-1.507538
	1	-0.548619	4.412714	-2.105448		1	0.372849	5.762738	-0.648878
	6	0.550460	2.028464	2.753379		6	3.607939	2.090318	0.994949
	1	0.665586	0.955105	2.925078		1	3.870319	1.040762	1.143301
	1	1.012932	2.560785	3.591549		1	4.531072	2.661271	0.842489
	1	-0.518709	2.259502	2.745408		1	3.140499	2.437663	1.921313
**TS8a**	6	3.155775	0.465738	0.859798	**TS10a**	6	0.007253	0.003579	0.003416
	6	3.028408	0.581604	-0.543503		6	0.003687	0.001523	1.459565
	6	4.159300	0.618201	-1.342488		6	1.313049	-0.009672	-0.532424
	6	5.423357	0.534334	-0.738640		6	2.325008	0.220987	0.576486
	6	5.552778	0.392733	0.646813		7	1.818426	1.422447	1.357858
	6	4.416849	0.340369	1.457152		1	-0.867579	0.363267	2.001729
	1	4.068104	0.683771	-2.422776		1	3.348279	0.384310	0.229944
	1	6.314455	0.554575	-1.359873		6	1.510880	-0.096941	-1.898230
	1	6.539303	0.310927	1.092830		1	2.513681	-0.079800	-2.315611
	1	4.509202	0.233297	2.534672		6	0.389175	-0.175033	-2.742095
	6	1.896875	0.502782	1.586002		1	0.532427	-0.255259	-3.815890
	6	0.964194	1.861357	-0.231536		6	-0.904842	-0.122568	-2.219552
	6	1.560696	0.582025	-0.917242		1	-1.759999	-0.163816	-2.887529
	1	1.878480	0.039370	2.571531		6	-1.107905	-0.017504	-0.838687
	1	1.713143	2.663023	-0.325426		1	-2.113197	0.010368	-0.427114
	1	1.369409	0.535125	-1.992028		44	2.639720	2.379451	3.097600
	7	0.937542	-0.555735	-0.180781		6	2.180887	-0.890830	1.598513
	6	1.328917	-1.851747	-0.405705		1	3.015520	-1.496186	1.931246
	8	1.043594	-2.805326	0.304754		6	0.930440	-0.924959	2.099548
	8	2.112555	-1.966870	-1.515002		1	0.611569	-1.534143	2.940026
	6	2.566598	-3.296634	-1.797351		6	1.357215	2.188899	4.905197
	1	3.173307	-3.214423	-2.699968		6	2.230188	3.321234	5.081227
	1	1.718678	-3.964704	-1.967456		6	2.188224	1.028162	4.785127
	1	3.164449	-3.688177	-0.969899		6	3.570199	2.863380	5.067657
	44	-0.860429	-0.060654	0.900845		6	3.555471	1.435016	4.859369
	6	0.826530	1.390606	1.210106		1	0.275878	2.211820	4.910020
	1	0.485134	2.101270	1.962775		1	1.915450	4.352184	5.177158
	6	-2.750063	-0.522887	2.009751		1	1.841527	0.014912	4.639940
	6	-1.993427	-1.760169	2.072437		1	4.453885	3.481124	5.144953
	6	-0.843684	-1.543079	2.852036		1	4.421806	0.790437	4.813606
	6	-0.835053	-0.168378	3.223821		53	5.040746	2.932627	1.687743
	6	-2.040061	0.456423	2.745965		6	1.669345	2.656621	0.750965
	1	-3.710446	-0.401815	1.529687		8	1.501118	3.637436	1.500973
	1	-2.270043	-2.687007	1.591147		8	1.753030	2.750061	-0.568759
	1	-0.058893	-2.263624	3.034472		6	1.751284	4.091578	-1.107056
	1	-0.083745	0.318496	3.833031		1	2.588803	4.655044	-0.691634
	1	-2.348970	1.475110	2.934636		1	0.811474	4.593852	-0.869646
	53	-2.029895	-1.389350	-1.358837		1	1.860854	3.966299	-2.183418

Species	Cartesian coordinates				Species	Cartesian coordinates			
	6	-0.391902	2.356025	-0.680897					
	6	-1.443843	1.677577	-0.190679					
	6	-0.433479	3.624486	-1.514595					
	1	-1.430387	3.767462	-1.937156					
	1	0.244806	3.514595	-2.373638					
	6	-2.886398	2.153022	-0.303130					
	1	-2.911338	3.210731	-0.000221					
	1	-3.507953	1.628118	0.427751					
	6	-3.574479	2.027256	-1.677023					
	1	-3.020366	2.547797	-2.462963					
	1	-3.665383	0.982375	-1.974596					
	1	-4.578617	2.465168	-1.629657					
	6	-0.042634	4.893980	-0.732911					
	1	0.967749	4.822741	-0.315454					
	1	-0.067694	5.776607	-1.381578					
	1	-0.732572	5.067480	0.099711					
**TS9a**	6	3.911572	-0.093831	0.064208	**TS10ax**	6	-2.596098	-0.948709	1.513210
	6	2.816580	0.773806	-0.126997		6	-3.159067	-0.587456	-0.939368
	6	2.835254	1.660449	-1.205552		7	-2.800827	0.719215	-0.261127
	6	3.903732	1.673712	-2.107262		44	2.205781	0.669051	-0.101021
	6	4.978536	0.799183	-1.930481		6	-3.993154	-1.223266	1.219428
	6	4.981292	-0.074753	-0.845158		6	-1.583090	-1.450136	0.631404
	1	2.004283	2.346616	-1.333406		6	-4.313376	-1.167940	-0.154254
	1	3.899135	2.372041	-2.939733		1	-2.323906	-0.646415	2.523261
	1	5.812354	0.806156	-2.626709		1	-3.394501	-0.444760	-1.996509
	1	5.818350	-0.752019	-0.691769		6	-5.595284	-1.458775	-0.580688
	6	3.919387	-0.988967	1.221055		1	-5.858214	-1.376976	-1.631226
	6	2.857612	-1.140775	2.026670		6	-6.565708	-1.829310	0.370553
	6	1.563736	-0.390917	1.838466		1	-7.575232	-2.059410	0.041901
	6	1.700783	0.853736	0.915309		6	-6.254237	-1.876085	1.728829
	1	4.833556	-1.545782	1.417523		1	-7.016940	-2.150245	2.451431
	1	2.921648	-1.814760	2.877187		6	-4.962490	-1.558521	2.167856
	1	1.292727	-0.002810	2.832515		1	-4.712391	-1.596115	3.224742
	1	1.996097	1.680537	1.570403		6	-1.880717	-1.345838	-0.690690
	7	0.388719	1.174055	0.356799		6	3.201168	-0.641295	1.291138
	6	-0.157908	2.395703	0.590620		6	3.002114	0.497298	1.822525
	8	-1.330691	2.721491	0.461378		1	-1.174346	-1.578963	-1.480765
	8	0.787132	3.327118	0.996058		1	-0.603536	-1.729207	1.004505
	6	0.257965	4.626340	1.271464		6	0.355387	1.882564	-0.471979
	1	-0.482440	4.591351	2.075755		6	1.045938	1.767120	-1.711617
	1	-0.217052	5.054065	0.384611		6	1.222088	2.574657	0.441676
	1	1.113485	5.233721	1.572530		6	2.350859	2.322243	-1.566549
	44	-0.620585	-0.601983	-0.432329		6	2.457264	2.842745	-0.228843
	6	0.332784	-1.281267	1.441162		1	-0.650031	1.523009	-0.267365
	6	-0.935702	-0.682628	1.466930		1	0.674391	1.259798	-2.590029
	6	-1.325838	-1.806203	-2.259365		1	0.972600	2.851559	1.456444
	6	-0.692456	-0.608277	-2.775418		1	3.119831	2.357201	-2.325582
	6	0.671841	-0.663788	-2.438382		1	3.316154	3.356521	0.181506
	6	0.895601	-1.833626	-1.653281		53	2.239262	-1.515932	-1.843553
	6	-0.346836	-2.565405	-1.591060		6	-3.608478	1.754437	-0.628549
	1	-2.365450	-2.069248	-2.382303		8	-4.404259	1.799673	-1.568684
	1	-1.187858	0.175334	-3.330200		8	-3.396805	2.824382	0.199967
	1	1.411041	0.094211	-2.649028		6	-4.196723	3.971891	-0.092334
	1	1.849503	-2.153356	-1.260137		6	3.707679	-2.035768	1.389143
	1	-0.511144	-3.524194	-1.122349		1	4.064558	-2.373381	0.412738
	53	-3.330522	0.316236	-0.680868		1	4.567405	-2.030144	2.072809
	6	0.327465	-2.747781	1.922036		6	3.131196	1.416063	2.984875
	1	-0.697932	-3.128706	1.861971		1	2.126971	1.629730	3.373411
	1	0.565006	-2.703835	2.995971		1	3.528942	2.380985	2.648380
	6	-1.894879	-0.174542	2.465665		1	-5.263777	3.732862	-0.054703
	1	-2.044100	0.886192	2.208087		1	-3.949506	4.709014	0.673639
	1	-2.873915	-0.617916	2.239218		1	-3.968361	4.370610	-1.085459
	6	-1.512892	-0.363049	3.939665		6	2.642729	-3.017848	1.906415
	1	-2.302642	0.044011	4.577750		1	3.069165	-4.022024	1.994846
	1	-1.391317	-1.419863	4.195708		1	2.269709	-2.716700	2.890525
	1	-0.584636	0.158491	4.190847		1	1.799114	-3.066812	1.212543
	6	1.263383	-3.773660	1.264421		6	4.015792	0.844292	4.103731
	1	1.204806	-4.714542	1.821836		1	3.611007	-0.099797	4.480365
	1	0.977960	-3.989768	0.234645		1	5.031632	0.657698	3.743184
	1	2.303273	-3.447189	1.262205		1	4.075548	1.546989	4.940829

Species	Cartesian coordinates	Species	Cartesian coordinates
**TS11a**	6 -1.019933 -2.086761 -1.136402	**INT8a**	6 -0.694650 1.952268 -0.872142
	6 -0.909427 -0.679274 -1.419872		6 -0.151146 0.673269 -1.422634
	6 -2.208228 0.044273 -1.452832		6 1.355888 0.943766 -1.648494
	6 -1.861267 -2.401077 -0.034215		6 1.054445 1.570676 0.828400
	6 -2.215721 -1.149706 0.757633		6 -0.084387 2.420084 0.309706
	7 -0.958847 -0.429146 1.012095		7 0.540883 0.234910 1.226406
	1 -0.013708 -0.284855 -1.894487		1 -0.663469 0.365096 -2.336514
	1 -2.735112 -1.392638 1.690776		1 1.521648 2.043710 1.691108
	6 -2.112012 -3.725308 0.283942		6 -0.493380 3.615240 0.897104
	1 -2.725170 -3.976968 1.144437		1 -0.016278 3.960329 1.811723
	6 -1.524209 -4.740684 -0.487477		6 -1.505890 4.373155 0.301449
	1 -1.715302 -5.780335 -0.235948		1 -1.822543 5.308757 0.753886
	6 -0.660604 -4.435261 -1.543659		6 -2.103831 3.926238 -0.879738
	1 -0.193722 -5.234804 -2.110489		1 -2.886908 4.517009 -1.347569
	6 -0.381692 -3.105657 -1.861446		6 -1.705908 2.721018 -1.461089
	1 0.288237 -2.853946 -2.676858		1 -2.183213 2.372353 -2.372936
	44 1.266612 1.055519 0.532666		6 2.055397 1.467092 -0.349468
	6 -3.063533 -0.274614 -0.174116		6 3.048757 0.329176 -0.546348
	6 -3.115272 1.241745 -0.000389		6 2.478510 -0.090276 -1.697170
	6 -2.408137 1.512933 -1.115512		1 2.502306 2.461484 -0.477850
	1 -4.025114 -0.756323 -0.382321		1 1.415799 1.674163 -2.471606
	1 -2.692891 -0.288000 -2.388714		6 4.283534 -0.042508 0.215083
	6 -3.767900 2.065863 1.063378		1 4.008742 -0.278507 1.251716
	1 -3.286785 1.840328 2.025490		1 4.718445 -0.954789 -0.212465
	1 -3.582136 3.128610 0.864847		6 2.902111 -1.042229 -2.776208
	6 -2.038450 2.744919 -1.877540		1 2.084446 -1.728240 -3.032834
	1 -0.960354 2.741877 -2.078971		1 3.727256 -1.667213 -2.410863
	1 -2.241773 3.630452 -1.263254		6 3.345660 -0.316166 -4.061514
	6 1.153140 2.791931 1.880365		1 2.535684 0.296941 -4.469434
	6 0.448783 3.096761 0.677873		1 4.196721 0.342986 -3.864058
	6 2.519799 2.515051 1.523287		1 3.642151 -1.035216 -4.831858
	6 1.374461 3.058001 -0.424480		6 5.343446 1.075162 0.230251
	6 2.649063 2.699168 0.104893		1 4.945537 1.990872 0.679496
	1 0.731846 2.750265 2.875683		1 6.220929 0.772499 0.810642
	1 -0.607710 3.314294 0.609099		1 5.674294 1.317526 -0.784803
	1 3.316582 2.239479 2.201003		1 0.797509 -3.258157 1.603708
	1 1.158001 3.281670 -1.459112		6 0.269999 -3.108292 0.674370
	1 3.553637 2.550282 -0.468372		6 0.830467 -2.613492 -0.541286
	53 2.788594 -0.685417 -1.091653		6 -1.116511 -3.289605 0.469973
	6 -0.099411 -0.966827 1.872883		6 -0.221265 -2.520153 -1.516935
	8 1.069419 -0.519246 2.027787		1 1.871321 -2.389419 -0.710613
	8 -0.525188 -2.008572 2.629421		44 -0.560851 -0.940029 0.053767
	6 0.415894 -2.520714 3.585389		6 -1.416628 -2.943536 -0.883783
	1 1.316479 -2.883392 3.084789		1 -1.837744 -3.598461 1.214736
	1 0.696440 -1.749239 4.307217		1 -0.117201 -2.233557 -2.553619
	1 -0.097805 -3.340672 4.088820		53 -3.207535 -0.231124 -0.022529
	6 -5.281876 1.817141 1.186996		1 -2.393934 -2.983021 -1.341955
	1 -5.494878 0.767711 1.416003		6 2.406430 -0.063744 4.302624
	1 -5.710986 2.426141 1.988571		8 1.998092 0.401804 3.006284
	1 -5.799954 2.067928 0.255780		6 0.989227 -0.305164 2.432858
	6 -2.792797 2.867280 -3.215886		8 0.524405 -1.304065 2.962156
	1 -3.874198 2.915693 -3.054128		1 2.799900 -1.081116 4.241232
	1 -2.486985 3.771312 -3.751527		1 3.183534 0.627874 4.628266
	1 -2.588989 2.010851 -3.867237		1 1.564026 -0.051238 4.998300
**TS12a**	6 -1.157178 1.452115 -1.623013	**INT9a**	6 -1.670056 1.404636 -1.513744
	6 -0.282753 0.289303 -1.916668		6 -0.698830 0.469177 -2.075245
	6 -0.834798 2.268845 -0.520708		6 -1.261857 2.233260 -0.448259
	6 0.397076 1.874187 0.269026		6 0.113622 1.967308 0.125282
	7 0.247678 0.594509 0.980908		7 0.128979 0.682637 0.853524
	1 -0.554990 -0.283693 -2.800720		1 -1.056565 -0.255190 -2.802183
	1 0.660070 2.658554 0.979137		1 0.413445 2.781106 0.788891
	6 -1.603070 3.394420 -0.240688		6 -2.136165 3.192224 0.052680
	1 -1.349996 4.016871 0.614394		1 -1.827794 3.813509 0.889062
	6 -2.694927 3.723353 -1.051302		6 -3.407722 3.345761 -0.510397
	1 -3.291570 4.603302 -0.827956		1 -4.083987 4.100159 -0.118105
	6 -3.013091 2.919069 -2.146991		6 -3.814875 2.525546 -1.565733
	1 -3.860247 3.170454 -2.779240		1 -4.805811 2.640826 -1.994793
	6 -2.248224 1.786579 -2.432418		6 -2.953744 1.546653 -2.058619
	1 -2.502628 1.155038 -3.279868		1 -3.268505 0.896169 -2.870033
	44 0.060341 -1.078808 -0.227835		44 0.546201 -0.925680 -0.332515

Species	Cartesian coordinates				Species	Cartesian coordinates			
	6	1.528680	1.687138	-0.771974		6	1.140034	1.823274	-1.046795
	1	1.699814	2.625040	-1.316798		1	1.181549	2.765935	-1.611873
	6	2.762678	1.043551	-0.229063		6	2.460888	1.415769	-0.463020
	6	2.618942	-0.248941	-0.545929		6	2.507029	0.146464	-0.028989
	6	1.126611	0.544132	-1.709676		6	0.661198	0.681299	-1.919715
	1	1.772698	0.357111	-2.563345		1	1.359810	0.274908	-2.644908
	6	-0.978354	-3.205768	0.022845		6	-0.040171	-3.036347	-1.097206
	6	-0.053418	-2.998615	1.104406		6	0.211210	-3.184705	0.326978
	6	-0.272590	-3.188732	-1.207205		6	1.172586	-2.684104	-1.725537
	6	1.229901	-2.876664	0.546540		6	1.547324	-2.853210	0.567665
	6	1.089673	-2.961610	-0.897863		6	2.145470	-2.518308	-0.707959
	1	-2.037007	-3.376023	0.134108		1	-0.984529	-3.223833	-1.585441
	1	-0.304861	-2.907551	2.150047		1	-0.520837	-3.472126	1.065944
	1	-0.698499	-3.322255	-2.191680		1	1.324244	-2.520193	-2.783909
	1	2.140604	-2.733936	1.107857		1	2.031995	-2.859204	1.532207
	1	1.886395	-2.940431	-1.624978		1	3.183267	-2.287376	-0.878925
	53	-2.772969	-0.665154	0.400606		53	-2.579955	-1.428675	0.306128
	6	3.905085	1.751060	0.448220		6	3.502470	2.494309	-0.237390
	1	3.496772	2.283211	1.318588		1	3.002209	3.369953	0.200974
	1	4.615714	1.018722	0.845942		1	4.245054	2.172096	0.498025
	6	3.692876	-1.271286	-0.768550		6	3.656664	-0.393398	0.806460
	1	3.468072	-2.260139	-0.370813		1	3.392890	-1.327910	1.299334
	1	4.552509	-0.909255	-0.186436		1	3.822392	0.311947	1.632170
	6	0.496530	0.485679	2.304781		6	0.153854	0.626128	2.210312
	8	0.508890	-0.558767	2.954767		8	0.390698	-0.377769	2.874656
	8	0.748776	1.697790	2.917344		8	-0.144646	1.830351	2.796103
	6	0.951571	1.621232	4.329350		6	-0.193168	1.803573	4.225908
	1	0.067609	1.220196	4.832941		1	-0.955181	1.102747	4.576757
	1	1.807248	0.985105	4.573159		1	0.772379	1.509391	4.646987
	1	1.137781	2.646006	4.656515		1	-0.445091	2.820359	4.531520
	6	4.644879	2.752969	-0.456572		6	4.217504	2.934794	-1.527916
	1	5.444588	3.252333	0.099199		1	4.925854	3.744697	-1.324221
	1	5.095747	2.250978	-1.318848		1	4.771094	2.103070	-1.975471
	1	3.970197	3.527409	-0.834692		1	3.505014	3.297916	-2.276301
	6	4.125016	-1.393951	-2.242393		6	4.991417	-0.578371	0.056306
	1	4.946019	-2.110811	-2.340256		1	5.753430	-0.982318	0.731738
	1	3.304054	-1.738410	-2.880491		1	4.904732	-1.265354	-0.792234
	1	4.464489	-0.429242	-2.631300		1	5.364366	0.368945	-0.340424
**TS13a**	6	2.873851	-0.286027	0.991808	**INT10a**	6	0.188332	1.421980	-1.985729
	6	1.599360	0.173264	1.500066		6	-0.136449	0.027276	-1.559163
	6	1.378813	1.646547	1.403750		6	1.186589	-0.776871	-1.644544
	6	3.236161	0.249594	-0.272522		6	1.868753	1.221860	-0.189509
	6	2.069038	0.993578	-0.905738		6	1.224730	2.063886	-1.271812
	7	0.917262	0.091286	-0.809691		7	0.867547	0.854116	0.833032
	1	1.104058	-0.395520	2.276069		1	-0.919263	-0.419046	-2.172925
	1	2.275364	1.278275	-1.942459		1	2.687777	1.763383	0.285356
	6	4.473180	-0.050046	-0.819791		6	1.593460	3.369607	-1.579940
	1	4.748587	0.335298	-1.797304		1	2.391804	3.853329	-1.021440
	6	5.350764	-0.890952	-0.117131		6	0.931174	4.062075	-2.602794
	1	6.317144	-1.135590	-0.549229		1	1.215209	5.083522	-2.839556
	6	4.985963	-1.445791	1.113435		6	-0.092780	3.434699	-3.315022
	1	5.672531	-2.105179	1.635537		1	-0.602965	3.967033	-4.113488
	6	3.737536	-1.160479	1.668111		6	-0.461425	2.119481	-3.011078
	1	3.452010	-1.576924	2.630437		1	-1.251515	1.630844	-3.576975
	44	-0.975247	-1.206572	0.406854		6	2.366848	-0.055340	-0.906296
	6	1.782375	2.202216	-0.002202		6	2.585829	-1.366260	-0.175240
	6	0.397668	2.838248	-0.007568		6	1.594216	-2.010351	-0.828305
	6	0.043572	2.357265	1.199818		1	3.189808	0.230904	-1.574455
	1	2.633952	2.891841	-0.003842		1	1.389973	-0.906257	-2.719647
	1	1.991163	2.058967	2.227686		6	3.639074	-1.778135	0.805783
	6	-0.482657	-2.442134	2.213696		1	3.576047	-1.133414	1.692514
	6	-1.273957	-3.187256	1.293417		1	3.439785	-2.800727	1.148057
	6	-1.262679	-1.294431	2.620242		6	1.241499	-3.462032	-0.941688
	6	-2.532327	-2.518112	1.128745		1	0.190123	-3.621373	-0.685140
	6	-2.525493	-1.351935	1.977639		1	1.834005	-4.026971	-0.210929
	1	0.504588	-2.710925	2.565729		1	-1.189423	3.084894	-0.397992
	1	-0.966610	-4.086360	0.775399		6	-1.759894	2.401100	0.211858
	1	-0.955000	-0.534626	3.327306		6	-1.712828	2.300965	1.644928
	1	-3.348329	-2.840580	0.497643		6	-2.771814	1.500702	-0.252871
	1	-3.329131	-0.636399	2.077391		6	-2.685895	1.376726	2.066082

Species	Cartesian coordinates				Species	Cartesian coordinates			
	53	-2.710626	0.248542	-1.262053		1	-1.020071	2.821224	2.290598
	6	0.859877	-0.961413	-1.610917		44	-1.036645	0.320358	0.437540
	8	0.032097	-1.892687	-1.405716		6	-3.315293	0.853330	0.905368
	8	1.697486	-1.009966	-2.672999		1	-3.109738	1.384409	-1.273093
	6	1.491105	-2.098911	-3.586892		1	-2.849564	1.043633	3.081667
	6	-0.272180	3.675620	-1.047652		53	-2.152286	-2.137013	0.040912
	1	-0.449221	3.049086	-1.932089		1	-4.091923	0.102803	0.895332
	1	-1.264390	3.970220	-0.688141		6	2.817182	1.308325	3.812409
	6	-1.150148	2.510457	2.085121		8	2.459667	1.281711	2.422864
	1	-1.638638	1.536546	2.207954		6	1.172468	0.910182	2.167696
	1	-1.882421	3.147042	1.576377		8	0.385447	0.645979	3.074270
	1	0.482540	-2.066339	-4.005865		1	2.229393	2.056380	4.350505
	1	2.233699	-1.962597	-4.373946		1	3.875640	1.569504	3.835410
	1	1.639393	-3.058783	-3.086050		1	2.652735	0.332021	4.274511
	6	0.539422	4.919537	-1.448464		6	5.066712	-1.691857	0.233870
	1	0.017897	5.487132	-2.225301		1	5.303275	-0.673129	-0.091174
	1	0.697196	5.584624	-0.592739		1	5.807497	-1.981945	0.986197
	1	1.523818	4.645397	-1.843447		1	5.184516	-2.352923	-0.630943
	6	-0.812845	3.099724	3.466458		6	1.498305	-4.021868	-2.353626
	1	-0.102284	2.467710	4.011945		1	2.549023	-3.909306	-2.641830
	1	-0.366044	4.095073	3.373774		1	1.245899	-5.086269	-2.399619
	1	-1.713734	3.189879	4.081394		1	0.888987	-3.503786	-3.101638
**TS14a**	6	3.162193	-0.852192	0.111396	**INT11a**	6	-3.033470	1.067484	-0.887657
	6	1.964195	-1.711923	0.136031		6	-1.864839	1.620095	-1.510520
	6	3.094389	0.402849	0.756491		6	-2.949607	0.654572	0.472026
	6	1.705212	0.833669	1.185522		6	-1.563305	0.592084	1.098154
	7	0.874244	0.926146	-0.023130		7	-0.879186	-0.524681	0.418267
	1	2.103127	-2.710792	-0.269011		1	-1.907520	1.849086	-2.573452
	1	1.734127	1.788578	1.715213		1	-1.628816	0.444373	2.181790
	6	4.233610	1.193014	0.860487		6	-4.109956	0.221881	1.116619
	1	4.172132	2.164217	1.343062		1	-4.053206	-0.101815	2.150400
	6	5.445700	0.748028	0.317676		6	-5.329263	0.201664	0.439091
	1	6.331890	1.371407	0.394632		1	-6.227994	-0.113926	0.962014
	6	5.513955	-0.483997	-0.335441		6	-5.408394	0.575996	-0.911242
	1	6.453973	-0.824040	-0.759946		1	-6.360520	0.538788	-1.431712
	6	4.374575	-1.284910	-0.439199		6	-4.265044	0.996565	-1.573610
	1	4.430951	-2.255167	-0.926972		1	-4.311203	1.300136	-2.616590
	44	0.066466	-0.848652	-0.635760		44	0.671014	-0.001031	-0.841032
	6	1.091506	-0.284759	2.073329		6	-0.815829	1.898137	0.750383
	1	1.630016	-0.339874	3.027446		1	-1.435558	2.730127	1.125536
	6	-0.405696	-0.273511	2.224673		6	0.610814	2.045769	1.244644
	6	-0.820600	-1.236097	1.352259		6	1.539283	1.437667	0.486652
	6	1.109690	-1.607929	1.298790		6	-0.754678	1.974640	-0.768875
	1	0.966619	-2.523998	1.868296		1	-0.045729	2.671723	-1.203908
	6	0.383262	-0.743382	-2.886347		6	0.234889	-0.228138	-3.102084
	6	-0.955493	-0.391689	-2.579049		6	0.546360	-1.554949	-2.729391
	6	0.576751	-2.098325	-2.476183		6	1.399884	0.608412	-2.871473
	6	-1.605573	-1.559006	-2.023427		6	1.865002	-1.546835	-2.218254
	6	-0.660976	-2.610985	-1.969856		6	2.406363	-0.212896	-2.318820
	1	1.132647	-0.078387	-3.290039		1	-0.695079	0.095149	-3.547676
	1	-1.409015	0.579498	-2.710779		1	-0.127994	-2.398296	-2.723532
	1	1.499555	-2.654913	-2.572114		1	1.494451	1.653507	-3.133525
	1	-2.637727	-1.580881	-1.701679		1	2.389824	-2.404282	-1.824368
	1	-0.835269	-3.617362	-1.614002		1	3.412359	0.080814	-2.057227
	53	-3.662436	1.055060	-0.266844		53	2.138126	-1.677429	1.073272
	6	-1.229020	0.608620	3.105987		6	0.852389	2.925649	2.456297
	1	-1.412028	1.535462	2.539630		1	0.143220	2.629782	3.243003
	1	-2.221278	0.168671	3.246365		1	1.849512	2.735638	2.865013
	6	-2.011148	-2.150489	1.423206		6	3.023878	1.706383	0.540965
	1	-2.586538	-2.120032	0.497456		1	3.590119	0.796717	0.331348
	1	-2.670971	-1.749794	2.202949		1	3.301598	2.001536	1.562503
	6	0.963476	2.041288	-0.813641		6	-1.332376	-1.800680	0.533139
	8	0.521091	2.153936	-1.949847		8	-1.037914	-2.737637	-0.202633
	8	1.606779	3.072132	-0.184820		8	-2.166137	-1.970233	1.611680
	6	1.665386	4.290563	-0.937547		6	-2.670211	-3.298616	1.775825
	1	2.207824	4.142815	-1.874946		1	-3.244935	-3.613571	0.900123
	1	0.660654	4.654837	-1.165370		1	-1.854124	-4.008600	1.931944
	1	2.192765	5.002559	-0.301261		1	-3.313927	-3.263437	2.656606
	6	-0.582658	0.941545	4.459417		6	0.694051	4.435252	2.194025
	1	-1.239104	1.600282	5.034563		1	0.831452	5.008377	3.117493

Species	Cartesian coordinates				Species	Cartesian coordinates			
	1	-0.407066	0.039662	5.055232		1	1.431638	4.787763	1.465933
	1	0.375055	1.457420	4.335699		1	-0.300051	4.677431	1.802022
	6	-1.640789	-3.602660	1.772303		6	3.466705	2.823112	-0.423724
	1	-2.544008	-4.217458	1.818792		1	4.548031	2.986188	-0.356911
	1	-0.978465	-4.039722	1.017124		1	3.232742	2.572384	-1.463972
	1	-1.139628	-3.668364	2.743798		1	2.967245	3.769728	-0.194099

Table S5.The optimized cartesian coordinates (Å) for reaction **b**, located at IDSCRF-B3LYP/BS1 level in dioxane solvent.

Species	Cartesian coordinates				Species	Cartesian coordinates			
**R1b**	6	3.263848	1.537550	-1.623793	**R2b**	6	-0.548287	0.049412	0.001466
	6	3.043977	0.545426	-0.648658		6	-1.972823	0.017504	0.000738
	6	2.486917	0.858672	0.575796		6	-2.709964	1.218292	-0.002448
	6	2.138897	2.202078	0.819849		6	-2.656756	-1.214382	0.003203
	6	2.356104	3.182802	-0.144193		6	-4.101198	1.181991	-0.003128
	6	2.929715	2.857269	-1.389401		1	-2.180925	2.165786	-0.004341
	1	2.308952	0.097383	1.330447		6	-4.048189	-1.239070	0.002473
	1	1.689703	2.473323	1.771030		1	-2.086919	-2.137932	0.005654
	1	2.075504	4.211715	0.062573		6	-4.772149	-0.043786	-0.000680
	1	3.093668	3.627177	-2.138718		1	-4.663724	2.110781	-0.005575
	6	3.898265	0.796299	-2.819280		1	-4.569649	-2.191538	0.004371
	6	5.284999	0.327736	-2.343487		1	-5.857978	-0.067568	-0.001229
	6	5.075755	-0.612846	-1.418864		6	0.666205	0.069787	0.002117
	6	3.551173	-0.766961	-1.284116		6	2.102938	0.175704	0.002649
	1	3.826508	1.263685	-3.797943		8	2.717464	1.225738	-0.000342
	1	6.221276	0.762691	-2.669573		8	2.682987	-1.043717	0.006914
	1	5.799493	-1.133697	-0.805041		6	4.122796	-1.032989	0.007795
	1	3.158542	-1.700920	-0.889444		1	4.502551	-0.522224	0.895854
	7	3.186552	-0.512347	-2.706863		1	4.420335	-2.080886	0.011274
	6	1.890393	-0.727400	-3.129657		1	4.503685	-0.527805	-0.882967
	8	1.148774	-1.553596	-2.620208					
	8	1.616257	0.044291	-4.203872					
	6	0.330841	-0.068971	-4.917453					
	6	-0.819983	0.311949	-3.981057					
	1	-0.921372	-0.410735	-3.171382					
	1	-0.646435	1.303289	-3.551221					
	1	-1.756908	0.344516	-4.546352					
	6	0.482708	0.961648	-6.038592					
	1	0.640598	1.961842	-5.624868					
	1	1.333954	0.713475	-6.679075					
	1	-0.420705	0.980909	-6.655104					
	6	0.174411	-1.479590	-5.493041					
	1	-0.724448	-1.523037	-6.116401					
	1	1.035258	-1.731706	-6.120140					
	1	0.087211	-2.219524	-4.697299					
**P1b**	6	-4.011401	-3.221382	0.240813	**P2b**	6	2.147859	-1.992156	0.212969
	6	-2.916414	-3.214594	1.103154		6	0.981989	-1.605222	-0.696242
	6	-1.741660	-2.528196	0.763128		6	-0.292664	-2.118829	0.037345
	6	-2.785739	-1.832253	-1.309225		6	0.861795	-0.230717	1.069343
	6	-3.941127	-2.543252	-0.977638		6	2.070810	-1.130477	1.324176
	1	-4.910716	-3.767590	0.510927		7	0.876966	-0.156437	-0.410534
	1	-2.954738	-3.767940	2.038054		6	1.554066	0.773892	-1.161378
	1	-2.736922	-1.284194	-2.245750		8	1.909942	0.580094	-2.314255
	1	-4.782090	-2.562124	-1.664546		8	1.705017	1.922104	-0.460841
	6	-1.693899	-1.798201	-0.442913		6	2.303631	3.118107	-1.078975
	6	-0.537212	-2.626519	1.589630		1	1.059651	-1.831513	-1.756833
	6	0.863064	-1.715685	-0.276528		1	0.841045	0.742266	1.554021
	6	0.670861	-2.298141	1.112165		6	2.961870	-1.243030	2.379902
	1	-0.639215	-3.045121	2.588194		1	2.904470	-0.584206	3.242414
	1	1.556061	-2.428419	1.727594		6	3.961380	-2.226421	2.300451
	6	-0.432044	-1.045830	-0.804839		1	4.686930	-2.319258	3.103627
	6	1.843951	-0.541687	-0.229527		6	4.037937	-3.080704	1.199517
	6	1.109187	0.587065	-0.105649		1	4.822683	-3.830636	1.156109
	1	1.196629	-2.495378	-0.973747		6	3.118102	-2.979433	0.143063
	1	-0.368150	-0.953805	-1.896409		1	3.180019	-3.650899	-0.708925
	7	-0.270121	0.342245	-0.244401		6	-0.381933	-1.150400	1.278050
	6	-1.199564	1.313061	0.074430		6	-1.713894	-0.680885	0.695801
	8	-2.407816	1.043026	-0.441996		6	-1.624778	-1.530341	-0.371335
	8	-0.899511	2.299693	0.733746		1	-0.409615	-1.605222	2.271559

Species	Cartesian coordinates				Species	Cartesian coordinates			
	6	-3.559973	1.930291	-0.154712		1	-0.262139	-3.193648	0.229404
	6	-3.295528	3.327851	-0.723038		6	1.451271	3.576736	-2.266151
	1	-4.199046	3.937163	-0.618372		1	1.836090	4.527914	-2.647822
	1	-2.474570	3.818892	-0.201853		1	1.471941	2.840423	-3.069721
	1	-3.051245	3.264085	-1.787901		1	0.414328	3.730936	-1.951761
	6	-4.705821	1.252825	-0.908140		6	2.246023	4.141845	0.057428
	1	-4.485233	1.196878	-1.978112		1	2.818528	3.792172	0.921502
	1	-4.872776	0.239593	-0.535468		1	2.668299	5.094876	-0.274382
	1	-5.624934	1.831329	-0.776397		1	1.212389	4.311478	0.372465
	6	-3.837274	1.948233	1.351053		6	3.754576	2.831243	-1.477996
	1	-3.021673	2.422142	1.897475		1	4.228750	3.754764	-1.825586
	1	-4.759552	2.505413	1.543917		1	4.319102	2.462634	-0.615822
	1	-3.971749	0.928190	1.723645		1	3.801470	2.088578	-2.274367
	6	3.299601	-0.699465	-0.308782		6	-2.605899	0.340563	1.232605
	6	3.900284	-1.938651	-0.008657		6	-2.314456	0.882164	2.500994
	6	4.143827	0.350401	-0.726976		6	-3.738458	0.820190	0.544736
	6	5.281868	-2.107455	-0.078519		6	-3.124914	1.863328	3.065504
	1	3.284814	-2.781035	0.288888		1	-1.448427	0.523655	3.048859
	6	5.523479	0.179083	-0.793612		6	-4.543900	1.803591	1.111540
	1	3.715929	1.294227	-1.045306		1	-3.973214	0.416210	-0.431243
	6	6.103707	-1.047815	-0.463213		6	-4.243576	2.328457	2.371573
	1	5.715266	-3.073063	0.166641		1	-2.883922	2.265007	4.045443
	1	6.147553	1.004984	-1.123509		1	-5.411784	2.164742	0.566992
	1	7.180119	-1.179757	-0.520679		1	-4.876981	3.095020	2.808766
	6	1.598278	2.003505	0.000055		6	-2.361229	-1.908257	-1.581551
	8	1.531750	2.808242	-0.906048		8	-1.931766	-2.726461	-2.378423
	8	2.182398	2.230909	1.184549		8	-3.556778	-1.290985	-1.736602
	6	2.692030	3.564455	1.369466		6	-4.290532	-1.660888	-2.917553
	1	3.466641	3.786007	0.631070		1	-5.213737	-1.083251	-2.882090
	1	1.883618	4.292321	1.272613		1	-3.719080	-1.417504	-3.816277
	1	3.107372	3.580663	2.376287		1	-4.507325	-2.731617	-2.914496
**TS2b**	6	2.238495	-2.426713	1.117454	**TS2b-n**	6	-3.105766	-1.829128	-0.090255
	6	2.368642	-1.193946	1.782795		6	-2.713767	-1.759745	1.259685
	6	3.010803	-1.101580	3.005305		6	-3.222721	-2.635544	2.201497
	6	3.548546	-2.278942	3.556167		6	-4.168190	-3.584895	1.771965
	6	3.419900	-3.501602	2.897050		6	-4.560478	-3.652153	0.435240
	6	2.748703	-3.591551	1.664068		6	-4.020537	-2.773429	-0.522118
	1	3.107935	-0.152366	3.525381		1	-2.910448	-2.592072	3.240898
	1	4.072906	-2.234221	4.506537		1	-4.598537	-4.274570	2.492573
	1	3.845742	-4.397319	3.340344		1	-5.292921	-4.393190	0.127557
	1	2.647990	-4.548465	1.159097		1	-4.323848	-2.838276	-1.563746
	6	1.433229	-2.117990	-0.153949		6	-2.284188	-0.743393	-0.806174
	6	0.010167	-1.796163	0.364084		6	-0.833509	-1.286545	-0.777269
	6	0.151519	-0.500633	1.024173		6	-0.450591	-1.213241	0.605030
	6	1.659457	-0.166209	0.889415		6	-1.679047	-0.623934	1.329918
	1	1.541150	-2.803976	-0.989711		1	-2.675790	-0.363489	-1.745453
	1	-0.521601	-2.590925	0.876817		1	-0.612446	-2.175345	-1.357482
	1	-0.328159	-0.390534	1.991519		1	0.087257	-2.031111	1.064575
	1	1.962228	0.869782	0.986704		1	-1.538565	-0.139749	2.288223
	7	1.905755	-0.747393	-0.443586		7	-2.188197	0.270696	0.271991
	6	2.968240	-0.488385	-1.271743		6	-3.146659	1.215962	0.562655
	8	3.275994	-1.219498	-2.205290		8	-3.342377	1.657914	1.683891
	8	3.545294	0.688211	-0.951327		8	-3.756790	1.621482	-0.576414
	6	4.644557	1.249453	-1.760237		6	-4.831258	2.634062	-0.547023
	6	4.159733	1.518984	-3.187934		6	-5.233676	2.733014	-2.020350
	1	3.924734	0.587950	-3.704478		1	-6.036164	3.466575	-2.140487
	1	3.268678	2.154952	-3.174413		1	-5.590124	1.767164	-2.390095
	1	4.941115	2.043603	-3.746975		1	-4.384318	3.047351	-2.634168
	6	4.952338	2.560136	-1.032217		6	-4.279025	3.970493	-0.041778
	1	5.262501	2.365513	-0.001533		1	-3.429746	4.290084	-0.654601
	1	5.760351	3.091855	-1.543103		1	-3.958674	3.893299	0.997343
	1	4.070008	3.206396	-1.009991		1	-5.055473	4.738651	-0.116365
	6	5.852848	0.308798	-1.721962		6	-6.001085	2.128243	0.303009
	1	6.702996	0.785034	-2.220891		1	-5.718441	2.046010	1.352687
	1	6.139200	0.100904	-0.686311		1	-6.333248	1.147944	-0.052723
	1	5.632214	-0.633459	-2.223885		1	-6.841905	2.823931	0.217531
	44	-1.340669	-0.665184	-0.855309		44	0.861567	0.082102	-0.942527
	6	-1.334751	0.077090	-3.004815		6	1.910192	1.534440	-2.281193
	6	-0.255116	-0.846098	-2.903188		6	1.035456	2.304505	-1.443228
	6	-0.809126	-2.122143	-2.567558		6	-0.303609	1.929509	-1.727890

Species	Cartesian coordinates				Species	Cartesian coordinates			
	6	-2.219344	-1.988009	-2.453349		6	-0.261767	0.931508	-2.757276
	6	-2.553845	-0.619115	-2.717477		6	1.094437	0.695005	-3.104902
	1	-1.262452	1.130047	-3.238285		1	2.987834	1.599159	-2.311883
	1	0.794106	-0.642814	-3.063590		1	1.347567	3.033650	-0.707508
	1	-0.254241	-3.038840	-2.425611		1	-1.196487	2.321607	-1.265783
	1	-2.916603	-2.773834	-2.198634		1	-1.115005	0.445361	-3.208203
	1	-3.543347	-0.186667	-2.712405		1	1.451067	-0.011147	-3.841563
	53	-3.344532	-1.514965	0.958701		53	2.392584	-2.255394	-1.416790
	6	-0.840935	1.315092	0.510339		6	1.222140	-0.006297	1.389014
	6	-1.886314	1.244884	-0.233327		6	2.169769	0.485265	0.673433
	6	-0.078262	2.295131	1.279560		6	3.450427	1.172695	0.744766
	6	0.683350	3.268790	0.608196		6	4.521530	0.817432	-0.095976
	6	-0.133241	2.316485	2.684274		6	3.623659	2.220895	1.670459
	6	1.370947	4.244503	1.329482		6	5.740704	1.484455	0.002089
	1	0.728098	3.252610	-0.476418		1	4.389532	0.002702	-0.800223
	6	0.557351	3.294399	3.398574		6	4.840149	2.896838	1.745016
	1	-0.737471	1.582795	3.209318		1	2.803013	2.488687	2.325890
	6	1.312786	4.257762	2.724953		6	5.902574	2.529747	0.915659
	1	1.951518	4.994931	0.800455		1	6.565736	1.189391	-0.640055
	1	0.498155	3.308750	4.483086		1	4.959987	3.708078	2.457719
	1	1.849844	5.017774	3.285112		1	6.851543	3.054510	0.981224
	6	-2.943776	2.177485	-0.652288		6	1.074078	-0.379318	2.804160
	8	-3.350761	2.306873	-1.793199		8	0.421862	-1.293419	3.275232
	8	-3.408833	2.886687	0.392658		8	1.821270	0.454244	3.565018
	6	-4.462995	3.818180	0.085324		6	1.848583	0.142171	4.970300
	1	-4.733551	4.274279	1.037017		1	2.498081	0.892537	5.420179
	1	-4.112722	4.576720	-0.619143		1	0.843407	0.198122	5.394983
	1	-5.318986	3.295787	-0.348194		1	2.249873	-0.860815	5.133484
**TS3b**	6	-1.195781	2.804614	0.156987	**TS3b-n**	6	0.006334	-0.887417	-0.120896
	6	-1.790310	2.274785	1.318353		6	-0.207103	-1.038413	1.413497
	6	-2.161093	3.100474	2.367898		6	0.229688	0.362968	1.697066
	6	-1.946638	4.481792	2.229450		6	0.713298	0.862405	0.532760
	6	-1.354847	5.005418	1.078038		44	1.826089	-0.320080	-1.108859
	6	-0.955869	4.164440	0.026924		6	-1.426890	-0.900886	-0.696888
	1	-2.614594	2.700376	3.270638		6	-1.755778	-1.254341	1.483626
	1	-2.248681	5.152623	3.028684		6	-1.828195	-2.375686	-0.571804
	1	-1.204637	6.077776	0.992261		6	-2.030230	-2.593975	0.803944
	1	-0.491722	4.576611	-0.864900		6	-2.359968	-3.852109	1.282812
	6	-0.883231	1.593214	-0.733290		6	-2.508601	-4.894811	0.353645
	6	0.303259	0.939514	0.005983		6	-2.305711	-4.676751	-1.010898
	6	-0.357546	0.259671	1.233718		6	-1.945778	-3.407705	-1.491436
	6	-1.831233	0.761876	1.110432		1	-2.512311	-4.031530	2.343429
	1	-0.785226	1.752957	-1.803364		1	-2.789000	-5.884951	0.701478
	1	1.102794	1.714950	0.295453		1	-2.431410	-5.498479	-1.710115
	1	0.095215	0.480662	2.203310		1	-1.782758	-3.245053	-2.553525
	1	-2.574110	0.187880	1.658203		1	-1.580664	-0.408049	-1.652221
	7	-1.981052	0.681781	-0.363197		1	0.642666	-1.732935	-0.561682
	6	-3.176496	0.712283	-1.051354		1	0.351416	-1.839085	1.903106
	8	-3.275345	1.110602	-2.201780		1	-2.216769	-1.063391	2.448081
	8	-4.161476	0.195226	-0.289771		7	-2.180288	-0.307490	0.427905
	6	-5.511410	-0.036290	-0.837520		6	-3.475569	0.157931	0.358183
	6	-6.248059	-0.656366	0.352231		8	-4.233876	0.178152	1.314045
	1	-7.281675	-0.883897	0.075405		8	-3.730830	0.635424	-0.881264
	1	-5.760280	-1.583218	0.667564		6	-5.032732	1.252071	-1.205617
	1	-6.261468	0.033947	1.200821		6	-4.863699	1.626011	-2.680110
	6	-5.434268	-1.024413	-2.005267		1	-5.775777	2.101330	-3.052503
	1	-4.893563	-0.593985	-2.848698		1	-4.666376	0.736147	-3.285197
	1	-4.929329	-1.943233	-1.690987		1	-4.032373	2.325315	-2.809690
	1	-6.446078	-1.285798	-2.331389		6	-6.154740	0.223657	-1.031097
	6	-6.148929	1.297305	-1.239396		1	-6.269703	-0.058147	0.015676
	1	-6.141205	1.992150	-0.393713		1	-5.942392	-0.674612	-1.619409
	1	-5.614831	1.750956	-2.074478		1	-7.098396	0.648448	-1.388053
	1	-7.190377	1.131146	-1.533304		6	-5.247983	2.502326	-0.347447
	44	2.294403	0.577411	-0.680274		1	-4.409143	3.195626	-0.465339
	6	3.409681	-0.306029	-2.356577		1	-5.345795	2.242142	0.706611
	6	2.317546	0.460864	-2.906021		1	-6.159535	3.015064	-0.670974
	6	2.516890	1.815399	-2.532102		6	2.824554	1.228837	-2.297004
	6	3.758223	1.903024	-1.797957		6	1.467465	1.114423	-2.771812
	6	4.312877	0.605029	-1.716493		6	1.294641	-0.197366	-3.286131
	1	3.565169	-1.369469	-2.470815		6	2.559107	-0.885810	-3.176316

Species	Cartesian coordinates				Species	Cartesian coordinates			
	1	1.481754	0.055577	-3.457816		6	3.498381	-0.001275	-2.597483
	1	1.871694	2.647893	-2.780867		1	3.269892	2.109135	-1.857058
	1	4.179618	2.800975	-1.365695		1	0.715482	1.888812	-2.719725
	1	5.219940	0.334266	-1.193908		1	0.391107	-0.602160	-3.722563
	53	3.437379	-0.036597	1.779544		1	2.750252	-1.911074	-3.465579
	6	-0.148277	-1.124448	0.684091		1	4.524203	-0.238116	-2.351881
	6	0.712143	-0.970932	-0.357725		53	3.609998	-1.222708	0.808871
	6	-0.929172	-2.300570	1.098924		6	0.846743	2.299187	0.198836
	6	-1.134425	-2.533356	2.471714		6	2.036366	2.998660	0.462454
	6	-1.508828	-3.184979	0.170755		6	-0.245951	2.989657	-0.352411
	6	-1.866718	-3.637452	2.903738		6	2.124799	4.361544	0.184715
	1	-0.694094	-1.858255	3.200294		1	2.878118	2.468223	0.896871
	6	-2.248103	-4.283107	0.606564		6	-0.143760	4.351060	-0.643794
	1	-1.407881	-2.991655	-0.892419		1	-1.175217	2.458304	-0.538004
	6	-2.425245	-4.516541	1.972682		6	1.039806	5.041538	-0.376509
	1	-2.001882	-3.811399	3.967501		1	3.044271	4.895188	0.409285
	1	-2.695466	-4.952961	-0.122559		1	-0.996109	4.874720	-1.068303
	1	-3.001348	-5.373769	2.309417		1	1.115347	6.102364	-0.597569
	6	0.920294	-2.020815	-1.397247		6	-0.005972	1.117416	2.950542
	8	0.532772	-1.966826	-2.552166		8	0.266502	2.284497	3.152296
	8	1.591398	-3.073197	-0.888528		8	-0.560972	0.309283	3.888224
	6	1.761094	-4.196175	-1.772587		6	-0.806944	0.927125	5.162659
	1	2.384118	-4.903757	-1.226749		1	-1.237671	0.146944	5.789696
	1	0.792323	-4.644458	-2.008888		1	-1.503508	1.762360	5.055729
	1	2.246411	-3.889592	-2.702111		1	0.126296	1.296511	5.594815
**TS4b**	6	-2.388431	-0.585372	-2.230593	**TS4b-n**	6	2.765014	1.581968	-1.413168
	6	-2.591951	0.662263	-1.618912		6	2.558693	2.250109	-0.194185
	6	-3.679813	1.446609	-1.968070		6	3.458240	3.209241	0.241459
	6	-4.576491	0.962987	-2.934546		6	4.577131	3.494167	-0.556795
	6	-4.386162	-0.287434	-3.529124		6	4.794072	2.817814	-1.760912
	6	-3.288328	-1.080288	-3.176197		6	3.891087	1.843263	-2.198148
	1	-3.846467	2.408695	-1.491801		1	3.311651	3.715222	1.191063
	1	-5.439367	1.562277	-3.211184		1	5.295965	4.237248	-0.223230
	1	-5.098804	-0.650583	-4.263584		1	5.673572	3.043523	-2.356544
	1	-3.131124	-2.049515	-3.641739		1	4.049461	1.320462	-3.137669
	6	-1.136685	-1.231499	-1.814167		6	1.674049	0.668343	-1.757958
	6	-0.141431	0.980898	-1.353575		6	0.094970	2.039329	-0.439228
	6	-1.500165	0.884330	-0.597035		6	1.312372	1.700754	0.460988
	1	-1.064413	-2.310063	-1.942600		1	1.890021	-0.129573	-2.466726
	1	-0.240615	1.641767	-2.222549		1	0.121801	3.090448	-0.743449
	1	-1.672231	1.737017	0.059091		1	1.179329	2.028918	1.490630
	7	-1.368303	-0.404755	0.146401		7	1.413622	0.209875	0.339671
	6	-2.448568	-0.769195	0.942130		44	-0.412765	-0.808004	-0.695777
	8	-2.686376	-1.907927	1.324823		6	-1.093399	-2.907379	0.141812
	8	-3.223323	0.301867	1.234043		6	0.293738	-2.715003	0.310833
	6	-4.465718	0.171902	2.022120		6	0.929824	-2.723897	-1.003814
	6	-5.460359	-0.733005	1.288302		6	-0.076413	-2.837685	-1.960754
	1	-5.100250	-1.761033	1.245975		6	-1.338460	-2.910269	-1.253645
	1	-6.421335	-0.718007	1.812725		1	-1.839708	-2.957498	0.920989
	1	-5.622311	-0.370710	0.268493		1	0.813811	-2.658523	1.253830
	6	-4.143772	-0.331828	3.432372		1	1.989522	-2.630488	-1.176951
	1	-3.778111	-1.358397	3.409726		1	0.038532	-2.842362	-3.035862
	1	-3.387567	0.306102	3.900545		1	-2.305418	-3.024495	-1.719448
	1	-5.048289	-0.293634	4.048069		53	-2.631133	-0.203826	-2.458404
	6	-4.975954	1.614750	2.067150		6	0.287802	1.061443	-1.603108
	1	-5.167711	1.988109	1.057117		1	-0.251743	1.224551	-2.531746
	1	-5.908216	1.664460	2.637449		6	-1.133422	1.748996	0.402169
	1	-4.241866	2.269562	2.545461		6	-1.477404	0.452936	0.557641
	44	0.754135	-1.338086	0.169257		6	-2.394337	-0.119540	1.565600
	6	1.438126	-2.381696	2.161597		6	-3.700559	-0.538258	1.259856
	6	0.039011	-2.462262	2.002627		6	-1.923469	-0.297987	2.880353
	6	-0.255382	-3.346204	0.878644		6	-4.509900	-1.108887	2.242105
	6	0.959286	-3.731938	0.313356		1	-4.074821	-0.408475	0.250253
	6	2.012658	-3.109465	1.088755		6	-2.734276	-0.874497	3.857816
	1	1.964929	-1.796481	2.900456		1	-0.914447	0.019952	3.128565
	1	-0.702711	-2.018219	2.647615		6	-4.032458	-1.283194	3.543876
	1	-1.251192	-3.610681	0.560794		1	-5.520680	-1.417742	1.988181
	1	1.109944	-4.361750	-0.552579		1	-2.351122	-1.001138	4.867109
	1	3.069962	-3.239114	0.915219		1	-4.665123	-1.732119	4.304591
	53	3.270927	-1.303755	-1.249278		6	-1.663852	2.918370	1.142902

Species	Cartesian coordinates				Species	Cartesian coordinates			
	6	0.101723	-0.483777	-1.743202		8	-1.097825	4.002536	1.183333
	1	0.840483	-0.717961	-2.503930		8	-2.843618	2.691518	1.756569
	6	0.854571	1.523790	-0.342767		6	-3.374330	3.796947	2.500732
	6	1.324513	0.618539	0.538213		1	-4.311340	3.438976	2.927213
	6	1.028614	2.999340	-0.278908		1	-3.554113	4.653784	1.846074
	6	1.328666	3.734326	-1.439933		1	-2.684433	4.099331	3.293221
	6	0.877653	3.702103	0.929968		6	2.423913	-0.289992	1.155000
	6	1.492538	5.117989	-1.390337		8	2.875946	0.335100	2.106983
	1	1.461296	3.213518	-2.384444		8	2.887909	-1.496008	0.756331
	6	1.038212	5.086933	0.978753		6	4.030936	-2.129919	1.452617
	1	0.620126	3.157138	1.833743		6	3.669956	-2.431736	2.910758
	6	1.346898	5.801202	-0.180588		1	3.502363	-1.513745	3.473276
	1	1.737609	5.663272	-2.297852		1	4.488527	-2.987952	3.378819
	1	0.912312	5.609276	1.923449		1	2.770358	-3.053802	2.965413
	1	1.469399	6.880068	-0.143187		6	5.273228	-1.240779	1.331509
	6	2.196872	0.953870	1.690936		1	5.152328	-0.313606	1.890833
	8	1.964031	0.659611	2.855164		1	5.465017	-0.998366	0.281507
	8	3.314246	1.616587	1.323338		1	6.143713	-1.778498	1.720760
	6	4.194096	1.992953	2.393335		6	4.228687	-3.429723	0.667633
	1	5.055077	2.459170	1.914498		1	3.333585	-4.056914	0.710641
	1	3.702085	2.701516	3.065528		1	5.062679	-3.995649	1.092641
	1	4.502399	1.116247	2.968370		1	4.459176	-3.220398	-0.381462
**TS5b**	6	0.654665	3.050387	-1.952808	**TS5b-n**	6	-2.438112	2.148075	1.945798
	6	1.379903	2.124073	-1.175776		6	-1.379390	2.226604	1.019007
	6	2.445829	2.579814	-0.399228		6	-1.405803	3.228708	0.048566
	6	2.800916	3.930778	-0.387263		6	-2.455072	4.149689	-0.007948
	6	2.091791	4.846958	-1.165414		6	-3.495059	4.082719	0.919813
	6	1.024158	4.403299	-1.942639		6	-3.482322	3.082321	1.889526
	1	3.007198	1.872540	0.200586		1	-0.602397	3.292200	-0.675389
	1	3.633962	4.263789	0.225868		1	-2.454936	4.919863	-0.774626
	1	2.365244	5.898428	-1.163214		1	-4.311583	4.798703	0.884385
	1	0.449728	5.110622	-2.536310		1	-4.298280	3.003527	2.604338
	6	-0.523161	2.608847	-2.692349		6	-2.471423	1.049731	2.903782
	6	-1.001610	1.359882	-2.620982		6	-1.589374	0.041054	2.898634
	6	-0.287407	0.247944	-1.909118		6	-0.371934	0.008663	2.013685
	6	1.088923	0.624449	-1.272479		6	-0.156906	1.314062	1.163460
	1	-1.055405	3.356461	-3.276587		1	-3.291037	1.037296	3.619573
	1	-1.912989	1.092704	-3.147603		1	-1.692134	-0.776690	3.599383
	1	-0.068639	-0.498912	-2.692633		1	0.482257	-0.034571	2.709697
	1	1.851558	0.228675	-1.951212		1	0.570342	1.912026	1.723125
	7	1.230127	-0.079328	-0.000009		7	0.457142	0.962076	-0.118342
	6	2.346036	-0.804992	0.292447		6	1.513224	1.652161	-0.613936
	8	2.461890	-1.545064	1.271931		8	2.150068	1.328646	-1.625367
	8	3.351303	-0.608582	-0.615485		8	1.812835	2.765139	0.124139
	6	4.635111	-1.311244	-0.508502		6	2.884875	3.689392	-0.259447
	6	4.422043	-2.826137	-0.608992		6	4.242735	2.977851	-0.238501
	1	3.870234	-3.198115	0.254233		1	4.303746	2.234098	-1.032667
	1	3.863868	-3.067118	-1.519758		1	4.395455	2.482010	0.725555
	1	5.391151	-3.333357	-0.659455		1	5.044923	3.710905	-0.374289
	6	5.355664	-0.912391	0.784426		6	2.583175	4.315664	-1.625724
	1	4.798845	-1.249225	1.658593		1	2.586789	3.557422	-2.408956
	1	6.354602	-1.360607	0.803127		1	3.339218	5.072321	-1.860205
	1	5.472061	0.175035	0.833585		1	1.605534	4.807893	-1.610696
	6	5.399453	-0.793355	-1.730976		6	2.816871	4.749514	0.844599
	1	5.501685	0.294724	-1.687896		1	1.827499	5.214939	0.868713
	1	6.399597	-1.235839	-1.765900		1	3.564850	5.528284	0.667370
	1	4.873626	-1.052812	-2.654756		1	3.009402	4.300831	1.823780
	44	-0.567244	-0.292368	1.275931		44	-0.149973	-0.927843	-1.046448
	6	-1.563947	-1.737134	2.718678		6	0.521965	-2.759004	-2.233583
	6	-0.137627	-1.904655	2.797006		6	1.350688	-1.641900	-2.609975
	6	0.421313	-0.706524	3.339827		6	0.535037	-0.687641	-3.284746
	6	-0.630023	0.209901	3.542436		6	-0.788494	-1.168865	-3.273022
	6	-1.870756	-0.418670	3.136323		6	-0.804806	-2.458186	-2.607267
	1	-2.261403	-2.477897	2.353472		1	0.851233	-3.649999	-1.718099
	1	0.421939	-2.789820	2.536883		1	2.420283	-1.579035	-2.490910
	1	1.475906	-0.524857	3.484104		1	0.871198	0.273320	-3.644603
	1	-0.532676	1.223108	3.904474		1	-1.655159	-0.660520	-3.669444
	1	-2.851252	0.032299	3.186662		1	-1.676360	-3.077073	-2.453108
	53	-1.476438	2.383432	1.085585		53	-2.940905	-0.474959	-0.771697
	6	-1.171587	-0.599135	-0.944249		6	-0.155445	-1.277079	1.134893

Species	Cartesian coordinates				Species	Cartesian coordinates			
	6	-0.585161	-1.573385	-0.155065		6	1.066782	-1.278010	0.425017
	6	-2.556355	-0.942508	-1.443835		6	2.430003	-1.519282	0.789166
	6	-2.705766	-1.806137	-2.541664		6	2.703032	-2.154256	2.028493
	6	-3.696918	-0.410391	-0.830674		6	3.512584	-1.109831	-0.025485
	6	-3.976972	-2.124396	-3.020474		6	4.012039	-2.421379	2.406499
	1	-1.827076	-2.232705	-3.019419		1	1.881582	-2.440720	2.677486
	6	-4.966828	-0.728576	-1.315894		6	4.819679	-1.353010	0.380113
	1	-3.576710	0.263519	0.011356		1	3.304767	-0.540744	-0.923892
	6	-5.110843	-1.585138	-2.408827		6	5.071109	-2.022744	1.582486
	1	-4.080240	-2.796129	-3.868068		1	4.212319	-2.925203	3.347125
	1	-5.844489	-0.304460	-0.836263		1	5.647161	-1.019868	-0.238891
	1	-6.100634	-1.834023	-2.780852		1	6.094986	-2.222094	1.886336
	6	-0.473752	-3.052018	-0.239331		6	-0.604721	-2.596243	1.683835
	8	-1.179903	-3.831523	0.365912		8	-1.033645	-2.785065	2.806769
	8	0.501713	-3.388480	-1.092031		8	-0.428138	-3.608879	0.802262
	6	0.760139	-4.806087	-1.218412		6	-0.809959	-4.910319	1.282779
	1	1.608706	-4.884540	-1.895689		1	-0.227082	-5.183729	2.166046
	1	1.002419	-5.233375	-0.243265		1	-0.605002	-5.596072	0.461059
	1	-0.115177	-5.312116	-1.630756		1	-1.871664	-4.925440	1.538269
**TS6b**	6	2.282929	-2.426765	0.961592	**TS6b-n**	6	-2.973399	-1.753566	-0.490055
	6	2.581925	-1.200837	1.586635		6	-2.689076	-2.062088	0.854164
	6	3.554631	-1.110294	2.566962		6	-3.452766	-2.974337	1.561078
	6	4.254947	-2.281768	2.909817		6	-4.541114	-3.573239	0.900623
	6	3.960087	-3.496852	2.291847		6	-4.823592	-3.270303	-0.431391
	6	2.955232	-3.585480	1.310561		6	-4.029680	-2.358081	-1.150608
	1	3.785782	-0.166023	3.053116		1	-3.233897	-3.217046	2.597496
	1	5.038209	-2.237207	3.661393		1	-5.170803	-4.277805	1.436616
	1	4.517774	-4.387624	2.567632		1	-5.671476	-3.742332	-0.920135
	1	2.731339	-4.536293	0.834424		1	-4.253856	-2.130557	-2.189401
	6	1.130935	-2.115069	-0.010350		6	-1.881920	-0.759911	-0.918044
	6	-0.061478	-1.836189	0.933103		6	-0.600590	-1.627428	-0.958699
	6	0.236127	-0.536602	1.541604		6	-0.294703	-1.906035	0.428735
	6	1.626724	-0.174104	0.952368		6	-1.449026	-1.226408	1.211964
	1	0.969994	-2.783067	-0.851198		1	-2.075644	-0.110825	-1.765214
	1	-0.312728	-2.659787	1.595376		1	-0.603210	-2.437162	-1.682854
	1	0.125320	-0.425568	2.613894		1	0.019903	-2.892444	0.749079
	1	1.930485	0.866394	0.976968		1	-1.286721	-0.991106	2.260123
	7	1.478417	-0.735213	-0.401761		7	-1.652442	-0.044494	0.360108
	6	2.373907	-0.505165	-1.427039		6	-2.401812	1.012870	0.836455
	8	2.616870	-1.315372	-2.305475		8	-2.474954	1.286929	2.026214
	8	2.882826	0.743671	-1.323567		8	-2.978005	1.683786	-0.177423
	6	3.803206	1.274878	-2.342841		6	-3.741371	2.922482	0.063167
	6	3.104033	1.327892	-3.704896		6	-4.164847	3.330796	-1.349887
	1	2.880426	0.325084	-4.069113		1	-4.753715	4.252168	-1.312330
	1	2.168931	1.891291	-3.629643		1	-4.774802	2.548457	-1.811150
	1	3.751526	1.832813	-4.429410		1	-3.287989	3.502404	-1.980417
	6	4.091541	2.686572	-1.825013		6	-2.833589	3.985810	0.688822
	1	4.549043	2.648562	-0.831851		1	-1.945306	4.138427	0.068453
	1	4.778065	3.202291	-2.503019		1	-2.518730	3.692068	1.690439
	1	3.167522	3.268440	-1.758446		1	-3.374604	4.935680	0.752380
	6	5.086339	0.437780	-2.377292		6	-4.967376	2.616598	0.930183
	1	5.812811	0.907186	-3.048656		1	-4.670824	2.306567	1.932406
	1	5.531490	0.383255	-1.378651		1	-5.565353	1.820714	0.474995
	1	4.883441	-0.573814	-2.729158		1	-5.593811	3.511337	1.006712
	44	-1.893723	-0.815989	0.494096		44	1.478157	-0.970430	-0.898000
	6	-0.733677	1.216845	1.018836		6	3.209787	-1.003502	-2.425725
	6	-1.610011	1.212247	0.071996		6	2.256823	-2.034603	-2.751750
	6	-4.033744	-1.375143	0.056687		6	2.246820	-2.955325	-1.669195
	6	-3.335127	-2.524862	0.580354		6	3.172074	-2.484450	-0.676117
	6	-2.985869	-2.226937	1.929737		6	3.797014	-1.302643	-1.171211
	6	-3.446630	-0.907181	2.232081		1	3.441615	-0.150172	-3.047261
	6	-4.115870	-0.397903	1.082290		1	1.686772	-2.102663	-3.667702
	1	-4.431925	-1.280640	-0.943154		1	1.653882	-3.858118	-1.598485
	1	-3.157103	-3.452266	0.054475		1	3.370384	-2.964938	0.273145
	1	-2.466789	-2.885602	2.613813		1	4.545374	-0.713453	-0.659023
	1	-3.328189	-0.394016	3.177818		53	0.626333	1.295989	-2.312747
	1	-4.535700	0.591523	0.966194		6	1.167702	-0.879752	1.334489
	53	-1.459784	-1.123198	-2.264780		6	1.616344	0.244839	0.850639
	6	-0.090750	2.203182	1.889836		6	2.164294	1.510838	1.261467
	6	0.556417	3.307940	1.303347		6	1.785221	2.066932	2.504039

Species	Cartesian coordinates				Species	Cartesian coordinates			
	6	-0.151991	2.114850	3.291465		6	3.082055	2.209885	0.452470
	6	1.122370	4.297303	2.106126		6	2.329560	3.275446	2.925598
	1	0.601209	3.378001	0.221838		1	1.062715	1.541839	3.117854
	6	0.416358	3.108798	4.086932		6	3.637537	3.407932	0.891619
	1	-0.667377	1.280845	3.758712		1	3.336486	1.808261	-0.521396
	6	1.057980	4.200845	3.498223		6	3.262117	3.944730	2.126409
	1	1.619159	5.144204	1.641158		1	2.026693	3.698391	3.879245
	1	0.352998	3.031861	5.168669		1	4.350208	3.934106	0.263166
	1	1.503371	4.971793	4.120270		1	3.686042	4.887760	2.460293
	6	-2.397714	2.199308	-0.674946		6	1.470609	-1.702735	2.504653
	8	-3.614025	2.234071	-0.716165		8	1.549992	-2.922741	2.512146
	8	-1.603119	3.081722	-1.313534		8	1.672529	-0.941855	3.603517
	6	-2.293643	4.069517	-2.102957		6	2.042600	-1.661112	4.793689
	1	-1.511421	4.663904	-2.573944		1	1.263912	-2.376508	5.068739
	1	-2.915345	3.583718	-2.858296		1	2.982104	-2.198585	4.642698
	1	-2.924418	4.696678	-1.468200		1	2.156519	-0.902305	5.567232
**TS7b**	6	0.911091	-2.710233	0.765692	**TS7b-n**	6	1.443800	-2.669642	-0.209790
	6	1.776443	-2.115318	1.704799		6	2.225902	-2.246469	-1.301721
	6	2.292197	-2.849617	2.761740		6	2.860051	-3.167501	-2.121791
	6	1.944057	-4.207257	2.854526		6	2.718586	-4.530920	-1.816075
	6	1.082077	-4.794081	1.924374		6	1.939237	-4.948588	-0.733777
	6	0.537938	-4.042902	0.870933		6	1.274078	-4.016945	0.078246
	1	2.958458	-2.399814	3.493132		1	3.461207	-2.850668	-2.969838
	1	2.355426	-4.811268	3.658466		1	3.226397	-5.271781	-2.427392
	1	0.834036	-5.847902	2.013920		1	1.851044	-6.008922	-0.514402
	1	-0.137245	-4.498441	0.152220		1	0.662176	-4.343198	0.914434
	6	0.518429	-1.585866	-0.205594		6	0.869941	-1.387648	0.409008
	6	-0.436622	-0.761104	0.683935		6	-0.184486	-0.988710	-0.643946
	6	0.521601	0.020636	1.616685		6	0.656442	-0.398116	-1.806816
	6	1.894354	-0.651103	1.288258		6	2.115552	-0.725844	-1.327277
	1	0.173525	-1.854179	-1.198344		1	0.558759	-1.413954	1.447785
	1	-1.227037	-1.451072	1.157852		1	-0.864805	-1.869829	-0.878128
	1	0.277471	-0.016695	2.682797		1	0.453287	-0.806215	-2.801038
	1	2.777556	-0.098279	1.596833		1	2.898047	-0.154578	-1.815715
	7	1.736196	-0.757783	-0.179371		7	1.947082	-0.428983	0.112431
	6	-0.728660	1.264613	0.102282		6	2.938271	-0.154641	1.024367
	6	0.346462	1.352620	0.921249		8	2.795113	-0.294512	2.230457
	6	2.761224	-0.976878	-1.076502		8	4.029360	0.332912	0.395943
	8	2.606736	-1.520293	-2.157488		6	5.193423	0.820405	1.156968
	8	3.911030	-0.457242	-0.593228		6	6.140192	1.288067	0.048731
	6	5.133819	-0.417987	-1.414093		1	7.061766	1.684221	0.485382
	6	6.131038	0.281213	-0.486491		1	5.673373	2.074309	-0.551614
	1	7.100118	0.381053	-0.984330		1	6.399863	0.457058	-0.613570
	1	5.772835	1.279361	-0.218215		6	4.780390	1.993925	2.050972
	1	6.272592	-0.294510	0.433073		1	4.089517	1.669008	2.829326
	6	4.887990	0.416200	-2.675162		1	4.300669	2.776046	1.454054
	1	4.163113	-0.067405	-3.330493		1	5.668901	2.424175	2.524349
	1	4.514456	1.408885	-2.404822		6	5.815501	-0.329927	1.955135
	1	5.828837	0.542270	-3.220662		1	6.054667	-1.166615	1.291178
	6	5.594675	-1.843046	-1.736166		1	5.135818	-0.678917	2.732751
	1	5.713292	-2.421277	-0.814307		1	6.745343	0.009430	2.423084
	1	4.876480	-2.349713	-2.381039		44	-2.263080	-0.513779	-0.547308
	1	6.564343	-1.809128	-2.243500		6	-3.957744	0.857911	-0.869339
	44	-2.426636	-0.083097	0.508987		6	-4.521505	-0.441656	-0.638543
	6	-3.180896	1.761760	1.575802		6	-4.058210	-1.300203	-1.661183
	6	-3.979142	1.479553	0.406051		6	-3.251501	-0.532315	-2.584188
	6	-4.669014	0.242115	0.641140		6	-3.221669	0.798692	-2.110085
	6	-4.225147	-0.266525	1.882174		1	-4.120185	1.738871	-0.265259
	6	-3.306392	0.679823	2.475553		1	-5.123863	-0.728852	0.211450
	1	-2.556175	2.633515	1.703635		1	-4.253097	-2.362961	-1.731171
	1	-4.109838	2.128050	-0.447831		1	-2.787380	-0.905818	-3.486936
	1	-5.345637	-0.247459	-0.044641		1	-2.702742	1.620053	-2.582546
	1	-4.511804	-1.218420	2.311697		53	-2.398496	-1.370589	2.088164
	1	-2.831737	0.585593	3.442679		6	0.279606	1.037445	-1.572956
	53	-2.756314	-1.456923	-1.865446		6	-0.711010	1.099913	-0.643006
	6	1.338780	2.442063	1.021754		6	-1.066616	2.219308	0.255150
	6	1.853625	2.793527	2.283612		6	-1.828283	3.323252	-0.169939
	6	1.815132	3.129130	-0.109784		6	-0.603404	2.191001	1.583147
	6	2.788803	3.819019	2.414608		6	-2.122522	4.363190	0.710625
	1	1.505474	2.268392	3.169413		1	-2.162805	3.384725	-1.199926

Species	Cartesian coordinates				Species	Cartesian coordinates			
	6	2.751359	4.153907	0.024178		6	-0.899021	3.234868	2.457607
	1	1.472736	2.838242	-1.097427		1	-0.011578	1.349201	1.926789
	6	3.239276	4.505987	1.284861		6	-1.664194	4.321737	2.029244
	1	3.166343	4.081527	3.399102		1	-2.706538	5.210737	0.361991
	1	3.110284	4.670461	-0.861852		1	-0.530954	3.194252	3.478766
	1	3.970945	5.302621	1.385399		1	-1.898141	5.131037	2.715005
	6	-1.030471	2.214238	-1.007455		6	1.091227	2.118036	-2.166965
	8	-0.827396	2.003434	-2.184492		8	2.009069	1.902529	-2.946111
	8	-1.558328	3.376528	-0.539990		8	0.717050	3.362357	-1.805399
	6	-1.804329	4.383497	-1.541269		6	1.501163	4.430441	-2.359620
	1	-2.466602	3.999338	-2.320443		1	2.548744	4.334219	-2.063387
	1	-2.268993	5.215399	-1.012315		1	1.072427	5.346824	-1.954593
	1	-0.864519	4.701217	-1.999724		1	1.442400	4.427842	-3.451170
**TS8b**	6	2.354040	1.724866	1.779337	**TS8b-n**	6	-2.427978	2.403346	1.189058
	6	2.083520	2.200729	0.476450		6	-2.156551	2.663858	-0.174393
	6	2.866560	3.201688	-0.073859		6	-2.918068	3.588547	-0.869278
	6	3.935564	3.718808	0.674843		6	-3.960527	4.248080	-0.202459
	6	4.228241	3.225980	1.950985		6	-4.254662	3.969527	1.137881
	6	3.446782	2.212301	2.508874		6	-3.501647	3.029592	1.840432
	1	2.672290	3.562838	-1.079421		1	-2.729514	3.776601	-1.921726
	1	4.559701	4.498093	0.246458		1	-4.568542	4.967401	-0.743876
	1	5.070752	3.626934	2.506057		1	-5.078552	4.479165	1.628000
	1	3.662073	1.829804	3.502999		1	-3.717482	2.811834	2.883087
	6	1.410429	0.736832	2.271903		6	-1.515996	1.469305	1.814465
	6	-0.292863	1.731816	0.815187		6	0.205376	2.183259	0.212230
	6	0.931234	1.416817	-0.116687		6	-1.029434	1.768788	-0.646381
	1	1.755899	0.075585	3.065024		1	-1.867816	0.944562	2.702096
	1	-0.246967	2.797024	1.082582		1	0.205329	3.276610	0.310133
	1	0.739133	1.632728	-1.170247		1	-0.854377	1.807394	-1.723558
	7	1.219801	-0.024706	0.128039		7	-1.355420	0.385551	-0.183692
	6	2.328906	-0.640167	-0.408511		44	0.266621	-0.622737	0.850516
	8	2.728934	-1.742416	-0.053242		6	1.341844	-2.385844	1.718285
	8	2.920950	0.136055	-1.350632		6	-0.022699	-2.847515	1.562759
	6	4.157652	-0.289471	-2.029354		6	-0.846511	-2.114832	2.438924
	6	5.289178	-0.440578	-1.006789		6	-0.024910	-1.156961	3.098458
	1	5.086889	-1.263881	-0.321306		6	1.338514	-1.351577	2.687301
	1	6.231186	-0.638895	-1.528576		1	2.209144	-2.786893	1.214721
	1	5.406603	0.482623	-0.430323		1	-0.345358	-3.621742	0.881596
	6	3.910216	-1.578018	-2.819923		1	-1.920100	-2.200295	2.522086
	1	3.688391	-2.409897	-2.151890		1	-0.354930	-0.444181	3.843625
	1	3.068733	-1.446154	-3.506480		1	2.202865	-0.817466	3.056057
	1	4.800533	-1.820386	-3.409880		53	0.489462	-1.808129	-1.742533
	6	4.436499	0.880765	-2.976726		6	-0.096982	1.473580	1.528347
	1	4.579426	1.808171	-2.414531		1	0.560249	1.656853	2.380301
	1	5.342259	0.684713	-3.558419		6	1.576303	1.734776	-0.230153
	1	3.602711	1.020598	-3.670812		6	1.932070	0.481486	0.146842
	44	-0.386300	-1.150680	1.042762		6	3.325053	0.000981	0.289600
	6	-0.004679	0.827429	2.009066		6	4.151592	0.687232	1.205287
	1	-0.662028	0.881817	2.877478		6	3.864578	-1.117091	-0.370058
	6	-1.482462	-2.991137	1.663477		6	5.460856	0.275314	1.447912
	6	-0.121281	-3.444224	1.440313		1	3.752031	1.553418	1.725159
	6	0.714829	-2.845411	2.401375		6	5.180120	-1.517984	-0.136478
	6	-0.097924	-1.981902	3.191417		1	3.250765	-1.657428	-1.080621
	6	-1.466300	-2.100847	2.764033		6	5.984440	-0.830585	0.775218
	1	-2.358658	-3.298461	1.112985		1	6.073187	0.822994	2.159644
	1	0.194754	-4.119154	0.657992		1	5.577769	-2.375617	-0.672885
	1	1.789370	-2.947854	2.456264		1	7.005559	-1.152832	0.959432
	1	0.243446	-1.379090	4.023897		6	2.389222	2.738931	-0.951908
	1	-2.328132	-1.607544	3.188112		8	2.049615	3.906115	-1.100515
	53	-0.728841	-2.071480	-1.649064		8	3.529968	2.246297	-1.481687
	6	-1.665226	1.385452	0.272980		6	4.335993	3.190666	-2.197780
	6	-1.975096	0.078865	0.399227		1	4.650333	4.010095	-1.545412
	6	-3.336611	-0.488228	0.217745		1	3.784049	3.608800	-3.043786
	6	-2.499721	2.502958	-0.260045		1	5.202407	2.629433	-2.547893
	6	-2.847315	3.584432	0.568924		6	-2.497733	-0.159277	-0.724352
	6	-2.900178	2.539674	-1.605495		8	-3.147831	0.385328	-1.611681
	6	-3.586215	4.659537	0.075250		8	-2.832653	-1.322724	-0.118995
	1	-2.549047	3.574389	1.614647		6	-4.009967	-2.086155	-0.567988
	6	-3.631954	3.619614	-2.101824		6	-3.863562	-2.488147	-2.039459
	1	-2.634798	1.713717	-2.255842		1	-3.900746	-1.615653	-2.691256

Species	Cartesian coordinates				Species	Cartesian coordinates			
	6	-3.979989	4.681500	-1.264722		1	-4.676810	-3.169058	-2.312215
	1	-3.854999	5.478867	0.736799		1	-2.911852	-3.003344	-2.197381
	1	-3.929287	3.630966	-3.147163		6	-5.286237	-1.276684	-0.309434
	1	-4.551869	5.520025	-1.652719		1	-5.311955	-0.381207	-0.930272
	8	-3.903106	-1.182272	1.050512		1	-5.343171	-0.980660	0.743357
	8	-3.917756	-0.160537	-0.953796		1	-6.163641	-1.891048	-0.536795
	6	-5.242100	-0.679394	-1.156703		6	-3.972697	-3.327149	0.328322
	1	-5.921744	-0.324162	-0.378026		1	-3.043270	-3.883972	0.178850
	1	-5.553915	-0.310696	-2.133797		1	-4.811517	-3.987255	0.088483
	1	-5.230105	-1.772165	-1.146484		1	-4.050521	-3.049015	1.384286
**TS9b**	6	-0.119420	3.985080	0.277895	**TS9b-n**	6	2.233969	3.087005	1.068936
	6	-0.863373	2.822342	-0.019709		6	2.040309	1.693855	1.167821
	6	-1.758959	2.852357	-1.090720		6	3.124292	0.837682	0.955107
	6	-1.919761	4.006659	-1.864638		6	4.387293	1.341775	0.636608
	6	-1.185633	5.156049	-1.568042		6	4.581217	2.721836	0.534349
	6	-0.290616	5.140880	-0.500000		6	3.508734	3.584561	0.750349
	1	-2.335261	1.966020	-1.333108		1	2.973954	-0.234417	1.029937
	1	-2.624853	4.006696	-2.691313		1	5.215055	0.657864	0.472504
	1	-1.310967	6.056871	-2.161924		1	5.561149	3.121985	0.289622
	1	0.290061	6.028975	-0.261635		1	3.648524	4.659946	0.667885
	6	0.838106	3.959231	1.383129		6	1.102791	3.998988	1.250831
	6	1.179276	2.826084	2.014931		6	-0.145435	3.566421	1.475832
	6	0.544441	1.497862	1.703680		6	-0.502581	2.113898	1.673513
	6	-0.782390	1.610215	0.915488		6	0.696099	1.105444	1.589748
	1	1.301376	4.901449	1.668822		1	1.303103	5.065149	1.164719
	1	1.917972	2.847145	2.810605		1	-0.966203	4.269303	1.587213
	1	0.281692	1.057447	2.677755		1	-0.901607	2.072004	2.698671
	1	-1.573353	1.762875	1.657591		1	0.848087	0.730956	2.602609
	7	-1.030831	0.334652	0.247992		7	0.310999	-0.064241	0.776207
	6	-2.314610	-0.146313	0.131989		6	0.189424	-1.311679	1.379873
	8	-2.676726	-0.988287	-0.675457		8	-0.401983	-2.250142	0.872655
	8	-3.160662	0.453076	1.028531		8	0.812774	-1.358300	2.586833
	6	-4.599560	0.164808	1.028564		6	0.797226	-2.580506	3.413749
	6	-4.843394	-1.304909	1.387506		6	-0.642670	-2.945813	3.788900
	1	-4.428132	-1.963785	0.625015		1	-1.212859	-3.246083	2.909797
	1	-4.379709	-1.537908	2.351770		1	-1.139499	-2.094448	4.266033
	1	-5.918912	-1.491542	1.475507		1	-0.633834	-3.774870	4.503936
	6	-5.224855	0.541341	-0.319845		6	1.515600	-3.717612	2.681574
	1	-4.857710	-0.106370	-1.115887		1	0.974956	-4.008213	1.781090
	1	-6.313953	0.445966	-0.259241		1	1.595647	-4.584791	3.345479
	1	-4.989949	1.581682	-0.567924		1	2.527069	-3.409368	2.400124
	6	-5.123088	1.084807	2.136203		6	1.585887	-2.147900	4.652739
	1	-4.922978	2.133115	1.895073		1	2.596501	-1.833933	4.376370
	1	-6.203339	0.956528	2.254161		1	1.662640	-2.981553	5.357058
	1	-4.640856	0.852246	3.090400		1	1.090180	-1.313474	5.158018
	44	0.697338	-0.404290	-0.910021		44	0.170064	0.147886	-1.371797
	6	2.009897	-0.846643	-2.719474		6	-0.214872	0.918341	-3.503532
	6	0.729318	-0.406901	-3.241728		6	1.176810	0.807509	-3.285069
	6	0.535092	0.930707	-2.853056		6	1.533829	1.659075	-2.177100
	6	1.646769	1.329920	-2.048988		6	0.346955	2.338200	-1.755127
	6	2.582077	0.232855	-2.014153		6	-0.735051	1.849259	-2.546382
	1	2.445026	-1.824970	-2.862995		1	-0.783760	0.368827	-4.241745
	1	0.040984	-1.012749	-3.813061		1	1.851049	0.157697	-3.823139
	1	-0.342226	1.529524	-3.049591		1	2.527800	1.799052	-1.776761
	1	1.791903	2.310664	-1.619462		1	0.276037	3.073313	-0.968109
	1	3.534534	0.229238	-1.504273		1	-1.770514	2.145959	-2.445461
	53	-0.123819	-3.107022	-1.242207		53	1.619815	-2.197980	-1.816907
	6	1.453223	0.387960	1.059943		6	-1.665863	1.602306	0.832177
	6	0.777847	-0.848293	0.967066		6	-1.496419	0.550021	-0.008874
	6	2.941520	0.440649	1.236768		6	-2.491985	-0.475484	-0.363428
	6	3.656952	-0.677043	1.705498		6	-3.126246	-1.178000	0.686191
	6	3.666180	1.604754	0.912409		6	-2.846583	-0.797916	-1.688400
	6	5.042508	-0.622887	1.859154		6	-4.081008	-2.149382	0.419624
	1	3.127307	-1.589512	1.955391		1	-2.857811	-0.945554	1.710539
	6	5.051101	1.651561	1.063642		6	-3.801584	-1.779843	-1.951894
	1	3.145578	2.473643	0.530060		1	-2.407182	-0.258241	-2.515478
	6	5.747094	0.538974	1.541720		6	-4.417448	-2.460005	-0.902748
	1	5.570428	-1.497695	2.228250		1	-4.562027	-2.671709	1.241507
	1	5.586671	2.559675	0.800905		1	-4.065866	-2.007104	-2.980348
	1	6.826302	0.576866	1.659467		1	-5.159075	-3.226105	-1.110397

Species	Cartesian coordinates				Species	Cartesian coordinates			
	6	0.379278	-1.906462	1.919990		6	-2.974862	2.193774	1.158146
	8	1.232392	-2.628691	2.404674		8	-3.170066	2.915059	2.128254
	8	-0.918608	-1.955156	2.195942		8	-3.944155	1.923972	0.249499
	6	-1.325528	-3.048946	3.048900		6	-5.240103	2.458129	0.558298
	1	-2.398079	-2.921169	3.182624		1	-5.612015	2.055122	1.503988
	1	-1.108736	-3.999621	2.557851		1	-5.886176	2.151992	-0.264561
	1	-0.803561	-3.000682	4.006862		1	-5.202238	3.548011	0.631796
**INT1b**	6	2.114106	-2.001567	1.777208	**INT1b-n**	6	-2.747461	-1.846371	0.568419
	6	2.595130	-0.727999	2.127979		6	-2.732445	-1.326447	1.874521
	6	3.305491	-0.532435	3.302423		6	-3.428270	-1.945819	2.901110
	6	3.550202	-1.648587	4.119310		6	-4.172763	-3.096333	2.593423
	6	3.068388	-2.912918	3.772018		6	-4.187736	-3.614456	1.296382
	6	2.328520	-3.102126	2.594057		6	-3.459383	-2.998313	0.265980
	1	3.674135	0.450008	3.585071		1	-3.411221	-1.551479	3.913520
	1	4.124764	-1.527148	5.033356		1	-4.747228	-3.587661	3.373863
	1	3.273445	-3.761536	4.418438		1	-4.773659	-4.503799	1.080825
	1	1.952860	-4.087197	2.330008		1	-3.468348	-3.412925	-0.738927
	6	1.370072	-1.822476	0.450666		6	-1.826183	-0.941845	-0.250599
	6	0.051822	-1.087292	0.748241		6	-0.373304	-1.286643	0.208404
	6	0.520787	0.286709	1.282942		6	-0.345991	-0.696912	1.656398
	6	2.076670	0.210398	1.045071		6	-1.803156	-0.122039	1.822775
	1	1.321555	-2.700709	-0.184611		1	-2.027153	-0.908214	-1.316710
	1	-0.652246	-1.652159	1.411362		1	-0.252620	-2.368329	0.218310
	1	0.342403	0.360800	2.364385		1	-0.171126	-1.455113	2.425928
	1	2.583962	1.159846	0.896934		1	-1.924558	0.646133	2.580351
	7	2.106706	-0.682374	-0.138742		7	-2.029638	0.356414	0.440206
	6	3.138657	-0.870669	-1.023720		6	-3.007274	1.277150	0.158425
	8	3.213807	-1.855291	-1.751966		8	-3.467496	2.058810	0.977039
	8	3.977269	0.182117	-1.012247		8	-3.317710	1.240017	-1.165441
	6	5.094018	0.286730	-1.972621		6	-4.326651	2.151066	-1.735896
	6	5.723383	1.631167	-1.600353		6	-4.361521	1.730806	-3.207624
	1	6.581650	1.834238	-2.247526		1	-5.089533	2.338094	-3.753439
	1	4.999921	2.443013	-1.718255		1	-4.648100	0.679375	-3.302741
	1	6.067207	1.622242	-0.561982		1	-3.381286	1.866570	-3.674590
	6	6.083426	-0.860385	-1.745768		6	-3.862225	3.604389	-1.596178
	1	6.411832	-0.877739	-0.701864		1	-2.875329	3.735181	-2.052054
	1	5.631525	-1.821880	-1.990487		1	-3.810254	3.897685	-0.547605
	1	6.965440	-0.712821	-2.377247		1	-4.564087	4.265343	-2.115028
	6	4.550822	0.316589	-3.404301		6	-5.686205	1.907592	-1.072417
	1	4.084165	-0.633204	-3.666967		1	-5.666061	2.192117	-0.020370
	1	3.814484	1.118806	-3.515292		1	-5.961676	0.851058	-1.148611
	1	5.371494	0.510607	-4.102287		1	-6.453745	2.497231	-1.584024
	44	-1.626298	-1.127741	-0.484979		44	1.269552	-0.539569	-1.005989
	6	-2.366212	-1.018164	-2.549519		6	2.172390	0.496017	-3.077884
	6	-0.946304	-0.816800	-2.580678		6	1.207943	1.327637	-2.424516
	6	-0.332602	-2.044858	-2.214567		6	-0.038937	0.643841	-2.374887
	6	-1.359572	-3.025362	-2.018141		6	0.183739	-0.667427	-2.913101
	6	-2.606919	-2.402026	-2.213979		6	1.556629	-0.733172	-3.362354
	1	-3.104568	-0.268616	-2.792882		1	3.210683	0.746844	-3.250359
	1	-0.451408	0.108205	-2.835456		1	1.387457	2.331090	-2.066953
	1	0.735222	-2.213041	-2.162184		1	-0.976808	1.041812	-2.015911
	1	-1.201597	-4.056326	-1.727342		1	-0.560953	-1.438155	-3.059206
	1	-3.578585	-2.865607	-2.111864		1	2.027875	-1.596981	-3.811777
	53	-3.987292	-0.914325	0.822356		53	2.529040	-2.871486	-0.531480
	6	-0.252991	1.366277	0.573016		6	0.690869	0.377357	1.740713
	6	-1.304651	0.932669	-0.156643		6	1.313451	0.778609	0.603424
	6	0.156147	2.783682	0.760109		6	2.202506	1.945200	0.460861
	6	0.549594	3.261909	2.024173		6	3.573657	1.785412	0.182851
	6	0.198507	3.678744	-0.324768		6	1.678077	3.250320	0.543317
	6	0.942115	4.588261	2.200919		6	4.394023	2.897824	-0.003513
	1	0.527883	2.596639	2.882719		1	3.994700	0.784796	0.138958
	6	0.589562	5.005650	-0.147313		6	2.500596	4.358220	0.348847
	1	-0.066601	3.325384	-1.316435		1	0.621420	3.382875	0.758799
	6	0.962631	5.467872	1.116282		6	3.861072	4.186641	0.073527
	1	1.229506	4.935970	3.189660		1	5.452604	2.757342	-0.204026
	1	0.614492	5.676289	-1.002150		1	2.081045	5.358266	0.417000
	1	1.272070	6.500151	1.253681		1	4.500747	5.051987	-0.074800
	6	-2.224611	1.877812	-0.850050		6	0.958344	0.906280	3.116815
	8	-2.375058	1.950590	-2.062814		8	0.194113	0.724319	4.050518
	8	-2.912055	2.651906	0.011691		8	2.127120	1.558722	3.243741

Species	Cartesian coordinates				Species	Cartesian coordinates			
	6	-3.821783	3.591598	-0.583556		6	2.407212	2.076895	4.555130
	1	-4.321262	4.083765	0.250666		1	3.378221	2.564356	4.473950
	1	-3.276952	4.322578	-1.187142		1	1.640414	2.794587	4.857292
	1	-4.548301	3.076924	-1.216835		1	2.442407	1.266964	5.287913
**INT2b**	6	2.472674	-0.262043	2.355222	**INT2b-n**	6	-2.884407	1.344771	1.313760
	6	2.604040	0.978212	1.715383		6	-2.557669	2.248693	0.294052
	6	3.492715	1.938049	2.179777		6	-3.283245	3.417008	0.106879
	6	4.262339	1.625831	3.310745		6	-4.363542	3.661437	0.967585
	6	4.125880	0.392589	3.955155		6	-4.687249	2.763491	1.989774
	6	3.219352	-0.568793	3.484856		6	-3.942094	1.590732	2.179718
	1	3.593603	2.902538	1.689515		1	-3.024208	4.123853	-0.676157
	1	4.974670	2.352727	3.690726		1	-4.956620	4.562475	0.839079
	1	4.736291	0.173085	4.826518		1	-5.530266	2.974943	2.641533
	1	3.120061	-1.527818	3.986140		1	-4.196675	0.895806	2.975635
	6	1.415141	-1.022594	1.579693		6	-1.872750	0.222799	1.213522
	6	-0.000813	-0.441496	1.726906		6	-0.439737	0.636748	1.590114
	6	0.148984	1.048955	1.283605		6	-0.125463	1.847156	0.660140
	6	1.571148	0.989324	0.607643		6	-1.305729	1.710323	-0.365004
	1	1.480434	-2.105601	1.616185		1	-2.184722	-0.745593	1.592406
	1	-0.511985	-0.619866	2.667117		1	-0.219350	0.754407	2.645462
	1	0.177454	1.740786	2.131199		1	-0.184311	2.812012	1.170159
	1	1.720935	1.679728	-0.217428		1	-1.088387	2.037169	-1.377517
	7	1.562427	-0.455520	0.177142		7	-1.521213	0.215079	-0.267717
	6	2.640004	-0.926847	-0.626536		6	-2.410008	-0.310373	-1.256916
	8	3.127486	-2.031466	-0.483973		8	-2.317790	0.002637	-2.425335
	8	2.962053	-0.012688	-1.540662		8	-3.277820	-1.167701	-0.714472
	6	4.076350	-0.226957	-2.503438		6	-4.391073	-1.754662	-1.516226
	6	4.078636	1.082625	-3.292660		6	-5.136982	-2.589769	-0.475156
	1	4.862038	1.053758	-4.055347		1	-6.008609	-3.062275	-0.936777
	1	3.116552	1.237309	-3.788940		1	-5.480713	-1.961434	0.351069
	1	4.270101	1.933178	-2.632373		1	-4.495522	-3.378634	-0.071076
	6	5.385982	-0.410776	-1.733068		6	-3.826672	-2.635256	-2.632722
	1	5.544569	0.425444	-1.045442		1	-3.168364	-3.407113	-2.221975
	1	5.387502	-1.341689	-1.165121		1	-3.274732	-2.043838	-3.363845
	1	6.220289	-0.432067	-2.440943		1	-4.653104	-3.138561	-3.144187
	6	3.754512	-1.416157	-3.410847		6	-5.275351	-0.624468	-2.048136
	1	3.719583	-2.348642	-2.846398		1	-4.746723	-0.017012	-2.783428
	1	2.796906	-1.262833	-3.918600		1	-5.608469	0.017924	-1.227684
	1	4.529720	-1.503844	-4.178456		1	-6.160804	-1.055611	-2.525160
	44	-0.759085	-1.339741	-0.088767		44	0.495500	-0.967222	0.468476
	6	-1.341550	-2.535953	-2.042931		6	1.455429	-3.096819	0.608558
	6	0.052763	-2.574103	-1.808322		6	1.213331	-2.862003	-0.761879
	6	0.311118	-3.375515	-0.615947		6	-0.180786	-2.659991	-0.899034
	6	-0.922283	-3.725461	-0.072304		6	-0.825223	-2.945233	0.378869
	6	-1.952345	-3.165681	-0.934629		6	0.180538	-3.194968	1.306971
	1	-1.839205	-2.005508	-2.841229		1	2.423625	-3.256026	1.059803
	1	0.807994	-2.154615	-2.459445		1	1.958912	-2.740562	-1.533945
	1	1.292895	-3.615552	-0.234829		1	-0.688332	-2.430156	-1.827145
	1	-1.102121	-4.291275	0.831121		1	-1.889420	-2.926538	0.560469
	1	-3.015590	-3.276475	-0.781321		1	0.060509	-3.402954	2.361035
	53	-3.139391	-1.227702	1.432453		53	2.397099	-0.583730	2.540140
	6	-0.820938	1.519769	0.218141		6	1.154609	1.735532	-0.139184
	6	-1.340691	0.562494	-0.580488		6	1.606374	0.498501	-0.444257
	6	-0.940635	2.988611	0.009357		6	2.657348	0.141539	-1.420252
	6	-1.190569	3.849010	1.094038		6	3.954980	-0.231881	-1.031434
	6	-0.791923	3.558977	-1.267884		6	2.335226	0.123106	-2.790635
	6	-1.307759	5.225238	0.904815		6	4.901343	-0.602409	-1.986799
	1	-1.322348	3.434043	2.089443		1	4.213009	-0.226678	0.022174
	6	-0.905701	4.936649	-1.456110		6	3.283688	-0.252229	-3.741851
	1	-0.575150	2.915836	-2.116076		1	1.332559	0.403669	-3.102403
	6	-1.164014	5.776130	-0.370994		6	4.572372	-0.616860	-3.344794
	1	-1.515185	5.868649	1.755656		1	5.902362	-0.880575	-1.667253
	1	-0.783110	5.354660	-2.451786		1	3.015060	-0.257437	-4.795096
	1	-1.249870	6.849336	-0.516535		1	5.311731	-0.909785	-4.085077
	6	-2.245660	0.830514	-1.726964		6	1.627923	3.024743	-0.700489
	8	-2.043196	0.469793	-2.878259		8	0.950244	4.044070	-0.699459
	8	-3.356695	1.505538	-1.365424		8	2.881795	2.984908	-1.194645
	6	-4.279618	1.797858	-2.425934		6	3.360902	4.211507	-1.763809
	1	-5.128824	2.286768	-1.948813		1	4.369841	3.996064	-2.115281
	1	-3.821145	2.463273	-3.162721		1	2.725742	4.528442	-2.595392

Species	Cartesian coordinates				Species	Cartesian coordinates			
	1	-4.596642	0.879958	-2.927208		1	3.378261	5.005590	-1.012704
**INT3b**	6	1.792252	3.003843	1.194217	**INT3b-n**	6	-2.697921	2.614913	0.888975
	6	1.684785	2.680368	-0.178858		6	-2.434748	2.241113	-0.449820
	6	2.568429	3.253217	-1.095475		6	-3.395824	2.473995	-1.435209
	6	3.581441	4.116500	-0.666962		6	-4.630642	3.041823	-1.108015
	6	3.721210	4.400053	0.692728		6	-4.913554	3.366907	0.219932
	6	2.830368	3.849048	1.613831		6	-3.952310	3.156312	1.208522
	1	2.470848	3.011955	-2.149342		1	-3.180282	2.198978	-2.463030
	1	4.257698	4.559524	-1.392373		1	-5.365913	3.224227	-1.886384
	1	4.512844	5.061407	1.034279		1	-5.875164	3.798979	0.483399
	1	2.918525	4.092504	2.669819		1	-4.158861	3.438028	2.238167
	6	0.773398	2.520265	2.127893		6	-1.630862	2.509350	1.885362
	6	-0.397803	2.048137	1.676955		6	-0.353764	2.360698	1.503118
	6	-0.685148	1.992972	0.186913		6	0.009656	2.288833	0.032050
	6	0.585760	1.708622	-0.619895		6	-1.084536	1.599620	-0.779685
	1	0.966419	2.613155	3.193768		1	-1.892685	2.620818	2.934614
	1	-1.183928	1.744529	2.359449		1	0.451175	2.344688	2.229988
	1	-0.954542	3.021780	-0.098716		1	0.006684	3.325499	-0.335895
	1	0.376762	1.872175	-1.685277		1	-0.880436	1.736980	-1.849464
	7	1.033752	0.292926	-0.490479		7	-1.105218	0.128241	-0.536850
	6	2.305430	0.093307	-1.130378		6	-2.265516	-0.479674	-1.132913
	8	2.525778	0.413176	-2.284052		8	-2.545237	-0.354626	-2.310596
	8	3.164218	-0.478934	-0.285313		8	-2.946818	-1.172286	-0.220129
	6	4.595836	-0.689654	-0.636967		6	-4.264353	-1.798954	-0.523837
	6	5.149235	-1.329630	0.636913		6	-4.630102	-2.443622	0.813777
	1	4.640984	-2.273753	0.853292		1	-3.886986	-3.191890	1.104371
	1	6.217608	-1.531547	0.517078		1	-5.603572	-2.936110	0.734169
	1	5.013638	-0.663292	1.492802		1	-4.683978	-1.689135	1.602919
	6	4.725727	-1.635435	-1.833788		6	-4.124122	-2.852623	-1.625671
	1	5.786131	-1.841078	-2.010933		1	-5.086519	-3.356903	-1.758385
	1	4.232892	-2.592062	-1.636370		1	-3.387751	-3.614483	-1.352663
	1	4.304216	-1.192859	-2.736787		1	-3.837597	-2.399301	-2.574965
	6	5.258114	0.664077	-0.901843		6	-5.267807	-0.706968	-0.899483
	1	4.869865	1.125899	-1.810012		1	-5.010822	-0.241342	-1.851342
	1	5.092225	1.344658	-0.062740		1	-5.302170	0.066789	-0.128258
	1	6.336527	0.517255	-1.018982		1	-6.264175	-1.151791	-0.987090
	44	0.018919	-1.342930	-0.156159		44	0.331449	-1.103069	-0.066758
	6	-0.928383	-3.328909	-0.767592		6	1.777332	-2.826127	-0.528508
	6	-0.458894	-2.610136	-1.911598		6	1.130665	-2.366671	-1.715303
	6	0.980673	-2.608029	-1.861944		6	-0.258062	-2.735711	-1.623718
	6	1.390104	-3.330304	-0.715267		6	-0.458022	-3.424986	-0.403487
	6	0.217230	-3.737043	-0.024251		6	0.784522	-3.444692	0.285205
	1	-1.958111	-3.516451	-0.502347		1	2.825815	-2.729117	-0.294569
	1	-1.068239	-2.188914	-2.699063		1	1.611204	-1.891972	-2.558343
	1	1.626156	-2.140415	-2.593419		1	-1.006870	-2.527063	-2.375824
	1	2.405547	-3.487157	-0.381117		1	-1.396265	-3.802143	-0.022942
	1	0.193339	-4.266159	0.918310		1	0.950338	-3.857665	1.270549
	53	-0.661986	-1.538208	2.471762		53	0.978708	-0.885895	2.585661
	6	-1.866074	1.113431	-0.190444		6	1.408038	1.771292	-0.254022
	6	-1.743429	-0.213329	-0.374050		6	1.731043	0.464735	-0.367083
	6	-3.181220	1.821164	-0.312476		6	3.084112	0.033436	-0.812867
	6	-3.327985	2.929354	-1.166513		6	3.432616	0.148083	-2.170543
	6	-4.294060	1.412859	0.442535		6	4.040621	-0.484447	0.078225
	6	-4.546985	3.596435	-1.273394		6	4.695107	-0.240731	-2.623764
	1	-2.485025	3.259518	-1.768426		1	2.707805	0.557947	-2.869452
	6	-5.514569	2.084041	0.337651		6	5.302761	-0.864934	-0.374874
	1	-4.195762	0.571485	1.120703		1	3.788472	-0.572995	1.130122
	6	-5.646735	3.176552	-0.519914		6	5.636555	-0.749359	-1.728205
	1	-4.639847	4.443453	-1.947941		1	4.942224	-0.139810	-3.677360
	1	-6.359582	1.755354	0.936714		1	6.031694	-1.250561	0.333247
	1	-6.595582	3.699891	-0.599069		1	6.619666	-1.051460	-2.078093
	6	-2.940089	-0.999418	-0.801437		6	2.381701	2.896418	-0.459845
	8	-3.585659	-1.777444	-0.122376		8	2.062931	3.992589	-0.895782
	8	-3.225346	-0.779785	-2.112963		8	3.640494	2.619621	-0.070179
	6	-4.410317	-1.431875	-2.603151		6	4.601952	3.662299	-0.289972
	1	-5.291189	-1.073127	-2.064881		1	4.323370	4.566417	0.257352
	1	-4.475806	-1.167496	-3.658643		1	4.675861	3.902759	-1.353779
	1	-4.339742	-2.515798	-2.481815		1	5.548765	3.267073	0.077636
**INT4b**	6	1.814797	-1.902295	1.882793	**INT4b-n**	6	2.671922	-1.073353	-1.526773
	6	2.450372	-0.673742	2.138479		6	2.890476	0.143465	-2.199027

Species	Cartesian coordinates				Species	Cartesian coordinates			
	6	3.324588	-0.525033	3.204757		6	3.792233	0.229418	-3.249018
	6	3.580662	-1.646320	4.011009		6	4.503646	-0.927657	-3.606368
	6	2.949791	-2.867088	3.759071		6	4.287412	-2.136171	-2.939250
	6	2.044477	-3.004621	2.694593		6	3.352182	-2.226003	-1.895776
	1	3.813711	0.424139	3.409264		1	3.960005	1.165839	-3.774598
	1	4.283088	-1.565256	4.836092		1	5.236766	-0.880719	-4.407047
	1	3.169136	-3.723823	4.390572		1	4.855456	-3.016631	-3.227589
	1	1.555876	-3.957101	2.505876		1	3.183093	-3.170587	-1.385064
	6	0.897484	-1.658812	0.683633		6	1.573391	-0.788977	-0.502288
	6	-0.293597	-0.810210	1.237625		6	0.281158	-0.646368	-1.371205
	6	0.409755	0.572872	1.501847		6	0.487565	0.776196	-2.031570
	6	1.897815	0.289100	1.095668		6	1.938160	1.143858	-1.558486
	1	0.654977	-2.520746	0.073402		1	1.514276	-1.439458	0.361851
	1	-0.624287	-1.260429	2.176899		1	0.300937	-1.434961	-2.121966
	1	0.376247	0.849773	2.564445		1	0.442695	0.755991	-3.126149
	1	2.494740	1.162031	0.846285		1	2.195723	2.196562	-1.621240
	7	1.660924	-0.623225	-0.047901		7	1.865557	0.626515	-0.172049
	6	2.639112	-0.984038	-0.952080		6	2.789416	1.039005	0.764093
	8	2.666444	-2.057705	-1.532559		8	3.354241	2.123127	0.719976
	8	3.507628	0.039315	-1.130409		8	2.921195	0.112818	1.739397
	6	4.587206	-0.044936	-2.126053		6	3.779583	0.353893	2.909819
	6	5.271004	1.318298	-1.989505		6	3.609963	-0.934710	3.718889
	1	6.107143	1.391468	-2.691391		1	4.203833	-0.882496	4.636492
	1	4.565445	2.126380	-2.203394		1	3.942825	-1.800391	3.138954
	1	5.657208	1.456835	-0.975238		1	2.560901	-1.085437	3.988802
	6	5.553720	-1.175927	-1.758033		6	3.263982	1.561747	3.699404
	1	5.918132	-1.045075	-0.734118		1	2.212888	1.416651	3.968875
	1	5.065617	-2.147419	-1.837272		1	3.358808	2.478429	3.116668
	1	6.416590	-1.156181	-2.431867		1	3.839745	1.669109	4.624544
	6	3.996780	-0.216784	-3.529579		6	5.236168	0.527637	2.465805
	1	3.489906	-1.177118	-3.625492		1	5.360419	1.436840	1.877402
	1	3.280741	0.583680	-3.740587		1	5.552640	-0.329870	1.863660
	1	4.798083	-0.162465	-4.273873		1	5.884292	0.582814	3.346655
	44	-2.176060	-0.594737	0.154987		44	-1.776431	-0.904931	-0.542228
	6	-4.680772	-0.476961	0.308172		6	-3.991725	-1.715467	-1.174261
	6	-4.214038	-1.776500	0.579924		6	-3.124157	-2.811240	-1.068457
	6	-3.292445	-1.709488	1.687894		6	-2.008219	-2.583896	-1.952602
	6	-3.210636	-0.337995	2.100697		6	-2.256081	-1.356478	-2.664798
	6	-4.032190	0.417318	1.211081		6	-3.445930	-0.793780	-2.137972
	1	-5.347967	-0.193637	-0.495148		1	-4.891036	-1.560493	-0.591676
	1	-4.478175	-2.675355	0.039809		1	-3.232280	-3.650616	-0.395755
	1	-2.819418	-2.552496	2.173603		1	-1.196583	-3.275687	-2.132305
	1	-2.660567	0.046035	2.948274		1	-1.648691	-0.941222	-3.456406
	1	-4.160500	1.490562	1.244437		1	-3.902876	0.129511	-2.467155
	53	-1.541775	-2.348734	-1.764448		53	-0.898580	-2.201850	1.713877
	6	-0.246643	1.652298	0.688482		6	-0.522675	1.705279	-1.414773
	6	-1.427428	1.315145	0.116896		6	-1.425108	1.087745	-0.635775
	6	0.409452	2.974562	0.541746		6	-2.272808	1.467558	0.487470
	6	1.033105	3.592357	1.642121		6	-3.686812	1.518890	0.426059
	6	0.448235	3.630581	-0.702236		6	-1.639777	1.740803	1.727675
	6	1.643512	4.838436	1.509042		6	-4.432412	1.851370	1.554377
	1	1.021365	3.104931	2.612620		1	-4.185867	1.345129	-0.519795
	6	1.067360	4.872531	-0.834781		6	-2.391500	2.067886	2.846482
	1	0.015383	3.148545	-1.573648		1	-0.558127	1.678982	1.779423
	6	1.663134	5.483903	0.270414		6	-3.788898	2.121735	2.763925
	1	2.106291	5.305292	2.374162		1	-5.515215	1.902981	1.489523
	1	1.093873	5.357698	-1.806536		1	-1.894066	2.276352	3.788886
	1	2.146668	6.451041	0.166024		1	-4.373922	2.373674	3.643887
	6	-2.303229	2.243401	-0.646067		6	-0.423075	3.163091	-1.634150
	8	-2.691852	2.045630	-1.785584		8	0.369813	3.682475	-2.404758
	8	-2.698647	3.315167	0.085222		8	-1.324310	3.879175	-0.920923
	6	-3.518079	4.271450	-0.612800		6	-1.261298	5.301372	-1.104404
	1	-3.776300	5.030341	0.125492		1	-0.278440	5.685046	-0.819041
	1	-2.959479	4.718588	-1.439005		1	-1.452840	5.567076	-2.147451
	1	-4.417652	3.795416	-1.010280		1	-2.034773	5.715163	-0.457250
**INT5b**	6	-2.292614	1.877127	-1.767184	**INT5b-n**	6	-2.543485	2.225082	1.038552
	6	-2.050320	2.392503	-0.482761		6	-2.148197	2.674939	-0.232039
	6	-2.680158	3.549261	-0.045027		6	-2.727607	3.798290	-0.804136
	6	-3.574868	4.183468	-0.920303		6	-3.728096	4.464232	-0.080639
	6	-3.811869	3.674385	-2.200414		6	-4.119474	4.019104	1.185433

Species	Cartesian coordinates				Species	Cartesian coordinates			
	6	-3.165229	2.510544	-2.641662		6	-3.523526	2.889314	1.764362
	1	-2.490967	3.956051	0.944499		1	-2.421174	4.154277	-1.783437
	1	-4.092143	5.082601	-0.597521		1	-4.207528	5.338359	-0.512128
	1	-4.513014	4.180752	-2.857655		1	-4.899722	4.549880	1.723520
	1	-3.358444	2.114070	-3.634956		1	-3.832319	2.544322	2.747822
	6	-1.453674	0.617815	-1.885539		6	-1.711383	0.991168	1.329277
	6	0.065757	0.842301	-1.912672		6	-0.210836	1.253200	1.523485
	6	0.324916	1.790116	-0.726467		6	0.182949	2.127043	0.315890
	6	-0.978236	1.509334	0.122710		6	-1.001510	1.777888	-0.656554
	1	-1.842746	-0.153827	-2.543955		1	-2.159125	0.251662	1.987832
	1	0.558005	1.087442	-2.851809		1	0.158948	1.560541	2.500153
	1	0.307561	2.843718	-1.026127		1	0.142998	3.198893	0.533521
	1	-0.854564	1.498542	1.201815		1	-0.763991	1.704815	-1.714184
	7	-1.366175	0.148596	-0.441261		7	-1.439338	0.442973	-0.067204
	6	-2.550188	-0.505619	0.059202		6	-2.520317	-0.193511	-0.777461
	8	-3.186126	-1.270014	-0.636290		8	-2.776055	0.063055	-1.930763
	8	-2.786164	-0.146180	1.310900		8	-3.139556	-1.060185	0.024884
	6	-3.959657	-0.669210	2.067040		6	-4.326322	-1.825595	-0.445915
	6	-3.773164	-0.019850	3.438276		6	-4.677178	-2.671870	0.778597
	1	-4.591620	-0.315155	4.101157		1	-5.566726	-3.273198	0.570642
	1	-2.828532	-0.335951	3.888700		1	-4.886093	-2.036833	1.644648
	1	-3.772280	1.070739	3.354355		1	-3.856918	-3.350973	1.029240
	6	-5.240345	-0.178234	1.388148		6	-3.942881	-2.710271	-1.633393
	1	-5.228964	0.911470	1.289572		1	-3.080212	-3.334489	-1.384294
	1	-5.358818	-0.623203	0.399114		1	-3.698064	-2.113581	-2.511771
	1	-6.103494	-0.457129	2.000572		1	-4.785017	-3.366513	-1.875904
	6	-3.888302	-2.193810	2.163813		6	-5.455395	-0.846690	-0.778325
	1	-4.027014	-2.661575	1.188854		1	-5.202635	-0.228743	-1.640547
	1	-2.922549	-2.505618	2.569558		1	-5.663953	-0.196120	0.076620
	1	-4.678558	-2.543184	2.836253		1	-6.365321	-1.409535	-1.008539
	44	0.474337	-1.112976	-1.023565		44	0.349322	-0.736247	0.806510
	6	0.371444	-3.385188	-1.678489		6	0.255988	-3.003838	1.544220
	6	-0.416450	-2.718572	-2.632992		6	-0.673545	-2.344287	2.366277
	6	0.399203	-1.715323	-3.240194		6	0.010529	-1.274716	3.021488
	6	1.729439	-1.812142	-2.707643		6	1.399705	-1.330272	2.666027
	6	1.706524	-2.826184	-1.709913		6	1.549088	-2.378184	1.714334
	1	0.037179	-4.167146	-1.011306		1	0.045740	-3.826194	0.874730
	1	-1.470634	-2.880966	-2.813858		1	-1.731864	-2.555772	2.430892
	1	0.084538	-1.039337	-4.024212		1	-0.428194	-0.586768	3.732209
	1	2.589563	-1.228529	-3.000054		1	2.189232	-0.701988	3.052554
	1	2.546900	-3.156793	-1.119137		1	2.472279	-2.689661	1.247528
	53	0.385053	-2.191457	1.582381		53	0.482599	-1.861520	-1.774908
	6	1.630997	1.441488	-0.058271		6	1.575472	1.758903	-0.111205
	6	1.944222	0.131495	-0.155912		6	1.947335	0.484575	0.165411
	6	2.411396	2.542392	0.576987		6	3.351603	0.015077	0.238476
	6	2.691255	3.717262	-0.147222		6	4.168910	0.565084	1.248188
	6	2.844500	2.469661	1.912402		6	3.908498	-0.971108	-0.593615
	6	3.389482	4.774161	0.434854		6	5.487214	0.145502	1.419803
	1	2.374904	3.796806	-1.184334		1	3.756594	1.333759	1.896206
	6	3.538087	3.531155	2.496071		6	5.232878	-1.378385	-0.429281
	1	2.642875	1.570775	2.481890		1	3.300393	-1.407188	-1.376754
	6	3.814217	4.686181	1.762831		6	6.028255	-0.829232	0.578980
	1	3.602669	5.665875	-0.148877		1	6.093340	0.586142	2.207210
	1	3.861174	3.453245	3.530825		1	5.644161	-2.132049	-1.095873
	1	4.354289	5.510643	2.220429		1	7.056300	-1.156504	0.707544
	6	3.254540	-0.398479	0.307366		6	2.375594	2.841181	-0.727282
	8	3.710855	-0.344513	1.433246		8	1.993262	4.002050	-0.806392
	8	3.953500	-0.972652	-0.715523		8	3.558031	2.436844	-1.241840
	6	5.251487	-1.472877	-0.356373		6	4.347438	3.465186	-1.853299
	1	5.670029	-1.892728	-1.271723		1	3.811868	3.919435	-2.691264
	1	5.887096	-0.666246	0.017938		1	4.595850	4.248267	-1.131603
	1	5.171274	-2.241418	0.416829		1	5.252915	2.969394	-2.203506
**INT6b**	6	-2.903474	2.418785	0.899545	**INT6b-n**	6	3.361895	2.198476	0.495369
	6	-2.338636	1.276029	1.510395		6	2.680422	1.225898	1.261386
	6	-3.028820	0.626901	2.537190		6	3.335624	0.592583	2.320253
	6	-4.269548	1.098155	2.973688		6	4.657796	0.914444	2.637096
	6	-4.829373	2.229433	2.376226		6	5.333971	1.878508	1.886412
	6	-4.151538	2.880854	1.345691		6	4.690320	2.511801	0.823077
	1	-2.588644	-0.252562	3.000664		1	2.804357	-0.155788	2.903132
	1	-4.791703	0.586788	3.776938		1	5.152851	0.418159	3.466545

Species	Cartesian coordinates				Species	Cartesian coordinates			
	1	-5.791555	2.604979	2.713386		1	6.360653	2.138065	2.129561
	1	-4.585306	3.763596	0.881813		1	5.215353	3.264427	0.239580
	6	-2.180678	3.087426	-0.183945		6	2.672772	2.845659	-0.622204
	6	-0.888771	2.825057	-0.424684		6	1.344215	2.755441	-0.772496
	6	-0.109357	1.889866	0.479779		6	0.498974	2.056029	0.277457
	6	-0.988285	0.747519	1.047860		6	1.240912	0.860026	0.931360
	1	-2.722005	3.816276	-0.783044		1	3.272644	3.416241	-1.327766
	1	-0.354344	3.328855	-1.223943		1	0.828182	3.242833	-1.591694
	1	0.121674	2.486152	1.379706		1	0.415852	2.775408	1.112667
	1	-0.453016	0.360817	1.922512		1	0.714689	0.654557	1.870011
	7	-1.071045	-0.431928	0.150646		7	1.089914	-0.400513	0.167670
	6	-2.347692	-0.826794	-0.381723		6	2.254444	-1.039268	-0.384555
	8	-2.887224	-1.863436	-0.050406		8	2.648057	-2.113831	0.023584
	8	-2.782878	0.046564	-1.287217		8	2.761151	-0.334661	-1.393279
	6	-4.092710	-0.133159	-1.977072		6	3.983266	-0.783810	-2.120333
	6	-4.180641	1.121471	-2.846225		6	4.212040	0.355430	-3.113763
	1	-3.329695	1.179050	-3.530793		1	3.339587	0.487479	-3.759840
	1	-5.100998	1.097528	-3.437112		1	5.078639	0.128870	-3.741840
	1	-4.186797	2.021498	-2.226979		1	4.397543	1.295272	-2.588750
	6	-4.042880	-1.395117	-2.842030		6	3.685352	-2.095159	-2.851731
	1	-4.976721	-1.483092	-3.406112		1	4.556713	-2.381614	-3.448937
	1	-3.222732	-1.335400	-3.564975		1	2.839662	-1.971254	-3.536152
	1	-3.924255	-2.290164	-2.230007		1	3.469492	-2.900527	-2.148488
	6	-5.228301	-0.176297	-0.952228		6	5.158743	-0.914088	-1.149139
	1	-5.172422	-1.071646	-0.332789		1	5.004426	-1.730283	-0.443155
	1	-5.197918	0.704733	-0.306213		1	5.302366	0.015110	-0.591917
	1	-6.185277	-0.179094	-1.484046		1	6.070059	-1.115359	-1.721549
	44	0.450280	-1.517134	-0.316253		44	-0.600488	-1.286925	-0.091754
	6	1.916435	-2.860601	-1.466316		6	-2.319838	-2.538807	-0.991556
	6	1.055230	-3.751349	-0.761481		6	-1.532936	-3.445161	-0.223349
	6	-0.269894	-3.610936	-1.269306		6	-0.240834	-3.553837	-0.818687
	6	-0.245985	-2.588780	-2.249533		6	-0.198920	-2.662215	-1.917413
	6	1.108648	-2.118224	-2.378246		6	-1.487122	-2.029022	-2.029443
	1	2.983897	-2.764812	-1.341583		1	-3.350732	-2.276048	-0.810393
	1	1.355617	-4.425282	0.029252		1	-1.862190	-3.971464	0.662201
	1	-1.147630	-4.132675	-0.912312		1	0.585280	-4.143200	-0.444326
	1	-1.093943	-2.231866	-2.817955		1	0.643812	-2.498404	-2.574969
	1	1.472561	-1.360172	-3.055523		1	-1.786000	-1.328907	-2.796277
	53	1.451305	-1.878579	2.206454		53	-1.414514	-1.197697	2.533659
	6	1.238890	1.479196	-0.093049		6	-0.928081	1.767125	-0.162351
	6	1.647707	0.226281	-0.371131		6	-1.570094	0.591870	-0.305261
	6	3.052180	0.029156	-0.850233		6	-2.979806	0.576980	-0.788688
	6	2.145261	2.655787	-0.340917		6	-3.264651	0.961745	-2.112482
	6	2.527635	3.504975	0.712111		6	-4.053753	0.207016	0.040972
	6	2.611968	2.946956	-1.633370		6	-4.576807	0.972218	-2.592194
	6	3.357908	4.600829	0.481721		1	-2.448547	1.267771	-2.761138
	1	2.191613	3.292499	1.723435		6	-5.362650	0.224256	-0.438011
	6	3.441745	4.046806	-1.864603		1	-3.851303	-0.086269	1.065521
	1	2.323721	2.303247	-2.458350		6	-5.631553	0.601808	-1.757696
	6	3.816990	4.877954	-0.808857		1	-4.771062	1.274518	-3.617854
	1	3.650274	5.237800	1.312229		1	-6.178133	-0.055399	0.223839
	1	3.789422	4.254247	-2.873097		1	-6.652795	0.609051	-2.128286
	1	4.460832	5.734587	-0.987951		6	-1.685895	3.046550	-0.437027
	8	3.385793	-0.259687	-1.990314		8	-1.445060	3.822095	-1.345349
	8	3.948073	0.194266	0.141992		8	-2.645423	3.269798	0.480450
	6	5.328393	0.065583	-0.237091		6	-3.444039	4.448337	0.270173
	1	5.897442	0.203197	0.682025		1	-3.974404	4.382916	-0.683116
	1	5.524128	-0.921120	-0.664415		1	-2.817732	5.343755	0.269104
	1	5.595079	0.829850	-0.971645		1	-4.150364	4.474112	1.099492
**P3b**	6	-4.685344	-2.153648	0.436680	**INT8b**	6	-1.776107	-0.046237	2.113755
	6	-3.583805	-2.596066	1.166673		6	-1.347376	1.062384	1.210729
	6	-2.277575	-2.313393	0.739876		6	0.031213	1.521015	1.747529
	6	-3.196188	-1.118532	-1.157190		6	0.402190	-1.000094	1.439585
	6	-4.492706	-1.430893	-0.742198		6	-0.888246	-1.137193	2.215469
	1	-5.690217	-2.391257	0.774211		7	0.134933	-0.928381	-0.017558
	1	-3.728546	-3.195111	2.062139		1	-2.069985	1.880042	1.181247
	1	-3.042310	-0.540918	-2.063414		1	1.063615	-1.839962	1.642941
	1	-5.344800	-1.110136	-1.334407		6	-1.186626	-2.222643	3.036063
	6	-2.087015	-1.524243	-0.415021		1	-0.494876	-3.059576	3.100116
	6	-1.120871	-2.916592	1.405389		6	-2.367149	-2.225797	3.784571

Species	Cartesian coordinates				Species	Cartesian coordinates			
	6	0.372417	-2.258219	-0.492848		1	-2.599213	-3.068153	4.430129
	6	0.083256	-2.951513	0.820049		6	-3.243153	-1.139979	3.704463
	1	-1.290566	-3.423274	2.352612		1	-4.158382	-1.138332	4.290381
	1	0.914834	-3.483670	1.272212		6	-2.954104	-0.059047	2.870273
	6	-0.681135	-1.181670	-0.861433		1	-3.646199	0.775852	2.800061
	6	1.644935	-1.434073	-0.365242		6	1.025878	0.330167	1.939384
	6	1.319691	-0.134586	-0.121420		6	2.027379	1.131387	1.116026
	1	0.466285	-3.004100	-1.290910		6	1.152137	2.168825	0.946134
	1	-0.676317	-1.022555	-1.946519		1	1.337101	0.186790	2.980993
	7	-0.065010	0.050001	-0.236264		1	-0.156500	2.054365	2.690929
	6	-0.717602	1.199207	0.222636		1	0.090806	0.081240	-3.731350
	8	-1.808060	1.453330	-0.520141		6	-0.465525	0.788605	-3.136287
	8	-0.304942	1.871711	1.148206		6	0.080502	1.682843	-2.184973
	6	-2.707938	2.580632	-0.170695		6	-1.880957	0.896123	-3.066282
	6	-1.948436	3.905421	-0.291962		6	-1.003704	2.396612	-1.560696
	1	-2.647044	4.735857	-0.148294		1	1.130265	1.828488	-1.971457
	1	-1.155627	3.977692	0.451656		44	-1.217216	0.279687	-0.852357
	1	-1.509940	4.001850	-1.290256		6	-2.213881	1.923290	-2.128794
	6	-3.797855	2.490928	-1.240424		1	-2.592307	0.305333	-3.627232
	1	-3.363795	2.565897	-2.241817		1	-0.922149	3.189749	-0.833516
	1	-4.342446	1.547291	-1.164098		53	-3.728395	-0.787286	-0.601230
	1	-4.508144	3.312798	-1.111518		1	-3.209810	2.262083	-1.882131
	6	-3.296119	2.363071	1.225884		1	2.419520	-5.227639	-2.100041
	1	-2.525233	2.425362	1.994325		6	1.797460	-4.433146	-1.675504
	1	-4.050264	3.131254	1.424576		6	2.696275	-3.406589	-0.979483
	1	-3.781767	1.384621	1.284348		1	1.110093	-4.888737	-0.955846
	6	2.937112	-2.101713	-0.481462		1	1.218010	-3.975967	-2.477390
	8	3.047551	-3.319626	-0.550475		8	1.866940	-2.419016	-0.251469
	8	4.003125	-1.269655	-0.533694		6	3.491767	-4.064271	0.150665
	6	5.280326	-1.913822	-0.646591		6	3.631249	-2.684714	-1.954177
	1	6.013372	-1.107391	-0.655351		6	0.951562	-1.664879	-0.890904
	1	5.456075	-2.581556	0.200711		1	2.820490	-4.540392	0.871514
	1	5.341305	-2.495905	-1.569757		1	4.156459	-4.830297	-0.259395
	6	2.221988	1.015931	0.123581		1	4.099998	-3.322630	0.675054
	6	2.233115	2.099144	-0.766133		1	3.067232	-2.204773	-2.753801
	6	3.083049	1.026302	1.227467		1	4.220432	-1.928284	-1.427691
	6	3.099499	3.170732	-0.558939		1	4.322499	-3.409785	-2.396272
	1	1.569377	2.095173	-1.626210		8	0.807617	-1.648448	-2.106409
	6	3.939717	2.104381	1.439296		6	1.302821	3.530958	0.428876
	1	3.070707	0.191666	1.920557		6	3.427315	0.836201	0.836035
	6	3.952707	3.177617	0.546079		6	4.029109	-0.258526	1.490062
	1	3.106684	4.000862	-1.259419		6	4.211807	1.606398	-0.047653
	1	4.597381	2.106996	2.303760		6	5.370506	-0.567475	1.279632
	1	4.623337	4.016047	0.711243		1	3.441648	-0.860829	2.175534
						6	5.549577	1.284002	-0.264221
						1	3.763056	2.457677	-0.545407
						6	6.135086	0.200872	0.397125
						1	5.820939	-1.405391	1.804391
						1	6.140525	1.884827	-0.949880
						1	7.180732	-0.041447	0.228920
						8	2.187981	3.943121	-0.306884
						8	0.299529	4.331195	0.875746
						6	0.379563	5.708951	0.468693
						1	-0.485211	6.197152	0.917158
						1	1.306118	6.161398	0.830073
						1	0.347032	5.792692	-0.620613
**TS11b**	6	1.134973	0.783622	2.376548	**INT9b**	6	1.855556	0.800595	2.418979
	6	0.230060	-0.247013	1.910879		6	1.251726	-0.517964	2.513508
	6	-1.200677	0.030946	2.247936		6	1.196084	1.790392	1.655662
	6	0.730586	2.098943	2.031561		6	-0.045065	1.367734	0.891414
	6	-0.410643	2.056971	1.024440		7	0.313491	0.547539	-0.282395
	7	-0.028282	1.083411	-0.011749		1	1.844666	-1.322784	2.940850
	1	0.581548	-1.261876	1.749255		1	-0.610399	2.247234	0.576221
	1	-0.617482	3.038688	0.589121		6	1.732405	3.073127	1.592129
	6	1.450914	3.189390	2.489363		1	1.237761	3.828627	0.989133
	1	1.163023	4.198437	2.208250		6	2.901253	3.383527	2.295065
	6	2.587048	2.972120	3.285971		1	3.305876	4.390809	2.248245
	1	3.160310	3.823428	3.642635		6	3.555881	2.405303	3.049751
	6	3.012688	1.676846	3.592365		1	4.467765	2.649680	3.586219
	1	3.908029	1.528175	4.188184		6	3.043398	1.111887	3.099262

Species	Cartesian coordinates				Species	Cartesian coordinates			
	6	2.300148	0.569794	3.125141		1	3.552671	0.339394	3.669050
	1	2.620895	-0.440418	3.356728		44	0.348433	-1.477095	0.047803
	44	0.445891	-1.278725	-1.116786		6	-0.924608	0.480682	1.822425
	6	-1.614825	1.463704	1.768903		1	-1.213504	1.072498	2.701414
	6	-2.743788	0.765734	1.008355		6	-2.105167	-0.011546	1.034428
	6	-2.380334	-0.474923	1.451518		6	-1.785388	-0.959100	0.133580
	1	-1.954879	2.133702	2.564915		6	-0.081421	-0.717009	2.226793
	1	-1.287586	-0.117296	3.336344		1	-0.587190	-1.595027	2.618392
	6	-0.827209	-1.443869	-2.907760		6	1.546116	-3.448628	-0.401505
	6	-1.603115	-1.826065	-1.776694		6	1.118876	-2.837878	-1.649994
	6	0.295097	-2.343121	-2.993457		6	0.400291	-3.825682	0.329510
	6	-0.993237	-2.981851	-1.171430		6	-0.282679	-2.833781	-1.667897
	6	0.167601	-3.300872	-1.930257		6	-0.732904	-3.430046	-0.419144
	1	-1.030806	-0.612765	-3.569536		1	2.569008	-3.625014	-0.108827
	1	-2.502458	-1.337458	-1.430936		1	1.769848	-2.438335	-2.412974
	1	1.076378	-2.326828	-3.741259		1	0.389299	-4.297740	1.302628
	1	-1.354855	-3.522450	-0.310269		1	-0.912060	-2.434345	-2.447934
	1	0.865124	-4.099065	-1.717710		1	-1.756448	-3.616709	-0.130243
	53	2.828102	-2.159868	0.139114		53	3.414640	-0.869379	-0.170994
	6	0.959332	1.426561	-0.848916		6	0.368364	1.070265	-1.539331
	8	1.454546	0.579711	-1.640488		8	0.506968	0.397363	-2.557073
	8	1.362218	2.710567	-0.826464		8	0.277955	2.435079	-1.530632
	6	2.392334	3.240123	-1.745441		6	0.329286	3.204963	-2.778869
	6	3.730982	2.545444	-1.480581		6	0.198199	4.650237	-2.288093
	1	3.687208	1.487847	-1.740914		1	1.027821	4.908869	-1.623060
	1	4.514089	3.025385	-2.076753		1	0.210536	5.339565	-3.137859
	1	4.001932	2.635441	-0.424331		1	-0.740425	4.790396	-1.743266
	6	1.928404	3.092994	-3.197650		6	1.677191	2.995993	-3.478152
	1	1.870520	2.043982	-3.488359		1	2.498523	3.227454	-2.792743
	1	0.945096	3.555021	-3.332355		1	1.783481	1.965686	-3.816471
	1	2.636244	3.602832	-3.859131		1	1.752211	3.666354	-4.341057
	6	2.456629	4.716382	-1.344850		6	-0.855476	2.833632	-3.678061
	1	3.200831	5.237265	-1.954287		1	-0.784113	1.796193	-4.004334
	1	1.487138	5.201347	-1.493556		1	-1.798327	2.970484	-3.138351
	1	2.738416	4.817453	-0.292872		1	-0.869832	3.485051	-4.558351
	6	-3.794084	1.364122	0.198686		6	-3.423985	0.660022	1.186113
	6	-3.813950	2.764541	0.038538		6	-3.968762	0.862091	2.467303
	6	-4.798467	0.596742	-0.428257		6	-4.145306	1.130849	0.074641
	6	-4.800001	3.380720	-0.726773		6	-5.201322	1.491971	2.630583
	1	-3.052760	3.370645	0.520128		1	-3.430170	0.505894	3.341496
	6	-5.781521	1.219838	-1.192547		6	-5.378655	1.763249	0.239815
	1	-4.798762	-0.479678	-0.298510		1	-3.727914	1.017717	-0.920857
	6	-5.786537	2.609245	-1.346342		6	-5.912786	1.944666	1.516437
	1	-4.800167	4.460953	-0.840344		1	-5.607691	1.628870	3.629039
	1	-6.549640	0.618204	-1.670230		1	-5.915948	2.125521	-0.632495
	1	-6.556257	3.088674	-1.944664		1	-6.871070	2.440249	1.644012
	6	-2.951905	-1.820142	1.391125		6	-2.818004	-1.473062	-0.807807
	8	-3.904393	-2.183614	0.716550		8	-2.852496	-1.293482	-2.011759
	8	-2.283616	-2.651724	2.226039		8	-3.755867	-2.209870	-0.157771
	6	-2.799383	-3.991076	2.319528		6	-4.860536	-2.658101	-0.966060
	1	-3.823709	-3.979869	2.700536		1	-5.472121	-3.276188	-0.309114
	1	-2.792890	-4.479674	1.342105		1	-5.435264	-1.801308	-1.326664
	1	-2.137940	-4.511683	3.011105		1	-4.506262	-3.235648	-1.823001
**TS12b**	6	1.812662	0.096770	2.337466	**INT10b**	6	-1.486310	-1.291998	2.209748
	6	1.251692	-1.170141	1.803765		6	-1.635942	-0.056952	1.380276
	6	1.120681	1.296981	2.080362		6	-0.616135	0.957540	1.949493
	6	-0.130654	1.181240	1.232000		6	0.868481	-1.086571	1.488619
	7	0.149670	0.771906	-0.152642		6	-0.187690	-1.846145	2.268619
	1	1.805346	-2.072999	2.050667		7	0.528874	-1.069882	0.050340
	1	-0.686349	2.118852	1.230385		1	-2.649997	0.343466	1.407534
	6	1.581224	2.494048	2.618602		1	1.849710	-1.541153	1.621268
	1	1.046195	3.416951	2.408217		6	0.056406	-3.003374	3.001433
	6	2.726871	2.511384	3.421910		1	1.059934	-3.421575	3.035848
	1	3.082090	3.448795	3.840478		6	-0.991301	-3.632076	3.687468
	6	3.409859	1.322758	3.683835		1	-0.802064	-4.538784	4.254968
	1	4.300317	1.331959	4.306256		6	-2.276277	-3.088255	3.640182
	6	2.956627	0.118302	3.141909		1	-3.088703	-3.569165	4.178540
	1	3.494648	-0.805640	3.337660		6	-2.523354	-1.921473	2.908199
	44	0.817567	-1.178864	-0.389703		1	-3.523804	-1.495340	2.884361
	6	-0.986066	0.059644	1.873831		6	0.826165	0.355410	2.044865

Species	Cartesian coordinates				Species	Cartesian coordinates			
	1	-1.290589	0.341992	2.887860		6	1.292624	1.555674	1.230438
	6	-2.089538	-0.419391	0.974633		6	0.038207	2.092099	1.181076
	6	-1.489097	-1.465556	0.338947		1	1.221764	0.347365	3.067933
	6	-0.180454	-1.242818	1.853215		1	-1.003006	1.286409	2.924854
	1	-0.623232	-2.113154	2.329577		1	-1.902436	-3.329051	0.553190
	6	2.168565	-2.097024	-2.152374		6	-2.067743	-2.842561	-0.396021
	6	0.916627	-1.719070	-2.718846		6	-1.287832	-3.022111	-1.587596
	6	1.946889	-3.074004	-1.125548		6	-3.167624	-1.982982	-0.721493
	6	-0.081119	-2.467458	-2.070738		6	-1.892367	-2.304539	-2.635768
	6	0.560440	-3.312462	-1.071322		1	-0.368969	-3.586201	-1.659053
	1	3.132085	-1.745454	-2.482280		44	-1.316503	-0.770602	-0.678780
	1	0.759224	-0.950269	-3.459640		6	-3.027001	-1.634104	-2.102804
	1	2.712402	-3.551477	-0.530311		1	-3.983917	-1.707297	-0.068441
	1	-1.135775	-2.429028	-2.297997		1	-1.504668	-2.195615	-3.639224
	1	0.070363	-4.025279	-0.426252		53	-2.361872	1.596093	-1.491457
	53	3.409990	0.257685	-0.579238		1	-3.688323	-0.981475	-2.653938
	6	-0.202788	1.537774	-1.215494		6	3.810360	-2.090234	-1.261639
	8	-0.112906	1.176762	-2.390288		8	2.657067	-1.774060	-0.392682
	8	-0.665385	2.774039	-0.846245		6	1.460449	-1.420863	-0.909042
	6	-1.147495	3.734935	-1.840863		8	1.203231	-1.404902	-2.112186
	6	-0.006142	4.150807	-2.775900		6	3.508690	-3.347588	-2.083446
	1	0.330334	3.304148	-3.373787		1	2.708667	-3.162760	-2.800412
	1	-0.346401	4.949685	-3.443634		1	4.407964	-3.652067	-2.628628
	1	0.840230	4.529040	-2.193935		1	3.217029	-4.172519	-1.425095
	6	-2.344239	3.157316	-2.606549		6	4.922626	-2.361665	-0.245525
	1	-2.037126	2.314867	-3.226063		1	5.847393	-2.626561	-0.766592
	1	-3.114123	2.818350	-1.905554		1	5.110067	-1.473518	0.363708
	1	-2.781928	3.930713	-3.246782		1	4.650106	-3.189441	0.416180
	6	-1.589013	4.919039	-0.973998		6	4.160241	-0.885900	-2.140628
	1	-0.747456	5.301933	-0.388940		1	5.084583	-1.096888	-2.688505
	1	-1.968786	5.728629	-1.604765		1	3.367129	-0.678625	-2.859073
	1	-2.382192	4.617513	-0.283002		1	4.324035	0.002260	-1.524060
	6	-3.467594	0.075187	0.972168		6	2.645899	1.940750	0.848319
	6	-3.989514	0.721883	2.111994		6	3.736716	1.251899	1.416017
	6	-4.284079	-0.020605	-0.174450		6	2.907340	2.981709	-0.065659
	6	-5.284273	1.234238	2.113090		6	5.046937	1.599943	1.095149
	1	-3.385028	0.811200	3.009016		1	3.552605	0.448667	2.123118
	6	-5.577091	0.497304	-0.167851		6	4.219679	3.317027	-0.391715
	1	-3.892511	-0.493896	-1.065874		1	2.073138	3.520398	-0.499732
	6	-6.084295	1.123526	0.973372		6	5.293015	2.632865	0.186149
	1	-5.668577	1.721683	3.004614		1	5.875801	1.068012	1.554301
	1	-6.188266	0.418456	-1.062472		1	4.406221	4.119581	-1.099970
	1	-7.092496	1.528037	0.972736		1	6.314129	2.903200	-0.068514
	6	-2.250820	-2.662328	-0.087792		6	-0.469453	3.427393	0.836653
	8	-3.085669	-2.700135	-0.973938		8	0.074959	4.254080	0.121728
	8	-1.956423	-3.736567	0.683993		8	-1.635688	3.669017	1.481040
	6	-2.720571	-4.928271	0.402202		6	-2.229359	4.947958	1.217810
	1	-2.567017	-5.249172	-0.630789		1	-3.130271	4.986443	1.830290
	1	-2.351441	-5.680391	1.098542		1	-1.545711	5.756542	1.489330
	1	-3.784717	-4.743493	0.563520		1	-2.484415	5.040130	0.158709
**TS13b**	6	1.974441	-0.858315	2.392543	**INT11b**	6	2.256270	1.718936	2.121121
	6	0.668757	-1.288918	1.913484		6	1.109989	1.152348	2.768226
	6	-0.460635	-0.494716	2.516972		6	2.166537	2.045825	0.738394
	6	2.186340	0.542034	2.339802		6	0.951998	1.533591	-0.023580
	6	1.076722	1.221062	1.544943		7	1.114247	0.070126	-0.078300
	7	0.913185	0.425260	0.326318		1	1.211488	0.834694	3.803364
	1	0.522562	-2.335866	1.686995		1	0.889111	1.991375	-1.016521
	1	1.300022	2.268325	1.318855		6	3.230855	2.717528	0.135947
	6	3.342604	1.091470	2.868808		1	3.167561	2.975438	-0.915246
	1	3.517969	2.162211	2.814455		6	4.362292	3.058920	0.877890
	6	4.306150	0.243173	3.437675		1	5.170578	3.606983	0.401302
	1	5.220501	0.667556	3.842949		6	4.471694	2.699940	2.230614
	6	4.118732	-1.141596	3.459850		1	5.365792	2.955185	2.791056
	1	4.881110	-1.784428	3.889501		6	3.428209	2.026225	2.846393
	6	2.955569	-1.702997	2.926715		1	3.490010	1.754150	3.897155
	1	2.797346	-2.777739	2.955504		44	-0.211468	-1.037703	1.073517
	44	-0.027599	-1.452077	-1.021480		6	-0.301376	1.851857	0.822462
	6	-0.201182	1.041069	2.376611		1	-0.304638	2.938990	0.995508
	6	-1.579706	1.117185	1.724328		6	-1.638707	1.410400	0.250079
	6	-1.802158	-0.224795	1.863016		6	-1.893879	0.100423	0.453477

Species	Cartesian coordinates				Species	Cartesian coordinates			
	1	-0.123773	1.577495	3.327935		6	-0.128873	1.126589	2.151108
	1	-0.533030	-0.837562	3.561997		1	-1.009505	1.008364	2.775376
	6	0.245206	-3.637077	-0.617467		6	0.429538	-1.943617	3.112783
	6	0.250963	-3.381336	-2.024716		6	1.081220	-2.795919	2.181163
	6	-1.038984	-3.225696	-0.116550		6	-1.000591	-2.045430	2.933210
	6	-1.018534	-2.833343	-2.384991		6	0.078107	-3.376207	1.384141
	6	-1.828034	-2.742204	-1.196856		6	-1.219937	-2.916763	1.848601
	1	1.049167	-4.083033	-0.047028		1	0.925434	-1.370949	3.883401
	1	1.079567	-3.549287	-2.699928		1	2.146786	-2.905273	2.042279
	1	-1.361550	-3.277789	0.913729		1	-1.764809	-1.557933	3.521121
	1	-1.313910	-2.524733	-3.377259		1	0.243162	-4.050387	0.556848
	1	-2.837692	-2.361803	-1.136863		1	-2.181431	-3.203554	1.451717
	53	-1.118847	0.648863	-2.563291		53	-0.874710	-2.133306	-1.516820
	6	1.920620	0.372881	-0.545695		6	2.150882	-0.512396	-0.745105
	8	1.912292	-0.532279	-1.427688		8	2.508840	-1.676859	-0.579893
	8	2.886825	1.297797	-0.445141		8	2.731455	0.337203	-1.647428
	6	3.997530	1.397247	-1.416467		6	3.843335	-0.108860	-2.496672
	6	3.446433	1.672511	-2.817544		6	-2.518209	2.459455	-0.341599
	1	2.860249	0.830387	-3.185412		6	-2.864879	3.602390	0.401340
	1	2.809298	2.561608	-2.808765		6	-2.960339	2.370416	-1.671895
	1	4.279061	1.853658	-3.505355		6	-3.642133	4.615253	-0.160150
	6	4.781354	2.602580	-0.891293		1	-2.537317	3.691233	1.434378
	1	5.131992	2.420853	0.128954		6	-3.728296	3.388983	-2.236884
	1	5.650482	2.789705	-1.528652		1	-2.693490	1.497696	-2.257213
	1	4.154264	3.498721	-0.889333		6	-4.074764	4.513041	-1.484181
	6	4.851048	0.126562	-1.357080		1	-3.908988	5.483774	0.436107
	1	5.168063	-0.070265	-0.328069		1	-4.054468	3.304394	-3.270107
	1	4.297468	-0.736254	-1.728322		1	-4.674622	5.304134	-1.925756
	1	5.747583	0.260305	-1.971143		6	3.389073	-1.256550	-3.405903
	6	-2.301100	2.296168	1.278687		1	3.136099	-2.139128	-2.819372
	6	-1.751681	3.567124	1.542600		1	2.509687	-0.955998	-3.983787
	6	-3.532647	2.216325	0.597003		1	4.190983	-1.508885	-4.108148
	6	-2.415087	4.725243	1.148597		6	4.155711	1.135755	-3.334171
	1	-0.802009	3.644859	2.063759		1	4.490464	1.962054	-2.700019
	6	-4.190588	3.378711	0.206004		1	4.950157	0.914226	-4.053353
	1	-3.953694	1.242292	0.380694		1	3.269083	1.459225	-3.887344
	6	-3.637933	4.633292	0.478916		6	5.053276	-0.495335	-1.637427
	1	-1.979170	5.697579	1.359387		1	4.835720	-1.379468	-1.038461
	1	-5.136154	3.305838	-0.323536		1	5.912427	-0.705303	-2.283517
	1	-4.155634	5.536034	0.166482		1	5.322226	0.328417	-0.968519
	6	-2.981594	-1.080706	1.737632		6	-3.243777	-0.517928	0.403134
	8	-4.018780	-0.828552	1.147989		8	-3.694468	-1.222439	1.295671
	8	-2.802923	-2.242218	2.430772		8	-3.961220	-0.206248	-0.693636
	6	-3.938119	-3.125878	2.448472		6	-5.287184	-0.757207	-0.745964
	1	-4.181872	-3.464264	1.438213		1	-5.879609	-0.425238	0.110301
	1	-3.643833	-3.969226	3.073014		1	-5.719566	-0.388863	-1.676125
	1	-4.809710	-2.620707	2.871078		1	-5.246902	-1.849350	-0.748648
**TS14b**	6	2.905231	-1.883627	0.964564					
	6	1.539807	-2.438665	0.898991					
	6	3.051984	-0.512029	1.268871					
	6	1.767471	0.295986	1.259456					
	7	1.205370	0.224643	-0.094619					
	1	1.479922	-3.516734	0.777785					
	1	1.943948	1.330470	1.558155					
	6	4.319660	0.028340	1.450567					
	1	4.427100	1.086412	1.671427					
	6	5.452253	-0.786219	1.320886					
	1	6.441749	-0.358705	1.454814					
	6	5.313439	-2.138652	1.004933					
	1	6.193253	-2.766727	0.900814					
	6	4.041875	-2.689038	0.827674					
	1	3.930635	-3.747571	0.605055					
	44	0.118078	-1.452579	-0.485371					
	6	0.757350	-0.397811	2.225118					
	1	1.125065	-0.326442	3.254638					
	6	-0.678094	0.016842	2.038439					
	6	-1.152666	-1.007607	1.230036					
	6	0.544220	-1.840744	1.760972					
	1	0.025984	-2.517797	2.437031					
	6	0.790172	-2.018411	-2.607044					

Species	Cartesian coordinates	Species	Cartesian coordinates
	6 -0.492085 -1.420670 -2.663153<br>6 0.673936 -3.211466 -1.832387<br>6 -1.416981 -2.271044 -1.941927<br>6 -0.695876 -3.382018 -1.444550<br>1 1.697605 -1.601196 -3.018264<br>1 -0.743242 -0.475242 -3.122551<br>1 1.485677 -3.889628 -1.604429<br>1 -2.470810 -2.060288 -1.821957<br>1 -1.095036 -4.194089 -0.854393<br>53 -3.041141 1.209354 -1.964140<br>6 1.657504 1.085697 -1.070432<br>8 1.430753 0.945855 -2.266319<br>8 2.370680 2.115151 -0.541677<br>6 2.818188 3.241849 -1.380232<br>6 3.826589 2.750193 -2.424052<br>1 3.348578 2.088488 -3.145964<br>1 4.251158 3.608035 -2.955828<br>1 4.646175 2.212329 -1.936387<br>6 1.609766 3.937927 -2.013851<br>1 1.104994 3.284351 -2.724997<br>1 0.893015 4.233972 -1.241906<br>1 1.941613 4.841300 -2.536178<br>6 3.499906 4.161143 -0.362789<br>1 4.339205 3.651113 0.120320<br>1 3.882913 5.055363 -0.863303<br>1 2.791759 4.474691 0.409847<br>6 -1.300546 1.197006 2.596180<br>6 -0.692968 1.856388 3.692013<br>6 -2.468323 1.755425 2.027942<br>6 -1.254340 3.009637 4.223355<br>1 0.208619 1.450098 4.139733<br>6 -3.011473 2.922139 2.557729<br>1 -2.916883 1.296493 1.156254<br>6 -2.417257 3.545692 3.656871<br>1 -0.787878 3.495349 5.075392<br>1 -3.898458 3.348956 2.099724<br>1 -2.851100 4.452914 4.068184<br>6 -2.514355 -1.595036 1.317805<br>8 -3.513755 -0.977390 1.620340<br>8 -2.503413 -2.930330 1.099623<br>6 -3.783287 -3.577666 1.257596<br>1 -3.602177 -4.633664 1.059418<br>1 -4.159996 -3.436140 2.273269<br>1 -4.506687 -3.168543 0.549151		

Table S6.The optimized cartesian coordinates (Å) for reaction **c**, located at IDSCRF-B3LYP/BS1 level in dioxane solvent.

Species	Cartesian coordinates	Species	Cartesian coordinates
**R2c**	6 0.586445 -0.146989 0.034703<br>6 -0.569135 0.206995 0.006700<br>6 1.976412 -0.594358 0.078795<br>1 2.201181 -0.942392 1.100101<br>1 2.092989 -1.466225 -0.585634<br>6 -1.964571 0.635630 -0.049453<br>1 -2.563736 -0.157736 -0.525685<br>1 -2.034353 1.520414 -0.702892<br>8 2.840659 0.471477 -0.309402<br>1 3.750115 0.142248 -0.246528<br>8 -2.436314 0.921654 1.265584<br>1 -3.353992 1.222581 1.183210	**P1c**	6 2.497457 3.568450 0.155021<br>6 1.266720 3.572411 0.807926<br>6 0.268198 2.648026 0.466124<br>6 1.760773 1.698602 -1.183417<br>6 2.737446 2.644087 -0.862851<br>1 3.256458 4.298399 0.422164<br>1 1.054997 4.320162 1.568152<br>1 1.952838 0.967482 -1.961699<br>1 3.680130 2.654103 -1.402286<br>6 0.541573 1.662595 -0.507005<br>6 -1.085805 2.773080 1.007329<br>6 -1.966585 1.207081 -0.738794<br>6 -2.128539 2.173379 0.419398<br>1 -1.237291 3.449702 1.845368<br>1 -3.143687 2.325798 0.771274<br>6 -0.526924 0.645954 -0.867789<br>6 -2.784369 -0.039171 -0.455459<br>6 -1.988236 -0.998018 0.050482<br>1 -2.272340 1.695957 -1.675216<br>1 -0.361382 0.309186 -1.898420

Species	Cartesian coordinates				Species	Cartesian coordinates			
						7	-0.615012	-0.604444	-0.016669
						6	0.417036	-1.461232	0.290215
						8	1.585381	-1.072357	-0.243705
						8	0.274587	-2.472705	0.978805
						6	2.834505	-1.817750	0.057737
						6	-4.266348	-0.079432	-0.635450
						1	-4.511466	0.366063	-1.612671
						1	-4.620711	-1.118651	-0.659635
						6	-2.443738	-2.311939	0.637569
						1	-2.134563	-2.353618	1.689157
						1	-3.535590	-2.320129	0.618700
						6	2.733378	-3.252704	-0.469353
						1	3.712639	-3.735048	-0.387243
						1	2.006483	-3.835798	0.094080
						1	2.446787	-3.249565	-1.525686
						6	3.896265	-1.042392	-0.724513
						1	3.671352	-1.046098	-1.795181
						1	3.954955	-0.006606	-0.383715
						1	4.872426	-1.514121	-0.579051
						6	3.124235	-1.757620	1.560007
						1	2.374122	-2.304227	2.131125
						1	4.106170	-2.199610	1.756740
						1	3.145512	-0.717922	1.900187
						8	-4.911422	0.655493	0.416422
						1	-5.842473	0.766693	0.170418
						8	-2.006462	-3.457573	-0.075418
						1	-1.078678	-3.566674	0.195025
**P2c**	6	0.394979	2.105429	-0.686070	**TS14c**	6	2.703765	-2.113990	0.149425
	6	0.767019	0.716496	-1.205709		6	1.298245	-2.478814	-0.090085
	6	2.184257	0.433854	-0.622093		6	2.973871	-1.018492	1.001491
	6	0.335587	0.456971	0.979432		6	1.771170	-0.170455	1.369865
	6	0.128089	1.943293	0.687522		7	1.202962	0.375315	0.128045
	7	-0.060744	-0.135926	-0.321490		1	1.142752	-3.391225	-0.659891
	6	-1.332715	-0.507761	-0.676549		1	2.048930	0.630675	2.057628
	8	-1.732692	-0.548314	-1.830872		6	4.284057	-0.727005	1.361602
	8	-2.038592	-0.871437	0.423382		1	4.488377	0.119945	2.009980
	6	-3.414886	-1.379136	0.309394		6	5.336107	-1.508445	0.865893
	1	0.622663	0.503070	-2.261508		1	6.359375	-1.270967	1.142004
	1	-0.191210	0.032768	1.831003		6	5.076368	-2.577829	0.006562
	6	-0.195020	3.029349	1.485643		1	5.895117	-3.177760	-0.379227
	1	-0.395721	2.911446	2.547313		6	3.762205	-2.882859	-0.352093
	6	-0.273657	4.295584	0.882669		1	3.554464	-3.731661	-0.998983
	1	-0.548303	5.158841	1.482409		44	-0.069188	-0.893546	-0.844950
	6	-0.010217	4.456621	-0.478135		6	0.692923	-1.101368	1.992147
	1	-0.082946	5.443882	-0.925752		1	1.012635	-1.455201	2.980226
	6	0.341039	3.357123	-1.278681		6	-0.725110	-0.597046	1.988287
	1	0.549176	3.490129	-2.336966		6	-1.267070	-1.298466	0.949769
	6	1.875708	0.234479	0.917954		6	0.371402	-2.235647	1.005995
	6	2.387366	-1.183644	0.712021		1	-0.107937	-3.129288	1.408156
	6	2.667636	-1.006151	-0.591894		6	0.516757	-0.615022	-3.010846
	1	2.421079	0.860776	1.627732		6	-0.604493	0.201440	-2.704180
	1	2.908471	1.204257	-0.902389		6	0.111249	-1.977281	-2.845829
	6	2.462413	-2.327696	1.664253		6	-1.717594	-0.666783	-2.391424
	1	1.437387	-2.629879	1.938575		6	-1.273394	-2.009195	-2.493186
	1	2.935203	-3.194068	1.176195		1	1.505583	-0.262877	-3.266597
	6	3.242753	-1.845768	-1.682140		1	-0.611101	1.281335	-2.693954
	1	3.337961	-2.890478	-1.347382		1	0.745729	-2.841399	-2.993046
	1	4.260785	-1.485702	-1.910963		1	-2.704837	-0.322354	-2.099024
	6	-3.432407	-2.674604	-0.508376		1	-1.866803	-2.897841	-2.323781
	1	-4.438785	-3.105470	-0.492769		53	-4.537330	1.229145	-0.157913
	1	-3.144040	-2.486666	-1.542600		6	-1.410536	0.250993	3.031428
	1	-2.742779	-3.405992	-0.075225		1	-0.666175	0.851851	3.562824
	6	-3.788795	-1.654790	1.768176		1	-2.123102	0.932915	2.550215
	1	-3.733380	-0.737126	2.360982		6	-2.663793	-1.866630	0.778731
	1	-4.809376	-2.044199	1.827623		1	-2.577521	-2.961242	0.834160
	1	-3.110742	-2.391994	2.208018		1	-3.077833	-1.613922	-0.200110
	6	-4.325275	-0.300890	-0.287552		6	1.760113	1.502883	-0.433287
	1	-5.365508	-0.641102	-0.258947		8	1.498088	1.914845	-1.559230
	1	-4.252670	0.621937	0.296420		8	2.622150	2.115118	0.418767
	1	-4.051584	-0.087120	-1.320703		6	3.267391	3.395595	0.071040

Species	Cartesian coordinates				Species	Cartesian coordinates			
	8	2.400956	-1.732006	-2.829090		6	4.180446	3.207225	-1.144839
	1	2.845649	-2.166171	-3.571723		6	2.206387	4.477316	-0.153644
	8	3.191767	-1.912356	2.820995		6	4.092440	3.700958	1.324127
	1	3.110402	-2.610358	3.487370		1	4.754364	4.123538	-1.316832
						1	4.887841	2.391371	-0.964902
						1	3.599606	2.981912	-2.039062
						1	1.602536	4.259764	-1.034468
						1	1.548088	4.551977	0.717491
						1	2.696962	5.446171	-0.292139
						1	4.827032	2.910893	1.507198
						1	4.628416	4.646208	1.198217
						1	3.445997	3.785609	2.202554
						8	-3.541973	-1.433912	1.795379
						1	-3.996280	-0.629796	1.439160
						8	-2.029316	-0.596843	3.987973
						1	-2.759230	-1.024654	3.498268
**TS2c**	6	-1.983369	2.459231	-0.518836	**INT1c**	6	-1.977271	2.243981	-0.870219
	6	-2.117069	2.095930	0.834349		6	-2.419169	2.224581	0.464116
	6	-2.662052	2.966248	1.762087		6	-3.045673	3.327342	1.024220
	6	-3.098238	4.223964	1.306879		6	-3.248051	4.455062	0.212020
	6	-2.966168	4.584923	-0.033792		6	-2.807209	4.474227	-1.113385
	6	-2.393903	3.702199	-0.967984		6	-2.149969	3.365232	-1.668968
	1	-2.764287	2.692439	2.808854		1	-3.381118	3.324520	2.057877
	1	-3.546268	4.921146	2.009179		1	-3.756997	5.323979	0.619769
	1	-3.312803	5.560381	-0.363126		1	-2.979056	5.356603	-1.723332
	1	-2.290838	3.992179	-2.010102		1	-1.804428	3.387804	-2.699247
	6	-1.302716	1.260312	-1.198543		6	-1.306998	0.888463	-1.108744
	6	0.140533	1.269135	-0.649722		6	0.038560	0.925084	-0.346646
	6	0.000000	0.897037	0.747495		6	-0.409234	0.953941	1.142927
	6	-1.525175	0.681659	0.938003		6	-1.975042	0.876916	1.018785
	1	-1.448527	1.153763	-2.270110		1	-1.310494	0.528274	-2.131773
	1	0.760151	2.119506	-0.911502		1	0.697411	1.768842	-0.657501
	1	0.530758	1.503603	1.473736		1	-0.137065	1.901517	1.628504
	1	-1.868794	0.059159	1.756985		1	-2.508922	0.482396	1.879546
	7	-1.860958	0.149382	-0.397494		7	-2.073729	0.025963	-0.186666
	6	-3.017376	-0.497574	-0.756583		6	-3.136207	-0.733494	-0.602194
	8	-3.390250	-0.587403	-1.920040		8	-3.247542	-1.149083	-1.750623
	8	-3.606564	-1.054978	0.320572		8	-3.964401	-0.998488	0.428777
	6	-4.812002	-1.897993	0.185231		6	-5.114121	-1.910289	0.274646
	6	-4.495586	-3.127884	-0.670901		6	-5.716356	-1.923128	1.681718
	1	-4.267156	-2.842561	-1.698137		1	-6.597499	-2.570953	1.705985
	1	-3.643647	-3.673984	-0.252868		1	-4.991065	-2.297494	2.410020
	1	-5.358301	-3.801638	-0.676046		1	-6.019144	-0.915621	1.981314
	6	-5.097455	-2.301032	1.633668		6	-6.106662	-1.329679	-0.737373
	1	-5.278140	-1.417264	2.252402		1	-6.397401	-0.315860	-0.444924
	1	-5.984365	-2.939877	1.676112		1	-5.672969	-1.298719	-1.737146
	1	-4.252952	-2.853284	2.056214		1	-7.009346	-1.948634	-0.761707
	6	-5.965209	-1.068403	-0.387168		6	-4.625776	-3.307152	-0.121174
	1	-6.884694	-1.662298	-0.372568		1	-4.180768	-3.299257	-1.116516
	1	-6.128547	-0.174052	0.222136		1	-3.884670	-3.669121	0.598587
	1	-5.758572	-0.764308	-1.413476		1	-5.469838	-4.004222	-0.117918
	6	1.005536	-0.714211	1.730540		44	1.675978	-0.298135	-0.824380
	6	2.044257	-1.246065	1.209529		6	1.593078	-2.435679	-1.394350
	44	1.318011	-0.534041	-0.631581		6	0.400843	-1.811114	-1.880758
	6	1.734274	-2.677949	-1.413935		6	0.799555	-0.883400	-2.888327
	6	0.325027	-2.458646	-1.481838		6	2.214412	-0.997173	-3.093157
	6	0.098038	-1.372941	-2.375114		6	2.699686	-1.950605	-2.182009
	6	1.363865	-0.893043	-2.836840		1	1.641753	-3.223998	-0.656831
	6	2.379071	-1.713301	-2.233190		1	-0.611301	-1.999533	-1.551833
	1	2.229888	-3.437340	-0.824223		1	0.140946	-0.243887	-3.459730
	1	-0.435498	-3.026835	-0.964145		1	2.806498	-0.421881	-3.792316
	1	-0.876498	-0.998732	-2.654875		1	3.736074	-2.230906	-2.044973
	1	1.534205	-0.098347	-3.549580		53	4.048528	0.631808	0.136131
	1	3.445179	-1.611152	-2.381220		6	0.258824	-0.180332	1.857204
	53	3.535598	1.211264	-0.369328		6	1.232523	-0.823118	1.184675
	6	0.395079	-0.619676	3.101960		6	-0.056218	-0.419189	3.326351
	1	-0.646085	-0.960854	3.091888		1	-1.047651	-0.020510	3.567900
	1	0.383585	0.434510	3.430758		1	-0.095281	-1.501471	3.529744
	6	3.271908	-1.965444	1.662152		6	2.098208	-1.860759	1.884705
	1	3.057344	-3.030849	1.801228		1	1.468267	-2.630705	2.348174

Species	Cartesian coordinates				Species	Cartesian coordinates			
	1	4.071405	-1.868157	0.920510		1	2.770609	-2.367031	1.184664
	8	1.078205	-1.451049	4.010193		8	0.856014	0.232295	4.203355
	1	2.039748	-1.341984	3.870604		1	1.718384	-0.175151	3.997670
	8	3.712884	-1.482995	2.938708		8	2.873488	-1.314254	2.968019
	1	4.059392	-0.585504	2.797634		1	3.485293	-0.670675	2.569833
**TS3c**	6	1.666329	2.155784	-0.027208	**INT2c**	6	-2.037369	2.097030	-1.237511
	6	2.137098	1.702324	-1.273821		6	-2.407359	2.146753	0.114459
	6	2.573044	2.596415	-2.239035		6	-3.321021	3.082155	0.577432
	6	2.552719	3.965794	-1.927445		6	-3.873098	3.976595	-0.353304
	6	2.083735	4.414745	-0.690885		6	-3.499610	3.933803	-1.699213
	6	1.617719	3.508732	0.274979		6	-2.565909	2.991806	-2.157578
	1	2.930539	2.255871	-3.206951		1	-3.607998	3.126497	1.624664
	1	2.909720	4.685824	-2.658244		1	-4.601495	4.711496	-0.022295
	1	2.083022	5.478588	-0.471617		1	-3.943900	4.633766	-2.401175
	1	1.249795	3.864141	1.233644		1	-2.281364	2.960472	-3.205938
	6	1.227376	0.895526	0.730533		6	-1.020447	0.974008	-1.352108
	6	-0.069353	0.491958	0.000031		6	-0.072447	1.628362	0.801146
	6	0.436007	-0.087651	-1.353805		6	-1.561999	1.096311	0.812253
	6	1.974654	0.183652	-1.249528		1	-0.950002	0.489551	-2.320350
	1	1.203814	0.935916	1.815750		1	-0.075782	2.703605	1.025859
	1	-0.774492	1.391473	-0.148495		1	-1.917068	0.712254	1.764817
	1	-0.002084	0.344990	-2.258188		7	-1.450435	0.044144	-0.240666
	1	2.611258	-0.405179	-1.905709		6	-2.500892	-0.864269	-0.470746
	7	2.173705	-0.101714	0.192793		8	-2.746964	-1.318988	-1.574346
	6	3.395250	-0.287967	0.808454		8	-3.105144	-1.176298	0.680928
	8	3.589113	-0.044110	1.990069		6	-4.238072	-2.133152	0.732805
	8	4.279118	-0.819298	-0.058955		6	-5.402277	-1.595282	-0.103015
	6	5.641937	-1.193270	0.370601		1	-5.153600	-1.576215	-1.164542
	6	6.254895	-1.744672	-0.918627		1	-6.277372	-2.236736	0.040445
	1	7.285501	-2.062676	-0.736234		1	-5.666186	-0.583215	0.218533
	1	5.685969	-2.605974	-1.280587		6	-3.771976	-3.515746	0.269814
	1	6.262388	-0.980375	-1.701203		1	-3.501147	-3.509074	-0.786541
	6	5.569950	-2.279315	1.448150		1	-2.912620	-3.847049	0.861851
	1	5.116086	-1.895753	2.362240		1	-4.579908	-4.239062	0.417946
	1	4.984890	-3.132431	1.090481		6	-4.588328	-2.142889	2.221687
	1	6.580067	-2.633357	1.677634		1	-4.880389	-1.143810	2.557321
	6	6.397778	0.052122	0.844050		1	-5.422987	-2.826174	2.402679
	1	6.384795	0.822803	0.066989		1	-3.733814	-2.473326	2.818958
	1	5.954445	0.458885	1.753185		44	1.123777	-0.652186	-0.489230
	1	7.441466	-0.208938	1.046353		6	1.673379	-2.971138	-0.642391
	6	0.022061	-1.479896	-1.015560		6	0.324409	-2.728145	-1.011654
	6	-0.696352	-1.392354	0.127036		6	0.298314	-1.984051	-2.256636
	44	-2.023818	0.321704	0.830753		6	1.619090	-1.662094	-2.575704
	6	-3.125258	-0.595829	2.500303		6	2.472799	-2.247286	-1.551400
	6	-1.876179	-0.088080	3.014844		1	2.016414	-3.534228	0.213778
	6	-1.874027	1.319908	2.830439		1	-0.547660	-3.113836	-0.499303
	6	-3.142445	1.697372	2.252324		1	-0.599668	-1.721378	-2.796024
	6	-3.912490	0.526396	2.076278		1	1.966174	-1.090788	-3.425382
	1	-3.451820	-1.627117	2.510258		1	3.551236	-2.183720	-1.534309
	1	-1.081809	-0.671146	3.461272		53	3.569124	0.759356	-0.611421
	1	-1.081738	2.000211	3.114027		6	0.320319	1.324064	-0.681459
	1	-3.442228	2.700579	1.979406		1	0.919812	2.083593	-1.175042
	1	-4.894808	0.474676	1.627432		6	0.722010	0.883034	1.848965
	53	-3.493716	0.048784	-1.503440		6	1.417239	-0.214212	1.478456
	6	0.416104	-2.751872	-1.733400		6	0.564953	1.472904	3.246075
	1	1.071415	-3.352543	-1.076948		1	-0.502376	1.657875	3.432944
	1	1.006761	-2.501973	-2.621364		1	1.034240	2.477176	3.216333
	6	-1.085948	-2.644562	0.894909		6	2.343842	-1.020105	2.360529
	1	-1.263881	-2.454534	1.955568		1	2.920134	-1.725646	1.755211
	1	-0.277796	-3.384219	0.831611		1	1.763467	-1.595393	3.090625
	8	-0.697957	-3.504438	-2.186123		8	3.251962	-0.220377	3.140537
	1	-1.328437	-3.587390	-1.447048		1	3.736909	0.344485	2.515163
	8	-2.222100	-3.276329	0.287092		8	1.030457	0.709158	4.329261
	1	-2.856846	-2.577066	0.047985		1	1.974429	0.521493	4.153416
**TS4c**	6	2.003955	-1.300856	-1.897735	**INT3c**	6	2.139875	2.688485	0.806003
	6	2.420627	-1.731014	-0.626900		6	2.230618	2.074199	-0.465273
	6	3.585166	-2.468283	-0.482818		6	3.457804	2.040339	-1.130295
	6	4.341909	-2.764544	-1.628111		6	4.607259	2.571392	-0.536851
	6	3.939465	-2.319145	-2.890211		6	4.532319	3.130754	0.739801
	6	2.764813	-1.572985	-3.036517		6	3.306215	3.191136	1.401975

Species	Cartesian coordinates				Species	Cartesian coordinates			
	1	3.917605	-2.795388	0.498387		1	3.516442	1.586111	-2.114514
	1	5.262335	-3.333006	-1.528719		1	5.553500	2.543562	-1.069457
	1	4.545844	-2.545713	-3.762031		1	5.423253	3.535388	1.212304
	1	2.442906	-1.231279	-4.016552		1	3.238720	3.656990	2.382076
	6	0.703578	-0.620946	-1.877840		6	0.825938	2.872831	1.424687
	6	0.061868	-1.899665	0.133256		6	-0.294333	2.744616	0.699592
	6	1.437833	-1.223178	0.404180		6	-0.229643	2.372122	-0.771862
	1	0.475293	0.046404	-2.706794		6	0.965090	1.470403	-1.083075
	1	0.193709	-2.977750	-0.036787		1	0.787109	3.189960	2.463948
	1	1.770528	-1.349938	1.434489		1	-1.272402	2.959782	1.115012
	7	1.172945	0.201940	0.053530		1	0.012527	3.308499	-1.302223
	6	2.236328	1.082689	0.204999		1	1.097942	1.417514	-2.171581
	8	2.330133	2.172557	-0.346905		7	0.755640	0.067824	-0.627838
	8	3.176199	0.578085	1.039551		6	1.938093	-0.719385	-0.863193
	6	4.433884	1.299072	1.322266		8	2.480608	-0.785195	-1.950844
	6	5.236743	1.480812	0.030296		8	2.321784	-1.335702	0.255201
	1	4.728341	2.156516	-0.657623		6	3.591793	-2.113531	0.331654
	1	6.221147	1.896372	0.268955		6	3.589668	-2.590970	1.784333
	1	5.384426	0.515513	-0.463563		1	2.719809	-3.221815	1.989502
	6	4.132614	2.630220	2.017793		1	4.493141	-3.174325	1.984042
	1	3.617745	3.317251	1.346224		1	3.565256	-1.739652	2.469568
	1	3.512203	2.464026	2.904148		6	3.567223	-3.295720	-0.641136
	1	5.070601	3.091995	2.342708		1	4.476490	-3.888470	-0.499731
	6	5.151888	0.341418	2.276978		1	2.713974	-3.952644	-0.448709
	1	5.332867	-0.622500	1.792812		1	3.533464	-2.956912	-1.676856
	1	6.115220	0.764269	2.576990		6	4.774660	-1.180736	0.064610
	1	4.553224	0.170838	3.176401		1	4.785016	-0.838164	-0.970461
	44	-1.070099	0.737619	-0.275611		1	4.735436	-0.309346	0.723021
	6	-1.759406	2.907721	0.410986		1	5.706154	-1.720836	0.261697
	6	-0.396166	2.893178	0.047222		44	-0.876386	-0.960980	-0.338339
	6	-0.298288	2.672313	-1.390319		6	-2.376097	-2.655993	-0.741146
	6	-1.589071	2.464723	-1.875994		6	-1.531975	-2.436981	-1.868984
	6	-2.495705	2.576254	-0.753711		6	-0.203221	-2.853577	-1.499895
	1	-2.165630	3.094670	1.394413		6	-0.242539	-3.353882	-0.173698
	1	0.442556	3.106334	0.691126		6	-1.563587	-3.187921	0.306908
	1	0.630797	2.660000	-1.937420		1	-3.441319	-2.489318	-0.681521
	1	-1.881260	2.247559	-2.894216		1	-1.834748	-2.088983	-2.847017
	1	-3.570395	2.493331	-0.813782		1	0.663090	-2.811740	-2.146860
	53	-3.676938	-0.440806	-0.794020		1	0.596211	-3.715580	0.402238
	6	-0.410403	-1.159576	-1.125038		1	-1.906983	-3.422103	1.305161
	1	-1.224599	-1.575175	-1.712836		53	-2.150174	-0.194201	1.971812
	6	-0.796934	-1.632041	1.352035		6	-1.554272	1.884311	-1.324392
	6	-1.348505	-0.410395	1.434531		6	-1.982533	0.610937	-1.239385
	6	-0.806504	-2.777741	2.354201		6	-2.467841	3.006339	-1.830852
	1	0.225158	-3.144741	2.463422		1	-1.863943	3.880902	-2.093412
	1	-1.366648	-3.615557	1.892463		1	-2.992127	2.698848	-2.748335
	6	-2.226363	0.080223	2.567509		6	-3.411445	0.303332	-1.668195
	1	-2.687476	1.037537	2.311064		1	-3.641677	0.804957	-2.614394
	1	-1.630992	0.226321	3.475576		1	-3.585938	-0.760201	-1.836117
	8	-3.263859	-0.848120	2.941185		8	-3.388612	3.445751	-0.838567
	1	-3.755727	-1.056297	2.128328		1	-3.936556	2.663385	-0.636321
	8	-1.266091	-2.493893	3.652221		8	-4.389277	0.802170	-0.734846
	1	-2.159308	-2.106273	3.553247		1	-4.121577	0.494002	0.149166
**TS5c**	6	0.012601	3.298283	1.081186	**INT4c**	6	-1.939275	2.377096	-0.699991
	6	-0.748602	2.319450	0.408746		6	-2.603133	2.057748	0.499411
	6	-1.419431	2.669562	-0.762782		6	-3.684887	2.809436	0.932095
	6	-1.345419	3.968539	-1.272685		6	-4.108349	3.884660	0.132966
	6	-0.601651	4.940889	-0.602863		6	-3.443646	4.202494	-1.053483
	6	0.071918	4.602280	0.569039		6	-2.333870	3.454544	-1.478963
	1	-2.006519	1.921161	-1.283369		1	-4.203153	2.572800	1.857626
	1	-1.873383	4.219193	-2.188765		1	-4.967795	4.474176	0.440049
	1	-0.543953	5.953656	-0.991884		1	-3.793926	5.033501	-1.659378
	1	0.669493	5.346973	1.089738		1	-1.819845	3.705881	-2.403048
	6	0.780008	2.929486	2.267561		6	-0.798072	1.358946	-0.816269
	6	0.883726	1.666232	2.703499		6	0.234749	1.817335	0.260721
	6	0.120629	0.525816	2.094085		6	-0.460067	1.459596	1.599756
	6	-0.944139	0.935890	1.030766		6	-1.838723	0.879911	1.096934
	1	1.314631	3.724743	2.783123		1	-0.441141	1.140387	-1.823283
	1	1.482504	1.435771	3.580274		1	0.464262	2.875779	0.166424
	1	-0.439429	0.075764	2.929611		1	-0.654457	2.348328	2.214230

Species	Cartesian coordinates				Species	Cartesian coordinates			
	1	-1.901840	0.993309	1.559509		1	-2.348298	0.226912	1.800732
	7	-1.038858	-0.146906	0.054759		7	-1.384306	0.188641	-0.133089
	6	-2.236809	-0.668119	-0.332060		6	-2.153684	-0.703861	-0.856155
	8	-2.366282	-1.661673	-1.050732		8	-2.038323	-0.861207	-2.062408
	8	-3.299183	0.040146	0.160794		8	-2.979182	-1.380751	-0.033969
	6	-4.685657	-0.357172	-0.115607		6	-3.834351	-2.471845	-0.533821
	6	-4.964458	-1.750414	0.459779		6	-4.550560	-2.937162	0.736595
	1	-4.380924	-2.509875	-0.060594		1	-5.223567	-3.768042	0.504760
	1	-4.715249	-1.776739	1.525864		1	-3.827531	-3.271881	1.485729
	1	-6.028618	-1.986635	0.356313		1	-5.140646	-2.123043	1.167664
	6	-4.970886	-0.284127	-1.619995		6	-4.836472	-1.923556	-1.555044
	1	-4.377954	-1.019100	-2.163989		1	-5.400823	-1.090810	-1.123481
	1	-6.033151	-0.476790	-1.803102		1	-4.328273	-1.577135	-2.455225
	1	-4.737651	0.714993	-2.001871		1	-5.547061	-2.709952	-1.829657
	6	-5.491244	0.707066	0.636082		6	-2.967211	-3.594105	-1.112614
	1	-5.251445	1.706441	0.261878		1	-2.435350	-3.258796	-2.003324
	1	-6.563165	0.532998	0.502128		1	-2.237531	-3.931576	-0.370155
	1	-5.267172	0.677782	1.706638		1	-3.601571	-4.445996	-1.378588
	44	0.855161	-1.109790	-0.592961		44	1.939178	0.625133	-0.232533
	6	1.621356	-3.179001	-1.166777		6	4.117443	0.820791	-1.170979
	6	0.267365	-3.034001	-1.631509		6	3.429570	1.866753	-1.801704
	6	0.256632	-2.036715	-2.655416		6	2.866055	2.694230	-0.780675
	6	1.562275	-1.526397	-2.778179		6	3.285174	2.212908	0.497562
	6	2.418515	-2.217523	-1.835203		6	4.020480	1.004031	0.263982
	1	1.964004	-3.877751	-0.414482		1	4.608634	-0.008541	-1.661846
	1	-0.594562	-3.603507	-1.319185		1	3.283349	1.984710	-2.867337
	1	-0.625729	-1.689626	-3.172710		1	2.270518	3.580230	-0.956155
	1	1.876140	-0.726276	-3.432799		1	3.095394	2.670961	1.457918
	1	3.479528	-2.056238	-1.710609		1	4.526413	0.410058	1.012642
	53	2.461459	1.197062	-0.923386		53	1.829970	-1.957335	-1.075521
	6	0.985819	-0.675470	1.588131		6	0.369233	0.459617	2.339632
	6	0.268086	-1.769677	1.108943		6	1.430164	-0.064349	1.695179
	6	2.258294	-0.949232	2.364067		6	-0.111178	0.000878	3.706380
	1	2.881707	-0.048323	2.355717		1	0.742459	-0.115380	4.393113
	1	2.820047	-1.768577	1.903318		1	-0.766886	0.762659	4.143746
	6	-0.351568	-2.968502	1.749570		6	2.231065	-1.180227	2.349609
	1	-1.301722	-2.621874	2.197939		1	3.147777	-1.404318	1.795488
	1	-0.602261	-3.746782	1.027008		1	2.523789	-0.892471	3.367187
	8	1.872525	-1.292999	3.713334		8	-0.878726	-1.199377	3.646894
	1	2.673927	-1.437627	4.241460		1	-0.269387	-1.854958	3.257879
	8	0.533540	-3.534928	2.695015		8	1.463803	-2.387925	2.513220
	1	0.861388	-2.829497	3.291703		1	1.259246	-2.702224	1.615332
**TS6c**	6	-2.037849	2.427596	-0.568916	**INT5c**	6	2.671493	-1.723030	-0.835767
	6	-2.284195	2.084276	0.774641		6	2.728620	-1.414591	0.533641
	6	-3.128845	2.840936	1.567963		6	3.877281	-1.657365	1.274172
	6	-3.753078	3.959728	0.985703		6	4.983450	-2.211067	0.611786
	6	-3.509187	4.301733	-0.344145		6	4.924828	-2.523431	-0.749514
	6	-2.633596	3.539254	-1.139374		6	3.758536	-2.285088	-1.491388
	1	-3.320925	2.580626	2.605751		1	3.924438	-1.427255	2.334964
	1	-4.436721	4.560771	1.578673		1	5.899497	-2.398376	1.164985
	1	-4.006489	5.166330	-0.775031		1	5.796324	-2.946655	-1.240817
	1	-2.448782	3.812850	-2.174676		1	3.719954	-2.522466	-2.551285
	6	-1.022696	1.387068	-1.073481		6	1.287648	-1.314365	-1.305725
	6	0.268522	1.757247	-0.311028		6	0.131714	-2.150832	-0.738776
	6	0.025968	1.352062	1.064829		6	0.402456	-2.197129	0.776167
	6	-1.436448	0.826025	1.036820		6	1.342910	-0.933761	0.910203
	1	-0.952538	1.208715	-2.142193		1	1.206273	-1.014407	-2.346961
	1	0.640905	2.759446	-0.502305		1	-0.138798	-3.090161	-1.217061
	1	0.294795	2.020575	1.876506		1	0.979015	-3.085074	1.067226
	1	-1.769010	0.169093	1.831964		1	1.216545	-0.347938	1.815878
	7	-1.447440	0.203932	-0.298173		7	0.941833	-0.178542	-0.353462
	6	-2.465370	-0.605018	-0.758888		6	1.585884	1.077933	-0.649211
	8	-2.792768	-0.677420	-1.931054		8	1.714086	1.464013	-1.792356
	8	-2.988904	-1.319859	0.265274		8	1.972693	1.676643	0.466559
	6	-4.054031	-2.309974	0.029408		6	2.700703	2.977283	0.445615
	6	-3.540495	-3.422387	-0.889919		6	2.899345	3.258781	1.935108
	1	-3.324104	-3.037159	-1.886374		1	3.443085	4.199131	2.063858
	1	-2.628281	-3.865537	-0.478364		1	1.935194	3.341547	2.443797
	1	-4.296543	-4.210346	-0.969908		1	3.475240	2.458174	2.407959
	6	-4.324462	-2.848977	1.436639		6	4.041393	2.780093	-0.265801

Species	Cartesian coordinates				Species	Cartesian coordinates			
	1	-4.650050	-2.045033	2.103547		1	4.604352	1.963831	0.196838
	1	-5.110188	-3.609362	1.402870		1	3.898896	2.558049	-1.324354
	1	-3.422278	-3.303393	1.856964		1	4.633983	3.696168	-0.179342
	6	-5.300426	-1.617871	-0.532725		6	1.836285	4.054910	-0.210306
	1	-5.607615	-0.797252	0.123433		1	1.688748	3.854955	-1.271857
	1	-5.111329	-1.220682	-1.529989		1	0.859584	4.110591	0.277035
	1	-6.124831	-2.336195	-0.587999		1	2.334627	5.023719	-0.101716
	44	2.001256	0.527528	0.032887		44	-1.279579	-0.481445	-0.900036
	6	0.893511	-0.379415	1.919777		6	-2.457839	0.640553	-2.642856
	6	1.647933	-1.165302	1.249088		6	-1.419855	-0.023407	-3.314381
	6	4.066168	0.599188	-0.975445		6	-1.515311	-1.410540	-2.987451
	6	3.415201	1.871233	-1.103705		6	-2.686095	-1.620144	-2.178897
	6	3.249895	2.397472	0.215148		6	-3.250535	-0.340203	-1.928148
	6	3.786629	1.450200	1.145739		1	-2.633901	1.707134	-2.636743
	6	4.311442	0.353556	0.400243		1	-0.638089	0.442333	-3.899282
	1	4.318409	-0.061941	-1.792343		1	-0.864934	-2.190228	-3.360946
	1	3.141220	2.351190	-2.032789		1	-3.069908	-2.573067	-1.842382
	1	2.819346	3.357059	0.471179		1	-4.154697	-0.132449	-1.373858
	1	3.815936	1.559817	2.222027		53	-1.817934	1.900503	0.548168
	1	4.789098	-0.525659	0.810661		6	-0.896802	-2.122301	1.519541
	53	1.259724	-1.199527	-2.071248		6	-1.857398	-1.415027	0.897636
	6	0.365777	-0.329786	3.333296		6	-1.017268	-2.710921	2.912857
	1	-0.656547	-0.741427	3.361157		1	-1.975125	-3.247637	3.011812
	1	0.302161	0.703256	3.690489		1	-0.222539	-3.446302	3.081633
	8	1.214477	-1.023957	4.220693		6	-3.220619	-1.220670	1.538112
	1	1.361762	-1.912402	3.841660		1	-3.919143	-0.722113	0.858835
	6	2.238772	-2.522634	1.380431		1	-3.654230	-2.194747	1.798482
	1	2.137895	-3.071769	0.437501		8	-0.882526	-1.731229	3.940968
	1	3.308843	-2.442720	1.601348		1	-1.637297	-1.130792	3.791059
	8	1.664575	-3.257842	2.473839		8	-3.164242	-0.494249	2.781015
	1	0.793443	-3.577337	2.189983		1	-2.872973	0.405237	2.545959
**TS7c**	6	1.407669	2.377298	-0.269727	**INT6c**	6	-3.172743	-2.006181	0.116028
	6	2.098050	1.915118	-1.407068		6	-2.155649	-1.689361	-0.813964
	6	2.720096	2.802301	-2.272499		6	-2.433232	-1.725249	-2.182492
	6	2.662486	4.172271	-1.968697		6	-3.703252	-2.077749	-2.644681
	6	1.975284	4.629783	-0.841018		6	-4.710255	-2.393867	-1.730141
	6	1.321516	3.732842	0.017789		6	-4.446046	-2.354830	-0.361499
	1	3.250904	2.455333	-3.155254		1	-1.647377	-1.475702	-2.890788
	1	3.163863	4.886047	-2.616463		1	-3.903002	-2.106478	-3.711749
	1	1.952207	5.693791	-0.622598		1	-5.699547	-2.672192	-2.082653
	1	0.783420	4.092099	0.890698		1	-5.227867	-2.606323	0.351293
	6	0.813268	1.125525	0.389253		6	-2.877367	-1.960917	1.550249
	6	-0.325642	0.775306	-0.596899		6	-1.613763	-1.920485	1.996058
	6	0.416172	0.122100	-1.788626		6	-0.451780	-2.004298	1.025705
	6	1.915859	0.401168	-1.412416		6	-0.769780	-1.302577	-0.319245
	1	0.573552	1.165048	1.446391		1	-3.715329	-1.971963	2.243738
	1	-0.968788	1.683617	-0.793898		1	-1.401696	-1.881511	3.060883
	1	0.167942	0.537247	-2.773121		1	-0.375038	-3.068459	0.736974
	1	2.661827	-0.192889	-1.934087		1	-0.021889	-1.655587	-1.037384
	7	1.829044	0.118310	0.036395		7	-0.539539	0.159804	-0.236019
	6	2.885776	-0.160546	0.870246		6	-1.655874	1.047705	-0.435081
	8	2.878231	0.062873	2.070705		8	-1.846745	1.603390	-1.497003
	8	3.880064	-0.758603	0.171744		8	-2.363605	1.192052	0.683523
	6	5.091552	-1.253096	0.847136		6	-3.578542	2.056103	0.733230
	6	5.897563	-1.847698	-0.310806		6	-4.057632	1.857695	2.171487
	1	6.841071	-2.258556	0.060481		1	-3.293686	2.181226	2.884489
	1	5.337921	-2.651215	-0.798654		1	-4.963981	2.444962	2.345163
	1	6.124412	-1.081039	-1.057677		1	-4.280989	0.804840	2.361067
	6	4.719463	-2.337974	1.863087		6	-3.182999	3.512924	0.480055
	1	4.128184	-1.922410	2.679339		1	-4.058742	4.153522	0.623909
	1	4.143757	-3.131611	1.376459		1	-2.413781	3.836862	1.188317
	1	5.631089	-2.782108	2.275992		1	-2.818283	3.652036	-0.538521
	6	5.847395	-0.085874	1.490961		6	-4.621215	1.559655	-0.270570
	1	6.054822	0.688931	0.746031		1	-4.278980	1.690927	-1.297329
	1	5.269699	0.352307	2.304919		1	-4.844218	0.502644	-0.106416
	1	6.804122	-0.442050	1.886676		1	-5.544792	2.131477	-0.134649
	6	0.043439	-1.326901	-1.599585		44	1.173811	0.964953	0.083326
	6	-0.935660	-1.500311	-0.693253		6	2.050165	2.387690	1.670594
	44	-2.311391	0.162804	-0.329728		6	2.884430	2.476213	0.530466
	6	-4.088153	-1.189067	-0.607242		6	2.096297	3.006803	-0.544793

Species	Cartesian coordinates				Species	Cartesian coordinates			
	6	-4.610690	0.110348	-0.306799		6	0.798048	3.308936	-0.042876
	6	-4.178949	0.981594	-1.331029		6	0.753069	2.891888	1.313484
	6	-3.452535	0.225654	-2.327589		1	2.333881	2.027017	2.649659
	6	-3.422511	-1.112418	-1.886342		1	3.926416	2.196867	0.466958
	1	-4.257385	-2.084808	-0.026911		1	2.442822	3.179206	-1.554725
	1	-5.165320	0.385674	0.578723		1	-0.023783	3.715472	-0.616355
	1	-4.345789	2.051946	-1.359807		1	-0.099387	2.953883	1.976884
	1	-3.043204	0.612615	-3.250456		53	2.612026	-0.364474	-1.845971
	1	-2.941475	-1.933146	-2.399552		6	0.899807	-1.686848	1.631423
	53	-2.171264	0.455997	2.398234		6	1.733530	-0.683678	1.299997
	6	0.838858	-2.392779	-2.333090		6	1.351871	-2.798933	2.590301
	1	1.791879	-2.568357	-1.805527		1	1.982057	-2.386994	3.390446
	1	1.101684	-1.989485	-3.320910		1	0.475207	-3.242949	3.073922
	6	-1.336386	-2.769230	0.012261		6	3.182341	-0.723318	1.772587
	1	-2.194351	-3.246655	-0.488713		1	3.723931	0.179473	1.484817
	1	-1.652944	-2.511160	1.028429		1	3.237079	-0.797576	2.865335
	8	0.146953	-3.604736	-2.565795		8	2.007488	-3.859261	1.910783
	1	0.037354	-4.003459	-1.682174		1	2.817368	-3.445699	1.556727
	8	-0.236650	-3.694295	0.073936		8	3.896755	-1.870147	1.278456
	1	-0.447876	-4.379502	0.725507		1	3.897623	-1.790591	0.308227
**TS8c**	6	2.420713	1.991082	0.882914	**INT8c**	6	1.745539	0.123848	1.958745
	6	2.425299	1.696766	-0.499456		6	1.182166	-1.123799	1.364927
	6	3.566083	1.919001	-1.253056		6	-0.034511	-1.492509	2.251617
	6	4.712294	2.424706	-0.620189		6	-0.554085	0.931103	1.597972
	6	4.722068	2.685703	0.754296		6	0.858586	1.217103	2.039686
	6	3.578469	2.455946	1.521382		7	-0.641696	0.531731	0.162410
	1	3.581524	1.681039	-2.312574		1	1.917064	-1.929261	1.310863
	1	5.613399	2.593801	-1.203053		1	-1.195498	1.799236	1.762910
	1	5.624737	3.059466	1.227763		6	1.273703	2.433299	2.575858
	1	3.575751	2.662843	2.588183		1	0.580056	3.269576	2.624933
	6	1.142267	1.781798	1.539457		6	2.577016	2.569044	3.063323
	6	0.039535	2.231363	-0.604753		1	2.902012	3.513864	3.489616
	6	1.089510	1.101486	-0.891040		6	3.456151	1.484581	3.006569
	1	1.159399	1.606981	2.614122		1	4.467504	1.587173	3.390756
	1	0.490039	3.191955	-0.902721		6	3.047246	0.270824	2.451259
	1	1.051872	0.720137	-1.914211		1	3.741079	-0.563697	2.393064
	7	0.777404	0.047352	0.117642		6	-1.013136	-0.284396	2.452697
	6	1.574839	-1.067005	0.268165		6	-2.106135	-1.223164	1.976124
	8	1.485990	-1.837030	1.217008		6	-1.271516	-2.270237	1.806165
	8	2.466634	-1.179722	-0.746020		1	-1.105120	0.052457	3.493121
	6	3.447007	-2.279926	-0.787102		1	0.371145	-1.854853	3.209602
	6	4.384748	-2.188462	0.421451		6	-3.581269	-1.026212	1.778247
	1	3.843142	-2.372581	1.349552		1	-4.154381	-1.475782	2.598629
	1	5.181837	-2.932838	0.324184		1	-3.823636	0.045729	1.774227
	1	4.847610	-1.197582	0.469261		6	-1.485206	-3.719703	1.478768
	6	2.722674	-3.627330	-0.864859		1	-1.690596	-4.259300	2.421368
	1	2.164089	-3.824952	0.049704		1	-0.560427	-4.155442	1.081378
	1	2.026998	-3.635816	-1.709065		1	-1.473277	-1.027173	-2.963165
	1	3.455793	-4.426879	-1.015380		6	-0.599270	-1.535203	-2.583285
	6	4.207701	-1.998885	-2.085793		6	-0.588564	-2.470776	-1.506676
	1	4.693435	-1.019598	-2.045469		6	0.739270	-1.351061	-3.013104
	1	4.976717	-2.761314	-2.242022		6	0.769005	-2.894254	-1.300399
	1	3.526577	-2.013833	-2.941564		1	-1.447000	-2.855730	-0.969500
	44	-1.185951	0.164645	0.994488		44	0.606201	-0.686523	-0.746022
	6	-2.931460	-0.620636	2.166303		6	1.581798	-2.211421	-2.232885
	6	-1.818809	-1.465337	2.563948		1	1.079632	-0.678037	-3.788263
	6	-0.888125	-0.677710	3.263172		1	1.106851	-3.626081	-0.580199
	6	-1.370648	0.663262	3.250432		53	3.007114	0.640925	-1.146740
	6	-2.661632	0.694010	2.615524		1	2.653971	-2.300090	-2.328998
	1	-3.828476	-0.962098	1.669859		1	-3.898750	3.468364	-2.918979
	1	-1.726392	-2.520520	2.350339		6	-3.131695	2.824752	-2.477072
	1	0.067730	-1.014096	3.638955		6	-3.152317	2.988832	-0.955649
	1	-0.887517	1.514871	3.712967		1	-2.160738	3.128813	-2.879511
	1	-3.306733	1.556554	2.521624		1	-3.332040	1.793678	-2.769119
	53	-1.688788	-1.979806	-0.875258		8	-1.994979	2.269567	-0.359427
	6	-0.109846	2.124409	0.908541		6	-2.869597	4.438502	-0.558037
	1	-0.738097	2.851874	1.423155		6	-4.455029	2.490387	-0.325187
	6	-1.334782	2.081301	-1.214451		6	-1.830282	0.949664	-0.481703
	6	-2.153574	1.242183	-0.559828		1	-1.911812	4.771312	-0.967021
	6	-1.574267	2.907077	-2.469768		1	-3.658550	5.089963	-0.944735

Species	Cartesian coordinates				Species	Cartesian coordinates			
	1	-0.858671	2.553366	-3.240641		1	-2.837081	4.543703	0.530194
	1	-1.279721	3.946418	-2.258902		1	-4.652762	1.448878	-0.580953
	6	-3.605663	0.989294	-0.922694		1	-4.417438	2.592493	0.764157
	1	-4.172631	1.923862	-0.861356		1	-5.288011	3.099719	-0.689491
	1	-4.061982	0.270920	-0.236607		8	-2.597005	0.202499	-1.081458
	8	-3.789767	0.528997	-2.274066		8	-2.512890	-3.972191	0.529201
	1	-3.263795	-0.287318	-2.354292		1	-3.193078	-3.270550	0.602107
	8	-2.887920	2.958605	-2.967653		8	-4.058994	-1.651883	0.580192
	1	-3.216782	2.037881	-3.023875		1	-3.613405	-1.192278	-0.157492
**TS9c**	6	3.426964	-1.861352	0.051375	**INT9c**	6	1.690919	-0.834179	2.131366
	6	2.729649	-0.685597	-0.299812		6	0.490814	-1.642198	1.924471
	6	3.007076	-0.074819	-1.524751		6	1.583665	0.565034	1.998324
	6	3.943504	-0.622026	-2.406968		6	0.261175	1.096810	1.490733
	6	4.623523	-1.793454	-2.066100		7	0.074928	0.741687	0.072847
	6	4.366273	-2.404383	-0.840459		1	0.606161	-2.722725	1.913449
	1	2.486823	0.841824	-1.782406		1	0.212436	2.180254	1.616791
	1	4.146402	-0.128614	-3.353601		6	2.685318	1.369384	2.272707
	1	5.353917	-2.222411	-2.746229		1	2.606098	2.446138	2.150104
	1	4.894489	-3.313659	-0.562656		6	3.888507	0.793184	2.693979
	6	3.158423	-2.500749	1.340170		1	4.743279	1.427128	2.912805
	6	2.180697	-2.089983	2.161566		6	3.997547	-0.593732	2.824095
	6	1.275055	-0.931923	1.842394		1	4.935248	-1.039261	3.142946
	6	1.779128	-0.014655	0.692912		6	2.905142	-1.408507	2.530883
	1	3.790878	-3.340002	1.623253		1	2.986133	-2.488553	2.618830
	1	2.014671	-2.592009	3.111014		44	-0.783985	-1.121482	-0.198914
	1	1.209851	-0.306756	2.743774		6	-0.910909	0.411261	2.278824
	1	2.370470	0.776761	1.164360		1	-0.844719	0.669049	3.345365
	7	0.620012	0.617256	0.060346		6	-2.200355	0.813830	1.633694
	6	0.528848	1.975772	-0.023406		6	-2.384174	0.286070	0.411124
	8	-0.484249	2.605619	-0.303391		6	-0.770484	-1.083606	2.058436
	8	1.742679	2.582214	0.218893		1	-1.614711	-1.708116	2.335067
	6	1.869406	4.043247	0.244261		6	-0.829319	-3.248960	-1.101710
	6	0.971028	4.643887	1.331941		6	-0.936265	-2.348613	-2.239000
	1	-0.081967	4.505330	1.087771		6	-1.990107	-3.100351	-0.314381
	1	1.177506	4.170865	2.297688		6	-2.113167	-1.609512	-2.096063
	1	1.175955	5.715319	1.428930		6	-2.770054	-2.059713	-0.886254
	6	1.567238	4.625580	-1.141409		1	-0.024336	-3.948912	-0.934087
	1	0.525993	4.453369	-1.413274		1	-0.214837	-2.257252	-3.036403
	1	1.763980	5.702975	-1.141288		1	-2.225518	-3.645441	0.589903
	1	2.213160	4.163537	-1.895400		1	-2.464816	-0.851099	-2.778986
	6	3.346754	4.242171	0.599398		1	-3.730347	-1.712150	-0.526921
	1	3.989806	3.765225	-0.146206		53	2.088983	-1.665745	-1.222916
	1	3.586548	5.309376	0.632897		6	-3.114175	1.840528	2.260529
	1	3.572108	3.806538	1.577600		1	-2.722203	2.134818	3.243209
	44	-0.957888	-0.850270	-0.387648		1	-3.173990	2.749970	1.642805
	6	-2.106897	-2.268414	-1.771060		6	-3.529429	0.750681	-0.468420
	6	-1.302354	-1.426054	-2.636916		1	-3.343021	0.568246	-1.524915
	6	0.051835	-1.697505	-2.376848		1	-3.612694	1.844577	-0.359150
	6	0.122291	-2.649451	-1.315525		6	0.131225	1.665187	-0.922145
	6	-1.227262	-3.038789	-0.983285		8	-0.274820	1.468290	-2.066627
	1	-3.186184	-2.304670	-1.756880		8	0.713765	2.832006	-0.507542
	1	-1.687611	-0.714264	-3.352554		6	0.961535	3.933595	-1.443292
	1	0.899569	-1.201600	-2.826459		8	-4.430706	1.268929	2.408875
	1	1.028343	-3.067631	-0.902218		1	-5.038357	1.972001	2.687097
	1	-1.512481	-3.789582	-0.259606		8	-4.773248	0.129265	-0.144432
	53	-3.231441	0.807142	-0.825178		1	-4.921964	0.357094	0.793459
	6	-0.202158	-1.365597	1.581436		6	1.642560	4.980617	-0.556096
	6	-1.172388	-0.362118	1.458928		1	2.569791	4.582254	-0.133629
	6	-0.675144	-2.618535	2.303957		1	1.884546	5.872523	-1.142028
	1	-0.001598	-3.457765	2.086568		1	0.985731	5.276612	0.267825
	1	-1.688867	-2.884495	1.983909		6	-0.364065	4.474710	-1.991744
	6	-1.800806	0.584429	2.398671		1	-0.863163	3.724431	-2.604813
	1	-1.712198	1.592561	1.972444		1	-1.027274	4.759457	-1.167933
	1	-2.884892	0.367540	2.399093		1	-0.177290	5.366222	-2.599734
	8	-0.652934	-2.302792	3.706796		6	1.907999	3.478130	-2.559692
	1	-0.980166	-3.064660	4.209922		1	2.825854	3.063970	-2.131178
	8	-1.228306	0.568872	3.688734		1	1.437034	2.716366	-3.180735
	1	-1.172758	-0.359920	3.977012		1	2.178285	4.334474	-3.186709
**TS11c**	6	-1.651501	1.102872	-1.915853	**INT10c**	6	1.210532	2.005731	1.571754
	6	-0.216375	0.989265	-1.833801		6	1.429074	0.539949	1.374417

Species	Cartesian coordinates				Species	Cartesian coordinates			
	6	0.475901	2.295717	-1.652697		6	0.520724	-0.176115	2.407032
	6	-2.232813	1.917781	-0.907743		6	-1.144006	1.359790	1.209586
	6	-1.225080	2.219706	0.195425		6	-0.126429	2.451789	1.471101
	7	-0.597730	0.943717	0.568664		7	-0.856184	0.691569	-0.075250
	1	0.292641	0.111710	-2.225457		1	2.475640	0.258384	1.497344
	1	-1.697032	2.703386	1.057613		1	-2.156428	1.764869	1.185791
	6	-3.590339	2.193179	-0.942835		6	-0.436723	3.799766	1.620006
	1	-4.049225	2.795203	-0.163848		1	-1.469720	4.130496	1.538628
	6	-4.376313	1.644975	-1.968320		6	0.582216	4.729510	1.867826
	1	-5.441883	1.855898	-1.995701		1	0.340936	5.782759	1.979216
	6	-3.816742	0.796154	-2.929311		6	1.905709	4.297367	1.972005
	1	-4.446354	0.359075	-3.698278		1	2.696426	5.015651	2.171796
	6	-2.453254	0.501785	-2.898768		6	2.219762	2.941298	1.827635
	1	-2.004485	-0.149037	-3.642843		1	3.251104	2.608454	1.920808
	44	1.159430	-0.976139	0.572182		6	-0.957837	0.351247	2.368869
	6	-0.143094	3.096621	-0.451409		6	-1.374217	-1.098048	2.229948
	6	1.284665	3.127441	0.088503		6	-0.107678	-1.564035	2.277914
	6	1.812748	2.470795	-0.964080		1	-1.283534	0.832289	3.300380
	1	-0.556138	4.073612	-0.725332		1	0.989085	-0.021043	3.392080
	1	0.379637	2.822137	-2.619031		6	-2.735282	-1.732479	2.208972
	6	1.781582	3.776310	1.344517		1	-3.025799	-1.967382	3.251339
	1	1.763843	4.872543	1.194001		1	-3.464158	-1.000592	1.844386
	1	1.063305	3.570495	2.148689		6	0.438919	-2.956545	2.409937
	6	3.156900	2.105219	-1.491600		1	0.255759	-3.332856	3.424555
	1	3.339610	2.683051	-2.412848		1	1.520407	-2.969195	2.244187
	1	3.163296	1.041651	-1.772706		1	1.258260	3.112073	-0.846510
	6	2.093720	-0.955119	2.562339		6	1.443156	2.269832	-1.495621
	6	2.763337	0.011483	1.758391		6	0.590409	1.817495	-2.560456
	6	2.257321	-2.243000	1.935689		6	2.633045	1.472867	-1.522805
	6	3.376675	-0.669031	0.648558		6	1.237460	0.774213	-3.244645
	6	3.067247	-2.053984	0.764499		1	-0.400698	2.191574	-2.773864
	1	1.538549	-0.755909	3.469291		44	0.969772	0.233483	-0.756680
	1	2.809097	1.078467	1.940710		6	2.474787	0.529199	-2.589234
	1	1.871745	-3.187453	2.295179		1	3.509539	1.604344	-0.903719
	1	3.993719	-0.208557	-0.107424		1	0.821147	0.188632	-4.052627
	1	3.356276	-2.830686	0.070018		53	2.370053	-2.110054	-0.530818
	53	0.535361	-2.581150	-1.689578		1	3.192475	-0.229909	-2.864552
	6	-1.358253	-0.000689	1.127091		1	-5.346627	0.796884	-3.028543
	8	-0.903665	-1.167310	1.290321		6	-4.406369	1.024985	-2.516204
	8	-2.595385	0.352582	1.527796		6	-4.310147	0.199391	-1.229705
	6	-3.504218	-0.578981	2.225797		1	-4.395307	2.095793	-2.287967
	6	-3.856888	-1.750648	1.304868		1	-3.578241	0.793374	-3.186894
	6	-2.877297	-1.036754	3.546301		8	-3.098622	0.607741	-0.480317
	6	-4.733223	0.296264	2.486436		6	-5.436121	0.571719	-0.261861
	1	-4.628515	-2.367230	1.777611		6	-4.298580	-1.309142	-1.499648
	1	-4.251206	-1.378911	0.354192		6	-1.863333	0.342835	-0.952081
	1	-2.983467	-2.371062	1.103514		1	-5.388708	1.633278	-0.001244
	1	-3.606359	-1.626740	4.111089		1	-6.406171	0.373264	-0.726880
	1	-1.992298	-1.648350	3.369747		1	-5.371273	-0.016171	0.658141
	1	-2.597048	-0.171008	4.155026		1	-3.577651	-1.565948	-2.275528
	1	-5.154205	0.658967	1.544451		1	-4.043499	-1.866900	-0.593261
	1	-5.500681	-0.282095	3.009149		1	-5.295986	-1.618500	-1.829725
	1	-4.469710	1.159646	3.104738		8	-1.632817	-0.170052	-2.047817
	8	4.167207	2.379150	-0.520633		8	-0.217063	-3.893791	1.540653
	1	5.040644	2.339187	-0.936147		1	0.055056	-3.669749	0.634219
	8	3.049798	3.345852	1.799212		8	-2.847898	-2.878476	1.382120
	1	3.609385	3.170680	1.020027		1	-2.014757	-3.384332	1.456891
**TS12c**	6	1.012327	-1.028293	2.214126	**INT11c**	6	-2.244207	-2.436257	0.355968
	6	-0.039261	-1.769012	1.469328		6	-0.923156	-2.836930	0.743477
	6	0.947790	0.379060	2.243432		6	-2.385915	-1.553346	-0.750938
	6	-0.186606	1.014519	1.462846		6	-1.121182	-0.879492	-1.261635
	7	-0.072369	0.803545	0.010141		7	-0.694753	0.027003	-0.175260
	1	0.036653	-2.853696	1.506229		1	-0.809796	-3.437275	1.643996
	1	-0.247757	2.082506	1.673052		1	-1.312290	-0.342280	-2.196296
	6	1.877218	1.101973	2.984185		6	-3.666828	-1.270068	-1.225423
	1	1.825096	2.187998	2.995426		1	-3.783978	-0.601551	-2.071239
	6	2.873024	0.437389	3.708441		6	-4.786859	-1.842028	-0.620035
	1	3.595795	1.006748	4.286026		1	-5.776457	-1.633068	-1.017223
	6	2.934000	-0.956820	3.686972		6	-4.652001	-2.677944	0.499314
	1	3.705987	-1.477033	4.247223		1	-5.533027	-3.100935	0.972065

Species	Cartesian coordinates				Species	Cartesian coordinates			
	6	2.008204	-1.688685	2.940778		6	-3.387747	-2.966147	0.991188
	1	2.062356	-2.774019	2.916209		1	-3.264888	-3.625775	1.846640
	44	-0.345618	-1.146644	-0.639782		44	0.975817	-0.556855	0.870167
	6	-1.480246	0.302184	1.918699		6	-0.038079	-1.964936	-1.439154
	1	-1.696365	0.497367	2.976260		1	-0.445895	-2.720606	-2.131890
	6	-2.655750	0.509130	1.017312		6	1.338912	-1.514600	-1.890025
	6	-2.608364	-0.557963	0.196120		6	2.096299	-0.994882	-0.910762
	6	-1.351029	-1.195905	1.614186		6	0.172070	-2.593775	-0.070015
	1	-2.143682	-1.839581	1.988703		1	1.056038	-3.210163	0.057749
	6	0.483571	-2.061161	-2.689958		6	0.413433	-1.094969	3.066226
	6	-0.564200	-1.175955	-3.042829		6	0.788994	0.253786	3.131812
	6	-0.039003	-3.098268	-1.833080		6	1.567203	-1.857390	2.612858
	6	-1.734037	-1.641742	-2.412230		6	2.171440	0.335559	2.745038
	6	-1.412946	-2.850031	-1.667845		6	2.658136	-0.968416	2.458346
	1	1.498147	-1.998578	-3.047305		1	-0.559734	-1.497421	3.310043
	1	-0.462304	-0.266035	-3.614019		1	0.144685	1.090282	3.356884
	1	0.526521	-3.924555	-1.426504		1	1.615350	-2.933111	2.505650
	1	-2.709129	-1.197973	-2.537183		1	2.754921	1.243419	2.710996
	1	-2.102941	-3.480607	-1.124436		1	3.668072	-1.230476	2.177356
	53	2.628460	-1.043093	-0.683671		53	1.848279	2.126960	0.017054
	6	-3.811180	1.438603	1.275787		6	1.676732	-1.798380	-3.346646
	1	-3.435288	2.422764	1.575872		1	1.027772	-1.149585	-3.969440
	1	-4.398853	1.577317	0.355761		6	3.574803	-0.663877	-1.022125
	6	-3.860457	-1.192078	-0.345250		1	4.138606	-1.576781	-1.241026
	1	-3.685458	-2.141907	-0.851861		1	3.951263	-0.247168	-0.084270
	1	-4.315568	-0.502127	-1.075360		6	-1.478753	1.076082	0.216841
	6	0.066650	1.831037	-0.860672		8	-1.372574	1.656584	1.294206
	8	0.054811	1.707587	-2.087975		8	-2.374932	1.432765	-0.751320
	8	0.228773	3.040849	-0.233247		6	-3.274310	2.580245	-0.565572
	6	0.444978	4.271686	-0.995703		1	1.362304	-2.827613	-3.575440
	6	1.739788	4.172893	-1.810746		6	-2.460824	3.869265	-0.403122
	6	-0.772592	4.577698	-1.876655		1	-1.877582	3.847907	0.516841
	6	0.585602	5.327912	0.105812		1	-1.777455	3.994764	-1.248519
	1	1.952965	5.136668	-2.285586		1	-3.137954	4.729878	-0.379019
	1	2.579609	3.921153	-1.155640		6	-4.060349	2.612803	-1.880412
	1	1.654503	3.406384	-2.580653		1	-4.648750	1.699334	-2.008562
	1	-0.890480	3.817695	-2.648718		1	-4.747166	3.464587	-1.885679
	1	-1.682067	4.609221	-1.267271		1	-3.381214	2.711124	-2.732322
	1	-0.649688	5.556345	-2.352767		6	-4.217030	2.336556	0.618800
	1	1.429640	5.091125	0.760392		1	-3.662488	2.310657	1.556683
	1	0.755820	6.314705	-0.335523		1	-4.961413	3.138436	0.667686
	1	-0.321913	5.374124	0.715776		1	-4.746260	1.386543	0.493900
	8	-4.759120	-1.411713	0.759513		8	3.889134	0.235787	-2.098379
	1	-5.567074	-1.830227	0.421900		1	3.402088	1.058634	-1.910323
	8	-4.617063	0.966144	2.350968		8	3.026279	-1.702490	-3.726383
	1	-4.913240	0.083666	2.065554		1	3.365838	-0.844365	-3.398677
**TS13c**	6	2.188365	2.118582	0.806750					
	6	0.751574	2.135473	0.969459					
	6	0.013158	2.871945	-0.099813					
	6	2.631032	1.788572	-0.501274					
	6	1.478144	1.273977	-1.352606					
	7	0.793660	0.262476	-0.540856					
	1	0.329984	2.054295	1.963320					
	1	1.822536	0.861794	-2.306195					
	6	3.982243	1.832555	-0.803630					
	1	4.332156	1.560709	-1.795245					
	6	4.898869	2.191082	0.197601					
	1	5.960048	2.216503	-0.034046					
	6	4.470493	2.481041	1.497001					
	1	5.196265	2.741120	2.261358					
	6	3.112839	2.429730	1.815182					
	1	2.770155	2.668198	2.818407					
	44	-0.798993	-0.577565	1.260688					
	6	0.512046	2.454191	-1.525613					
	6	-0.958564	2.199416	-1.818674					
	6	-1.394212	2.574568	-0.601039					
	1	0.983366	3.258559	-2.101042					
	1	0.189804	3.938469	0.134282					
	6	-0.558857	0.073169	3.384793					
	6	-0.772553	-1.335487	3.311753					

Species	Cartesian coordinates	Species	Cartesian coordinates
	6 -1.728096 0.713401 2.827328		
	6 -2.054775 -1.570191 2.718063		
	6 -2.653640 -0.287740 2.437996		
	1 0.301783 0.563214 3.820682		
	1 -0.070515 -2.100228 3.617570		
	1 -1.891357 1.780289 2.747231		
	1 -2.502613 -2.535582 2.529160		
	1 -3.623942 -0.125760 1.990504		
	53 -2.261653 -1.667598 -0.920348		
	6 1.394100 -0.905505 -0.329545		
	8 0.927412 -1.694834 0.542876		
	8 2.474132 -1.198585 -1.075028		
	6 3.164277 -2.504092 -1.006202		
	6 3.761718 -2.701078 0.390477		
	6 2.204920 -3.630059 -1.399756		
	6 4.271247 -2.341927 -2.051140		
	1 4.382825 -3.602640 0.398340		
	1 4.394690 -1.848589 0.656463		
	1 2.978215 -2.808225 1.140972		
	1 2.755612 -4.574918 -1.452681		
	1 1.396805 -3.732639 -0.675277		
	1 1.769151 -3.433232 -2.383803		
	1 4.929059 -1.507277 -1.791212		
	1 4.873045 -3.253904 -2.103278		
	1 3.843662 -2.152816 -3.039987		
	6 -1.634020 1.693471 -3.055089		
	1 -1.674580 2.483050 -3.817178		
	1 -1.042052 0.869721 -3.480053		
	6 -2.756743 2.753374 0.007717		
	1 -2.704218 3.525487 0.787280		
	1 -3.054364 1.817112 0.507270		
	8 -3.733904 3.172975 -0.924433		
	1 -3.710845 2.521362 -1.654091		
	8 -2.977256 1.280289 -2.824580		
	1 -2.931442 0.444614 -2.313688		

Table S7.The optimized cartesian coordinates (Å) for reaction **d**, located at IDSCRF-B3LYP/BS1 level in dioxane solvent.

Species	Cartesian coordinates	Species	Cartesian coordinates
**R2d**	6 -1.203056 0.075558 0.001961	**P1d**	6 2.336025 3.699603 0.208612
	6 -0.012363 0.286317 0.010297		6 1.102257 3.630699 0.852186
	6 -2.640083 -0.181791 -0.003180		6 0.155677 2.661592 0.488007
	1 -2.947172 -0.695600 0.914422		6 1.705050 1.814514 -1.163225
	1 -3.213072 0.749669 -0.069583		6 2.629433 2.804005 -0.820795
	6 1.425288 0.550897 -0.001503		1 3.054929 4.463127 0.492098
	1 1.591728 1.627775 -0.169147		1 0.847100 4.354965 1.621745
	1 1.881259 0.024215 -0.855921		1 1.940542 1.105470 -1.949918
	8 2.004769 0.126948 1.232596		1 3.574231 2.870071 -1.352506
	1 2.955059 0.310395 1.181212		6 0.484401 1.706281 -0.497614
	1 -2.929319 -0.814187 -0.849827		6 -1.207272 2.710685 1.018851
			6 -1.995263 1.128600 -0.758822
			6 -2.212196 2.069043 0.409775
			1 -1.400159 3.365693 1.865429
			1 -3.233827 2.191000 0.760295
			6 -0.528785 0.641365 -0.880697
			6 -2.759170 -0.156945 -0.493695
			6 -1.919789 -1.087533 -0.008806
			1 -2.317264 1.613309 -1.692050
			1 -0.340085 0.330280 -1.915475
			7 -0.561781 -0.618944 -0.047608
			6 0.507153 -1.418749 0.271079
			8 1.664025 -0.966147 -0.242450
			8 0.409301 -2.440879 0.953649
			6 2.944276 -1.644166 0.080003
			6 -4.241543 -0.223689 -0.684562
			1 -4.507269 0.038048 -1.716857
			1 -4.649465 -1.215314 -0.479261
			6 -2.303641 -2.445711 0.524538
			1 -2.028711 -2.506658 1.584918

Species	Cartesian coordinates	Species	Cartesian coordinates
			1 -3.391009 -2.527725 0.462557
			6 2.930193 -3.081596 -0.450460
			1 3.932627 -3.510663 -0.353034
			1 2.226392 -3.703997 0.100022
			1 2.660923 -3.091463 -1.511310
			6 3.977499 -0.812353 -0.682523
			1 3.770649 -0.824282 -1.756773
			1 3.976698 0.224029 -0.338434
			1 4.974444 -1.233023 -0.521993
			6 3.205188 -1.572325 1.587203
			1 2.473924 -2.157731 2.144160
			1 4.204920 -1.963810 1.800728
			1 3.167046 -0.533516 1.928790
			8 -1.767998 -3.538533 -0.206138
			1 -0.845355 -3.595791 0.095918
			1 -4.767944 0.489814 -0.036777
**P2d**	6 0.374653 2.146734 -0.595699	**TS14d**	6 2.685960 -2.068799 0.306521
	6 0.783913 0.781395 -1.148699		6 1.289070 -2.500747 0.139995
	6 2.182527 0.494822 -0.523233		6 2.938853 -0.908774 1.073550
	6 0.277139 0.440604 1.008721		6 1.717529 -0.081784 1.423565
	6 0.059928 1.933741 0.760924		7 1.091085 0.361660 0.171721
	7 -0.063542 -0.110851 -0.324954		1 1.152518 -3.456060 -0.360204
	6 -1.316909 -0.484439 -0.738643		1 1.986219 0.770115 2.051579
	8 -1.675565 -0.485472 -1.907215		6 4.247902 -0.544032 1.366968
	8 -2.056987 -0.897519 0.320616		1 4.438649 0.352486 1.949526
	6 -3.419845 -1.420357 0.136014		6 5.313797 -1.316019 0.887517
	1 0.680357 0.603122 -2.215714		1 6.335171 -1.021687 1.111011
	1 -0.274951 -0.020155 1.824710		6 5.069413 -2.450282 0.110450
	6 -0.305649 2.987582 1.583619		1 5.898675 -3.043146 -0.263758
	1 -0.544241 2.830402 2.632356		6 3.757649 -2.828668 -0.180099
	6 -0.378149 4.273310 1.022461		1 3.563124 -3.726135 -0.762240
	1 -0.685732 5.111804 1.641065		44 -0.165959 -1.026335 -0.671689
	6 -0.067192 4.484779 -0.321282		6 0.688053 -1.005809 2.137529
	1 -0.136089 5.486198 -0.736932		1 1.052697 -1.280632 3.135654
	6 0.326683 3.417956 -1.145859		6 -0.739185 -0.531073 2.141722
	1 0.571508 3.590297 -2.190535		6 -1.299556 -1.312586 1.166300
	6 1.822987 0.241583 0.996034		6 0.381021 -2.213949 1.238691
	6 2.366157 -1.164096 0.769898		1 -0.072778 -3.089708 1.703734
	6 2.683143 -0.938988 -0.519036		6 0.352735 -0.857864 -2.868117
	1 2.328139 0.861905 1.742721		6 -0.793160 -0.068545 -2.577964
	1 2.904774 1.285377 -0.747887		6 0.008362 -2.221524 -2.611158
	6 2.458077 -2.329592 1.695624		6 -1.860294 -0.959385 -2.184012
	1 1.470900 -2.584974 2.100650		6 -1.364757 -2.287309 -2.216360
	1 2.856856 -3.215788 1.192819		1 1.319015 -0.482165 -3.171669
	6 3.304428 -1.742346 -1.610499		1 -0.844991 1.008720 -2.630555
	1 3.368700 -2.800002 -1.308528		1 0.674152 -3.067201 -2.723508
	1 4.338184 -1.392508 -1.778112		1 -2.850025 -0.632653 -1.882066
	6 -3.387325 -2.682996 -0.731275		1 -1.916688 -3.186713 -1.978015
	1 -4.386633 -3.128796 -0.768506		53 -4.408374 1.238847 0.045906
	1 -3.065471 -2.450571 -1.746351		6 -1.364182 0.487215 3.028707
	1 -2.701804 -3.420425 -0.301952		1 -0.615778 0.985665 3.649728
	6 -3.842637 -1.758392 1.568050		1 -1.895259 1.235788 2.428010
	1 -3.823617 -0.863950 2.197570		6 -2.687870 -1.927493 1.085913
	1 -4.858458 -2.164389 1.574242		1 -2.562244 -3.013594 1.207320
	1 -3.169604 -2.502466 2.003950		1 -3.133077 -1.754829 0.101996
	6 -4.324425 -0.332664 -0.452484		6 1.579360 1.469636 -0.476317
	1 -5.359627 -0.688312 -0.475925		8 1.264105 1.802840 -1.615376
	1 -4.287752 0.567629 0.168893		8 2.446939 2.168628 0.305507
	1 -4.016151 -0.074873 -1.465643		6 3.022936 3.448868 -0.142155
	8 2.523884 -1.576159 -2.794127		6 3.902998 3.223819 -1.376257
	1 2.994482 -2.001591 -3.525682		6 1.910482 4.471396 -0.394589
	1 3.105030 -2.107680 2.553899		6 3.875668 3.866957 1.059250
			1 4.429330 4.150921 -1.625698
			1 4.651623 2.451620 -1.171811
			1 3.302627 2.917100 -2.232557
			1 1.287334 4.174061 -1.237756
			1 1.278625 4.573370 0.493243
			1 2.354365 5.448885 -0.609465
			1 4.649365 3.120734 1.264345
			1 4.365871 4.823674 0.856454

Species	Cartesian coordinates				Species	Cartesian coordinates			
						1	3.255634	3.979981	1.953469
						8	-3.525453	-1.462933	2.110214
						1	-3.956465	-0.650937	1.759035
						1	-2.120697	0.012782	3.662796
**TS2d**	6	-1.913528	2.402761	-0.620784	**INT1d**	6	-1.903790	2.352876	-0.670202
	6	-2.084593	2.127566	0.748871		6	-2.363268	2.262677	0.655402
	6	-2.651167	3.057641	1.603019		6	-2.981383	3.340329	1.271046
	6	-3.069653	4.284536	1.056490		6	-3.156637	4.516874	0.524280
	6	-2.899558	4.558337	-0.300495		6	-2.698233	4.606404	-0.792151
	6	-2.306138	3.615696	-1.159703		6	-2.050235	3.521414	-1.403620
	1	-2.782717	2.852403	2.662214		1	-3.332313	3.281727	2.297991
	1	-3.533778	5.026534	1.700042		1	-3.658707	5.368020	0.975790
	1	-3.232894	5.511489	-0.701209		1	-2.849289	5.525293	-1.351672
	1	-2.173454	3.837707	-2.215118		1	-1.691262	3.598905	-2.426652
	6	-1.220575	1.161333	-1.203733		6	-1.246403	1.005083	-0.977165
	6	0.209030	1.201557	-0.621322		6	0.089940	0.987063	-0.192773
	6	0.033197	0.919323	0.799101		6	-0.383479	0.913100	1.288383
	6	-1.498851	0.723480	0.959704		6	-1.950107	0.877712	1.135697
	6	0.983697	-0.576721	1.944639		1	-1.237680	0.703954	-2.019380
	6	2.014733	-1.165355	1.470213		1	0.736897	1.859158	-0.431153
	44	1.372376	-0.590927	-0.447182		1	-0.102711	1.815829	1.849788
	1	-1.340847	0.987236	-2.269645		1	-2.503979	0.442096	1.962950
	1	0.830983	2.038355	-0.919163		7	-2.040394	0.105243	-0.119484
	1	0.535821	1.589346	1.489537		6	-3.053632	-0.684851	-0.580835
	1	-1.864208	0.156686	1.808891		8	-3.126910	-1.064036	-1.746397
	7	-1.802878	0.105475	-0.347078		8	-3.889204	-1.024321	0.423882
	6	-2.951082	-0.561226	-0.692905		6	-4.990113	-1.982393	0.214413
	8	-3.299172	-0.723080	-1.856233		6	-5.616781	-2.075340	1.607894
	8	-3.566910	-1.051801	0.403446		1	-6.466563	-2.764047	1.591534
	6	-4.776255	-1.890780	0.291084		1	-4.887746	-2.442017	2.336417
	6	-4.453159	-3.173339	-0.481385		1	-5.972232	-1.094717	1.937457
	1	-4.197604	-2.952299	-1.517989		6	-5.991198	-1.412712	-0.795512
	1	-3.616707	-3.700672	-0.011108		1	-6.336260	-0.425620	-0.472178
	1	-5.322346	-3.838632	-0.466725		1	-5.540616	-1.323397	-1.784238
	6	-5.099061	-2.203229	1.754180		1	-6.862188	-2.072784	-0.861123
	1	-5.285071	-1.282106	2.314090		6	-4.429694	-3.340031	-0.221021
	1	-5.992847	-2.830981	1.814460		1	-3.969093	-3.275633	-1.207145
	1	-4.269736	-2.736026	2.228759		1	-3.683933	-3.692011	0.498869
	6	-5.909005	-1.088326	-0.356575		1	-5.239556	-4.075727	-0.257361
	1	-6.834213	-1.672750	-0.327176		44	1.716161	-0.234114	-0.725724
	1	-6.077215	-0.157313	0.193644		6	1.568337	-2.317421	-1.492966
	1	-5.676448	-0.848610	-1.394286		6	0.433855	-1.594719	-1.976427
	6	1.770770	-2.797731	-1.069256		6	0.929136	-0.590329	-2.859085
	6	0.373460	-2.551706	-1.224961		6	2.349635	-0.756623	-3.003642
	6	0.220406	-1.520123	-2.194542		6	2.739476	-1.817553	-2.171064
	6	1.519602	-1.097306	-2.616231		1	1.541002	-3.170451	-0.830505
	6	2.481770	-1.900369	-1.908994		1	-0.603125	-1.767845	-1.723759
	1	2.215005	-3.531632	-0.411004		1	0.332688	0.135206	-3.395564
	1	-0.427604	-3.069992	-0.716144		1	3.004700	-0.145886	-3.610565
	1	-0.728988	-1.144324	-2.548091		1	3.752671	-2.161935	-2.008175
	1	1.746873	-0.353006	-3.366425		53	4.009853	0.613207	0.462901
	1	3.556232	-1.831631	-2.006691		6	0.230203	-0.298535	1.923890
	53	3.584993	1.165983	-0.268380		6	1.182482	-0.929841	1.214697
	6	0.260893	-0.443067	3.236920		6	-0.220541	-0.670182	3.318795
	1	-0.680868	-1.003128	3.247604		1	-1.235055	-1.091957	3.312753
	1	0.041406	0.596339	3.504235		1	0.455246	-1.386507	3.785054
	6	3.182082	-1.954824	1.966798		6	1.967861	-2.110579	1.775330
	1	2.992577	-3.024772	1.819245		1	1.283249	-2.940044	2.000486
	1	4.071397	-1.692982	1.378612		1	2.689180	-2.482534	1.037404
	8	3.407456	-1.784689	3.360911		8	2.639619	-1.831839	3.003476
	1	3.729846	-0.879338	3.490993		1	3.160712	-1.025779	2.858582
	1	0.915322	-0.872720	4.002351		1	-0.255201	0.220070	3.961003
**TS3d**	6	1.584619	2.018388	0.583408	**INT2d**	6	-1.726474	2.402810	-0.600456
	6	2.068814	1.988639	-0.738033		6	-2.238416	2.278467	0.703918
	6	2.483487	3.149724	-1.371332		6	-2.898154	3.330031	1.320705
	6	2.428197	4.352424	-0.647964		6	-3.062119	4.521764	0.595590
	6	1.946999	4.380885	0.662751		6	-2.551826	4.647586	-0.698389
	6	1.502933	3.207445	1.292802		6	-1.863048	3.587353	-1.309425
	1	2.851564	3.137310	-2.393655		1	-3.290354	3.240388	2.330537
	1	2.767960	5.273016	-1.113886		1	-3.595470	5.354372	1.045714

Species	Cartesian coordinates				Species	Cartesian coordinates			
	1	1.919484	5.322409	1.203750		1	-2.693699	5.577548	-1.242022
	1	1.125264	3.236546	2.311272		1	-1.462262	3.695213	-2.313733
	6	1.175392	0.575205	0.903344		6	-1.021298	1.078193	-0.898033
	6	-0.107594	0.384490	0.063573		6	-0.303207	0.935478	1.407501
	6	0.424015	0.257022	-1.389524		6	-1.843509	0.879623	1.166800
	6	1.950630	0.534723	-1.189867		1	-0.914413	0.802487	-1.943325
	6	0.072414	-1.192049	-1.496099		1	-0.061513	1.733571	2.120926
	6	-0.682058	-1.482022	-0.409208		1	-2.449771	0.425240	1.946571
	44	-2.058389	-0.043196	0.753687		7	-1.862058	0.140860	-0.117002
	1	1.144175	0.273524	1.946499		6	-2.911022	-0.547289	-0.661966
	1	-0.827444	1.282999	0.184655		8	-2.990006	-0.822301	-1.854169
	1	-0.029615	0.922423	-2.130151		8	-3.778124	-0.934618	0.303026
	1	2.608350	0.198531	-1.987788		6	-4.959084	-1.758292	-0.015531
	7	2.152676	-0.179809	0.094391		44	1.488600	-0.692519	-0.107214
	6	3.369271	-0.543335	0.630527		6	1.976735	-3.000017	-0.520441
	8	3.557063	-0.680496	1.830245		6	0.572775	-2.702121	-0.655786
	8	4.261146	-0.776310	-0.355055		6	0.387316	-1.873480	-1.811474
	6	5.619978	-1.269106	-0.055774		6	1.667422	-1.548342	-2.298811
	6	6.243175	-1.386305	-1.448777		6	2.650597	-2.260331	-1.499417
	1	7.272194	-1.748730	-1.369185		1	2.428272	-3.625609	0.237045
	1	5.676656	-2.087348	-2.068728		1	-0.223488	-3.125344	-0.057779
	1	6.256928	-0.413798	-1.949513		1	-0.568423	-1.534548	-2.188397
	6	5.540257	-2.640136	0.622463		1	1.892821	-0.910943	-3.142745
	1	5.078383	-2.564148	1.607150		1	3.721656	-2.209921	-1.637992
	1	4.958341	-3.335189	0.008926		53	3.666486	1.039663	-0.536648
	1	6.548546	-3.050877	0.736509		6	0.259638	1.094599	-0.028730
	6	6.373586	-0.239995	0.792532		1	0.860340	1.985509	-0.199495
	1	6.366720	0.737091	0.299355		6	0.334866	-0.405400	1.794264
	1	5.923593	-0.140636	1.780477		6	1.732422	-0.504192	1.769851
	1	7.415617	-0.554795	0.908678		6	-0.485467	-1.460662	2.513530
	6	-3.038935	-1.474833	2.147245		1	-0.807115	-1.073799	3.490675
	6	-1.863496	-0.942156	2.787241		1	0.086073	-2.376922	2.683945
	6	-2.045909	0.462259	2.918180		6	2.802921	-0.658634	2.779374
	6	-3.352279	0.797177	2.398898		1	2.444191	-1.326066	3.577138
	6	-3.961023	-0.401229	1.952463		1	2.915731	0.338015	3.251496
	1	-3.197864	-2.503004	1.857804		6	-5.619876	-1.933558	1.354082
	1	-1.001647	-1.507594	3.115408		1	-6.527803	-2.536081	1.257770
	1	-1.347460	1.158970	3.363573		1	-4.942568	-2.438077	2.049619
	1	-3.785754	1.787178	2.355380		1	-5.892198	-0.962931	1.778699
	1	-4.925641	-0.478936	1.469896		6	-4.518428	-3.110792	-0.583937
	53	-3.479877	0.369788	-1.605518		1	-5.392694	-3.756961	-0.712627
	6	0.626862	-2.097004	-2.553650		1	-4.026422	-2.987981	-1.548975
	1	1.716657	-2.190095	-2.458508		1	-3.829997	-3.608214	0.107138
	1	0.426626	-1.670669	-3.544283		6	-5.883922	-0.997305	-0.970911
	6	-0.958612	-2.896694	0.045141		1	-6.812328	-1.560934	-1.108337
	1	-0.704001	-3.012422	1.104652		1	-6.138960	-0.018533	-0.552761
	1	-0.289975	-3.572074	-0.510226		1	-5.411258	-0.854173	-1.942743
	8	-2.306598	-3.330579	-0.114360		1	-1.386143	-1.716851	1.948388
	1	-2.707308	-2.801511	-0.822014		8	4.019041	-1.178127	2.311529
	1	0.196258	-3.099660	-2.525249		1	4.289678	-0.638318	1.547087
**TS4d**	6	-1.946713	1.951908	-1.166413	**INT3d**	6	-2.596750	-2.338736	0.706023
	6	-2.395927	1.845549	0.160340		6	-2.511884	-1.730720	-0.568464
	6	-3.574374	2.460236	0.553090		6	-3.678420	-1.477733	-1.292436
	6	-4.311980	3.178186	-0.402195		6	-4.932449	-1.788769	-0.757926
	6	-3.876611	3.267052	-1.727147		6	-5.022827	-2.345833	0.518942
	6	-2.687474	2.645641	-2.125062		6	-3.862123	-2.619873	1.242555
	1	-3.931762	2.373863	1.575442		1	-3.605273	-1.022185	-2.275170
	1	-5.242830	3.656482	-0.110571		1	-5.831032	-1.591735	-1.335470
	1	-4.468159	3.814367	-2.454943		1	-5.994508	-2.580332	0.945090
	1	-2.340021	2.718773	-3.152115		1	-3.928698	-3.080452	2.225240
	6	-0.634977	1.327500	-1.381942		6	-1.366371	-2.733298	1.394648
	6	-0.055274	1.718540	0.986206		6	-0.206313	-2.814056	0.726699
	6	-1.425657	0.980141	0.932291		6	-0.140303	-2.480450	-0.753467
	7	-1.130899	-0.188528	0.054148		6	-1.128971	-1.373023	-1.119888
	44	1.130553	-0.533220	-0.406002		1	-1.430886	-3.016928	2.442294
	6	0.453513	1.539530	-0.450868		1	0.699012	-3.158083	1.215049
	6	0.787817	1.000953	2.020663		1	-0.546119	-3.365459	-1.273556
	6	1.361635	-0.145485	1.618831		1	-1.194581	-1.300093	-2.213482
	1	-0.379129	1.041996	-2.400623		7	-0.671838	-0.043209	-0.634434
	1	-0.202901	2.773976	1.253942		6	-1.692876	0.953312	-0.807361

Species	Cartesian coordinates				Species	Cartesian coordinates			
	1	-1.781996	0.687372	1.920023		8	-2.221619	1.184179	-1.879387
	6	-2.182831	-1.060783	-0.188973		8	-1.951106	1.556524	0.354006
	8	-2.248284	-1.837808	-1.134175		6	-3.047155	2.552905	0.501392
	8	-3.149175	-0.940312	0.754088		6	-2.958121	2.912503	1.985285
	6	-4.402847	-1.716720	0.685995		1	-1.981421	3.342603	2.225051
	6	-5.171741	-1.364182	-0.591380		1	-3.730487	3.645125	2.236912
	1	-4.637414	-1.703700	-1.478775		1	-3.103771	2.024441	2.605856
	1	-6.155956	-1.843265	-0.567956		6	-2.785425	3.776380	-0.380804
	1	-5.320244	-0.282077	-0.658276		1	-3.553799	4.530162	-0.181875
	6	-4.101280	-3.214760	0.793715		1	-1.814377	4.225792	-0.152022
	1	-3.559859	-3.570494	-0.082837		1	-2.822601	3.517205	-1.439468
	1	-3.505600	-3.419128	1.689081		6	-4.387430	1.889171	0.178291
	1	-5.040544	-3.771011	0.877705		1	-4.458607	1.630573	-0.878508
	6	-5.157865	-1.227520	1.924998		1	-4.520639	0.981038	0.771895
	1	-5.338797	-0.150438	1.866029		1	-5.197850	2.583227	0.422959
	1	-6.122932	-1.737099	2.001327		44	1.127450	0.652699	-0.355703
	1	-4.584645	-1.433689	2.833584		6	2.904735	1.967600	-0.948925
	6	1.832175	-2.796668	-0.595141		6	1.869098	2.013983	-1.933613
	6	0.480335	-2.646756	-0.972224		6	0.752059	2.721893	-1.360742
	6	0.421549	-1.887293	-2.214599		6	1.100562	3.115816	-0.047571
	6	1.722236	-1.500807	-2.535591		6	2.405325	2.617525	0.215747
	6	2.596891	-2.034327	-1.512544		1	3.867705	1.486546	-1.041184
	1	2.210971	-3.347139	0.253608		1	1.930557	1.658246	-2.952816
	1	-0.373795	-3.095639	-0.490056		1	-0.178612	2.928320	-1.871147
	1	-0.490869	-1.666756	-2.745338		1	0.468592	3.637619	0.657289
	1	2.041347	-0.903256	-3.378446		1	2.935235	2.709964	1.153615
	1	3.671100	-1.928534	-1.498238		53	2.241246	-0.356552	1.947207
	53	3.719392	0.773807	-0.354620		6	1.267785	-2.282090	-1.294118
	1	1.271117	2.160332	-0.807390		6	1.907347	-1.098219	-1.288319
	6	0.744826	1.573705	3.418195		6	1.839186	-3.595252	-1.810733
	1	-0.289751	1.632338	3.786589		1	1.626157	-4.392100	-1.086728
	1	1.133024	2.601662	3.424740		1	1.357942	-3.896206	-2.751512
	6	2.200196	-1.069169	2.481051		6	3.278748	-0.963182	-1.925336
	1	2.807184	-1.719320	1.840095		1	3.512788	-1.866260	-2.503010
	1	1.543595	-1.724479	3.070090		1	3.287045	-0.141963	-2.650883
	8	3.041248	-0.413023	3.430865		8	4.354068	-0.696333	-1.019411
	1	3.592152	0.205798	2.925460		1	4.104858	-1.056933	-0.154511
	1	1.336864	0.980972	4.113742		1	2.917086	-3.585215	-1.970844
**TS5d**	6	0.672444	3.281658	0.875527	**INT4d**	6	-2.059322	2.267512	-0.664526
	6	-0.201192	2.405271	0.200729		6	-2.671900	1.984982	0.570332
	6	-0.669293	2.753583	-1.065692		6	-3.750936	2.733504	1.015030
	6	-0.282586	3.953625	-1.668219		6	-4.225559	3.767963	0.191288
	6	0.574410	4.827675	-0.997893		6	-3.612488	4.049476	-1.031536
	6	1.046608	4.489001	0.268862		6	-2.505095	3.305183	-1.469527
	1	-1.342802	2.080004	-1.584411		1	-4.229175	2.525474	1.968663
	1	-0.655610	4.205825	-2.657254		1	-5.084090	4.353768	0.507848
	1	0.875393	5.764058	-1.459451		1	-4.001609	4.849013	-1.655806
	1	1.729737	5.153758	0.792601		1	-2.031399	3.528680	-2.421893
	6	1.230159	2.888930	2.166206		6	-0.901349	1.270224	-0.780600
	6	1.030186	1.679939	2.709467		6	0.159623	1.782460	0.242832
	6	0.100100	0.655803	2.121189		6	-0.481363	1.464050	1.619839
	6	-0.739715	1.161370	0.904960		6	-1.862951	0.844620	1.183198
	1	1.873160	3.606631	2.671571		1	-0.571913	1.019041	-1.789185
	1	1.502197	1.432168	3.656254		1	0.362402	2.841868	0.106315
	1	-0.625493	0.423169	2.918877		1	-0.678615	2.376577	2.199239
	1	-1.707871	1.467920	1.314823		1	-2.356510	0.215724	1.919779
	7	-0.974219	0.031738	0.010559		7	-1.439951	0.113868	-0.034523
	6	-2.231293	-0.323813	-0.355952		6	-2.211666	-0.825471	-0.690988
	8	-2.533410	-1.403718	-0.880514		8	-2.141155	-1.029236	-1.893368
	8	-3.154688	0.653720	-0.093756		8	-2.985104	-1.492192	0.190972
	6	-4.578267	0.478015	-0.399032		6	-3.822817	-2.627880	-0.234573
	6	-5.158285	-0.696401	0.397951		6	-4.477441	-3.066177	1.077968
	1	-4.732492	-1.641911	0.061979		1	-5.138866	-3.919292	0.900298
	1	-4.949472	-0.568606	1.465368		1	-3.718419	-3.361887	1.807950
	1	-6.245051	-0.730194	0.268051		1	-5.070351	-2.251944	1.505210
	6	-4.780477	0.307716	-1.909063		6	-4.879861	-2.150983	-1.236021
	1	-4.322219	-0.617114	-2.259270		1	-5.456124	-1.321468	-0.814001
	1	-5.850979	0.284716	-2.138567		1	-4.417005	-1.822188	-2.166670
	1	-4.336637	1.151061	-2.447739		1	-5.572824	-2.969834	-1.455165
	6	-5.185023	1.801417	0.078201		6	-2.941704	-3.744709	-0.802326

Species	Cartesian coordinates				Species	Cartesian coordinates			
	1	-4.730279	2.644620	-0.449403		1	-2.457948	-3.430489	-1.727374
	1	-6.262826	1.812721	-0.110400		1	-2.170878	-4.025787	-0.078223
	1	-5.019771	1.938327	1.151102		1	-3.556568	-4.627450	-1.006312
	44	0.706540	-1.353596	-0.328751		44	1.878596	0.612323	-0.251788
	6	1.201928	-3.549604	-0.789283		6	3.985499	0.809754	-1.344292
	6	-0.122888	-3.251655	-1.266001		6	3.205285	1.751721	-2.028580
	6	-0.005166	-2.312042	-2.336137		6	2.660026	2.651201	-1.059612
	6	1.354735	-1.976919	-2.476660		6	3.183040	2.324268	0.229776
	6	2.112409	-2.735334	-1.499703		6	3.966745	1.135012	0.068300
	1	1.457852	-4.252631	-0.007383		1	4.488992	-0.041903	-1.781511
	1	-1.048272	-3.702415	-0.940769		1	2.987054	1.752111	-3.088440
	1	-0.837995	-1.880005	-2.871133		1	2.007857	3.485032	-1.282845
	1	1.771007	-1.260060	-3.169750		1	3.029730	2.869489	1.150304
	1	3.184440	-2.702959	-1.370921		1	4.548146	0.645004	0.836745
	53	2.824523	0.514633	-0.566110		53	1.839911	-2.045775	-0.820087
	6	0.722322	-0.752877	1.867450		6	0.412504	0.540241	2.390200
	6	-0.107437	-1.743804	1.369829		6	1.501610	0.066200	1.756677
	6	1.834280	-1.172537	2.813875		6	0.026150	0.242356	3.821573
	1	2.714270	-0.547369	2.636671		1	0.679289	-0.498858	4.280986
	1	2.118438	-2.216291	2.664497		1	0.034558	1.158450	4.429096
	6	-1.062756	-2.729657	1.898809		6	2.490069	-0.853459	2.467608
	1	-1.969269	-2.627746	1.276354		1	3.372687	-1.035032	1.842978
	1	-0.670793	-3.743644	1.704912		1	2.842898	-0.370459	3.388958
	8	-1.297544	-2.495179	3.275373		8	1.926138	-2.094637	2.887966
	1	-2.047030	-3.041064	3.556842		1	1.545278	-2.505439	2.095168
	1	1.514315	-1.047205	3.856155		1	-0.996051	-0.155188	3.876064
**TS6d**	6	-1.861737	2.424393	-0.942910	**INT5d**	6	2.647367	-1.831252	-0.576672
	6	-2.222528	2.323473	0.414424		6	2.726099	-1.364918	0.746268
	6	-3.079133	3.238518	1.001643		6	3.885800	-1.523888	1.492237
	6	-3.597383	4.268055	0.194734		6	4.980637	-2.155967	0.883171
	6	-3.239512	4.370570	-1.149533		6	4.900198	-2.625598	-0.430881
	6	-2.352251	3.448855	-1.734939		6	3.723021	-2.470650	-1.177760
	1	-3.360482	3.164796	2.049082		1	3.951547	-1.170279	2.517588
	1	-4.288661	4.988291	0.623311		1	5.904811	-2.280391	1.440607
	1	-3.656385	5.170210	-1.755606		1	5.762975	-3.107077	-0.882348
	1	-2.078215	3.535955	-2.782913		1	3.667494	-2.830150	-2.201921
	6	-0.873920	1.272273	-1.186163		6	1.257440	-1.475290	-1.071004
	6	0.388092	1.705119	-0.400109		6	0.106950	-2.234308	-0.392925
	6	0.025141	1.555658	1.016993		6	0.400209	-2.103911	1.113206
	6	-1.465101	1.096490	0.948117		6	1.346570	-0.837866	1.083365
	1	-0.741003	0.910859	-2.201582		1	1.162087	-1.298791	-2.138818
	1	0.820570	2.649458	-0.721784		1	-0.173273	-3.221856	-0.754195
	1	0.245003	2.378802	1.691448		1	0.981232	-2.953645	1.497790
	1	-1.884349	0.589863	1.810580		1	1.240593	-0.139405	1.908596
	7	-1.405706	0.260261	-0.256763		7	0.928302	-0.235017	-0.253339
	44	1.984894	0.459828	0.266735		6	1.560279	0.979753	-0.703060
	6	0.651070	0.067721	2.181182		8	1.675211	1.229123	-1.885139
	6	1.234524	-0.953996	1.662218		8	1.951361	1.711033	0.329586
	6	-2.356334	-0.666250	-0.622697		6	2.643762	3.017489	0.145374
	8	-2.557834	-1.010932	-1.774905		6	2.845696	3.480469	1.588337
	8	-2.974615	-1.147672	0.480522		1	3.365947	4.442603	1.598374
	6	-3.783142	-2.377812	0.419771		1	1.883087	3.599656	2.092703
	6	-2.910600	-3.534649	-0.077044		1	3.445856	2.756911	2.147564
	1	-2.638429	-3.396889	-1.124455		6	3.983713	2.771132	-0.551884
	1	-1.996307	-3.598164	0.521191		1	4.572662	2.031917	-0.000506
	1	-3.458464	-4.477649	0.021666		1	3.838387	2.418510	-1.573952
	6	-4.178566	-2.586869	1.883945		1	4.551864	3.706067	-0.583807
	1	-4.746811	-1.729222	2.257064		6	1.745698	3.987078	-0.624456
	1	-4.801053	-3.481431	1.980015		1	1.600534	3.661925	-1.654896
	1	-3.289470	-2.714358	2.508153		1	0.769409	4.069664	-0.140044
	6	-5.023706	-2.154150	-0.450904		1	2.215867	4.975844	-0.631616
	1	-5.592177	-1.291162	-0.089208		44	-1.299596	-0.591461	-0.718619
	1	-4.743870	-1.981363	-1.489919		6	-2.475259	0.377659	-2.551961
	1	-5.672227	-3.034978	-0.397908		6	-1.449504	-0.361626	-3.160651
	6	4.155883	0.168583	-0.468243		6	-1.562013	-1.711082	-2.704692
	6	3.664176	1.454237	-0.888277		6	-2.731831	-1.827090	-1.877057
	6	3.408857	2.212684	0.289854		6	-3.277575	-0.521172	-1.746780
	6	3.731210	1.392439	1.424550		1	-2.636577	1.442636	-2.644639
	6	4.227521	0.146456	0.946228		1	-0.662134	0.036552	-3.786601
	1	4.428354	-0.642561	-1.128254		1	-0.923911	-2.531508	-3.005165

Species	Cartesian coordinates				Species	Cartesian coordinates			
	1	3.550746	1.785277	-1.911041		1	-3.127367	-2.738407	-1.450844
	1	3.058513	3.236006	0.329699		1	-4.175157	-0.249162	-1.210216
	1	3.653419	1.689256	2.462666		53	-1.827277	1.883287	0.566688
	1	4.551430	-0.688353	1.553035		6	-0.891772	-1.953801	1.863435
	53	1.364272	-1.460547	-1.709351		6	-1.872485	-1.355186	1.167668
	6	0.244699	0.531662	3.541440		6	-0.964104	-2.477594	3.277588
	1	-0.743248	1.000050	3.570991		1	-1.922874	-2.250968	3.743842
	1	0.965239	1.261349	3.930495		1	-0.791141	-3.562730	3.309666
	6	1.474927	-2.403298	1.956885		6	-3.280680	-1.174259	1.715389
	1	2.518418	-2.660068	1.723631		1	-3.927952	-0.696040	0.971035
	1	1.319389	-2.582123	3.029610		1	-3.715713	-2.159125	1.933641
	8	0.569269	-3.256985	1.279575		8	-3.341467	-0.455754	2.947934
	1	0.672985	-3.076286	0.327483		1	-2.916691	0.400959	2.778050
	1	0.237577	-0.327096	4.220630		1	-0.181923	-2.022456	3.901682
**TS7d**	6	1.517642	2.074496	0.674904	**INT6d**	6	-3.224642	-1.876788	0.447830
	6	2.198850	2.170096	-0.554405		6	-2.192596	-1.797279	-0.515950
	6	2.838032	3.343048	-0.926071		6	-2.476689	-2.060533	-1.857800
	6	2.806458	4.425309	-0.031225		6	-3.768463	-2.408568	-2.259066
	6	2.129097	4.329751	1.187037		6	-4.790825	-2.488756	-1.310952
	6	1.458752	3.150687	1.549246		6	-4.519915	-2.221681	0.030593
	1	3.362679	3.426979	-1.874305		1	-1.679465	-1.990353	-2.593242
	1	3.320860	5.347338	-0.287462		1	-3.973802	-2.615353	-3.305242
	1	2.125962	5.177556	1.866401		1	-5.797515	-2.760703	-1.616077
	1	0.927786	3.082231	2.494706		1	-5.313855	-2.290123	0.770604
	6	0.894043	0.671922	0.691048		6	-2.917521	-1.599200	1.853591
	6	-0.242155	0.841892	-0.348419		6	-1.650901	-1.582317	2.292542
	6	0.498098	0.755405	-1.705997		6	-0.507126	-1.919983	1.353752
	6	1.996222	0.825524	-1.244940		6	-0.785049	-1.413524	-0.082652
	1	0.640333	0.231730	1.650100		1	-3.748765	-1.420971	2.532292
	1	-0.826990	1.778667	-0.105806		1	-1.425092	-1.374167	3.334365
	1	0.258258	1.538992	-2.434118		1	-0.537571	-3.018745	1.234696
	1	2.738024	0.522305	-1.979241		1	-0.058679	-1.905223	-0.738959
	7	1.901756	-0.083631	-0.077213		7	-0.483973	0.034061	-0.203815
	44	-2.278848	0.312502	-0.368159		6	-1.550273	0.924737	-0.582460
	6	0.111176	-0.656765	-2.079772		8	-1.739503	1.237519	-1.739396
	6	-0.866621	-1.128183	-1.276649		8	-2.218747	1.360638	0.484682
	6	2.968734	-0.682964	0.553538		6	-3.376949	2.290406	0.353431
	8	2.963386	-1.007407	1.730382		6	-3.832831	2.445573	1.804778
	8	3.966632	-0.905061	-0.334578		1	-3.035455	2.871671	2.420714
	6	5.199476	-1.605073	0.067902		1	-4.699788	3.110837	1.852969
	6	6.009430	-1.623963	-1.231009		1	-4.111791	1.476186	2.225705
	1	6.967954	-2.125337	-1.067902		6	-2.901020	3.626631	-0.222046
	1	5.468304	-2.159512	-2.016705		1	-3.733651	4.337096	-0.218696
	1	6.206644	-0.605558	-1.578697		1	-2.098825	4.051047	0.389909
	6	4.868739	-3.030049	0.522748		1	-2.550917	3.511818	-1.248852
	1	4.273224	-3.020889	1.435834		6	-4.472566	1.652391	-0.503322
	1	4.313452	-3.559298	-0.258203		1	-4.148479	1.529556	-1.536917
	1	5.796821	-3.579494	0.710958		1	-4.755085	0.674769	-0.105412
	6	5.928685	-0.801753	1.150020		1	-5.356149	2.298542	-0.488314
	1	6.105549	0.222850	0.807835		44	1.260643	0.797179	0.026735
	1	5.348673	-0.770972	2.072482		6	2.202357	2.397015	1.401747
	1	6.899420	-1.263551	1.357461		6	3.037153	2.284955	0.264334
	6	-3.650260	-0.582223	-1.895766		6	2.271311	2.687882	-0.879860
	6	-4.487421	-0.203992	-0.795817		6	0.986977	3.110989	-0.432518
	6	-4.373691	1.190341	-0.620625		6	0.926925	2.895972	0.970066
	6	-3.484650	1.693882	-1.649307		1	2.472111	2.167321	2.423064
	6	-3.076859	0.608824	-2.463104		1	4.065066	1.952276	0.246601
	1	-3.507394	-1.595593	-2.238671		1	2.624169	2.699627	-1.902155
	1	-5.049002	-0.881916	-0.167860		1	0.183956	3.469586	-1.061757
	1	-4.848451	1.779034	0.153031		1	0.077093	3.084428	1.612585
	1	-3.205550	2.731556	-1.785068		53	2.659068	-0.867503	-1.653464
	1	-2.432276	0.657617	-3.328502		6	0.870298	-1.614022	1.916248
	53	-2.274862	-0.701433	2.196425		6	1.739854	-0.688860	1.472813
	6	0.940616	-1.441897	-3.064754		6	1.227650	-2.582232	3.039633
	1	0.450816	-2.366359	-3.380162		1	2.039194	-2.224354	3.672748
	1	1.917895	-1.710417	-2.642461		1	0.354886	-2.773048	3.675077
	6	-1.072728	-2.592216	-0.956469		6	3.170727	-0.670384	2.003525
	1	-0.437651	-2.818300	-0.086193		1	3.752179	0.124241	1.528346
	1	-0.686624	-3.189144	-1.792261		1	3.169267	-0.461057	3.082690
	8	-2.396157	-3.048064	-0.730905		8	3.850376	-1.913840	1.855441

Species	Cartesian coordinates				Species	Cartesian coordinates			
	1	-2.664758	-2.703256	0.136753		1	3.799623	-2.142009	0.913293
	1	1.131870	-0.844384	-3.964844		1	1.543928	-3.548632	2.628536
**TS8d**	6	-2.435214	-2.045741	0.498899	**INT8d**	6	-1.539741	-0.821398	1.854182
	6	-2.494862	-1.465670	-0.788060		6	-1.206747	0.598564	1.529906
	6	-3.686987	-1.463270	-1.493734		6	-0.029966	0.958820	2.475077
	6	-4.828420	-2.029048	-0.904562		6	0.849559	-1.160259	1.312062
	6	-4.781146	-2.573255	0.383391		6	-0.484098	-1.747097	1.719066
	6	-3.584489	-2.572656	1.102923		7	0.760795	-0.565246	-0.042414
	1	-3.743795	-1.006833	-2.477580		1	-2.063107	1.265359	1.653897
	1	-5.768969	-2.022939	-1.448377		1	1.623526	-1.926432	1.317947
	1	-5.679673	-2.989557	0.828481		6	-0.679110	-3.094323	2.014697
	1	-3.537495	-3.000535	2.100771		1	0.142908	-3.797617	1.901020
	6	-1.110078	-2.052758	1.095520		6	-1.924836	-3.535495	2.471136
	6	-0.157194	-2.094682	-1.165834		1	-2.076468	-4.584658	2.709069
	6	-1.144995	-0.875468	-1.135383		6	-2.970387	-2.621772	2.625810
	1	-1.056683	-2.112827	2.181536		1	-3.937981	-2.961299	2.985815
	1	-0.679609	-2.943374	-1.635655		6	-2.782206	-1.274105	2.313652
	1	-1.136982	-0.286442	-2.055471		1	-3.603191	-0.570205	2.421802
	7	-0.706732	-0.079875	0.049116		6	1.150549	-0.066352	2.366498
	44	1.287243	-0.496018	0.759936		6	2.047133	1.136243	2.115669
	6	0.076910	-2.325971	0.322385		6	1.040341	2.023162	2.250425
	1	0.679482	-3.180868	0.630327		1	1.368393	-0.575762	3.314805
	6	-1.419242	1.015652	0.485576		1	-0.452936	0.988016	3.492689
	8	-1.226781	1.559512	1.566574		6	3.525141	1.246029	1.945767
	8	-2.360274	1.388430	-0.416905		1	4.047470	0.844385	2.824674
	6	-3.283153	2.504988	-0.147529		1	3.865243	0.659765	1.085073
	6	-4.148041	2.183928	1.076287		6	1.019113	3.514634	2.395631
	1	-3.541175	2.139915	1.980964		1	1.378786	3.784212	3.402867
	1	-4.910827	2.959669	1.200179		1	-0.015050	3.889330	2.331493
	1	-4.656598	1.224007	0.941103		1	0.973124	1.712774	-3.135358
	6	-2.498766	3.810139	0.018220		6	0.265024	2.095258	-2.416276
	1	-1.872854	3.779587	0.909745		6	0.575983	2.619410	-1.139922
	1	-1.859527	3.981767	-0.852842		6	-1.152380	2.036724	-2.525458
	1	-3.198807	4.648284	0.101350		6	-0.657798	2.947023	-0.475970
	6	-4.136504	2.544800	-1.418294		1	1.555716	2.780028	-0.713742
	1	-4.669116	1.599385	-1.556253		44	-0.659226	0.709045	-0.616819
	1	-4.872851	3.351254	-1.350345		6	-1.725581	2.609755	-1.346526
	1	-3.509504	2.721629	-2.297001		1	-1.708864	1.634082	-3.360682
	6	3.138197	-0.075806	1.955028		1	-0.754082	3.433882	0.480266
	6	2.105865	0.718204	2.598414		53	-2.967146	-0.637702	-1.253820
	6	1.166384	-0.154069	3.174164		1	-2.779907	2.742564	-1.150658
	6	1.559297	-1.483124	2.841501		1	4.312753	-3.376850	-2.747472
	6	2.809025	-1.441246	2.130114		6	3.468158	-2.909174	-2.231549
	1	4.025506	0.318611	1.481079		6	3.996462	-1.913411	-1.194571
	1	2.069587	1.797859	2.621274		1	2.890322	-3.699140	-1.741796
	1	0.256222	0.140654	3.677053		1	2.836883	-2.415799	-2.970771
	1	1.047199	-2.388957	3.141445		8	2.877688	-1.399323	-0.374041
	1	3.391615	-2.294342	1.811292		6	4.867147	-2.623855	-0.155263
	53	1.862358	1.958530	-0.645585		6	4.756881	-0.749777	-1.837773
	6	1.185781	-1.875991	-1.819929		6	1.822437	-0.768609	-0.935195
	6	2.093664	-1.258671	-1.047550		1	4.312333	-3.433578	0.327501
	6	1.380949	-2.387591	-3.227252		1	5.750928	-3.050969	-0.638175
	1	0.627741	-1.957732	-3.902608		1	5.199948	-1.923834	0.616564
	1	1.255095	-3.478647	-3.276786		1	4.130085	-0.223138	-2.556939
	6	3.545851	-1.049867	-1.446379		1	5.094828	-0.042259	-1.073923
	1	4.032562	-2.027980	-1.562170		1	5.641775	-1.135608	-2.354280
	1	4.087587	-0.513868	-0.658380		8	1.777393	-0.429782	-2.110785
	8	3.722443	-0.390051	-2.698825		8	1.852314	4.104347	1.395933
	1	3.262922	0.462309	-2.615093		1	1.934731	5.050600	1.586777
	1	2.362303	-2.118284	-3.617304		1	3.825308	2.287173	1.805589
**TS9d**	6	3.529712	-1.701738	0.274174	**INT9d**	6	0.585943	2.284777	1.557885
	6	2.746669	-0.626586	-0.195569		6	1.596411	1.231754	1.657575
	6	2.956847	-0.152880	-1.492595		6	-0.770716	1.908491	1.605388
	6	3.910212	-0.741850	-2.328538		6	-1.057176	0.424222	1.583692
	6	4.675047	-1.816468	-1.868706		7	-0.719077	-0.145149	0.267105
	6	4.485040	-2.288386	-0.571629		1	2.635598	1.525394	1.535382
	1	2.369307	0.689083	-1.843541		1	-2.107506	0.234962	1.813795
	1	4.059644	-0.355761	-3.333206		6	-1.759351	2.888220	1.601154
	1	5.418724	-2.276987	-2.512838		1	-2.805322	2.594433	1.618600
	1	5.079287	-3.120486	-0.200933		6	-1.406206	4.241000	1.561913

Species	Cartesian coordinates				Species	Cartesian coordinates			
	6	3.334192	-2.188172	1.639780		1	-2.181609	5.001963	1.564097
	6	2.350352	-1.741171	2.434536		6	-0.061053	4.616015	1.509105
	6	1.355998	-0.693318	2.008061		1	0.210322	5.666897	1.469704
	6	1.777645	0.101588	0.737197		6	0.934625	3.640324	1.495729
	1	4.027930	-2.942144	2.006649		1	1.982154	3.925319	1.445861
	1	2.254871	-2.136409	3.442510		44	1.308759	-0.664781	0.118845
	1	1.274763	0.035810	2.827808		6	-0.139654	-0.313708	2.617126
	1	2.335054	0.973610	1.094133		1	-0.376110	0.026202	3.635668
	7	0.573340	0.590193	0.064899		6	-0.322895	-1.793791	2.420772
	6	-0.098071	-1.250516	1.874064		6	0.237324	-2.260760	1.292290
	6	-1.141028	-0.345561	1.656678		6	1.291427	0.007118	2.242553
	44	-0.936957	-0.994245	-0.141613		1	2.072448	-0.570937	2.728491
	6	0.400229	1.924912	-0.140847		6	3.334964	-0.578172	-1.046318
	8	-0.661741	2.475383	-0.414196		6	2.486892	-1.458980	-1.837045
	8	1.588922	2.618536	-0.033124		6	3.565422	-1.185397	0.204317
	6	1.630155	4.077934	-0.158583		6	2.171747	-2.567321	-1.055944
	6	0.763119	4.737923	0.920487		6	2.794310	-2.375990	0.246120
	1	-0.292118	4.519178	0.758184		1	3.768896	0.347438	-1.391586
	1	1.054814	4.377034	1.912655		1	2.146393	-1.260169	-2.842761
	1	0.909177	5.823065	0.899313		1	4.174476	-0.788455	1.005430
	6	1.213275	4.502164	-1.571802		1	1.509127	-3.375492	-1.330604
	1	0.170288	4.247439	-1.758698		1	2.789800	-3.083159	1.062006
	1	1.345465	5.583112	-1.688667		53	1.127940	1.626975	-1.835893
	1	1.840477	4.001727	-2.316926		6	-1.219523	-2.518772	3.396096
	6	3.111046	4.391471	0.080737		1	-0.774916	-2.516626	4.399663
	1	3.736998	3.876596	-0.653984		1	-2.186632	-2.006520	3.482253
	1	3.288285	5.467917	-0.005527		6	0.034933	-3.659459	0.753629
	1	3.418911	4.069490	1.080335		1	-0.666652	-4.168075	1.431445
	6	-2.028543	-2.611047	-1.335791		1	0.965881	-4.243190	0.804774
	6	-1.243290	-1.864035	-2.300755		6	-1.672746	-0.593362	-0.570088
	6	0.115376	-2.057348	-1.999911		8	-1.461403	-1.397894	-1.494375
	6	0.205415	-2.861857	-0.822393		8	-2.898284	-0.062058	-0.313554
	6	-1.133490	-3.245827	-0.449802		6	-4.082400	-0.431474	-1.103642
	1	-3.106271	-2.677880	-1.320296		8	-0.442356	-3.763453	-0.576727
	1	-1.643705	-1.261401	-3.103188		1	-0.823451	-2.904533	-0.872140
	1	0.951986	-1.598699	-2.506107		6	-5.188375	0.402143	-0.449136
	1	1.120161	-3.196336	-0.354949		1	-4.955761	1.468979	-0.515439
	1	-1.403901	-3.896133	0.369817		1	-6.142721	0.223625	-0.953585
	53	-3.312260	0.483336	-0.734702		1	-5.301260	0.136636	0.606528
	6	-0.422953	-2.495526	2.686846		6	-4.376919	-1.928194	-0.952892
	1	0.215174	-3.335488	2.397570		1	-3.587540	-2.531623	-1.400751
	1	-1.469499	-2.786066	2.578749		1	-4.468537	-2.192732	0.105744
	6	-1.788103	0.733364	2.409804		1	-5.325718	-2.167466	-1.444405
	1	-1.710416	1.639561	1.784885		6	-3.892561	-0.017379	-2.566649
	1	-2.866694	0.505293	2.463723		1	-3.641551	1.045946	-2.628643
	8	-1.185796	0.864951	3.688863		1	-3.094963	-0.593832	-3.034594
	1	-1.535573	1.667090	4.104451		1	-4.824154	-0.182202	-3.118356
	1	-0.240772	-2.286976	3.748947		1	-1.413558	-3.556669	3.122301
**TS11d**	6	1.451381	1.128221	1.908313	**INT10d**	6	0.475361	2.260311	1.444111
	6	0.025470	1.208360	1.701767		6	1.231325	0.983796	1.260557
	6	-0.464645	2.585143	1.408683		6	0.782445	0.055388	2.416914
	6	2.223667	1.798400	0.921909		6	-1.463156	0.729975	1.362199
	6	1.358985	2.192453	-0.268448		6	-0.932241	2.144577	1.491219
	7	0.583294	1.000517	-0.647367		7	-1.083016	0.161507	0.052182
	1	-0.628026	0.430023	2.087755		1	2.311544	1.137547	1.260609
	1	1.959385	2.560725	-1.106465		1	-2.548613	0.713939	1.459384
	6	3.599865	1.875207	1.058970		6	-1.729923	3.273602	1.648656
	1	4.201624	2.362049	0.296953		1	-2.812409	3.171862	1.681205
	6	4.213868	1.275528	2.170315		6	-1.139219	4.539825	1.758126
	1	5.293824	1.329586	2.277661		1	-1.762599	5.421660	1.875711
	6	3.461583	0.573361	3.116779		6	0.250808	4.662851	1.716766
	1	3.957393	0.092890	3.954627		1	0.711613	5.642741	1.809699
	6	2.075805	0.475437	2.982151		6	1.055886	3.528453	1.563764
	1	1.479800	-0.065519	3.709828		1	2.138895	3.628244	1.544757
	44	-1.321523	-0.859920	-0.539090		6	-0.778708	-0.073672	2.492475
	6	0.370118	3.242313	0.251757		6	-0.562662	-1.574549	2.421526
	6	-0.980711	3.461460	-0.422968		6	0.783089	-1.471066	2.410764
	6	-1.691630	2.921244	0.584957		1	-1.198253	0.264998	3.449519
	1	0.899929	4.158703	0.536043		1	1.213850	0.480439	3.337854
	1	-0.381443	3.130910	2.365880		6	-1.526685	-2.711703	2.488515

Species	Cartesian coordinates				Species	Cartesian coordinates			
	6	-1.292653	4.087775	-1.739557		1	-2.090647	-2.691485	3.431090
	1	-0.837077	5.083188	-1.816579		1	-2.265502	-2.651327	1.680568
	1	-0.879066	3.483758	-2.557032		6	1.881796	-2.477830	2.597579
	6	-3.132393	2.765718	0.930926		1	1.988099	-2.704103	3.667467
	1	-3.361346	3.398246	1.807030		1	2.839084	-2.057254	2.263799
	1	-3.323468	1.725459	1.239728		1	-0.090548	3.155819	-0.991911
	6	-2.479356	-0.559787	-2.399779		6	0.346724	2.405551	-1.632835
	6	-3.103965	0.178671	-1.347272		6	-0.349995	1.611407	-2.606123
	6	-2.485491	-1.944937	-2.017011		6	1.742818	2.102565	-1.742641
	6	-3.531430	-0.738565	-0.329646		6	0.588153	0.840970	-3.314956
	6	-3.141448	-2.051153	-0.747429		1	-1.421127	1.583058	-2.744621
	1	-2.068085	-0.149606	-3.312037		44	0.718367	0.381981	-0.800141
	1	-3.246419	1.251117	-1.319753		6	1.871658	1.108978	-2.765317
	1	-2.069554	-2.768209	-2.582606		1	2.549059	2.582361	-1.205519
	1	-4.068436	-0.495325	0.576349		1	0.363500	0.098647	-4.068368
	1	-3.289616	-2.965277	-0.189472		53	2.858765	-1.307125	-0.672842
	53	-0.589139	-2.414452	1.722856		1	2.798632	0.653312	-3.081699
	6	1.242450	-0.023602	-1.193497		1	-5.609532	-0.898616	-2.590472
	8	0.675294	-1.138081	-1.369711		6	-4.693865	-0.486788	-2.154196
	8	2.516863	0.197476	-1.577554		6	-4.360133	-1.237633	-0.860935
	6	3.328873	-0.818194	-2.275533		1	-4.866584	0.573803	-1.944501
	6	3.555155	-2.024556	-1.360053		1	-3.886071	-0.580466	-2.880277
	6	2.667754	-1.202956	-3.602828		8	-3.233137	-0.573294	-0.174857
	6	4.642784	-0.071976	-2.524012		6	-5.494647	-1.093812	0.156718
	1	4.259068	-2.716449	-1.834356		6	-4.040401	-2.714236	-1.113254
	1	3.983650	-1.700845	-0.406692		6	-2.007121	-0.488883	-0.741024
	1	2.621248	-2.550894	-1.162310		1	-5.697795	-0.039100	0.364404
	1	3.337651	-1.859542	-4.167454		1	-6.408116	-1.551284	-0.234442
	1	1.724783	-1.723621	-3.435458		1	-5.235750	-1.588275	1.097359
	1	2.479261	-0.309843	-4.207364		1	-3.210609	-2.824334	-1.811094
	1	5.090846	0.243454	-1.577398		1	-3.786113	-3.218269	-0.175711
	1	5.351847	-0.724108	-3.042375		1	-4.922858	-3.208718	-1.532097
	1	4.473876	0.815561	-3.141219		8	-1.725393	-0.952361	-1.846010
	8	-3.932557	3.136261	-0.186615		8	1.613433	-3.730218	1.971872
	1	-4.863973	3.015690	0.048997		1	1.530051	-3.551045	1.022018
	1	-2.370375	4.180069	-1.882095		1	-0.999858	-3.665742	2.417124
**TS12d**	6	-1.482809	-1.247803	1.856633	**INT11d**	6	2.256133	-2.417098	-0.115792
	6	-1.797997	0.155736	1.489037		6	0.928242	-2.913728	-0.337852
	6	-0.130259	-1.642402	1.888885		6	2.440709	-1.387676	0.848808
	6	0.888494	-0.574714	1.541884		6	1.189841	-0.695943	1.369973
	7	0.819316	-0.138099	0.137766		7	0.644003	0.048082	0.215444
	1	-2.854276	0.415610	1.516609		1	0.775415	-3.639753	-1.133865
	1	1.896929	-0.923864	1.763728		1	1.427689	-0.035399	2.210168
	6	0.208231	-2.936543	2.270705		6	3.739881	-0.997788	1.175434
	1	1.254169	-3.233821	2.288779		1	3.889756	-0.216933	1.912854
	6	-0.789002	-3.850585	2.628192		6	4.836139	-1.606419	0.562234
	1	-0.519172	-4.859775	2.926457		1	5.842360	-1.310462	0.846366
	6	-2.129063	-3.461082	2.601549		6	4.655025	-2.591127	-0.420965
	1	-2.907012	-4.167585	2.877763		1	5.515895	-3.043560	-0.903592
	6	-2.476163	-2.164818	2.216109		6	3.371197	-2.988053	-0.765112
	1	-3.520692	-1.865150	2.186131		1	3.214202	-3.762223	-1.512194
	44	-0.921103	0.854747	-0.395893		44	-1.072034	-0.726870	-0.615453
	6	0.533366	0.649824	2.424058		6	0.167245	-1.780873	1.772101
	1	0.612436	0.390502	3.487977		1	0.648307	-2.420846	2.531110
	6	1.225393	1.914469	2.035741		6	-1.187436	-1.307904	2.261930
	6	0.331908	2.503335	1.228515		6	-2.042124	-0.975544	1.281597
	6	-0.872492	1.128155	2.034450		6	-0.111337	-2.598777	0.520720
	1	-1.308635	1.923634	2.633016		1	-0.975970	-3.254139	0.543132
	6	-2.210761	1.102744	-2.383287		6	-0.677250	-1.605275	-2.726839
	6	-0.854418	1.516590	-2.644091		6	-1.009612	-0.267181	-2.996089
	6	-2.765596	1.932748	-1.379541		6	-1.826548	-2.238372	-2.095707
	6	-0.584041	2.618236	-1.819787		6	-2.349539	-0.060331	-2.542244
	6	-1.761681	2.856076	-0.999627		6	-2.863926	-1.281444	-2.011859
	1	-2.728026	0.308619	-2.896952		1	0.259507	-2.085688	-2.971131
	1	-0.162953	1.040017	-3.322114		1	-0.351606	0.492033	-3.391128
	1	-3.764737	1.864168	-0.972595		1	-1.905846	-3.281780	-1.820171
	1	0.348589	3.161441	-1.798457		1	-2.894793	0.868784	-2.613807
	1	-1.889853	3.625136	-0.250682		1	-3.862777	-1.443973	-1.633615
	53	-1.561755	-1.808485	-1.442786		53	-2.036747	1.992351	0.003192
	6	2.552320	2.380199	2.545928		6	-1.471295	-1.371100	3.743174

Species	Cartesian coordinates				Species	Cartesian coordinates			
	1	2.578756	2.365986	3.642181		1	-0.739971	-0.769005	4.300000
	1	3.339843	1.700640	2.196309		1	-2.465186	-0.988779	3.973380
	6	0.203615	3.975512	0.971312		6	-3.532559	-0.734278	1.469534
	1	0.193845	4.254863	-0.086157		1	-4.011718	-1.681403	1.753299
	1	1.094563	4.445225	1.415714		1	-3.987391	-0.409694	0.527327
	6	1.906112	-0.137508	-0.671552		6	1.338323	1.075644	-0.356601
	8	1.926826	0.330862	-1.811475		8	1.114917	1.523799	-1.478821
	8	3.006352	-0.719693	-0.086530		8	2.294169	1.578759	0.482328
	6	4.249982	-0.914245	-0.833102		6	3.123351	2.723926	0.083398
	6	4.006469	-1.808160	-2.054914		1	-1.379193	-2.399295	4.121572
	6	4.860471	0.438651	-1.219450		6	2.244830	3.958055	-0.151523
	6	5.143683	-1.631887	0.184298		1	1.583263	3.807215	-1.003934
	1	4.961075	-2.043086	-2.537852		1	1.634572	4.162181	0.733573
	1	3.538744	-2.748611	-1.747050		1	2.879653	4.830724	-0.339307
	1	3.355111	-1.311858	-2.773850		6	4.019972	2.938931	1.307248
	1	4.215031	0.964710	-1.922405		1	4.657308	2.067533	1.484775
	1	4.997497	1.061155	-0.328583		1	4.666098	3.808064	1.150754
	1	5.842761	0.285572	-1.679132		1	3.414626	3.114780	2.201307
	1	4.693442	-2.580716	0.490750		6	3.969063	2.371646	-1.146235
	1	6.125021	-1.839337	-0.253269		1	3.336633	2.217189	-2.020231
	1	5.287302	-1.014330	1.076515		1	4.672311	3.185003	-1.354791
	8	-0.986167	4.437118	1.622401		1	4.546310	1.460109	-0.961274
	1	-1.031340	5.400043	1.517448		8	-3.857039	0.179996	2.513432
	1	2.807258	3.386886	2.205459		1	-3.446711	1.024211	2.260579
**TS13d**	6	-2.225226	-2.062482	0.622373					
	6	-0.786099	-2.218597	0.610184					
	6	-0.234189	-2.847319	-0.629333					
	6	-2.769919	-1.512243	-0.567159					
	6	-1.667653	-0.990034	-1.476523					
	7	-0.799583	-0.167454	-0.625364					
	1	-0.263957	-2.332936	1.550790					
	1	-2.063094	-0.416471	-2.320130					
	6	-4.143436	-1.396318	-0.708025					
	1	-4.568155	-0.957147	-1.606084					
	6	-4.979271	-1.816304	0.338936					
	1	-6.056293	-1.717626	0.235236					
	6	-4.445697	-2.328218	1.525895					
	1	-5.107881	-2.634445	2.329925					
	6	-3.063317	-2.439101	1.682208					
	1	-2.641106	-2.848558	2.596026					
	44	0.980956	0.245308	1.183294					
	6	-0.853283	-2.214745	-1.919846					
	6	0.586448	-2.032548	-2.376999					
	6	1.123250	-2.561605	-1.260749					
	1	-1.471206	-2.887154	-2.526346					
	1	-0.468327	-3.922065	-0.513810					
	6	0.731035	-0.591817	3.246819					
	6	1.116388	0.780210	3.298573					
	6	1.793748	-1.306916	2.579072					
	6	2.398378	0.917369	2.672855					
	6	2.823681	-0.392544	2.243096					
	1	-0.170011	-1.017553	3.667995					
	1	0.525625	1.590223	3.706149					
	1	1.821984	-2.373370	2.395254					
	1	2.955354	1.836212	2.556356					
	1	3.748696	-0.626823	1.735594					
	53	2.487287	1.396693	-0.918151					
	6	-1.234519	1.022907	-0.225235					
	8	-0.616155	1.641987	0.689296					
	8	-2.324003	1.526213	-0.834748					
	6	-2.835811	2.885283	-0.565265					
	6	-3.303217	2.984237	0.890171					
	6	-1.771833	3.929502	-0.913307					
	6	-4.025797	2.980387	-1.523854					
	1	-3.802290	3.945608	1.049678					
	1	-4.019208	2.187035	1.113773					
	1	-2.461222	2.906962	1.578350					
	1	-2.200605	4.932356	-0.816731					
	1	-0.908408	3.849530	-0.252552					
	1	-1.434912	3.800353	-1.946230					

Species	Cartesian coordinates	Species	Cartesian coordinates
	1 -4.763143 2.202951 -1.302643		
	1 -4.510482 3.955953 -1.423873		
	1 -3.696616 2.862160 -2.560237		
	6 1.121821 -1.439897 -3.634507		
	1 2.203996 -1.568811 -3.688054		
	1 0.658487 -1.903720 -4.514592		
	6 2.520566 -2.847658 -0.800365		
	1 2.762950 -3.904612 -0.983755		
	1 2.579470 -2.706300 0.290133		
	8 3.503536 -2.092313 -1.480846		
	1 3.292494 -1.148338 -1.347354		
	1 0.903332 -0.365900 -3.671983		

Table S8.The optimized cartesian coordinates (Å) for reaction **e**, located at IDSCRF-B3LYP/BS1 level in dioxane solvent.

Species	Cartesian coordinates	Species	Cartesian coordinates
**R2e**	6 -0.608412 -0.007848 0.003301	**P1e**	6 -4.460585 -2.897049 0.072469
	6 0.608256 0.011497 0.001152		6 -3.421334 -2.979427 0.998004
	6 -2.033752 -0.030509 0.005819		6 -2.159964 -2.438582 0.708431
	6 -2.758430 0.320737 -1.151304		6 -3.004093 -1.702922 -1.439967
	6 -2.742806 -0.404548 1.165474		6 -4.247380 -2.274001 -1.158801
	6 -4.150249 0.297058 -1.143884		1 -5.428388 -3.332803 0.303996
	1 -2.217548 0.609502 -2.046892		1 -3.572863 -3.493264 1.943943
	6 -4.134693 -0.425124 1.162972		1 -2.843109 -1.194310 -2.386377
	1 -2.189858 -0.675923 2.059130		1 -5.045288 -2.226301 -1.894100
	6 -4.843326 -0.075176 0.010782		6 -1.964871 -1.758567 -0.512335
	1 -4.696537 0.569995 -2.042202		6 -1.019716 -2.642220 1.604742
	1 -4.668852 -0.715239 2.063198		6 0.566489 -1.962820 -0.209729
	1 -5.929261 -0.092440 0.012700		6 0.240729 -2.470569 1.182664
	6 2.033609 0.034158 -0.001367		1 -1.221257 -3.009647 2.608347
	6 2.742676 0.407531 -1.161235		1 1.073953 -2.678879 1.847802
	6 2.758282 -0.316421 1.155967		6 -0.608920 -1.160992 -0.817172
	6 4.134563 0.428107 -1.158731		6 1.676814 -0.911945 -0.158332
	1 2.189731 0.678387 -2.055053		6 1.086823 0.309706 -0.074568
	6 4.150102 -0.292740 1.148542		1 0.832400 -2.800987 -0.867060
	1 2.217390 -0.604668 2.051718		1 -0.478602 -1.106075 -1.905258
	6 4.843186 0.078826 -0.006334		7 -0.315242 0.211809 -0.272246
	1 4.668728 0.717701 -2.059122		6 -1.202082 1.165893 0.207313
	1 4.696383 -0.565156 2.047023		8 -2.372611 1.115075 -0.460493
	1 5.929120 0.096091 -0.008253		8 -0.915628 1.966477 1.081666
			6 -3.499480 1.984275 -0.057546
			6 -3.119791 3.456274 -0.251303
			1 -3.997587 4.084210 -0.067085
			1 -2.322344 3.751573 0.429423
			1 -2.790434 3.629699 -1.280774
			6 -4.605019 1.587324 -1.038131
			1 -4.282057 1.752666 -2.070310
			1 -4.869745 0.534145 -0.921544
			1 -5.496111 2.195191 -0.855136
			6 -3.913677 1.668802 1.382879
			1 -3.130873 1.948386 2.088359
			1 -4.823726 2.224669 1.630580
			1 -4.126082 0.600751 1.489690
			6 3.097850 -1.277944 -0.240260
			6 3.510496 -2.583639 0.097898
			6 4.087338 -0.389499 -0.712871
			6 4.846354 -2.971161 0.004477
			1 2.781245 -3.309144 0.442788
			6 5.419734 -0.780173 -0.807826
			1 3.805699 0.608639 -1.024147
			6 5.812320 -2.070874 -0.444166
			1 5.129298 -3.983198 0.280913
			1 6.155725 -0.072672 -1.180142
			1 6.852975 -2.372102 -0.520813
			6 1.723800 1.638868 0.078728
			6 1.521974 2.642291 -0.881261
			6 2.558132 1.904301 1.173800
			6 2.151132 3.879489 -0.753609

Species	Cartesian coordinates				Species	Cartesian coordinates			
						1	0.874947	2.446360	-1.731636
						6	3.183421	3.143062	1.301737
						1	2.707753	1.134964	1.924517
						6	2.983755	4.133996	0.338194
						1	1.990767	4.645572	-1.507031
						1	3.823533	3.336632	2.157796
						1	3.471298	5.099481	0.438861
**P2e**	6	-1.899933	-2.216199	-0.599595	**TS14e**	6	2.718341	-2.455993	0.362762
	6	-0.719666	-1.747359	0.252322		6	1.305346	-2.748375	0.053619
	6	0.511099	-1.774868	-0.711075		6	3.008113	-1.305033	1.129746
	6	-1.182257	-0.052875	-1.136682		6	1.839384	-0.361574	1.348986
	6	-2.191326	-1.149671	-1.471960		7	1.388240	0.103061	0.030144
	7	-0.981323	-0.293953	0.312441		1	1.127409	-3.705730	-0.428636
	6	-1.714932	0.256762	1.333846		1	2.120165	0.479393	1.985054
	8	-1.829743	-0.260074	2.436368		6	4.311482	-1.055242	1.542670
	8	-2.224880	1.451590	0.955515		1	4.530420	-0.163038	2.122182
	6	-2.957578	2.303400	1.907922		6	5.339746	-1.938234	1.187026
	1	-0.600824	-2.195819	1.236220		1	6.358672	-1.734249	1.503305
	1	-1.445810	0.974561	-1.375740		6	5.061697	-3.067513	0.415341
	6	-3.191139	-1.260581	-2.425607		1	5.861401	-3.747573	0.137157
	1	-3.417558	-0.443655	-3.105753		6	3.752613	-3.328733	0.003955
	6	-3.923621	-2.457656	-2.482246		1	3.529960	-4.220773	-0.576663
	1	-4.728257	-2.559913	-3.204964		44	0.133717	-1.169553	-0.943278
	6	-3.635489	-3.514030	-1.617119		6	0.677901	-1.180767	1.980196
	1	-4.218772	-4.428740	-1.673956		1	0.959702	-1.505273	2.988414
	6	-2.605220	-3.407245	-0.668480		6	-0.695533	-0.570594	1.887903
	1	-2.383332	-4.235319	-0.000625		6	-1.231047	-1.246966	0.810526
	6	0.184872	-0.571307	-1.676166		6	0.326313	-2.347710	1.051850
	6	1.449537	0.073428	-1.125293		1	-0.255018	-3.164781	1.475466
	6	1.761877	-0.992575	-0.331864		6	0.944167	-1.151138	-3.070994
	1	0.158726	-0.794265	-2.746520		6	-0.175052	-0.285382	-2.981759
	1	0.658490	-2.762530	-1.157691		6	0.485972	-2.475312	-2.785505
	6	-2.041328	2.698430	3.070177		6	-1.337558	-1.092898	-2.683216
	1	-2.554167	3.422333	3.711665		6	-0.928604	-2.442792	-2.572128
	1	-1.770664	1.827749	3.667689		1	1.964199	-0.847840	-3.256324
	1	-1.127930	3.166979	2.690775		1	-0.169478	0.790127	-3.085385
	6	-3.309649	3.525322	1.055960		1	1.107673	-3.360614	-2.766665
	1	-3.926808	3.234786	0.200783		1	-2.338346	-0.704051	-2.566154
	1	-3.867389	4.251841	1.654165		1	-1.561746	-3.290775	-2.348247
	1	-2.402969	4.008086	0.680224		53	-2.395367	2.572464	-1.648643
	6	-4.226782	1.588338	2.382137		6	2.058903	1.140675	-0.583860
	1	-4.825244	2.270572	2.994510		8	1.942883	1.428266	-1.768784
	1	-4.832412	1.281874	1.523523		8	2.852812	1.812104	0.290830
	1	-3.980554	0.706333	2.973257		6	3.570040	3.034912	-0.113740
	6	1.965491	1.416289	-1.390702		6	4.603970	2.701238	-1.194450
	6	1.667076	2.042929	-2.615580		1	4.115812	2.394356	-2.119238
	6	2.708909	2.140117	-0.438109		1	5.221375	3.582887	-1.395435
	6	2.119769	3.331840	-2.891126		1	5.261924	1.894758	-0.854605
	1	1.084712	1.506938	-3.359684		6	2.573386	4.106029	-0.565979
	6	3.155791	3.429890	-0.713274		1	2.058016	3.807353	-1.478462
	1	2.914964	1.692828	0.528550		1	1.826629	4.285055	0.213426
	6	2.868810	4.030258	-1.942356		1	3.106326	5.044685	-0.750430
	1	1.885604	3.792277	-3.846924		6	4.263860	3.456094	1.184818
	1	3.722616	3.973027	0.037834		1	4.949460	2.675912	1.530113
	1	3.219658	5.036032	-2.154694		1	4.839326	4.372145	1.021882
	6	2.866698	-1.407369	0.531203		1	3.527955	3.646117	1.971574
	6	2.676711	-2.464685	1.441203		6	-1.274743	0.418767	2.779735
	6	4.149279	-0.829584	0.446562		6	-0.717710	0.624285	4.061395
	6	3.716275	-2.906475	2.257276		6	-2.372954	1.211877	2.384084
	1	1.702944	-2.939268	1.511841		6	-1.264994	1.562069	4.929911
	6	5.188196	-1.276539	1.258046		1	0.134166	0.033499	4.384106
	1	4.332926	-0.038948	-0.272784		6	-2.907807	2.156056	3.255645
	6	4.976504	-2.312625	2.172088		1	-2.766767	1.129393	1.378720
	1	3.542006	-3.717575	2.958681		6	-2.364257	2.329100	4.530758
	1	6.170107	-0.819331	1.172521		1	-0.833487	1.699131	5.917214
	1	5.788350	-2.657465	2.805896		1	-3.740387	2.770868	2.927531
						1	-2.785195	3.068098	5.206578
						6	-2.625483	-1.702816	0.585284
						6	-3.549322	-0.945345	-0.154220
						6	-3.055759	-2.909981	1.168142

Species	Cartesian coordinates				Species	Cartesian coordinates			
						6	-4.865467	-1.385831	-0.300261
						1	-3.247898	0.002555	-0.594933
						6	-4.365524	-3.356179	0.998307
						1	-2.369298	-3.501491	1.767536
						6	-5.276596	-2.594800	0.262974
						1	-5.566685	-0.775189	-0.861990
						1	-4.676239	-4.292408	1.453602
						1	-6.299304	-2.938564	0.137133
**TS2e**	6	2.779093	-2.347405	0.470605	**INT1e**	6	2.226574	-2.624050	0.691172
	6	2.797260	-1.284311	1.392057		6	2.648744	-1.651400	1.613790
	6	3.513795	-1.369900	2.573102		6	3.327297	-2.010653	2.768413
	6	4.241852	-2.548528	2.816965		6	3.600249	-3.372354	2.977376
	6	4.224408	-3.602861	1.903715		6	3.177597	-4.338527	2.061104
	6	3.477918	-3.517193	0.714567		6	2.468580	-3.973263	0.905969
	1	3.525036	-0.554027	3.290854		1	3.649412	-1.265756	3.491193
	1	4.827118	-2.637239	3.727897		1	4.150111	-3.678472	3.862987
	1	4.796820	-4.502426	2.111936		1	3.404558	-5.385367	2.242248
	1	3.464639	-4.344840	0.010493		1	2.137599	-4.729544	0.199035
	6	1.843158	-1.889166	-0.657557		6	1.485269	-1.857776	-0.408217
	6	0.435633	-1.899745	-0.010776		6	0.147848	-1.413302	0.220417
	6	0.453682	-0.766009	0.916057		6	0.576061	-0.347078	1.263449
	6	1.893014	-0.199974	0.789786		6	2.133579	-0.322259	1.074929
	1	1.973685	-2.352742	-1.631752		1	1.462376	-2.324165	-1.387184
	1	0.069446	-2.856717	0.345927		1	-0.442601	-2.272150	0.634028
	1	0.056064	-0.948058	1.908756		1	0.341239	-0.660819	2.289580
	1	2.066799	0.821628	1.106526		1	2.639472	0.581797	1.399654
	7	2.109219	-0.435124	-0.649872		7	2.198078	-0.562868	-0.388405
	6	3.046836	0.156621	-1.454200		6	3.272594	-0.330745	-1.213452
	8	3.373051	-0.300631	-2.543669		8	3.451567	-0.927817	-2.266400
	8	3.477988	1.315381	-0.911587		8	4.030615	0.676487	-0.726010
	6	4.422498	2.192656	-1.628925		6	5.206907	1.173007	-1.464280
	6	3.795828	2.679272	-2.939177		6	5.731865	2.280155	-0.547025
	1	3.653135	1.853021	-3.636038		1	6.625936	2.735104	-0.983397
	1	2.828571	3.154267	-2.745531		1	4.978036	3.061208	-0.410994
	1	4.450717	3.423085	-3.404198		1	5.992863	1.876670	0.435569
	6	4.604339	3.353615	-0.648131		6	6.242571	0.053327	-1.609899
	1	5.008793	2.995711	0.302983		1	6.492059	-0.362628	-0.628747
	1	5.297160	4.090762	-1.064364		1	5.867858	-0.746952	-2.248285
	1	3.648443	3.848432	-0.452355		1	7.159299	0.457885	-2.050855
	6	5.748062	1.458561	-1.853536		6	4.772916	1.746746	-2.816754
	1	6.482431	2.150683	-2.277995		1	4.379949	0.963936	-3.465884
	1	6.141513	1.086211	-0.902565		1	4.003402	2.512488	-2.675781
	1	5.619814	0.618486	-2.536401		1	5.631413	2.215709	-3.308245
	44	-1.143304	-0.754613	-0.877929		44	-1.668036	-1.116924	-0.742102
	6	-1.357370	0.422477	-2.822790		6	-2.557566	-0.246225	-2.551800
	6	-0.184392	-0.373020	-2.960413		6	-1.142488	-0.384872	-2.768713
	6	-0.582305	-1.745781	-2.887034		6	-0.861216	-1.777216	-2.810192
	6	-1.987973	-1.798873	-2.690756		6	-2.101745	-2.498717	-2.728007
	6	-2.477599	-0.452296	-2.646867		6	-3.137924	-1.568290	-2.567316
	1	-1.397379	1.503369	-2.836165		1	-3.103296	0.684018	-2.496572
	1	0.825709	-0.021258	-3.115085		1	-0.430765	0.420139	-2.879107
	1	0.071646	-2.602952	-2.963912		1	0.111470	-2.218119	-2.975839
	1	-2.584163	-2.694035	-2.582260		1	-2.206586	-3.576398	-2.730020
	1	-3.509301	-0.156567	-2.524693		1	-4.186106	-1.795809	-2.427069
	53	-2.844062	-2.283801	0.810133		53	-3.619041	-1.356829	1.148668
	6	-0.777464	0.928390	0.852632		6	-0.156760	0.925140	0.922197
	6	-1.853440	0.934575	0.144498		6	-1.183912	0.811720	0.056790
	6	-0.163532	1.742037	1.906114		6	0.217118	2.191468	1.629255
	6	0.560349	2.903081	1.578637		6	-0.555896	2.647994	2.709136
	6	-0.333745	1.407309	3.261106		6	1.348789	2.937496	1.257536
	6	1.091784	3.712903	2.582900		6	-0.210464	3.812810	3.396107
	1	0.707905	3.158643	0.533429		1	-1.435188	2.082299	3.004109
	6	0.199606	2.220201	4.260902		6	1.695075	4.102585	1.944607
	1	-0.906655	0.522881	3.524320		1	1.954332	2.607851	0.417574
	6	0.914357	3.372972	3.925748		6	0.917401	4.543638	3.017081
	1	1.645405	4.608568	2.315384		1	-0.824266	4.150121	4.227163
	1	0.049259	1.956299	5.303911		1	2.570531	4.669069	1.637717
	1	1.328078	4.004123	4.707040		1	1.186342	5.451018	3.550945
	6	-3.009182	1.808136	-0.055785		6	-1.929839	1.993419	-0.448189
	6	-4.311717	1.287490	-0.158882		6	-1.274584	2.903259	-1.301799

Species	Cartesian coordinates				Species	Cartesian coordinates			
	6	-2.828643	3.201989	-0.141493		6	-3.275257	2.248723	-0.125057
	6	-5.401341	2.140250	-0.327187		6	-1.934688	4.022513	-1.809784
	1	-4.455394	0.214755	-0.081727		1	-0.232889	2.728116	-1.554790
	6	-3.922233	4.048603	-0.319656		6	-3.932350	3.372170	-0.626635
	1	-1.828035	3.615271	-0.066195		1	-3.802247	1.558571	0.524452
	6	-5.212344	3.522148	-0.412348		6	-3.268580	4.262691	-1.474838
	1	-6.402340	1.722746	-0.392615		1	-1.404097	4.709174	-2.464292
	1	-3.764896	5.121809	-0.385231		1	-4.968778	3.552168	-0.353118
	1	-6.063371	4.183080	-0.550683		1	-3.785256	5.133349	-1.869102
**TS3e**	6	-0.359460	3.371338	-0.635373	**INT2e**	6	-2.884948	-0.415298	-2.101046
	6	-1.086340	3.361774	0.571049		6	-2.872025	0.897458	-1.609874
	6	-1.170131	4.497377	1.360501		6	-3.759897	1.852878	-2.084448
	6	-0.524736	5.661917	0.913032		6	-4.678603	1.461821	-3.070519
	6	0.197992	5.669267	-0.281919		6	-4.686822	0.155184	-3.567485
	6	0.300275	4.511400	-1.069177		6	-3.779760	-0.802191	-3.089550
	1	-1.724173	4.498319	2.295204		1	-3.749445	2.872887	-1.709901
	1	-0.590652	6.570616	1.504542		1	-5.393736	2.184798	-3.452796
	1	0.685034	6.583539	-0.608680		1	-5.410569	-0.124294	-4.327881
	1	0.867538	4.521744	-1.995926		1	-3.792670	-1.817852	-3.475854
	6	-0.445371	1.942376	-1.177892		6	-1.778606	-1.146867	-1.365723
	6	0.485738	1.164705	-0.210345		6	-0.366665	-0.678532	-1.756594
	6	-0.333622	1.070148	1.102215		6	-0.378836	0.856566	-1.475386
	6	-1.600165	1.934982	0.737067		6	-1.712484	0.968639	-0.637684
	1	-0.311268	1.778061	-2.243486		1	-1.901912	-2.219936	-1.259723
	1	1.483902	1.721730	-0.053390		1	0.000338	-0.989972	-2.729179
	1	0.178705	1.440698	1.994861		1	-0.474923	1.451952	-2.388428
	1	-2.485422	1.771772	1.346027		1	-1.724335	1.759478	0.106632
	7	-1.775003	1.581908	-0.681832		7	-1.722882	-0.411548	-0.038899
	6	-2.697450	0.819019	-1.328429		6	-2.684878	-0.729334	0.953900
	8	-2.567482	0.451365	-2.492909		8	-3.222196	-1.818462	1.023568
	8	-3.753088	0.578807	-0.522828		8	-2.848775	0.305066	1.782065
	6	-4.969153	-0.088288	-1.024173		6	-3.824238	0.259034	2.901970
	6	-5.864051	-0.115283	0.217570		6	-3.680602	1.652188	3.516225
	1	-6.820668	-0.589394	-0.021397		1	-4.352395	1.750599	4.373687
	1	-5.386642	-0.680139	1.023248		1	-2.655589	1.821693	3.857875
	1	-6.060767	0.900101	0.574156		1	-3.935390	2.425861	2.786509
	6	-4.646621	-1.511612	-1.488680		6	-5.233861	0.045288	2.344907
	1	-4.008957	-1.501013	-2.372489		1	-5.462252	0.800300	1.586604
	1	-4.141726	-2.063848	-0.691145		1	-5.341588	-0.945298	1.902027
	1	-5.577938	-2.034460	-1.730295		1	-5.961419	0.146779	3.156107
	6	-5.597947	0.757736	-2.135624		6	-3.415001	-0.827382	3.899095
	1	-5.786288	1.775717	-1.780114		1	-3.486906	-1.820657	3.454531
	1	-4.945819	0.801956	-3.008893		1	-2.390935	-0.660451	4.248557
	1	-6.555619	0.319226	-2.434018		1	-4.076456	-0.787250	4.770131
	44	2.253334	0.088151	-0.655191		44	0.599283	-1.403557	0.023308
	6	3.089477	-1.461900	-1.978277		6	1.405128	-2.519966	1.961882
	6	2.219288	-0.623743	-2.765434		6	-0.009135	-2.483086	1.934489
	6	2.757125	0.690420	-2.752209		6	-0.480968	-3.337345	0.851456
	6	3.990995	0.659784	-2.001877		6	0.639271	-3.798377	0.161920
	6	4.202278	-0.664195	-1.554402		6	1.811475	-3.252141	0.823214
	1	2.959123	-2.518592	-1.795425		1	2.053208	-2.002456	2.653575
	1	1.313010	-0.941488	-3.260494		1	-0.642786	-1.989887	2.659989
	1	2.343646	1.556769	-3.251672		1	-1.520172	-3.538671	0.635513
	1	4.631381	1.507881	-1.797873		1	0.653246	-4.429371	-0.715730
	1	5.019011	-0.998810	-0.929870		1	2.833669	-3.435324	0.526793
	53	3.215628	-0.241491	1.937421		53	2.770105	-1.510028	-1.800939
	6	-0.544323	-0.414428	1.021181		6	0.731513	1.382320	-0.585742
	6	0.252291	-0.849176	0.006658		6	1.300741	0.513353	0.282768
	6	-1.519470	-1.130298	1.864350		6	0.951001	2.858177	-0.612124
	6	-1.875240	-0.554886	3.101420		6	1.165764	3.515429	-1.838578
	6	-2.117209	-2.358344	1.515089		6	0.935945	3.637021	0.558164
	6	-2.780661	-1.179203	3.957088		6	1.372174	4.893290	-1.890491
	1	-1.422326	0.383637	3.405822		1	1.195468	2.937076	-2.758057
	6	-3.028303	-2.976539	2.369135		6	1.135977	5.016878	0.505749
	1	-1.876240	-2.827091	0.570286		1	0.765403	3.154750	1.514864
	6	-3.365132	-2.393542	3.593769		6	1.355916	5.652093	-0.717727
	1	-3.027750	-0.716427	4.908481		1	1.548810	5.374457	-2.848838
	1	-3.477447	-3.921363	2.075384		1	1.116600	5.596722	1.424838
	1	-4.073600	-2.881662	4.257021		1	1.512218	6.726520	-0.757831
	6	0.197407	-2.170898	-0.660338		6	2.343402	0.849544	1.274771

Species	Cartesian coordinates				Species	Cartesian coordinates			
	6	0.881844	-3.268218	-0.103634		6	2.069711	0.789967	2.655240
	6	-0.570832	-2.366516	-1.822662		6	3.633155	1.241446	0.873517
	6	0.784260	-4.530737	-0.687157		6	3.044136	1.115804	3.597803
	1	1.482617	-3.117150	0.788216		1	1.075300	0.496495	2.982319
	6	-0.648835	-3.631329	-2.410726		6	4.610674	1.557584	1.817527
	1	-1.115982	-1.530155	-2.251875		1	3.861571	1.288345	-0.185826
	6	0.023440	-4.717337	-1.845850		6	4.322960	1.497708	3.182524
	1	1.306736	-5.370756	-0.237265		1	2.805196	1.073170	4.657493
	1	-1.246415	-3.767758	-3.308167		1	5.601296	1.854320	1.483021
	1	-0.044520	-5.700643	-2.302632		1	5.085406	1.746279	3.915551
**TS4e**	6	-2.726541	-0.708575	-2.052704	**INT3e**	6	2.306996	2.862502	1.091258
	6	-2.805790	0.604382	-1.561920		6	2.133925	2.521125	-0.270698
	6	-3.878489	1.415564	-1.897065		6	3.055090	2.971015	-1.218463
	6	-4.885221	0.893193	-2.724954		6	4.167023	3.725693	-0.832047
	6	-4.817219	-0.420393	-3.197430		6	4.367638	4.022920	0.517065
	6	-3.734725	-1.240175	-2.858692		6	3.441696	3.595957	1.468947
	1	-3.948889	2.429748	-1.514015		1	2.907833	2.718154	-2.263879
	1	-5.737035	1.513968	-2.988239		1	4.871922	4.073883	-1.581554
	1	-5.612910	-0.810867	-3.824817		1	5.235131	4.599674	0.826279
	1	-3.672575	-2.259382	-3.230535		1	3.579620	3.852589	2.516486
	6	-1.476068	-1.382743	-1.678552		6	1.260430	2.518772	2.055397
	6	-0.323989	0.797844	-1.547695		6	0.035217	2.168588	1.637649
	6	-1.611906	0.860043	-0.671244		6	-0.289916	2.106300	0.155966
	1	-1.472240	-2.471144	-1.690352		6	0.923911	1.669153	-0.665584
	1	-0.467173	1.375830	-2.467754		1	1.484102	2.617905	3.114758
	1	-1.676985	1.782734	-0.095798		1	-0.764926	1.974157	2.343144
	7	-1.473392	-0.353911	0.189022		1	-0.451907	3.151479	-0.151538
	6	-2.471206	-0.564837	1.132241		1	0.712231	1.832383	-1.730636
	8	-2.697419	-1.631638	1.690206		7	1.207502	0.215673	-0.511387
	8	-3.180709	0.567376	1.357611		6	2.434786	-0.139442	-1.168746
	6	-4.341716	0.588093	2.269433		8	2.661925	0.120558	-2.336403
	6	-5.427348	-0.364581	1.758780		8	3.249019	-0.773963	-0.323431
	1	-5.100402	-1.402747	1.818740		6	4.640275	-1.148465	-0.698123
	1	-6.331520	-0.244605	2.364389		6	5.152611	-1.806152	0.583899
	1	-5.678830	-0.130332	0.719836		1	4.552885	-2.684298	0.840268
	6	-3.896375	0.258049	3.697508		1	6.190624	-2.123891	0.449130
	1	-3.556686	-0.774747	3.774715		1	5.108028	-1.104383	1.420839
	1	-3.085355	0.925271	4.006382		6	4.639809	-2.138512	-1.866053
	1	-4.735905	0.405505	4.384596		1	5.667658	-2.462575	-2.057637
	6	-4.808202	2.043145	2.171826		1	4.051866	-3.030002	-1.628184
	1	-5.088887	2.290002	1.143986		1	4.247340	-1.680228	-2.774313
	1	-5.677994	2.201641	2.816175		6	5.438351	0.116619	-1.020588
	1	-4.014167	2.725997	2.487765		1	5.079378	0.590591	-1.934506
	44	0.597869	-1.397871	0.106674		1	5.367762	0.836027	-0.200730
	6	1.468500	-2.388102	2.061109		1	6.491367	-0.149949	-1.156348
	6	0.059131	-2.361809	2.090546		44	0.022619	-1.283489	-0.105218
	6	-0.442446	-3.280775	1.073479		6	-1.115533	-3.200242	-0.643513
	6	0.655621	-3.805632	0.390790		6	-0.580650	-2.573353	-1.809112
	6	1.840549	-3.225475	0.975081		6	0.852513	-2.703596	-1.752896
	1	2.141990	-1.829671	2.694208		6	1.190799	-3.418904	-0.578421
	1	-0.563339	-1.831665	2.794699		6	-0.015489	-3.685771	0.121697
	1	-1.488343	-3.480243	0.903140		1	-2.160286	-3.296325	-0.392884
	1	0.642136	-4.496823	-0.440699		1	-1.152056	-2.141727	-2.617898
	1	2.853871	-3.449345	0.678671		1	1.539678	-2.331372	-2.500624
	53	2.985394	-1.564362	-1.537471		1	2.186966	-3.651647	-0.230560
	6	-0.199028	-0.708835	-1.805271		1	-0.089240	-4.173979	1.083544
	1	0.444192	-1.063329	-2.605509		53	-0.620262	-1.314680	2.560347
	6	0.782195	1.379366	-0.678892		6	-1.572998	1.358700	-0.178331
	6	1.261489	0.566507	0.285574		6	-1.642932	0.023143	-0.336318
	6	1.069932	2.832299	-0.844859		6	-2.778990	2.249274	-0.290959
	6	1.333507	3.356146	-2.124568		6	-2.838865	3.251606	-1.274578
	6	1.069590	3.723344	0.243299		6	-3.848963	2.143379	0.611221
	6	1.598904	4.712696	-2.307997		6	-3.936534	4.107667	-1.364192
	1	1.353147	2.688401	-2.981889		1	-2.022009	3.352942	-1.985517
	6	1.329940	5.081390	0.059345		6	-4.945711	3.002974	0.527289
	1	0.860761	3.345954	1.238467		1	-3.814235	1.383825	1.385609
	6	1.596632	5.583456	-1.215984		6	-4.995672	3.986856	-0.461550
	1	1.810808	5.089098	-3.305321		1	-3.965084	4.868301	-2.140054
	1	1.319579	5.749831	0.916340		1	-5.760566	2.904998	1.239776
	1	1.799554	6.641367	-1.357884		1	-5.849864	4.655121	-0.527338

Species	Cartesian coordinates				Species	Cartesian coordinates			
	6	2.224850	0.950048	1.340570		6	-2.921608	-0.617624	-0.758467
	6	3.549592	1.307147	1.030286		6	-3.338925	-0.520457	-2.098795
	6	1.831698	0.975035	2.692875		6	-3.750927	-1.312933	0.140383
	6	4.444664	1.675084	2.035527		6	-4.537802	-1.094399	-2.525583
	1	3.870013	1.288333	-0.005634		1	-2.719262	0.027322	-2.803826
	6	2.725443	1.349283	3.695975		6	-4.951843	-1.881691	-0.284897
	1	0.810440	0.705335	2.949349		1	-3.444675	-1.401537	1.178293
	6	4.039285	1.698776	3.371577		6	-5.350616	-1.778978	-1.620565
	1	5.463846	1.945411	1.771808		1	-4.838956	-0.999564	-3.565650
	1	2.395689	1.369822	4.731632		1	-5.580380	-2.404433	0.431517
	1	4.738378	1.985884	4.152185		1	-6.284946	-2.224722	-1.950084
**TS5e**	6	-0.896472	-3.051907	-2.042804	**INT4e**	6	-2.273971	-2.075057	-1.429746
	6	-1.521402	-2.087153	-1.225780		6	-2.800097	-0.854554	-1.891609
	6	-2.616675	-2.472923	-0.451569		6	-3.738176	-0.817204	-2.912469
	6	-3.104556	-3.781958	-0.490408		6	-4.171917	-2.036898	-3.457511
	6	-2.502334	-4.728735	-1.318903		6	-3.650845	-3.249659	-2.999758
	6	-1.399972	-4.359923	-2.086637		6	-2.680691	-3.280536	-1.985077
	1	-3.098183	-1.750515	0.195607		1	-4.143174	0.123964	-3.276188
	1	-3.957534	-4.056287	0.124426		1	-4.926994	-2.037711	-4.238971
	1	-2.880533	-5.746495	-1.358836		1	-4.007313	-4.182064	-3.429375
	1	-0.900051	-5.094947	-2.713277		1	-2.277598	-4.227764	-1.636040
	6	0.329251	-2.708727	-2.754258		6	-1.237730	-1.707482	-0.367493
	6	0.942809	-1.526189	-2.620647		6	-0.026427	-1.121012	-1.167169
	6	0.339737	-0.357415	-1.891224		6	-0.600296	0.268345	-1.628916
	6	-1.084929	-0.617505	-1.295154		6	-2.076749	0.224292	-1.097512
	1	0.791617	-3.488753	-3.355533		1	-1.022548	-2.454423	0.387765
	1	1.892556	-1.359541	-3.117022		1	0.146091	-1.769334	-2.029723
	1	0.192167	0.415390	-2.666046		1	-0.612334	0.363412	-2.723026
	1	-1.793734	-0.150188	-1.986808		1	-2.569442	1.186526	-0.990593
	7	-1.184148	0.091924	-0.016598		7	-1.836606	-0.472999	0.188808
	6	-2.325230	0.742223	0.340353		6	-2.763819	-0.541271	1.206866
	8	-2.472354	1.363841	1.398621		8	-2.842216	-1.469156	1.997168
	8	-3.306001	0.652274	-0.606186		8	-3.516107	0.585493	1.226774
	6	-4.634895	1.246812	-0.413062		6	-4.506319	0.821087	2.287224
	6	-4.527890	2.769661	-0.273680		6	-5.072567	2.193643	1.912302
	1	-4.041240	3.042458	0.662402		1	-5.830114	2.500043	2.639934
	1	-3.953956	3.187219	-1.107076		1	-4.278976	2.946401	1.897427
	1	-5.529713	3.211358	-0.295682		1	-5.536273	2.162397	0.921832
	6	-5.334511	0.605464	0.790500		6	-5.600766	-0.250414	2.233969
	1	-4.799637	0.826528	1.714360		1	-6.039692	-0.293985	1.232103
	1	-6.355684	0.992276	0.871496		1	-5.198452	-1.231801	2.485485
	1	-5.393993	-0.480066	0.663253		1	-6.396075	0.000339	2.943676
	6	-5.352793	0.875503	-1.714498		6	-3.812144	0.875826	3.652274
	1	-5.374663	-0.210190	-1.844297		1	-3.384342	-0.092909	3.911410
	1	-6.382275	1.245725	-1.696306		1	-3.013536	1.624346	3.641845
	1	-4.842064	1.315040	-2.576544		1	-4.536678	1.161147	4.422106
	44	0.604403	0.156039	1.274797		44	1.970860	-0.945031	-0.326628
	6	1.856806	1.391677	2.726866		6	4.426446	-1.093437	-0.784214
	6	0.482875	1.798433	2.861769		6	3.831046	-2.367212	-0.809361
	6	-0.257699	0.707467	3.403033		6	2.784654	-2.362161	-1.802644
	6	0.619850	-0.383926	3.540568		6	2.763305	-1.058413	-2.403201
	6	1.941275	0.034999	3.112538		6	3.742108	-0.271754	-1.731803
	1	2.672551	2.012049	2.380923		1	5.211639	-0.767595	-0.114885
	1	0.100698	2.789501	2.675961		1	4.088252	-3.205517	-0.176312
	1	-1.324456	0.702559	3.569543		1	2.191221	-3.215064	-2.103894
	1	0.360361	-1.374315	3.884695		1	2.142641	-0.744080	-3.230493
	1	2.829705	-0.579387	3.131435		1	3.957626	0.768960	-1.926890
	53	1.251200	-2.595095	1.045116		53	1.421997	-2.320831	1.924543
	6	1.264191	0.376301	-0.860419		6	0.200922	1.384406	-1.014551
	6	0.642254	1.452638	-0.198160		6	1.371802	1.028094	-0.428877
	6	2.721188	0.519362	-1.236741		6	-0.340881	2.768354	-1.051262
	6	3.096265	0.850510	-2.554590		6	-0.922193	3.269680	-2.232298
	6	3.740359	0.334289	-0.293191		6	-0.321631	3.600089	0.083074
	6	4.437414	1.006421	-2.903666		6	-1.436469	4.564575	-2.284498
	1	2.336477	0.991357	-3.318040		1	-0.956012	2.647136	-3.121856
	6	5.083482	0.489106	-0.642248		6	-0.847992	4.889862	0.032692
	1	3.469718	0.032315	0.710666		1	0.092999	3.222356	1.011115
	6	5.439296	0.829617	-1.946779		6	-1.402364	5.380542	-1.151605
	1	4.697611	1.263873	-3.926728		1	-1.866851	4.935116	-3.210921
	1	5.852091	0.330398	0.109103		1	-0.829208	5.511343	0.923744

Species	Cartesian coordinates				Species	Cartesian coordinates			
	1	6.484220	0.947008	-2.219137		1	-1.810275	6.386688	-1.189677
	6	0.398443	2.849680	-0.462640		6	2.372055	1.931326	0.173533
	6	1.112715	3.502153	-1.498057		6	2.771899	1.761761	1.515101
	6	-0.566122	3.589088	0.262330		6	2.995710	2.946803	-0.584375
	6	0.899074	4.849586	-1.763611		6	3.757796	2.576314	2.076162
	1	1.857636	2.955001	-2.061101		1	2.288743	1.000055	2.120027
	6	-0.799182	4.926632	-0.039450		6	3.976632	3.757405	-0.020674
	1	-1.167377	3.081351	1.006615		1	2.689761	3.099136	-1.615527
	6	-0.058877	5.565152	-1.038777		6	4.364732	3.573063	1.311659
	1	1.473121	5.341551	-2.543219		1	4.045624	2.432645	3.113848
	1	-1.558039	5.475715	0.510092		1	4.439200	4.538266	-0.618340
	1	-0.231356	6.615485	-1.256465		1	5.130865	4.207257	1.748651
**TS6e**	6	2.440839	-2.358925	-1.031537	**INT5e**	6	2.653893	1.760408	1.666638
	6	2.700749	-2.011505	0.308129		6	2.383464	2.262299	0.382119
	6	3.666259	-2.670417	1.049916		6	3.067013	3.363929	-0.113481
	6	4.400232	-3.690055	0.416896		6	4.041580	3.957887	0.703103
	6	4.143533	-4.036668	-0.909856		6	4.305516	3.463337	1.983380
	6	3.145543	-3.377600	-1.650735		6	3.606864	2.354209	2.482882
	1	3.865862	-2.407266	2.085480		1	2.861604	3.758401	-1.104937
	1	5.178964	-4.210568	0.967343		1	4.600444	4.813101	0.333615
	1	4.726572	-4.823629	-1.380347		1	5.068349	3.937010	2.594691
	1	2.950820	-3.654692	-2.683288		1	3.821696	1.967794	3.475713
	6	1.283829	-1.448016	-1.470721		6	1.743775	0.560149	1.855353
	6	0.072999	-1.988032	-0.674317		6	0.244117	0.879595	1.947257
	6	0.329180	-1.593073	0.704524		6	-0.010557	1.824861	0.758957
	6	1.712531	-0.882834	0.636889		6	1.227295	1.437975	-0.147959
	1	1.152166	-1.258427	-2.530957		1	2.116381	-0.221652	2.511098
	1	-0.192958	-3.020692	-0.885150		1	-0.187838	1.167188	2.904082
	1	0.208670	-2.326243	1.492583		1	0.099339	2.877557	1.046597
	1	1.967165	-0.213722	1.452699		1	1.052541	1.406376	-1.219585
	7	1.581186	-0.232609	-0.679629		7	1.557166	0.068276	0.428150
	6	2.463593	0.713751	-1.159619		6	2.677187	-0.667021	-0.108554
	8	2.739852	0.844711	-2.339648		8	3.282166	-1.470532	0.570153
	8	2.919909	1.483109	-0.140857		8	2.900488	-0.325904	-1.367838
	6	3.867563	2.583621	-0.393202		6	4.014629	-0.925998	-2.155350
	6	3.221003	3.638691	-1.296425		6	3.830834	-0.269283	-3.523705
	1	3.039823	3.240428	-2.294698		1	4.608882	-0.618511	-4.208658
	1	2.267623	3.970441	-0.873824		1	2.855026	-0.524876	-3.945189
	1	3.882283	4.508019	-1.374829		1	3.902855	0.819301	-3.444435
	6	4.111272	3.143697	1.010832		6	5.343720	-0.517173	-1.516195
	1	4.518131	2.371649	1.670608		1	5.405382	0.571215	-1.421775
	1	4.825484	3.971121	0.964904		1	5.461844	-0.966589	-0.529146
	1	3.180677	3.517497	1.448539		1	6.168936	-0.852731	-2.152062
	6	5.168434	2.028072	-0.983150		6	3.843196	-2.443170	-2.244177
	1	5.576353	1.245963	-0.334867		1	3.980204	-2.916777	-1.271854
	1	5.001423	1.612053	-1.976719		1	2.848049	-2.693542	-2.620212
	1	5.909476	2.830668	-1.056155		1	4.589740	-2.843449	-2.937911
	44	-1.802709	-1.008766	-0.235723		44	-0.335155	-1.048385	1.107707
	6	-0.639351	-0.080799	1.513201		6	-0.416052	-3.342873	1.730053
	6	-1.169439	0.801629	0.714986		6	0.541575	-2.786219	2.596643
	6	-4.116133	-0.949509	0.306260		6	-0.084631	-1.710499	3.294013
	6	-3.950949	-1.370173	-1.037947		6	-1.468548	-1.654883	2.912025
	6	-3.209573	-2.602063	-1.056474		6	-1.669299	-2.643578	1.907294
	6	-2.886190	-2.917904	0.291894		1	-0.244223	-4.142162	1.023093
	6	-3.423140	-1.878398	1.130739		1	1.585544	-3.063361	2.654951
	1	-4.638336	-0.064201	0.641267		1	0.382044	-1.088134	4.046149
	1	-4.328068	-0.857328	-1.911169		1	-2.215231	-0.999528	3.336192
	1	-2.973221	-3.185547	-1.935265		1	-2.600124	-2.872831	1.409127
	1	-2.356667	-3.798138	0.633592		53	-0.377855	-2.124188	-1.507247
	1	-3.353225	-1.834889	2.209435		6	-1.394658	1.571293	0.215147
	53	-1.482194	0.351071	-2.667593		6	-1.811973	0.291469	0.349868
	6	-0.423994	-0.201511	2.960974		6	-2.141373	2.727684	-0.352554
	6	0.052739	0.905376	3.692246		6	-1.453895	3.708611	-1.094649
	6	-0.715764	-1.383854	3.667664		6	-3.520821	2.921343	-0.146481
	6	0.222419	0.828460	5.073143		6	-2.113713	4.816658	-1.625971
	1	0.282161	1.828384	3.172966		1	-0.387390	3.599443	-1.270764
	6	-0.543828	-1.455611	5.049712		6	-4.180342	4.030564	-0.673710
	1	-1.100997	-2.248876	3.136933		1	-4.077969	2.201631	0.441109
	6	-0.070949	-0.351674	5.760635		6	-3.483804	4.983913	-1.419959
	1	0.590838	1.696363	5.613204		1	-1.554458	5.549928	-2.201392

Species	Cartesian coordinates				Species	Cartesian coordinates			
	1	-0.783385	-2.378536	5.570663		1	-5.245087	4.153722	-0.492828
	1	0.067097	-0.409512	6.836277		1	-4.000470	5.847531	-1.829402
	6	-1.433054	2.215390	0.609811		6	-3.158024	-0.189526	-0.051535
	6	-2.690961	2.707642	0.210831		6	-4.125318	-0.540186	0.909002
	6	-0.410038	3.142261	0.906826		6	-3.524811	-0.272588	-1.407909
	6	-2.935739	4.076958	0.173935		6	-5.402467	-0.960174	0.533291
	1	-3.466383	2.004981	-0.072184		1	-3.882484	-0.456691	1.963976
	6	-0.655904	4.511985	0.849346		6	-4.799521	-0.693699	-1.784223
	1	0.582773	2.771562	1.140165		1	-2.797589	-0.002582	-2.164880
	6	-1.922071	4.984837	0.494967		6	-5.745119	-1.043306	-0.817604
	1	-3.915908	4.439732	-0.122343		1	-6.130940	-1.216527	1.298367
	1	0.143161	5.212261	1.076839		1	-5.054506	-0.748500	-2.839341
	1	-2.113127	6.053276	0.451734		1	-6.737208	-1.373367	-1.113035
**TS7e**	6	-0.952355	-2.891605	-0.436433	**INT6e**	6	3.200188	2.343625	-0.697861
	6	-1.852518	-2.439459	-1.420917		6	2.653915	1.216224	-1.350687
	6	-2.392677	-3.317413	-2.348305		6	3.408686	0.540289	-2.312822
	6	-2.033909	-4.672665	-2.261736		6	4.696897	0.969481	-2.642222
	6	-1.138360	-5.118379	-1.286201		6	5.239219	2.085873	-2.002023
	6	-0.570322	-4.223893	-0.365473		6	4.495945	2.764246	-1.036018
	1	-3.085284	-2.977956	-3.114019		1	2.981845	-0.327058	-2.810433
	1	-2.463065	-5.385497	-2.960402		1	5.269456	0.437441	-3.396241
	1	-0.881776	-6.172801	-1.235823		1	6.238341	2.429027	-2.256131
	1	0.132163	-4.570113	0.387264		1	4.915697	3.635746	-0.539071
	6	-0.537492	-1.640426	0.350968		6	2.407007	3.039115	0.316115
	6	0.382100	-0.930998	-0.670215		6	1.093654	2.810620	0.454446
	6	-0.612335	-0.305551	-1.678165		6	0.355952	1.886185	-0.496315
	6	-1.970280	-0.932310	-1.212306		6	1.254191	0.728981	-1.004990
	1	-0.161724	-1.768830	1.360282		1	2.914423	3.762918	0.950191
	1	1.151803	-1.682416	-1.080266		1	0.515780	3.344848	1.201109
	1	-0.401047	-0.506152	-2.733362		1	0.189138	2.487034	-1.407843
	1	-2.868172	-0.432811	-1.566019		1	0.785238	0.371140	-1.927816
	7	-1.763212	-0.830827	0.248594		7	1.226139	-0.464609	-0.126434
	6	0.636873	1.172284	-0.381746		6	2.434120	-0.904118	0.514042
	6	-0.442843	1.117134	-1.200373		8	2.955747	-1.968416	0.246300
	6	-2.739181	-0.871498	1.217026		8	2.829958	-0.033547	1.441551
	8	-2.525132	-1.196489	2.374793		6	4.064775	-0.258533	2.243717
	8	-3.916857	-0.451436	0.704315		6	4.133042	1.004099	3.103264
	6	-5.087126	-0.223186	1.569018		1	3.230635	1.107287	3.712523
	6	-6.135821	0.286471	0.576915		1	4.998490	0.950799	3.770325
	1	-7.072674	0.502502	1.099038		1	4.229457	1.893566	2.476329
	1	-5.789985	1.201718	0.087740		6	3.883841	-1.503718	3.115958
	1	-6.334734	-0.464044	-0.193900		1	4.751740	-1.614381	3.773635
	6	-4.761453	0.849865	2.612938		1	2.996110	-1.403720	3.749234
	1	-4.001480	0.498722	3.311636		1	3.796019	-2.403926	2.506228
	1	-4.399578	1.757969	2.120457		6	5.282278	-0.365248	1.322517
	1	-5.666728	1.103116	3.174125		1	5.240787	-1.266460	0.710523
	6	-5.534278	-1.542329	2.206730		1	5.345612	0.506703	0.666510
	1	-5.712593	-2.296384	1.433556		1	6.188888	-0.400707	1.935331
	1	-4.779519	-1.917448	2.898069		44	-0.364279	-1.506710	0.169413
	1	-6.470513	-1.387205	2.752869		6	-1.970057	-2.841049	1.169256
	44	2.375195	-0.252366	-0.658118		6	-1.152044	-3.721829	0.408608
	6	3.050433	1.361202	-2.074390		6	0.166885	-3.715609	0.953098
	6	3.843518	1.379931	-0.867575		6	0.187337	-2.782539	2.018054
	6	4.601357	0.162590	-0.828971		6	-1.137289	-2.236249	2.157053
	6	4.209709	-0.628093	-1.931562		1	-3.021567	-2.653353	1.014662
	6	3.245715	0.116030	-2.713927		1	-1.474898	-4.301660	-0.445177
	1	2.393715	2.148608	-2.413637		1	1.017756	-4.261528	0.568348
	1	3.912531	2.197134	-0.164322		1	1.039937	-2.542647	2.638173
	1	5.284734	-0.132046	-0.045254		1	-1.457816	-1.536883	2.914803
	1	4.555716	-1.631193	-2.145570		53	-1.135938	-1.668374	-2.474711
	1	2.788265	-0.212204	-3.637568		6	-1.038382	1.508077	-0.003796
	53	2.802765	-1.427683	1.838942		6	-1.533050	0.282698	0.258862
	6	-1.470118	2.150634	-1.464325		6	-2.948807	0.106033	0.689335
	6	-1.995954	2.277338	-2.763966		6	-3.321589	0.274265	2.035902
	6	-1.987445	2.982253	-0.454285		6	-3.954650	-0.228372	-0.234784
	6	-2.984799	3.218118	-3.051235		6	-4.644810	0.106577	2.446170
	1	-1.618224	1.638571	-3.558425		1	-2.564501	0.553644	2.763333
	6	-2.979066	3.918802	-0.742208		6	-5.278234	-0.392668	0.174454
	1	-1.617662	2.882510	0.559747		1	-3.686619	-0.365565	-1.276830
	6	-3.479843	4.044740	-2.040821		6	-5.630567	-0.231082	1.516904

Species	Cartesian coordinates				Species	Cartesian coordinates			
	1	-3.369423	3.303448	-4.063981		1	-4.905807	0.246689	3.491951
	1	-3.366563	4.549470	0.053398		1	-6.037779	-0.646419	-0.560405
	1	-4.252607	4.775825	-2.261185		1	-6.661378	-0.362597	1.833821
	6	1.009282	2.273360	0.532684		6	-1.927071	2.727110	0.097112
	6	1.313383	3.551855	0.024314		6	-2.479443	3.276697	-1.070907
	6	1.033287	2.083763	1.926107		6	-2.192999	3.360663	1.319749
	6	1.627657	4.604656	0.884016		6	-3.280410	4.418037	-1.017106
	1	1.278410	3.725470	-1.046913		1	-2.287663	2.795572	-2.026775
	6	1.344728	3.140063	2.780497		6	-2.990701	4.505786	1.375807
	1	0.816641	1.101936	2.332657		1	-1.773599	2.951084	2.234219
	6	1.646449	4.403718	2.265706		6	-3.537728	5.038159	0.207596
	1	1.854994	5.583864	0.471031		1	-3.705112	4.821510	-1.932553
	1	1.355423	2.971738	3.853730		1	-3.184870	4.980104	2.334261
	1	1.894313	5.222978	2.934595		1	-4.159982	5.927843	0.250557
**TS8e**	6	2.502038	1.596726	1.908298	**INT8e**	6	-1.884433	0.243801	2.048400
	6	2.362037	2.080469	0.587040		6	-1.223101	1.178999	1.084724
	6	3.238883	3.036587	0.101337		6	0.175354	1.437107	1.708967
	6	4.271258	3.499223	0.932090		6	0.101767	-1.136780	1.547845
	6	4.435567	2.996243	2.227343		6	-1.208933	-0.970868	2.284668
	6	3.558810	2.028637	2.721494		7	-0.139794	-1.170427	0.086138
	1	3.144413	3.403101	-0.916570		1	-1.793254	2.100536	0.947590
	1	4.967960	4.242558	0.554621		1	0.599064	-2.055728	1.850581
	1	5.251995	3.353720	2.847371		6	-1.718027	-1.905237	3.184245
	1	3.673951	1.639462	3.729618		1	-1.188115	-2.840324	3.351574
	6	1.470474	0.662870	2.325171		6	-2.900457	-1.631658	3.877782
	6	-0.053435	1.744112	0.734874		1	-3.296964	-2.356032	4.583762
	6	1.219976	1.358215	-0.097384		6	-3.565374	-0.421282	3.665167
	1	1.713059	-0.011105	3.145598		1	-4.480951	-0.203529	4.208660
	1	0.051907	2.802402	1.011730		6	-3.064481	0.509475	2.753191
	1	1.125114	1.574721	-1.163980		1	-3.592506	1.443829	2.583191
	7	1.409354	-0.095686	0.173178		6	0.947125	0.100481	1.943903
	6	2.524637	-0.774926	-0.263362		6	2.057876	0.698771	1.090240
	8	2.824174	-1.900767	0.118194		6	1.431793	1.910521	0.985892
	8	3.250015	-0.032518	-1.136213		1	1.241009	-0.026908	2.993641
	6	4.524802	-0.524476	-1.687157		1	-0.000851	1.994851	2.642361
	6	5.535418	-0.737812	-0.554864		1	1.394396	0.228494	-2.471284
	1	5.219283	-1.549279	0.101104		6	0.405534	0.658148	-2.550780
	1	6.513886	-0.987235	-0.978403		6	-0.051828	1.847530	-1.882281
	1	5.642843	0.177235	0.036351		6	-0.655952	0.146567	-3.331176
	6	4.290673	-1.796869	-2.507230		6	-1.421894	2.037664	-2.228930
	1	3.958453	-2.616471	-1.870561		1	0.550998	2.523637	-1.300085
	1	3.531074	-1.619097	-3.274174		44	-1.178304	0.206134	-0.905918
	1	5.222019	-2.085881	-3.005832		6	-1.790979	0.966310	-3.099932
	6	4.963098	0.628888	-2.593978		1	-0.634411	-0.767016	-3.906794
	1	5.099627	1.546493	-2.014533		1	-2.056857	2.853323	-1.912815
	1	5.910827	0.382320	-3.082106		53	-3.902524	-0.106211	-0.697231
	1	4.212966	0.815295	-3.367919		1	-2.777952	0.804781	-3.509678
	44	-0.345467	-1.107109	0.911828		1	1.293225	-6.077103	-1.352836
	6	0.090612	0.829413	1.945838		6	0.827243	-5.137173	-1.040104
	1	-0.632558	0.914686	2.757834		6	1.907554	-4.186629	-0.515852
	6	-1.598118	-2.899458	1.392771		1	0.102491	-5.363481	-0.251830
	6	-0.266251	-3.438063	1.200738		1	0.300561	-4.707407	-1.892306
	6	0.568069	-2.949249	2.224530		8	1.276652	-2.979365	0.062685
	6	-0.209179	-2.062360	3.022132		6	2.624074	-4.791360	0.693986
	6	-1.564024	-2.061608	2.535143		6	2.914763	-3.786357	-1.597912
	1	-2.468470	-3.130055	0.796602		6	0.497408	-2.168641	-0.677360
	1	0.033492	-4.096565	0.398206		1	1.909225	-5.039893	1.484031
	1	1.630697	-3.126179	2.308529		1	3.144689	-5.707293	0.399334
	1	0.137370	-1.529214	3.898520		1	3.358212	-4.087590	1.095421
	1	-2.402297	-1.534902	2.969469		1	2.410621	-3.352037	-2.461032
	53	-0.448343	-1.816970	-1.882526		1	3.633519	-3.063376	-1.201493
	6	-1.422252	1.496491	0.135538		1	3.467040	-4.674541	-1.922338
	6	-1.883930	0.234000	0.297849		8	0.326190	-2.296292	-1.881757
	6	-3.316623	-0.142142	0.248345		6	1.800517	3.284610	0.638464
	6	-3.855107	-1.170970	-0.548128		6	0.806818	4.285131	0.669858
	6	-4.203238	0.550896	1.101837		6	3.121940	3.678230	0.341291
	6	-5.215790	-1.475273	-0.506905		6	1.113625	5.616540	0.393069
	1	-3.199232	-1.716704	-1.216450		1	-0.214380	4.010754	0.917567
	6	-5.561455	0.240271	1.148001		6	3.426531	5.009404	0.069943
	1	-3.810013	1.346416	1.727600		1	3.915167	2.939541	0.342060

Species	Cartesian coordinates				Species	Cartesian coordinates			
	6	-6.077418	-0.776005	0.341539		6	2.425009	5.985061	0.088106
	1	-5.604346	-2.264638	-1.145360		1	0.328216	6.366773	0.420886
	1	-6.216972	0.795182	1.814350		1	4.452903	5.289057	-0.151654
	1	-7.135542	-1.020663	0.374442		1	2.666980	7.021905	-0.127058
	6	-2.144978	2.612868	-0.530372		6	3.338042	0.112536	0.691201
	6	-2.114425	3.927811	-0.026881		6	3.934360	-0.875052	1.499048
	6	-2.866265	2.384398	-1.717798		6	3.991185	0.474111	-0.503655
	6	-2.786846	4.964839	-0.673564		6	5.153236	-1.451151	1.144916
	1	-1.578496	4.143975	0.893399		1	3.442926	-1.177630	2.419026
	6	-3.538549	3.420199	-2.363952		6	5.203933	-0.111612	-0.863191
	1	-2.881358	1.384047	-2.137553		1	3.535479	1.210438	-1.158716
	6	-3.503196	4.717043	-1.846141		6	5.794682	-1.072173	-0.037653
	1	-2.752972	5.968482	-0.257436		1	5.604730	-2.196926	1.793628
	1	-4.083429	3.214751	-3.281622		1	5.687371	0.179804	-1.791616
	1	-4.023158	5.525489	-2.352644		1	6.742944	-1.523495	-0.315025
**TS9e**	6	0.296097	3.996714	-0.774648	**INT9e**	6	-2.259066	0.906973	-2.148542
	6	1.021898	2.880339	-0.308056		6	-1.565469	-0.351650	-2.409194
	6	1.942409	3.057899	0.726634		6	-1.573690	1.911420	-1.434517
	6	2.135571	4.313813	1.310875		6	-0.228572	1.528598	-0.855814
	6	1.409958	5.416526	0.855443		7	-0.401605	0.568456	0.251563
	6	0.497743	5.254860	-0.185505		1	-2.139073	-1.159758	-2.855519
	1	2.513981	2.206133	1.078508		1	0.306490	2.414014	-0.507645
	1	2.858213	4.429308	2.114046		6	-2.172866	3.153225	-1.246807
	1	1.558545	6.395014	1.303206		1	-1.650207	3.919603	-0.681554
	1	-0.070808	6.107101	-0.550560		6	-3.442813	3.407065	-1.775882
	6	-0.658890	3.820723	-1.868386		1	-3.902737	4.380739	-1.631469
	6	-1.014042	2.612397	-2.332214		6	-4.126069	2.411092	-2.478810
	6	-0.422729	1.326136	-1.819481		1	-5.115891	2.608317	-2.879772
	6	0.901332	1.535131	-1.027491		6	-3.541715	1.158167	-2.655004
	1	-1.095131	4.718262	-2.302509		1	-4.071179	0.375433	-3.191468
	1	-1.728284	2.537401	-3.147018		44	-0.466656	-1.396316	-0.379373
	1	-0.139523	0.757200	-2.718099		6	0.613578	0.796964	-1.952034
	1	1.705276	1.539328	-1.770965		1	0.787503	1.484238	-2.790113
	7	1.094666	0.378932	-0.155683		6	1.880134	0.301898	-1.309919
	6	2.298586	-0.266116	-0.111808		6	1.691244	-0.743696	-0.480447
	8	2.521320	-1.302193	0.502342		6	-0.180355	-0.418177	-2.384837
	8	3.261456	0.393866	-0.838629		1	0.335324	-1.172100	-2.972919
	6	4.626292	-0.133232	-0.951200		6	-1.556994	-3.461780	-0.358193
	6	4.616193	-1.507779	-1.630183		6	-1.044089	-3.270432	0.990689
	1	4.127097	-2.249691	-0.999620		6	-0.462065	-3.561995	-1.240945
	1	4.088138	-1.453187	-2.587771		6	0.346089	-3.198508	0.912817
	1	5.644657	-1.828495	-1.827157		6	0.715567	-3.341713	-0.484702
	6	5.296966	-0.174433	0.427093		1	-2.598156	-3.571718	-0.619604
	1	4.792433	-0.883695	1.082968		1	-1.647106	-3.173000	1.881135
	1	6.345260	-0.472092	0.318399		1	-0.514521	-3.722292	-2.309500
	1	5.273638	0.817729	0.889799		1	1.028026	-3.054681	1.735394
	6	5.308788	0.903949	-1.849117		1	1.725149	-3.389619	-0.861822
	1	5.269524	1.896137	-1.390015		53	-3.424929	-0.971547	0.547964
	1	6.357813	0.635136	-2.006400		6	-0.284725	0.941830	1.551131
	1	4.815403	0.956085	-2.824469		8	-0.191103	0.150834	2.492020
	44	-0.723190	-0.057413	1.033363		8	-0.302505	2.299969	1.698263
	6	-2.153983	0.011403	2.825728		6	-0.250839	2.929021	3.022743
	6	-0.908351	0.613966	3.269712		6	-0.316018	4.422055	2.685260
	6	-0.699935	1.784299	2.522892		1	-1.242415	4.655273	2.152034
	6	-1.756687	1.897227	1.568139		1	-0.285727	5.018102	3.602418
	6	-2.686855	0.818186	1.805617		1	0.529770	4.713912	2.055150
	1	-2.592359	-0.889978	3.226834		6	-1.467757	2.511855	3.855816
	1	-0.259248	0.219162	4.037377		1	-2.391541	2.731971	3.312033
	1	0.156935	2.438206	2.588468		1	-1.438846	1.445349	4.078383
	1	-1.884684	2.708002	0.866175		1	-1.480818	3.073640	4.795935
	1	-3.610217	0.646639	1.273179		6	1.072596	2.594969	3.720970
	53	-0.074413	-2.546169	2.333138		1	1.125427	1.534271	3.965593
	6	-1.358975	0.317162	-1.057420		1	1.917982	2.854278	3.075059
	6	-0.705116	-0.908720	-0.762378		1	1.160089	3.177158	4.644448
	6	-2.842869	0.297250	-1.298327		6	3.134371	1.087111	-1.494570
	6	-3.534642	-0.928489	-1.396548		6	3.547496	1.439951	-2.792580
	6	-3.607228	1.477334	-1.384648		6	3.912821	1.528183	-0.410828
	6	-4.912157	-0.970856	-1.600675		6	4.703267	2.191602	-3.001519
	1	-2.993756	-1.863495	-1.302683		1	2.964005	1.110039	-3.648382
	6	-4.986808	1.433647	-1.590253		6	5.065806	2.285168	-0.619300

Species	Cartesian coordinates				Species	Cartesian coordinates			
	1	-3.126312	2.440312	-1.276302		1	3.606668	1.282033	0.599941
	6	-5.648506	0.210947	-1.705599		6	5.468418	2.618420	-1.914091
	1	-5.409783	-1.933716	-1.674549		1	5.005512	2.443988	-4.014413
	1	-5.544651	2.364100	-1.651526		1	5.648081	2.620175	0.234903
	1	-6.722428	0.178582	-1.864692		1	6.366738	3.208041	-2.074284
	6	-0.253572	-1.977251	-1.611707		6	2.761370	-1.334273	0.352177
	6	0.610732	-3.008943	-1.176437		6	3.853412	-1.978713	-0.260779
	6	-0.697904	-1.989970	-2.962272		6	2.749044	-1.229279	1.757372
	6	1.005056	-4.009792	-2.059562		6	4.902861	-2.493339	0.501481
	1	0.981324	-2.988996	-0.161968		1	3.883129	-2.053437	-1.344485
	6	-0.316436	-3.005749	-3.826996		6	3.802763	-1.742236	2.514190
	1	-1.363540	-1.208609	-3.313400		1	1.904955	-0.745529	2.241843
	6	0.539582	-4.018465	-3.376548		6	4.882259	-2.376798	1.892348
	1	1.675521	-4.791227	-1.714716		1	5.737854	-2.981821	0.006439
	1	-0.676709	-3.009428	-4.851452		1	3.780795	-1.643860	3.596331
	1	0.843955	-4.810634	-4.055304		1	5.698985	-2.776336	2.486959
**TS11e**	6	1.307297	0.244964	2.463083	**INT10e**	6	0.128176	-2.085664	2.177313
	6	0.161201	-0.400503	1.853460		6	-0.884714	-1.434878	1.288707
	6	-1.128203	0.291339	2.169549		6	-1.120685	-0.029362	1.901109
	6	1.367064	1.644610	2.237299		6	1.440404	-0.078350	1.581402
	6	0.353705	2.054988	1.177505		6	1.347277	-1.389335	2.338728
	7	0.508845	1.107331	0.060613		7	1.347154	-0.323616	0.128242
	1	0.204614	-1.454140	1.593758		1	-1.811687	-2.008064	1.230042
	1	0.492557	3.087275	0.843530		1	2.378754	0.429169	1.801293
	6	2.356006	2.402417	2.842704		6	2.358669	-1.909212	3.140427
	1	2.424090	3.470278	2.655321		1	3.293391	-1.364644	3.254172
	6	3.297548	1.765898	3.667462		6	2.175509	-3.135751	3.793068
	1	4.079189	2.354660	4.139773		1	2.967497	-3.544171	4.414388
	6	3.267070	0.381578	3.856978		6	0.972895	-3.828490	3.642528
	1	4.018684	-0.096536	4.477721		1	0.825087	-4.776021	4.153956
	6	2.281468	-0.393952	3.241687		6	-0.048612	-3.304823	2.841890
	1	2.247829	-1.469342	3.381848		1	-0.988688	-3.842398	2.739100
	44	0.319305	-1.164857	-1.264816		6	0.223769	0.754912	2.045890
	6	-1.026215	1.808220	1.796552		6	-0.419218	1.831598	1.187751
	6	-2.262259	1.556468	0.937867		6	-1.636632	1.207313	1.169908
	6	-2.399222	0.255612	1.336484		1	0.406007	1.077477	3.079300
	1	-1.193850	2.480701	2.644843		1	-1.629528	-0.191007	2.864641
	1	-1.293921	0.111764	3.245974		1	1.628481	-3.615944	0.585653
	6	-0.420772	-0.962619	-3.319720		6	1.195780	-3.442369	-0.387888
	6	-1.510054	-0.683054	-2.449237		6	1.881109	-2.932235	-1.541359
	6	0.024848	-2.308090	-3.069318		6	-0.138231	-3.782095	-0.788083
	6	-1.764410	-1.866405	-1.664952		6	1.000613	-2.958269	-2.636868
	6	-0.839269	-2.866968	-2.057883		1	2.891979	-2.551356	-1.555973
	1	0.028756	-0.268576	-4.017913		44	0.031304	-1.574258	-0.712422
	1	-2.041637	0.255519	-2.379132		6	-0.252441	-3.447180	-2.173987
	1	0.825333	-2.824555	-3.580711		1	-0.889835	-4.263457	-0.178103
	1	-2.530798	-1.976931	-0.913019		1	1.205480	-2.577612	-3.627937
	1	-0.764644	-3.863373	-1.645504		53	-2.393234	-0.909587	-1.721308
	53	2.102537	-2.941329	0.032260		1	-1.142104	-3.565400	-2.775061
	6	1.632112	1.186424	-0.661785		6	4.288787	1.639707	-0.881866
	8	1.931194	0.283419	-1.491045		8	3.234249	0.936550	-0.124093
	8	2.398580	2.279754	-0.485743		6	2.286894	0.200299	-0.743093
	6	3.611207	2.544230	-1.286789		8	2.244774	0.007370	-1.957063
	6	4.662456	1.465912	-1.010914		6	5.194108	0.614095	-1.571281
	1	4.334424	0.491825	-1.374118		1	4.650236	0.074650	-2.347248
	1	5.599434	1.733533	-1.510427		1	6.045881	1.126558	-2.030087
	1	4.856479	1.392410	0.063421		1	5.583542	-0.103202	-0.841449
	6	3.250603	2.648734	-2.771801		6	5.046361	2.379837	0.223517
	1	2.914936	1.688350	-3.163460		1	5.879125	2.943896	-0.206880
	1	2.459474	3.390682	-2.920498		1	4.382083	3.078676	0.739257
	1	4.129958	2.971797	-3.338307		1	5.449261	1.674586	0.956734
	6	4.069355	3.901118	-0.745188		6	3.670739	2.631805	-1.871122
	1	4.981992	4.219749	-1.257282		1	4.468446	3.228736	-2.325796
	1	3.299466	4.662104	-0.904187		1	3.126112	2.114418	-2.660709
	1	4.278243	3.836546	0.326496		1	2.987801	3.311623	-1.354671
	6	-2.945885	2.504890	0.061222		6	0.148529	3.093904	0.713095
	6	-2.722799	3.884662	0.235475		6	1.125212	3.756546	1.482175
	6	-3.793063	2.099332	-0.989829		6	-0.221881	3.661439	-0.521715
	6	-3.338650	4.822429	-0.591190		6	1.678404	4.962305	1.053799
	1	-2.067228	4.222493	1.032844		1	1.434987	3.329355	2.431842

Species	Cartesian coordinates				Species	Cartesian coordinates			
	6	-4.402630	3.037686	-1.819354		6	0.337641	4.863710	-0.952100
	1	-3.962717	1.042321	-1.163979		1	-0.934962	3.139465	-1.152432
	6	-4.182746	4.403812	-1.621802		6	1.284147	5.524243	-0.163883
	1	-3.157102	5.881704	-0.431946		1	2.416076	5.466556	1.672472
	1	-5.047774	2.701393	-2.626291		1	0.039670	5.281643	-1.909830
	1	-4.660454	5.133776	-2.268859		1	1.715909	6.463243	-0.498574
	6	-3.413806	-0.796325	1.304311		6	-3.026503	1.606355	0.943708
	6	-3.070100	-2.102096	1.709975		6	-4.056769	0.692442	1.240258
	6	-4.758542	-0.542627	0.965499		6	-3.396705	2.904528	0.538895
	6	-4.021807	-3.120064	1.741040		6	-5.397488	1.051509	1.122740
	1	-2.045684	-2.324337	1.992762		1	-3.798088	-0.311653	1.560122
	6	-5.708594	-1.560408	1.001979		6	-4.737462	3.261761	0.418675
	1	-5.063600	0.462363	0.696405		1	-2.630805	3.641532	0.328792
	6	-5.345248	-2.855360	1.382619		6	-5.745929	2.338008	0.706490
	1	-3.728241	-4.119995	2.047632		1	-6.171383	0.324622	1.354128
	1	-6.739786	-1.339695	0.740364		1	-4.996564	4.268941	0.103122
	1	-6.088007	-3.647450	1.407137		1	-6.790895	2.619402	0.610190
**TS12e**	6	2.135576	0.454404	2.237180	**INT11e**	6	2.425929	1.649758	2.183813
	6	1.407727	-0.816290	1.999882		6	1.206548	1.178457	2.772997
	6	1.599678	1.635925	1.686928		6	2.440530	1.938078	0.790020
	6	0.292542	1.489902	0.934699		6	1.243680	1.478923	-0.034038
	7	0.404802	0.713423	-0.310845		7	1.309283	0.007742	-0.039041
	1	1.843581	-1.690946	2.479350		1	1.220860	0.879149	3.818924
	1	-0.123228	2.470391	0.705798		1	1.281474	1.903452	-1.043165
	6	2.233716	2.854637	1.904629		6	3.579063	2.526703	0.238264
	1	1.815947	3.761643	1.473985		1	3.596279	2.755633	-0.821407
	6	3.402545	2.914032	2.671417		6	4.682468	2.822552	1.039630
	1	3.894272	3.868204	2.838533		1	5.550266	3.306749	0.599954
	6	3.932633	1.745759	3.221921		6	4.687204	2.498405	2.405229
	1	4.840142	1.788161	3.817959		1	5.559516	2.716379	3.013736
	6	3.302441	0.518826	3.006140		6	3.568164	1.907750	2.972181
	1	3.721271	-0.392238	3.426203		1	3.548541	1.665572	4.031877
	44	0.980630	-1.255537	-0.084824		44	-0.183265	-0.966351	1.018790
	6	-0.646108	0.705232	1.897997		6	-0.034243	1.909217	0.719550
	1	-0.743851	1.246664	2.846914		1	0.034279	2.998341	0.869787
	6	-1.934529	0.253562	1.287352		6	-1.396523	1.564684	0.131410
	6	-1.640056	-0.992167	0.872209		6	-1.796442	0.291909	0.336943
	6	-0.043091	-0.689808	2.081739		6	0.012433	1.222977	2.079951
	1	-0.521577	-1.335163	2.814764		1	-0.911140	1.180295	2.648301
	6	2.255546	-2.928364	-1.157776		6	0.460108	-1.940528	3.020989
	6	1.300898	-2.384421	-2.104916		6	0.918197	-2.881890	2.071097
	6	1.553238	-3.538798	-0.099674		6	-0.984462	-1.840980	2.924568
	6	0.016477	-2.686994	-1.635173		6	-0.211578	-3.327095	1.348332
	6	0.169711	-3.352846	-0.360595		6	-1.397399	-2.696495	1.880241
	1	3.328157	-2.891942	-1.263742		1	1.079502	-1.430470	3.744796
	1	1.545156	-1.835602	-3.002032		1	1.945909	-3.131339	1.853707
	1	1.987275	-4.030249	0.759986		1	-1.624758	-1.261337	3.575407
	1	-0.912282	-2.430978	-2.116853		1	-0.195151	-4.034176	0.532702
	1	-0.633549	-3.723400	0.257118		1	-2.410444	-2.871842	1.549095
	53	3.564102	-0.083202	-0.765671		53	-0.683970	-2.035739	-1.623341
	6	-0.097906	1.166299	-1.488765		6	2.350352	-0.660649	-0.612807
	8	-0.160948	0.493151	-2.519199		8	2.618802	-1.840544	-0.394786
	8	-0.531795	2.464072	-1.415079		8	3.048963	0.123793	-1.489591
	6	-1.114026	3.144705	-2.573471		6	4.187932	-0.415768	-2.243220
	6	-0.076025	3.265374	-3.694932		6	-2.196801	2.686048	-0.439543
	1	0.192564	2.281351	-4.079096		6	-3.528401	2.937819	-0.063835
	1	-0.481662	3.871115	-4.512430		6	-1.595674	3.568127	-1.357221
	1	0.828222	3.756435	-3.321763		6	-4.231560	4.019105	-0.594580
	6	-2.387247	2.425361	-3.035749		1	-4.011765	2.286101	0.655176
	1	-2.149907	1.440912	-3.438735		6	-2.299882	4.647279	-1.890971
	1	-3.080685	2.309674	-2.196448		1	-0.568895	3.396859	-1.669089
	1	-2.885562	3.017454	-3.810825		6	-3.623646	4.878349	-1.512358
	6	-1.460251	4.526340	-2.007885		1	-5.258227	4.193770	-0.283391
	1	-0.560755	5.026865	-1.636963		1	-1.813279	5.306230	-2.605324
	1	-1.908862	5.152178	-2.785466		1	-4.173724	5.719636	-1.924755
	1	-2.172265	4.435165	-1.181943		6	-3.175944	-0.203338	0.119945
	6	-3.190073	1.025204	1.291310		6	-3.969716	-0.583347	1.218102
	6	-3.134584	2.405699	1.569898		6	-3.759400	-0.250597	-1.159859
	6	-4.455509	0.451018	1.058830		6	-5.290652	-1.001008	1.047827
	6	-4.290845	3.183645	1.596400		1	-3.549059	-0.533113	2.218591

Species	Cartesian coordinates				Species	Cartesian coordinates			
	1	-2.176988	2.877231	1.763286		6	-5.079102	-0.666403	-1.330033
	6	-5.609778	1.230229	1.091334		1	-3.167760	0.044257	-2.018405
	1	-4.540959	-0.610135	0.862681		6	-5.850999	-1.047927	-0.230051
	6	-5.535637	2.599997	1.355636		1	-5.882036	-1.284409	1.914712
	1	-4.217359	4.246407	1.809705		1	-5.505541	-0.694157	-2.329257
	1	-6.574115	0.762410	0.912974		1	-6.877747	-1.375704	-0.367025
	1	-6.438340	3.203758	1.378630		6	3.725767	-1.557498	-3.155654
	6	-2.496378	-2.096695	0.442402		1	3.376543	-2.406271	-2.568745
	6	-2.844059	-3.131703	1.332769		1	2.909225	-1.218932	-3.800695
	6	-3.070872	-2.090481	-0.844323		1	4.556826	-1.878126	-3.793229
	6	-3.732322	-4.133739	0.943714		6	4.640468	0.781970	-3.085216
	1	-2.420652	-3.140678	2.333398		1	4.982898	1.602970	-2.448351
	6	-3.972003	-3.085518	-1.221222		1	5.467881	0.489541	-3.738887
	1	-2.817515	-1.287243	-1.529855		1	3.818807	1.145179	-3.709503
	6	-4.300073	-4.113310	-0.333096		6	5.305044	-0.852776	-1.287851
	1	-3.989902	-4.924924	1.642111		1	4.987722	-1.703638	-0.685265
	1	-4.415969	-3.059240	-2.212345		1	6.192954	-1.135455	-1.863510
	1	-4.995527	-4.891798	-0.632799		1	5.578236	-0.028581	-0.621239
**TS13e**	6	1.802269	0.192504	2.708248					
	6	0.498740	-0.314857	2.309870					
	6	-0.584961	0.725297	2.375092					
	6	2.167712	1.422286	2.103156					
	6	1.197613	1.781188	0.983796					
	7	1.055435	0.563048	0.176289					
	1	0.259391	-1.346823	2.524221					
	1	1.541146	2.628219	0.382140					
	6	3.337102	2.061407	2.482255					
	1	3.630569	2.994634	2.010007					
	6	4.159709	1.469108	3.453823					
	1	5.083592	1.961144	3.745063					
	6	3.821951	0.240038	4.027401					
	1	4.477132	-0.211419	4.766297					
	6	2.646132	-0.412309	3.649222					
	1	2.370483	-1.360572	4.103032					
	44	0.178988	-1.776478	-0.321437					
	6	-0.147059	2.047137	1.674957					
	6	-1.452330	1.943660	0.899679					
	6	-1.842312	0.788812	1.511695					
	1	-0.064477	2.913924	2.340234					
	1	-0.781088	0.859423	3.453381					
	6	0.275486	-3.483238	1.128803					
	6	0.613940	-3.921960	-0.186691					
	6	-1.077456	-2.984960	1.079025					
	6	-0.515798	-3.705782	-1.036741					
	6	-1.574647	-3.135181	-0.240079					
	1	0.910036	-3.549517	2.002893					
	1	1.570181	-4.321324	-0.498247					
	1	-1.640504	-2.576861	1.906399					
	1	-0.566136	-3.930839	-2.092265					
	1	-2.556996	-2.851335	-0.587998					
	53	-0.689720	-0.663894	-2.777960					
	6	2.126854	0.124132	-0.483740					
	8	2.155307	-1.071212	-0.895644					
	8	3.133103	0.990054	-0.687934					
	6	4.318007	0.658849	-1.506684					
	6	3.890257	0.328927	-2.938808					
	1	3.288969	-0.579649	-2.973386					
	1	3.303222	1.150420	-3.359776					
	1	4.780626	0.189794	-3.560951					
	6	5.117621	1.963622	-1.463400					
	1	5.384386	2.219711	-0.433728					
	1	6.038760	1.857291	-2.043845					
	1	4.534696	2.786707	-1.886468					
	6	5.102893	-0.479944	-0.848177					
	1	5.332145	-0.230692	0.192689					
	1	4.537818	-1.411934	-0.872693					
	1	6.048335	-0.626947	-1.380512					
	6	-2.016253	2.922652	-0.038954					
	6	-1.964030	4.290170	0.294520					
	6	-2.580819	2.555013	-1.272225					

Species	Cartesian coordinates				Species	Cartesian coordinates
	6	-2.496584	5.254140	-0.558794		
	1	-1.520466	4.595472	1.238446		
	6	-3.105546	3.523780	-2.128235		
	1	-2.545805	1.517418	-1.587270		
	6	-3.074290	4.873345	-1.772964		
	1	-2.459644	6.303015	-0.277156		
	1	-3.526085	3.220300	-3.082919		
	1	-3.484887	5.624891	-2.441572		
	6	-3.066021	-0.005120	1.620056		
	6	-3.289011	-0.760610	2.789717		
	6	-4.066928	-0.013273	0.629401		
	6	-4.462264	-1.493004	2.964400		
	1	-2.542002	-0.758186	3.579219		
	6	-5.238814	-0.746606	0.806354		
	1	-3.921987	0.549894	-0.283311		
	6	-5.443651	-1.490903	1.971104		
	1	-4.610807	-2.062483	3.877829		
	1	-5.994222	-0.740443	0.025473		
	1	-6.357128	-2.063835	2.101999		

Table S9. The first three vibrational frequencies for stationary points corresponding to the formation process of **CAT3**, located at IDSCRF-B3LYP/BS1 level in dioxane solvent.

Species	Frequencies (cm^{-1})			Species	Frequencies (cm^{-1})			Species	Frequencies (cm^{-1})			Species	Frequencies (cm^{-1})		
CAT1	12	23	29	CAT2	8	79	301	CAT3	5	58	168	CAT4	14	21	26
MeI	511	910	910	MeCl	710	1035	1035	COM1_a	22	42	71	TS1_a	**-341**	9	55
COM2_a	19	43	57	COM1_b	25	27	37	TS1_b	**-338**	5	17	COM2_b	12	30	31
TS1_c	**-360**	15	20	TS1_d	**-338**	8	12	Dioxane	253	271	420	PPh_3	20	21	36

Table S10. The first three vibrational frequencies for stationary points of reaction **a**, located at IDSCRF-B3LYP/BS1 level in dioxane solvent.

Species	Frequencies (cm^{-1})			Species	Frequencies (cm^{-1})			Species	Frequencies (cm^{-1})			Species	Frequencies (cm^{-1})		
R1a	47	82	109	R2a	8	103	159	P1a	30	52	56	P2a	34	41	45
TS2a	**-200**	29	33	TS3a	**-196**	30	33	TS4a	**-372**	21	26	TS5a	**-143**	31	33
TS6a	**-242**	15	26	TS7a	**-41**	30	34	TS8a	**-355**	22	41	TS9a	**-36**	29	40
TS11a	**-187**	7	35	TS12a	**-241**	21	35	TS13a	**-182**	23	38	TS14a	**-206**	17	28
INT1a	26	30	42	INT2a	15	21	35	INT3a	26	37	42	INT4a	14	30	34
INT5a	32	44	51	INT6a	24	29	38	INT8a	25	37	40	INT9a	25	36	41
INT10a	23	30	39	INT11a	30	41	51	TS10a	**-275**	17	31	TS10ax	**-216**	2	9

Table S11. The first three vibrational frequencies for stationary points of reaction **a**, located at IDSCRF-B3LYP+D3/BS1 level in dioxane solvent.

Species	Frequencies (cm^{-1})			Species	Frequencies (cm^{-1})			Species	Frequencies (cm^{-1})			Species	Frequencies (cm^{-1})		
R1a	47	83	116	R2a	6	101	159	P1a	28	49	54	P2a	31	39	43
CAT3	8	64	171	INT1a	20	29	42	INT3a	27	34	38	INT6a	25	34	39
INT8a	25	37	46	INT9a	37	43	49	INT10a	31	41	47	INT11a	29	41	49
TS3a	**-195**	27	32	TS4a	**-374**	18	22	TS5a	**-135**	23	41	TS9a	**-33**	29	36
TS11a	**-190**	27	34	TS12a	**-238**	26	34	TS13a	**-209**	28	42	TS14a	**-226**	27	42

Table S12. The first three vibrational frequencies for stationary points of reaction **b**, located at IDSCRF-B3LYP/BS1 level in dioxane solvent.

Species	Frequencies (cm^{-1})			Species	Frequencies (cm^{-1})			Species	Frequencies (cm^{-1})			Species	Frequencies (cm^{-1})		
R1b	31	42	80	R2b	21	50	61	P1b	20	34	40	P2b	20	21	33
TS2b	**-177**	15	20	TS12b	**-248**	17	32	INT8b	17	27	35	TS7b-n	**-113**	13	24
TS3b	**-204**	17	24	TS13b	**-218**	15	28	INT9b	13	28	34	TS8b-n	**-342**	27	34
TS4b	**-398**	22	29	TS14b	**-202**	20	25	INT10b	17	30	35	TS9b-n	**-251**	18	27
TS5b	**-168**	17	22	INT1b	18	22	29	INT11b	21	25	32	INT1b-n	13	17	25
TS6b	**-218**	19	22	INT2b	23	25	30	TS2b-n	**-131**	19	23	INT2b-n	24	25	31
TS7b	**-98**	17	23	INT3b	17	27	31	TS3b-n	**-211**	17	22	INT3b-n	17	23	30
TS8b	**-350**	21	26	INT4b	12	20	24	TS4b-n	**-384**	15	24	INT4b-n	11	21	27

**TS9b**	**-167**	28	30	**INT5b**	20	26	31	**TS5b-n**	**-84**	27	31	**INT5b-n**	22	27	30
**TS11b**	**-226**	13	25	**INT6b**	16	23	26	**TS6b-n**	**-265**	7	25	**INT6b-n**	11	25	28
**P3b**	19	34	37												

Table S13. The first three vibrational frequencies for stationary points of reaction **b**, located at IDSCRF-B3LYP+D3/BS1 level in dioxane solvent.

Species	Frequencies (cm^{-1})			Species	Frequencies (cm^{-1})			Species	Frequencies (cm^{-1})			Species	Frequencies (cm^{-1})		
**R1b**	22	39	80	**R2b**	22	49	61	**P1b**	16	28	42	**P2b**	16	21	35
**INT1b**	33	36	43	**INT2b**	22	28	32	**INT3b**	18	27	35	**INT6b**	19	26	37
**INT8b**	20	27	44	**INT9b**	20	26	32	**INT10b**	17	30	45	**INT11b**	17	25	32
**TS3b**	**-216**	15	28	**TS4b**	**-411**	21	25	**TS5b**	**-152**	15	22	**TS9b**	**-151**	23	29
**TS11b**	**-255**	11	28	**TS12b**	**-229**	16	28	**TS13b**	**-232**	22	22	**TS14b**	**-227**	19	20

Table S14. The first three vibrational frequencies for stationary points of reaction **c**, located at IDSCRF-B3LYP/BS1 level in dioxane solvent.

Species	Frequencies (cm^{-1})			Species	Frequencies (cm^{-1})			Species	Frequencies (cm^{-1})			Species	Frequencies (cm^{-1})		
**R2c**	35	128	130	**P1c**	21	30	37	**P2c**	21	34	37	**TS2c**	**-191**	19	24
**TS3c**	**-223**	19	28	**TS4c**	**-388**	24	31	**TS5c**	**-183**	23	31	**TS6c**	**-213**	22	24
**TS7c**	**-120**	17	24	**TS8c**	**-353**	26	38	**TS9c**	**-64**	14	30	**TS11c**	**-207**	18	36
**TS12c**	**-254**	21	34	**TS13c**	**-188**	16	26	**TS14c**	**-193**	6	21	**INT1c**	12	22	31
**INT2c**	8	20	29	**INT3c**	17	31	37	**INT4c**	8	29	32	**INT5c**	27	34	45
**INT6c**	18	22	42	**INT8c**	23	32	37	**INT9c**	20	30	42	**INT10c**	24	33	42
**INT11c**	24	27	43												

Table S15. The first three vibrational frequencies for stationary points of reaction **c**, located at IDSCRF-B3LYP+D3/BS1 level in dioxane solvent.

Species	Frequencies (cm^{-1})			Species	Frequencies (cm^{-1})			Species	Frequencies (cm^{-1})			Species	Frequencies (cm^{-1})		
**R2c**	34	127	129	**P1c**	22	25	29	**P2c**	16	23	30				
**INT1c**	36	40	45	**INT2c**	25	30	36	**INT3c**	20	34	36	**INT6c**	13	26	45
**INT8c**	27	31	41	**INT9c**	20	31	39	**INT10c**	26	32	47	**INT11c**	23	33	46
**TS3c**	**-231**	17	26	**TS4c**	**-400**	24	34	**TS5c**	**-112**	19	36	**TS9c**	**-52**	24	32
**TS11c**	**-226**	16	34	**TS12c**	**-237**	6	33	**TS13c**	**-214**	24	34	**TS14c**	**-224**	20	30

Table S16. The first three vibrational frequencies for stationary points of reaction **d**, located at IDSCRF-B3LYP/BS1 level in dioxane solvent.

Species	Frequencies (cm^{-1})			Species	Frequencies (cm^{-1})			Species	Frequencies (cm^{-1})			Species	Frequencies (cm^{-1})		
**R2d**	14	136	191	**P1d**	20	31	56	**P2d**	21	37	47	**TS2d**	**-201**	21	25
**TS3d**	**-214**	19	28	**TS4d**	**-391**	24	30	**TS5d**	**-104**	25	38	**TS6d**	**-269**	20	29
**TS7d**	**-163**	20	28	**TS8d**	**-357**	27	39	**TS9d**	**-44**	22	26	**TS11d**	**-198**	18	32
**TS12d**	**-272**	10	37	**TS13d**	**-186**	16	32	**TS14d**	**-188**	15	27	**INT1d**	11	26	32
**INT2d**	20	23	38	**INT3d**	21	30	38	**INT4d**	13	29	34	**INT5d**	31	32	44
**INT6d**	20	24	44	**INT8d**	23	27	33	**INT9d**	25	35	41	**INT10d**	29	33	37
**INT11d**	24	33	43												

Table S17. The first three vibrational frequencies for stationary points of reaction **d**, located at IDSCRF-B3LYP+D3/BS1 level in dioxane solvent.

Species	Frequencies (cm^{-1})			Species	Frequencies (cm^{-1})			Species	Frequencies (cm^{-1})			Species	Frequencies (cm^{-1})		
**R2d**	13	134	190	**P1d**	22	30	57	**P2d**	16	40	47				
**INT1d**	13	19	38	**INT2d**	27	28	39	**INT3d**	24	35	45	**INT6d**	18	25	47
**INT8d**	21	32	36	**INT9d**	18	38	41	**INT10d**	20	36	42	**INT11d**	31	41	50
**TS3d**	**-214**	11	28	**TS4d**	**-413**	27	35	**TS5d**	**-83**	21	34	**TS9d**	**-33**	26	33
**TS11d**	**-222**	12	30	**TS12d**	**-265**	18	38	**TS13d**	**-215**	21	43	**TS14d**	**-221**	22	32

Table S18. The first three vibrational frequencies for stationary points of reaction **e**, located at IDSCRF-B3LYP/BS1 level in dioxane solvent.

Species	Frequencies (cm^{-1})	Species	Frequencies (cm^{-1})	Species	Frequencies (cm^{-1})	Species	Frequencies (cm^{-1})
**R2e**	11 45 53	**P1e**	17 34 38	**P2e**	17 25 29	**TS2e**	**-177** 15 20
**TS3e**	**-193** 19 28	**TS4e**	**-398** 22 25	**TS5e**	**-135** 27 30	**TS6e**	**-294** 16 24
**TS7e**	**-109** 20 23	**TS8e**	**-352** 23 26	**TS9e**	**-87** 20 21	**TS11e**	**-215** 18 22
**TS12e**	**-249** 16 21	**TS13e**	**-198** 15 21	**TS14e**	**-198** 22 28	**INT1e**	10 15 26
**INT2e**	14 24 29	**INT3e**	17 25 34	**INT4e**	6 20 23	**INT5e**	18 27 33
**INT6e**	15 22 26	**INT8e**	18 23 33	**INT9e**	24 31 33	**INT10e**	19 27 33
**INT11e**	22 26 34						

Table S19. The first three vibrational frequencies for stationary points of reaction **e**, located at IDSCRF-B3LYP+D3/BS1 level in dioxane solvent.

Species	Frequencies (cm^{-1})	Species	Frequencies (cm^{-1})	Species	Frequencies (cm^{-1})	Species	Frequencies (cm^{-1})
**R2e**	12 44 52	**P1e**	14 35 40	**P2e**	12 24 31		
**INT1e**	19 25 32	**INT2e**	22 27 30	**INT3e**	16 24 33	**INT6e**	18 23 33
**INT8e**	15 26 32	**INT9e**	21 26 30	**INT10e**	25 29 37	**INT11e**	22 24 39
**TS3e**	-209 26 32	**TS4e**	-412 19 23	**TS5e**	-117 26 27	**TS9e**	-72 19 27
**TS11e**	-233 18 27	**TS12e**	-238 30 35	**TS13e**	-220 23 29	**TS14e**	-230 12 24

Table S20. The total energies (*E*: a.u.), zero-point energies (*ZPE*: kcal·mol^{-1}) and Gibbs free energies [*G* and *G*(sol,323K): a.u.] for stationary points corresponding to the formation process of **CAT3**, located at IDSCRF-B3LYP/BS1 level in dioxane solvent.

Species	*E*	*ZPE*	*G*	*G*(sol,323K)
**CAT2**	-5096.57591	53.37975	-5096.52560	-5096.51643
**CAT3**	-11555.91997	53.11479	-11555.87233	-11555.86022
**CAT4**	-13628.60494	399.94132	-13628.04565	-13628.04420
**COM1_a**	-12056.04413	77.25515	-12055.96300	-12055.95549
**TS1_a**	-12055.98802	75.99723	-12055.90869	-12055.90173
**COM2_a**	-12056.04486	77.77019	-12055.96376	-12055.95708
**COM1_b**	-12363.71775	155.10742	-12363.52175	-12363.51573
**TS1_b**	-12363.65809	153.39877	-12363.47017	-12363.46565
**COM2_b**	-12363.72085	155.58957	-12363.52572	-12363.51987
**TS1_c**	-14128.57567	421.94681	-14127.98619	-14127.98480
**TS1_d**	-14436.24495	499.43993	-14435.54643	-14435.54801
**MeI**	-6959.44805	23.09248	-6959.43599	-6959.42579
**MeCl**	-500.11354	23.79091	-500.09826	-500.08882
**Dioxane**	-307.67035	76.93727	-307.57614	-307.56751
**PPh_3**	-1036.32439	171.69858	-1036.09754	-1036.09076

Table S21. The total energies (*E*: a.u.), zero-point energies (*ZPE*: kcal·mol^{-1}) and Gibbs free energies [*G* and *G*(sol,363K): a.u.] for stationary points of reaction **a**, located at IDSCRF-B3LYP/BS1 level in dioxane solvent.

Species	*E*	*ZPE*	*G*	*G*(sol,363K)
**R1a**	-669.07116	130.01256	-668.90247	-668.90157
**R2a**	-234.62057	89.23856	-234.51217	-234.50997
**CAT3**	-11555.91997	53.11479	-11555.87233	-11555.86441
**P1a**	-903.79319	222.87464	-903.48661	-903.48973
**P2a**	-903.75886	222.79900	-903.45305	-903.45670

**TS2a**	-12459.61722	275.44153	-12459.23884	-12459.24680
**INT1a**	-12459.64710	276.05877	-12459.26783	-12459.27550
**TS3a**	-12459.62120	275.15347	-12459.24297	-12459.25037
**INT2a**	-12459.63180	276.30584	-12459.25474	-12459.26337
**TS4a**	-12459.59973	274.25362	-12459.22515	-12459.23330
**INT3a**	-12459.65120	276.21952	-12459.27051	-12459.27737
**TS5a**	-12459.64302	274.98728	-12459.26438	-12459.27226
**TS6a**	-12459.60994	275.03484	-12459.23306	-12459.24105
**INT4a**	-12459.64071	276.26297	-12459.26221	-12459.27023
**TS7a**	-12459.61830	274.96993	-12459.24031	-12459.24820
**INT5a**	-12459.65695	276.94789	-12459.27426	-12459.28143
**TS8a**	-12459.60931	274.51194	-12459.23100	-12459.23752
**INT6a**	-12459.64901	275.95260	-12459.26985	-12459.27760
**TS9a**	-12459.63694	275.24295	-12459.25685	-12459.26397
**TS10a**	-12224.95821	182.12510	-12224.72047	-12224.72260
**TS10ax**	-12459.57346	272.07779	-12459.21193	-12459.22280
**TS11a**	-12459.61814	274.50506	-12459.24351	-12459.25167
**INT8a**	-12459.64918	276.10120	-12459.26980	-12459.27806
**TS12a**	-12459.60088	275.24550	-12459.22122	-12459.22856
**INT9a**	-12459.64154	276.42355	-12459.26047	-12459.26743
**TS13a**	-12459.61285	274.52196	-12459.23664	-12459.24358
**INT10a**	-12459.65217	276.15393	-12459.27330	-12459.27987
**TS14a**	-12459.62503	275.20293	-12459.24788	-12459.25479
**INT11a**	-12459.64068	275.51091	-12459.26116	-12459.26863

Table S22. The total energies (*E*: a.u.), zero-point energies (*ZPE*: kcal·mol^{-1}) and Gibbs free energies [*G* and *G*(sol,363K): a.u.] for stationary points of reaction **a**, located at IDSCRF-B3LYP+D3/BS1 level in dioxane solvent.

**Species**	***E***	***ZPE***	***G***	***G*(sol,363K)**
**R1a**	-669.09372	130.03893	-668.92489	-668.92361
**R2a**	-234.62758	89.28750	-234.51943	-234.51637
**CAT3**	-11555.92787	53.07083	-11555.87985	-11555.87542
**P1a**	-903.83944	223.26331	-903.53217	-903.53483
**P2a**	-903.80030	222.95666	-903.49428	-903.49715
**INT1a**	-12459.72590	276.88420	-12459.34494	-12459.35237
**TS3a**	-12459.69658	275.63150	-12459.31733	-12459.32501
**TS4a**	-12459.67466	274.53506	-12459.29961	-12459.30777
**INT3a**	-12459.73436	276.94457	-12459.35177	-12459.35873
**TS5a**	-12459.72884	275.69353	-12459.34829	-12459.35467
**INT6a**	-12459.73063	276.54906	-12459.34949	-12459.35610
**TS9a**	-12459.72098	275.86665	-12459.33940	-12459.34693
**TS11a**	-12459.69344	274.74611	-12459.31553	-12459.32206
**INT8a**	-12459.73097	276.71624	-12459.34876	-12459.35462
**TS12a**	-12459.68259	275.70770	-12459.30159	-12459.30838
**INT9a**	-12459.72483	276.91233	-12459.34181	-12459.34810

**TS13a**	-12459.68917	275.06726	-12459.31040	-12459.31766
**INT10a**	-12459.73280	276.63665	-12459.35109	-12459.35787
**TS14a**	-12459.70696	275.89320	-12459.32579	-12459.33238
**INT11a**	-12459.72326	276.15172	-12459.34228	-12459.34974

Table S23. The total energies (*E*: a.u.), zero-point energies (*ZPE*: kcal·mol^{-1}) and Gibbs free energies [*G* and *G*(sol,333K): a.u.] for stationary points of reaction **b**, located at IDSCRF-B3LYP/BS1 level in dioxane solvent.

**Species**	***E***	***ZPE***	***G***	***G*(sol,333K)**
**R1b**	-787.03072	182.68080	-786.78263	-786.77800
**R2b**	-536.28617	96.14506	-536.17216	-536.16644
**CAT3**	-11555.91997	53.11479	-11555.87233	-11555.86125
**P1b**	-1323.41472	282.18821	-1323.02282	-1323.02106
**P2b**	-1323.39194	282.52017	-1323.00079	-1322.99953
**P3b**	-1323.39520	282.00607	-1323.00417	-1323.00307
**TS2b**	-12879.24221	334.44959	-12878.78152	-12878.78246
**INT1b**	-12879.27611	335.49011	-12878.81175	-12878.81262
**TS3b**	-12879.24772	334.53085	-12878.78513	-12878.78557
**INT2b**	-12879.26507	336.06516	-12878.79897	-12878.79979
**TS4b**	-12879.22944	334.49839	-12878.76441	-12878.76503
**INT3b**	-12879.28203	335.09684	-12878.81763	-12878.81757
**TS5b**	-12879.25004	334.05180	-12878.78874	-12878.78987
**TS6b**	-12879.23060	334.40629	-12878.76865	-12878.76976
**INT4b**	-12879.26437	335.70132	-12878.80082	-12878.80205
**TS7b**	-12879.23862	334.37641	-12878.77606	-12878.77697
**INT5b**	-12879.28261	336.34733	-12878.81509	-12878.81658
**TS8b**	-12879.24112	334.02372	-12878.77739	-12878.77830
**INT6b**	-12879.28410	334.93968	-12878.82041	-12878.82141
**TS9b**	-12879.24560	334.76596	-12878.78013	-12878.78046
**TS11b**	-12879.24719	334.52643	-12878.78433	-12878.78481
**INT8b**	-12879.28059	335.90403	-12878.81405	-12878.81465
**TS12b**	-12879.22825	334.69830	-12878.76366	-12878.76494
**INT9b**	-12879.26973	335.63783	-12878.80456	-12878.80569
**TS13b**	-12879.23881	334.65192	-12878.77480	-12878.77572
**INT10b**	-12879.28113	335.91929	-12878.81465	-12878.81399
**TS14b**	-12879.24430	334.75413	-12878.78010	-12878.78128
**INT11b**	-12879.26501	334.90353	-12878.80080	-12878.80182
**TS2b-n**	-12879.24450	334.81745	-12878.78129	-12878.78252
**INT1b-n**	-12879.27271	335.97683	-12878.80870	-12878.81001
**TS3b-n**	-12879.24511	334.44490	-12878.78311	-12878.78389
**INT2b-n**	-12879.26542	335.96238	-12878.79962	-12878.80068
**TS4b-n**	-12879.22848	334.28438	-12878.76508	-12878.76526
**INT3b-n**	-12879.28213	335.11474	-12878.81768	-12878.81811
**TS5b-n**	-12879.27016	334.95260	-12878.80494	-12878.80639

**TS6b-n**	-12879.23890	334.24821	-12878.77427	-12878.77886
**INT4b-n**	-12879.26997	335.88095	-12878.80602	-12878.80681
**TS7b-n**	-12879.24161	334.35650	-12878.77978	-12878.78086
**INT5b-n**	-12879.28366	336.14528	-12878.81687	-12878.81722
**TS8b-n**	-12879.24282	334.13971	-12878.77837	-12878.77969
**INT6b-n**	-12879.28111	334.85015	-12878.81823	-12878.81941
**TS9b-n**	-12879.25858	334.82936	-12878.79416	-12878.79478

Table S24. The total energies (*E*: a.u.), zero-point energies (*ZPE*: kcal·mol^{-1}) and Gibbs free energies [*G* and *G*(sol,333K): a.u.] for stationary points of reaction **b**, located at IDSCRF-B3LYP+D3/BS1 level in dioxane solvent.

**Species**	***E***	***ZPE***	***G***	***G*(sol,333K)**
**R1b**	-787.06620	183.01817	-786.81765	-786.81259
**R2b**	-536.29782	96.15205	-536.18374	-536.17743
**CAT3**	-11555.92787	53.07083	-11555.87985	-11555.87201
**P1b**	-1323.48227	282.73996	-1323.08964	-1323.08868
**P2b**	-1323.45338	282.98918	-1323.06143	-1323.06044
**INT1b**	-12879.38020	336.60610	-12878.90982	-12878.90996
**TS3b**	-12879.34528	335.19244	-12878.88088	-12878.88208
**INT2b**	-12879.36573	336.92585	-12878.89738	-12878.89807
**TS4b**	-12879.33161	335.24186	-12878.86482	-12878.86521
**INT3b**	-12879.38844	336.05128	-12878.92157	-12878.92195
**TS5b**	-12879.35735	334.89914	-12878.89337	-12878.89439
**INT6b**	-12879.39039	335.95365	-12878.92353	-12878.92372
**TS9b**	-12879.35326	335.49189	-12878.88648	-12878.88681
**TS11b**	-12879.34710	335.34492	-12878.88152	-12878.88283
**INT8b**	-12879.38728	336.91012	-12878.91697	-12878.91689
**TS12b**	-12879.33287	335.57367	-12878.86596	-12878.86673
**INT9b**	-12879.37407	336.39196	-12878.90707	-12878.90808
**TS13b**	-12879.34065	335.52633	-12878.87371	-12878.87397
**INT10b**	-12879.38704	336.84753	-12878.91722	-12878.91699
**TS14b**	-12879.34702	335.58700	-12878.88128	-12878.88110
**INT11b**	-12879.36952	335.74015	-12878.90338	-12878.90305

Table S25. The total energies (*E*: a.u.), zero-point energies (*ZPE*: kcal·mol^{-1}) and Gibbs free energies [*G* and *G*(sol,323K): a.u.] for stationary points of reaction **c**, located at IDSCRF-B3LYP/BS1 level in dioxane solvent.

**Species**	***E***	***ZPE***	***G***	***G*(sol,323K)**
**R1b**	-787.03072	182.68080	-786.78263	-786.77627
**R2c**	-306.39492	59.01332	-306.33329	-306.32409
**P1c**	-1093.54242	246.49369	-1093.20165	-1093.19625
**P2c**	-1093.49843	245.44671	-1093.16101	-1093.15635
**TS2c**	-12649.37239	299.06898	-12648.96009	-12648.95618
**INT1c**	-12649.39805	299.71997	-12648.98517	-12648.98183

**TS3c**	-12649.36994	298.89766	-12648.95712	-12648.95368
**INT2c**	-12649.39343	300.23575	-12648.97936	-12648.97578
**TS4c**	-12649.35680	298.83132	-12648.94185	-12648.93827
**INT3c**	-12649.40612	299.98982	-12648.99063	-12648.98700
**TS5c**	-12649.39174	298.65317	-12648.97867	-12648.97457
**TS6c**	-12649.35963	298.57572	-12648.94874	-12648.94529
**INT4c**	-12649.39938	300.46701	-12648.98480	-12648.98138
**TS7c**	-12649.36705	298.34845	-12648.95544	-12648.95190
**INT5c**	-12649.41268	300.96017	-12648.99405	-12648.98878
**TS8c**	-12649.37017	298.61839	-12648.95521	-12648.95071
**INT6c**	-12649.41078	299.51572	-12648.99686	-12648.99177
**TS9c**	-12649.39352	298.51934	-12648.98036	-12648.97751
**TS11c**	-12649.36619	298.14605	-12648.95454	-12648.95124
**INT8c**	-12649.40117	300.41367	-12648.98363	-12648.97912
**TS12c**	-12649.34592	298.59512	-12648.93217	-12648.92886
**INT9c**	-12649.39725	300.08455	-12648.98161	-12648.97741
**TS13c**	-12649.36756	298.58606	-12648.95476	-12648.95148
**INT10c**	-12649.40296	300.07769	-12648.98707	-12648.98330
**TS14c**	-12649.38128	298.91462	-12648.96949	-12648.96583
**INT11c**	-12649.39359	299.47833	-12648.97868	-12648.97443

Table S26. The total energies (*E*: a.u.), zero-point energies (*ZPE*: kcal·mol^{-1}) and Gibbs free energies [*G* and *G*(sol,323K): a.u.] for stationary points of reaction **c**, located at IDSCRF-B3LYP+D3/BS1 level in dioxane solvent.

**Species**	***E***	***ZPE***	***G***	***G*(sol,323K)**
**R1b**	-787.06620	183.01817	-786.81765	-786.81088
**R2c**	-306.39953	58.95247	-306.33802	-306.32908
**CAT3**	-11555.92787	53.07083	-11555.87985	-11555.87090
**P1c**	-1093.59991	247.07613	-1093.25823	-1093.25224
**P2c**	-1093.54962	245.78038	-1093.21238	-1093.20777
**INT1c**	-12649.49074	300.56299	-12649.07244	-12649.06830
**TS3c**	-12649.45440	299.38930	-12649.04042	-12649.03623
**INT2c**	-12649.48338	301.19433	-12649.06504	-12649.06101
**TS4c**	-12649.44885	299.53997	-12649.03192	-12649.02847
**INT3c**	-12649.50211	300.77410	-12649.08437	-12649.08020
**TS5c**	-12649.48952	299.01543	-12649.07451	-12649.07066
**INT6c**	-12649.50581	300.33009	-12649.08980	-12649.08496
**TS9c**	-12649.48726	299.37713	-12649.07120	-12649.06629
**TS11c**	-12649.45524	298.81790	-12649.04155	-12649.03767
**INT8c**	-12649.49666	301.16821	-12649.07690	-12649.07278
**TS12c**	-12649.43666	299.15142	-12649.02281	-12649.01874
**INT9c**	-12649.49018	300.59245	-12649.07349	-12649.06974
**TS13c**	-12649.45726	299.37409	-12649.04155	-12649.03832
**INT10c**	-12649.49697	300.93250	-12649.07843	-12649.07434

**TS14c**	-12649.47076	299.98839	-12649.05389	-12649.05028
**INT11c**	-12649.48814	300.30825	-12649.07086	-12649.06706

Table S27. The total energies (*E*: a.u.), zero-point energies (*ZPE*: kcal·mol[-1]) and Gibbs free energies [*G* and *G*(sol,333K): a.u.] for stationary points of reaction **d**, located at IDSCRF-B3LYP/BS1 level in dioxane solvent.

**Species**	***E***	***ZPE***	***G***	***G*(sol,333K)**
**R2d**	-231.19157	55.93461	-231.13381	-231.12616
**P1d**	-1018.33395	243.33271	-1017.99621	-1017.99363
**P2d**	-1018.29239	242.39976	-1017.95743	-1017.95500
**TS2d**	-12574.15978	295.38001	-12573.75190	-12573.75173
**INT1d**	-12574.18392	295.83662	-12573.77615	-12573.77584
**TS3d**	-12574.16401	294.93864	-12573.75684	-12573.75676
**INT2d**	-12574.18832	295.97655	-12573.77934	-12573.77888
**TS4d**	-12574.14178	294.92407	-12573.73259	-12573.73208
**INT3d**	-12574.19475	296.02428	-12573.78432	-12573.78330
**TS5d**	-12574.17867	294.43197	-12573.77082	-12573.76963
**TS6d**	-12574.14641	295.06708	-12573.73844	-12573.73706
**INT4d**	-12574.18317	296.39376	-12573.77412	-12573.77393
**TS7d**	-12574.15998	294.79202	-12573.75263	-12573.75208
**INT5d**	-12574.19733	296.79659	-12573.78497	-12573.78360
**TS8d**	-12574.14178	294.60440	-12573.74606	-12573.73208
**INT6d**	-12574.19386	295.45481	-12573.78565	-12573.78563
**TS9d**	-12574.18063	294.86719	-12573.77201	-12573.77020
**TS11d**	-12574.15313	294.41488	-12573.74667	-12573.74668
**INT8d**	-12574.18408	295.74849	-12573.77510	-12573.77501
**TS12d**	-12574.13692	295.16723	-12573.72773	-12573.72637
**INT9d**	-12574.19277	296.84014	-12573.77965	-12573.77862
**TS13d**	-12574.15284	294.81582	-12573.74499	-12573.74416
**INT10d**	-12574.18928	296.22113	-12573.77868	-12573.77811
**TS14d**	-12574.16898	295.00965	-12573.76153	-12573.76089
**INT11d**	-12574.17924	295.59567	-12573.76956	-12573.76850

Table S28. The total energies (*E*: a.u.), zero-point energies (*ZPE*: kcal·mol[-1]) and Gibbs free energies [*G* and *G*(sol,333K): a.u.] for stationary points of reaction **d**, located at IDSCRF-B3LYP+D3/BS1 level in dioxane solvent.

**Species**	***E***	***ZPE***	***G***	***G*(sol,333K)**
**R2d**	-231.19499	55.86928	-231.13744	-231.13012
**P1d**	-1018.38928	243.93266	-1018.05016	-1018.04742
**P2d**	-1018.34188	242.75880	-1018.00643	-1018.00448
**INT1d**	-12574.26652	296.59861	-12573.85669	-12573.85629
**TS3d**	-12574.24571	295.51762	-12573.83755	-12573.83673
**INT2d**	-12574.26533	297.21571	-12573.85259	-12573.85199

**TS4d**	-12574.23468	295.74359	-12573.82240	-12573.82154
**INT3d**	-12574.28765	296.90969	-12573.87451	-12573.87319
**TS5d**	-12574.26970	295.18541	-12573.86026	-12573.85980
**INT6d**	-12574.28607	296.32459	-12573.87532	-12573.87426
**TS9d**	-12574.27098	295.81780	-12573.85945	-12573.85882
**TS11d**	-12574.23901	294.96504	-12573.83105	-12573.83032
**INT8d**	-12574.27359	296.47232	-12573.86243	-12573.86094
**TS12d**	-12574.22634	295.71629	-12573.81513	-12573.81382
**INT9d**	-12574.28462	297.39213	-12573.87064	-12573.87012
**TS13d**	-12574.24137	295.87416	-12573.82984	-12573.82855
**INT10d**	-12574.27903	297.00569	-12573.86635	-12573.86519
**TS14d**	-12574.25662	296.08142	-12573.84491	-12573.84422
**INT11d**	-12574.27112	296.48236	-12573.85872	-12573.85720

Table S29. The total energies (*E*: a.u.), zero-point energies (*ZPE*: kcal·mol^{-1}) and Gibbs free energies [*G* and *G*(sol,333K): a.u.] for stationary points of reaction **e**, located at IDSCRF-B3LYP/BS1 level in dioxane solvent.

**Species**	***E***	***ZPE***	***G***	***G*(sol,333K)**
**R2e**	-539.47998	120.09573	-539.32851	-539.32139
**P2e**	-1326.57530	306.39680	-1326.14614	-1326.14461
**P1e**	-1326.59906	305.95314	-1326.16973	-1326.16764
**TS2e**	-12882.42667	358.54337	-12881.92669	-12881.92745
**INT1e**	-12882.44921	358.95649	-12881.94939	-12881.94991
**TS3e**	-12882.43019	358.56868	-12881.92865	-12881.92966
**INT2e**	-12882.44364	359.73673	-12881.94068	-12881.94145
**TS4e**	-12882.40831	358.18431	-12881.90602	-12881.90605
**INT3e**	-12882.45950	358.87687	-12881.95719	-12881.95791
**TS5e**	-12882.44471	358.87256	-12881.94077	-12881.94119
**TS6e**	-12882.41323	358.16514	-12881.91286	-12881.91356
**INT4e**	-12882.44935	359.56169	-12881.94513	-12881.94582
**TS7e**	-12882.42222	358.29409	-12881.92149	-12881.92223
**INT5e**	-12882.46011	360.17197	-12881.95449	-12881.95486
**TS8e**	-12882.41847	357.78843	-12881.91711	-12881.91745
**INT6e**	-12882.45915	358.64676	-12881.95812	-12881.95890
**TS9a**	-12882.44140	359.10335	-12881.93706	-12881.93822
**TS11e**	-12882.42481	358.37561	-12881.92432	-12881.92494
**INT8e**	-12882.45901	359.66508	-12881.95552	-12881.95597
**TS12e**	-12882.40494	358.36386	-12881.90340	-12881.90421
**INT9e**	-12882.45270	359.62679	-12881.94810	-12881.94917
**TS13e**	-12882.41608	358.33166	-12881.91544	-12881.91646
**INT10e**	-12882.45824	359.64240	-12881.95460	-12881.95523
**TS14e**	-12882.42655	358.67152	-12881.92441	-12881.92491
**INT11e**	-12882.44020	358.68154	-12881.93797	-12881.93797

Table S30. The total energies (E: a.u.), zero-point energies (ZPE: kcal·mol^{-1}) and Gibbs free energies [G and G(sol,333K): a.u.] for stationary points of reaction **e**, located at IDSCRF-B3LYP+D3/BS1 level in dioxane solvent.

Species	*E*	*ZPE*	*G*	*G*(sol,333K)
**R2e**	-539.49436	120.11796	-539.34283	-539.33646
**P1e**	-1326.67308	306.70533	-1326.24208	-1326.24066
**P2e**	-1326.64128	306.96348	-1326.21129	-1326.20948
**INT1e**	-12882.55916	360.15967	-12882.05349	-12882.05292
**TS3e**	-12882.54177	359.71138	-12882.03548	-12882.03524
**INT2e**	-12882.55289	360.91049	-12882.04591	-12882.04648
**TS4e**	-12882.51746	358.98947	-12882.01337	-12882.01447
**INT3e**	-12882.57293	359.85366	-12882.06826	-12882.06813
**TS5e**	-12882.56029	359.77788	-12882.05400	-12882.05448
**INT6e**	-12882.57225	359.72740	-12882.06784	-12882.06836
**TS9e**	-12882.55706	360.16455	-12882.04970	-12882.04963
**TS11e**	-12882.53078	359.14732	-12882.02708	-12882.02679
**INT8e**	-12882.57005	360.63230	-12882.06342	-12882.06385
**TS12e**	-12882.51992	359.49742	-12882.01339	-12882.01294
**INT9e**	-12882.56508	360.39658	-12882.05942	-12882.05959
**TS13e**	-12882.52573	359.15994	-12882.02166	-12882.02084
**INT10e**	-12882.56982	360.63883	-12882.06246	-12882.06206
**TS14e**	-12882.53516	359.55474	-12882.03134	-12882.03205
**INT11e**	-12882.55212	359.67726	-12882.04724	-12882.04714
